# Global temporal trends and predictions in the burden of chronic kidney disease attributable to diet high in sugar-sweetened beverages: an age-period-cohort analysis for GBD 2021

**DOI:** 10.3389/fendo.2025.1660909

**Published:** 2025-09-10

**Authors:** Sijia Ma, Shuxin Song, Hui Tang, Fang-Fang He

**Affiliations:** ^1^ Department of Nephrology, Union Hospital, Tongji Medical College, Huazhong University of Science and Technology, Wuhan, China; ^2^ Department of Integrated Traditional Chinese and Western Medicine, Union Hospital, Tongji Medical College, Huazhong University of Science and Technology, Wuhan, China

**Keywords:** chronic kidney disease, sugar-sweetened beverages, mortality, age-period-cohort analysis, GBD 2021

## Abstract

**Objective:**

This study aims to evaluate temporal trends in chronic kidney disease (CKD) mortality attributable to excessive sugar-sweetened beverage (SSB) intake globally from 1990 to 2021 and to forecast future trends until 2040.

**Methods:**

Utilizing the Global Burden of Disease (GBD) 2021 dataset, we focused on the temporal trends in SSB-related CKD mortality. An age-period-cohort (APC) model was employed to analyze mortality trends by age, period, and cohort.

**Results:**

In 2021, the deaths attributable to SSB-related CKD were particularly elevated in high and middle sociodemographic index (SDI) regions. Among the countries studied, 62 had at least one million deaths in 2021, with the USA, Mexico, China, India, and Brazil being the top five. From 1990 to 2021, the net drift of SSB-related CKD mortality ranged from 0.54% (95% UI: -0.72 to 1.81) in low SDI regions to 3.83% (95% UI: 3.40 to 4.27) in high SDI regions. Local drift estimates indicated elevated mortality across all age groups. Globally, the increasing mortality of SSB-related CKD shifted towards the older population. Age effects showed a similar trend across all SDI regions, and the risk increased with age. Period effects for low-middle, middle, high-middle, and high SDI regions showed an improving trend before 2004, but an adverse trend after 2004. Low-middle, middle, and high SDI regions showed unfavorable trends in successive cohorts. Projections indicate a continuous increase in global deaths from SSB-related CKD over the next two decades.

**Conclusion:**

This study is the first to investigate the global temporal trends in the burden of SSB-related CKD. Over the past three decades, mortality from SSB-related CKD has demonstrated a generally upward trajectory. Evidence-based public health interventions and policies targeting SSB consumption and CKD management are imperative in high-burden regions to mitigate the advancing impact of SSB-related CKD on public health.

## 1 Introduction

Chronic kidney disease (CKD) poses a significant public health challenge, with diverse complications and adverse outcomes, leading to diminished quality of life, premature mortality, and substantial societal and individual burdens (1). Approximately 10% of the global population, an alarming 800 million individuals, are affected by CKD. It has emerged as one of the leading causes of death and is projected to rank fifth among causes of years of life lost worldwide by 2040 (2). A recent global forecasting study has further projected a continuing increase in CKD mortality, underscoring the urgency of preventive strategies (3). In 2015, the Sustainable Development Goals (SDGs) proposed by the United Nations sought to mitigate premature mortality resulting from non-communicable diseases (NCDs), including CKD (4). Despite ongoing efforts to address its rising incidence and growing threat to public health, the burden of CKD remains considerable (5). Among its various risk factors, unhealthy lifestyle and poor dietary habits play a critical role; importantly, these are modifiable factors that can be targeted for prevention and management (6).

Within this context, sugar-sweetened beverages (SSBs) have gained increasing attention as a potential dietary risk factor for CKD. SSBs are widely consumed worldwide and have been implicated in the development of obesity, type 2 diabetes, hypertension, and metabolic syndrome, all established risk factors for CKD (7, 8) Biological mechanisms linking sugar-sweetened beverage (SSB) intake to kidney damage include increased uric acid production, oxidative stress, inflammation, and renal hemodynamic alterations (9–11). Globally, SSB consumption has risen substantially over the past decades. The widespread availability, low cost, and aggressive marketing of SSBs amplify their public health impact, making them a modifiable yet challenging dietary risk factor (7). Consequently, curbing SSB consumption has emerged as a priority in NCD prevention strategies worldwide, highlighting the importance of policy measures, public health campaigns, and clinical counseling aimed at reducing sugar intake. Over the past decade, several studies have demonstrated a positive association between habitual SSB consumption and CKD incidence or progression (12–14).

To date, no global or regionally comprehensive epidemiological study has examined the long-term trends and demographic patterns of SSB-related CKD mortality. Existing research is largely limited in geographic scope and temporal coverage, preventing a comprehensive understanding of the global situation. This gap hampers policymakers’ ability to assess the evolving public health burden, identify high-risk populations, and implement targeted interventions. Furthermore, the absence of systematic trend analyses obscures regional disparities and emerging demographic vulnerabilities. Addressing this gap is essential not only for advancing epidemiological understanding but also for informing clinical practice and public health policy.

Therefore, this study aims to quantify the global, regional, and national trends in SSB-related CKD mortality from 1990 to 2021 using the Global Burden of Disease (GBD) 2021 dataset. We applied Joinpoint regression to assess temporal changes, Age–Period–Cohort (APC) modeling to disentangle age, period, and cohort effects, and Bayesian APC (BAPC) projections to forecast trends through 2040. By integrating these analytical approaches, our study fills a critical epidemiological void and provides actionable evidence to guide global and regional strategies for CKD prevention.

## 2 Methods

### 2.1 GBD 2021 overview

The GBD is a comprehensive international database of diseases and health conditions, published by the Institute for Health Metrics and Evaluation (IHME), a global health research center affiliated with the University of Washington. The GBD 2021 dataset is updated periodically and provides estimates based on epidemiological data spanning from 1990 to 2021. It covers 204 countries and territories and offers metrics on health loss for 371 diseases and injuries, as well as 88 risk factors. The database accounts for variations in age, sex, and periods, thereby enabling robust quantification of health losses across diverse populations. This facilitates improvements in health systems and addresses health disparities (15).

GBD estimates for CKD are derived from multiple high-quality data sources, including vital registration systems, household surveys, censuses, and published literature. These data are systematically compiled and synthesized using standardized statistical modeling approaches such as the Cause of Death Ensemble Model and DisMod-MR 2.1. These methods ensure comparability across locations and over time, and address data gaps through rigorous validation procedures. The credibility and widespread acceptance of GBD CKD estimates in epidemiological research and health policy planning are well established (16).

### 2.2 Data source

In this study, we retrieved data from the GBD 2021, which is accessible via https://vizhub.healthdata.org/gbd-results/. Our analysis focused on mortality from CKD attributable to excessive consumption of SSBs. These estimates were obtained from the Risk Factors dataset by selecting “Risk factor: Diet high in sugar-sweetened beverages” and “Cause: Chronic kidney disease.” According to the GBD database, a high intake of SSBs is defined as any intake (in grams per day) of beverages containing≥50 kcal per 226.8 g, encompassing carbonated beverages, sodas, energy drinks, and fruit drinks, but excluding the 100% fruit and vegetable juices. This exposure is defined by the GBD based on a systematic review of the scientific literature. The number of deaths attributable to this risk factor was estimated by multiplying the population attributable fraction (PAF), which varies by age, sex, location, and year. PAF is calculated based on estimates of risk exposures, relative risks, and theoretical minimum risk levels (TMRELs). The TMREL for SSB consumption was set to 0 g. The sociodemographic index (SDI) values ranged from 0 to 1, with higher values indicating a higher level of socioeconomic development. Based on the GBD 2021 SDI values, countries were classified into five quintiles: low, low-middle, middle, high-middle, and high SDI regions (15). We utilized various metrics to assess temporal trends in SSB-related CKD, including mortality, disability-adjusted life years (DALYs), all-age mortality rates, and age-standardized rates (ASRs), along with their corresponding 95% uncertainty intervals (UIs). ASRs were expressed as cases per 100,000 population, standardized by age.

### 2.3 Joinpoint regression analysis

The Joinpoint regression model, a statistical method that analyzes trends in disease rates over time using the least squares method, was employed in this study. The number of joinpoints was determined using the Monte Carlo permutation test, and the optimal number was selected based on model fit. We utilized this model to analyze global trends in SSB-related CKD mortality. The average annual percent change (AAPC) and its 95% confidence intervals (CIs) were calculated, representing weighted averages of the segment-specific trends. Trends were defined as increasing (AAPC > 0), decreasing (AAPC< 0), or stable (95% CI containing 0). This approach enabled us to identify significant years within changing trends (17).

### 2.4 Age-period-cohort model and statistical analysis

The APC model is a linear framework that quantifies the independent effects of age, period, and cohort on health outcomes. It is widely utilized to analyze trends in morbidity and mortality of chronic diseases as well as to predict future disease burdens (18). The APC analysis was implemented using a generalized linear model with a log link function:


$$\log\left({\text{λ}}_{ap}\right)=\mu +\alpha_{a}+\beta_{p}+\gamma_{c}+\epsilon_{ap},\;c=p-a$$

where λ*_ap_* denotes the mortality rate for age group *a* in period *p*, *μ* is the overall intercept, *α_a_* the age effect, *β_p_* the period effect, *γ_c_* the cohort effect for birth cohort *c*, and *ϵ_ap_* the random error term. To address the non-identifiability problem arising from the exact linear dependency cohort = period − age, sum-to-zero constraints ( $\sum \alpha_{a}=\sum \beta_{p}=\sum \gamma_{c}=0$ ) were applied to center the effects and ensure model identifiability.

The age-standardized mortality rates (ASMRs) for SSB-related CKD were categorized into 13 age groups with 5-year intervals (25-29, 30-34,…, 85-89 years), as well as consecutive 5-year periods from 1992 to 2021: 1992-1996 (median 1994), 1997-2001 (median 1999), 2002-2006 (median 2004), 2007-2011(median 2009), 2012-2016 (median 2014), and 2017-2021 (median 2019). Corresponding birth cohorts ranged from 1905-1909 to 1990-1994.

This study focuses on several key estimated parameters. Net drifts elucidate the overall annual percentage change in mortality rates by period and birth for the entire population. Local drifts indicate overall yearly percentage changes within specific age groups. Age effects reflect SSB-related CKD mortality across particular age brackets, while period effects represent variations influencing all age groups simultaneously over defined timeframes. Cohort effects refer to long-term trends in disease mortality among different birth cohorts. The results display longitudinal age curves, alongside relative risks of periods or cohorts. These curves illustrate the expected age-specific rates adjusted for temporal deviations. The rate ratio (RR) indicates the ratio of age-specific rates during a given period or cohort; an RR value > 1 signifies an elevated risk of SSB-related CKD mortality, whereas an RR value< 1 implies a decreased risk. Additionally, to forecast the mortality of SSB-related CKD from 2022 to 2040, we employed BAPC analysis, a widely recognized and validated approach for predicting future trends.

In this study, data were organized using Microsoft Excel 2019. Statistical analysis and graphical visualizations were conducted using R software (version 4.3.2) with the ggplot2 package. The Joinpoint Regression Program (Version 5.2.0.0) was used to analyze trends with the Joinpoint regression model. Parameter estimation and relevant hypothesis testing were performed using the APC web tool provided by the National Cancer Institute. The Wald chi-square test was used to assess the statistical significance of estimable parameters.

## 3 Results

### 3.1 The global burden of CKD attributable to diet high in SSBs by SDI

Over the past three decades, global deaths from CKD attributable to SSBs escalated from 1483.26 (95% UI: 689.86 to 2600.92) in 1990 to 6,782.03 (95% UI: 3266.15 to 11232.9) in 2021, representing an astonishing increase of 357.24% (95% UI: 306.17 to 413.38). The global all-age mortality rate increased from 0.028 (95% UI: 0.013 to 0.049) per 100,000 in 1990 to 0.086 (95% UI: 0.041 to 0.142) per 100,000 in 2021, with a growth of 209.04% (95% UI: 174.52 to 246.99). The global ASMRs rose from 0.045 (95% UI: 0.021 to 0.078) per 100,000 in 1990 to 0.082 (95% UI: 0.04 to 0.136) per 100,000 in 2021, showing an increase of 83.98% (95% UI: 64.85 to 105.82). Additionally, the net drifts remained consistently positive globally at 2.65 (95% UI: 2.42 to 2.89), signifying increased SSB-related CKD mortality throughout the study period (**Table 1**, **Supplementary Appendix S1**).

**Table 1:** Trends in the mortality of SSB-related CKD by SDI from 1999 to 2021.

Metrics	Global		High SDI		High-middle SDI		Middle SDI		Low-middle SDI		Low SDI	
	1990	2021	1990	2021	1990	2021	1990	2021	1990	2021	1990	2021
Population												
Number,n×1,000,000	5333.62 (5231.04, 5444.65)	7891.35 (7666.73, 8131.22)	879.53 (857.73, 901.2)	1094.05 (1063.56, 1125)	1063.53 (1028.13, 1100.54)	1304.03 (1251.36, 1359.98)	1722.91 (1667.96, 1774.14)	2448.54 (2353.58, 2542.11)	1161.41 (1120.14, 1200.68)	1921.11 (1821.41, 2023.27)	501.3 (487.91, 514.76)	1117.38 (1068.35, 1166.4)
Percentage of global,%	100	100	16.49	13.86	19.94	16.52	33.24	31.03	21.78	24.34	9.4	14.16
Deaths												
Number	1483.26 (689.86, 2600.92)	6782.03 (3266.15, 11232.9)	627.65 (291.53, 1071.7)	2929.07 (1428.19, 4867.13)	342.5 (153.27, 603.19)	1084.17 (513.35, 1825.56)	351.72 (163.77, 610.36)	2063.07 (972.53, 3420.81)	111.38 (50.76, 198.39)	580.61 (258.71, 976.68)	47.75 (20.73, 84.97)	118.55 (51.74, 204.9)
Percentage of global,%	100	100	42.32	43.19	23.09	15.99	23.71	30.42	7.51	8.56	3.22	1.75
Percentage change of rate 1990-2021,%	357.24 (306.17, 413.38)		366.67 (288.34, 462.16)		216.54 (173.74, 273.26)		486.57 (407.06, 569.65)		421.31 (310.52, 513.69)		148.25 (102.45, 201.42)	
All-age mortality rate												
Rate per 100,000	0.028 (0.013, 0.049)	0.086 (0.041, 0.142)	0.071 (0.033, 0.122)	0.268 (0.131, 0.445)	0.032 (0.014, 0.057)	0.083 (0.039, 0.14)	0.02 (0.01, 0.035)	0.084 (0.04, 0.14)	0.01 (0.004, 0.017)	0.03 (0.013, 0.051)	0.01 (0.004, 0.017)	0.011 (0.005, 0.018)
Percentage change of rate 1990-2021,%	209.04 (174.52, 246.99)		275.17 (212.19, 351.94)		158.17 (123.25, 204.42)		312.74 (256.79, 371.19)		215.16 (148.18, 271.01)		11.37 (-9.18, 35.23)	
Age-standardized mortality rate												
Rate per 100,000	0.045 (0.021, 0.078)	0.082 (0.04, 0.136)	0.058 (0.027, 0.099)	0.131 (0.064, 0.214)	0.043 (0.019, 0.077)	0.057 (0.027, 0.097)	0.041 (0.02, 0.069)	0.082 (0.039, 0.135)	0.021 (0.01, 0.037)	0.043 (0.02, 0.072)	0.023 (0.011, 0.041)	0.026 (0.011, 0.045)
Percentage change of rate 1990-2021,%	83.98 (64.85, 105.82)		126.1 (91.93, 167.25)		32.93 (15.27, 54.76)		101.83 (77.47, 129.43)		109.79 (62.16, 146.22)		11.82 (-7.46, 33.95)	
APC model estimate												
Net drift of mortality(% per year)	2.65 (2.42, 2.89)		3.83 (3.4, 4.27)		1.56 (0.95, 2.18)		2.68 (2.29, 3.07)		2.84 (2.12, 3.55)		0.54 (-0.72, 1.81)	

The table presents global and regional estimates for SSB-related CKD mortality, including death number, all-age mortality rate, and ASMR. Additionally, it displays net drift of mortality derived from the APC model. Data are stratified by five SDI regions for 1990 and 2021, with percentage changes calculated between these years. SSB, sugar-sweetened beverage; CKD, chronic kidney disease; SDI, sociodemographic index; ASMR, age-standardized mortality rate; APC, age-period-cohort.

Among different SDI regions, the highest mortality number was observed in high SDI regions at 2929.07 (95% UI: 1428.19 to 4867.13), followed by middle SDI regions at 2063.07 (95% UI: 972.53 to 3420.81) in 2021. Together, these two regions accounted for 73.61% of global deaths attributable to SSB-related CKD. In contrast, low SDI regions had fewer deaths at 118.55 (95% UI: 51.74, 204.9) in 2021, with the smallest increase of 148.25% (95% UI: 102.45 to 201.42) and the least increase in all-age mortality rate at 11.37% (95% UI: -9.18 to 35.23). Middle SDI regions recorded the largest increase in deaths from 1990 to 2021, reaching 486.57% (95% UI: 407.06 to 569.65), as well as the most conspicuous increase in all-age mortality rate at 312.74% (95% UI: 256.79 to 371.19). The ASMRs were highest in high SDI regions at 0.131 (95% UI: 0.064 to 0.214) per 100,000, exhibiting the greatest growth of 126.1% (95% UI: 91.93 to 167.25), followed by low-middle SDI regions at 109.79% (95% UI: 62.16 to 146.22) and middle SDI regions at 101.83% (95% UI: 77.47 to 129.43). Low SDI regions reported the lowest ASMRs at 0.026 (95% UI: 0.011, 0.045) per 100,000 in 2021, experiencing the least pronounced increase of 11.82% (95% UI: -7.46 to 33.95). Net drift values were similar in middle and low-middle SDI regions, at 2.68% (95% UI: 2.29 to 3.07) and 2.84% (95% UI: 2.12 to 3.55), respectively. High SDI regions manifested the most remarkable annual increase, reaching 3.83% (95% UI: 3.4 to 4.27), while the increase in low SDI regions was considerably slower at 0.54% (95% UI: -0.72 to 1.81) (**Table 1**, **Supplementary Appendix S1**).

**Figure 1:**
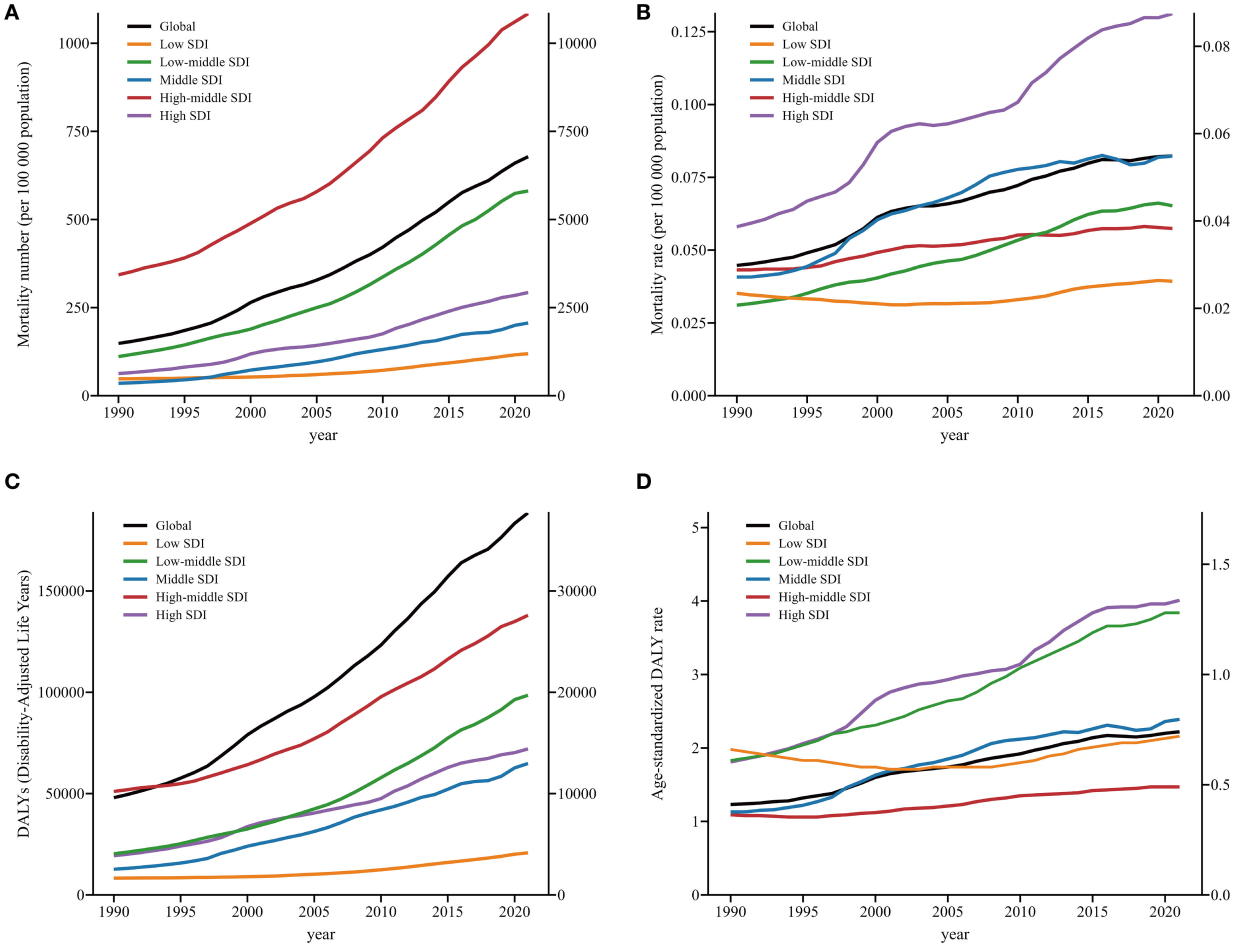
Temporal trends in mortality number, ASMRs, DALYs, and age-standardized DALY rate of SSB-related CKD from 1990 to 2021 by SDI. This figure depicts temporal patterns in key indicators of SSB-related CKD across global and SDI regions (1990–2021). **(A)** mortality number; **(B)** ASMRs; **(C)** DALYs; **(D)** age-standardized DALY rate. We adopted two different scales to distinguish the trends of each SDI region. **(A)** Y2 axis for Global, Middle SDI, High SDI; **(C)** Y2 axis for Low SDI, Low-middle SDI, High-middle SDI; **(B, D)** Y2 axis for Low SDI, Low-middle SDI. ASMRs, age-standardized mortality rates; DALYs, disability adjusted life-years; SSB, sugar-sweetened beverage; CKD, chronic kidney disease; SDI, sociodemographic index.

As illustrated in **Figure 1** and **Tables 2**, **3**, all SDI regions exhibited a generally growing trend in SSB-related CKD mortality from 1990 to 2021. The number of deaths continued to rise in five SDI regions, with relatively slower and more moderate growth observed in low SDI regions (**Figure 1A**). Regarding ASMRs, marked increases were observed in high, middle, and low-middle SDI regions, while high-middle and low SDI regions reported relatively smaller increments. Except for the periods 2010-2013 in high-middle SDI regions, 2016-2019 in middle SDI regions, and 1990-2001 in low SDI regions, ASMRs in other periods showed an upward trend (**Figure 1B**, **Table 2**). Additionally, DALYs displayed a general upward trajectory across SDI regions (**Figure 1C**). The age-standardized DALY rates exhibited trends similar to those of ASMRs (**Figure 1D**, **Table 3**).

**Table 2:** Joinpoint regression analysis for ASMRs of SSB-related CKD by SDI from 1990 to 2021.

Joinpoint model	Global		High SDI		High-middle SDI		Middle SDI		Low-middle SDI		Low SDI	
	Year	APC* (95% CI)	Year	APC* (95% CI)	Year	APC* (95% CI)	Year	APC* (95% CI)	Year	APC* (95% CI)	Year	APC* (95% CI)
Trend1	1990-1994	1.6* (1.2 to 2)	1990-1997	2.7* (2.4 to 3)	1990-1995	0.3* (0 to 0.6)	1990-1995	1.6* (1 to 2.2)	1990-1994	2.1* (1.4 to 2.9)	1990-2001	-1.0* (-1.1 to -1)
Trend2	1994-1997	2.9* (1.6 to 4.3)	1997-2001	6.9* (5.7 to 8.1)	1995-2002	2.3* (2 to 2.5)	1995-2000	6.8* (6 to 7.7)	1994-1997	4.2* (1.8 to 6.5)	2001-2008	0.3* (0.1 to 0.5)
Trend3	1997-2001	5.3* (4.6 to 6)	2001-2009	0.9* (0.6 to 1.2)	2002-2006	0.2 (-0.4 to 0.9)	2000-2005	2.3* (1.5 to 3.1)	1997-2000	1.9 (-0.3 to 4.2)	2008-2011	1.6* (0.4 to 2.8)
Trend4	2001-2005	0.7* (0.1 to 1.4)	2009-2015	4.1* (3.6 to 4.6)	2006-2010	1.6* (1 to 2.3)	2005-2009	3.2* (1.9 to 4.5)	2000-2003	3.3* (1 to 5.6)	2011-2015	2.9* (2.3 to 3.5)
Trend5	2005-2015	2.0* (1.9 to 2.1)	2015-2021	0.9* (0.6 to 1.3)	2010-2013	-0.1 (-1.3 to 1.2)	2009-2016	0.9* (0.5 to 1.3)	2003-2006	1.7 (-0.5 to 4)	2015-2021	0.9* (0.7 to 1.1)
Trend6	2015-2021	0.5* (0.3 to 0.7)	\	\	2013-2016	1.4* (0.2 to 2.7)	2016-2019	-1 (-3.5 to 1.5)	2006-2015	3.2* (3 to 3.5)	\	\
Trend7	\	\	\	\	2016-2021	0.1 (-0.2 to 0.4)	2019-2021	1.9 (-0.6 to 4.6)	2015-2021	1.0* (0.7 to 1.4)	\	\
AAPC*	1990-2021	2.0* (1.8 to 2.2)	1990-2021	2.7* (2.5 to 2.9)	1990-2021	0.9* (0.7 to 1.1)	1990-2021	2.3* (2 to 2.7)	1990-2021	2.5* (2.1 to 2.9)	1990-2021	0.4* (0.3 to 0.5)

This table presents results from Joinpoint regression analyses of ASMRs in SSB-related CKD. It displays the AAPC with corresponding 95% CIs. Trends are classified as increasing (AAPC > 0), decreasing (AAPC< 0), or stable (95% CI includes 0). This analysis identifies periods with significant temporal shifts in ASMRs. Asterisks (*) indicate values significantly different from 0 at α=0.05 (p<0.05). ASMRs, age-standardized mortality rates; SSB, sugar-sweetened beverage; CKD, chronic kidney disease; SDI, sociodemographic index; AAPC, average annual percent change; CIs, confidence intervals.

**Table 3:** Joinpoint regression analysis for age-standardized DALY rate of SSB-related CKD by SDI from 1990 to 2021.

Joinpoint model	Global		High SDI		High-middle SDI		Middle SDI		Low-middle SDI		Low SDI	
	Year	APC* (95% CI)	Year	APC* (95% CI)	Year	APC* (95% CI)	Year	APC* (95% CI)	Year	APC* (95% CI)	Year	APC* (95% CI)
Trend1	1990-1994	1.2* (0.9 to 1.4)	1990-1997	2.8* (2.5 to 3)	1990-1996	-0.4* (-0.6 to -0.2)	1990-1995	1.4* (0.7 to 2.1)	1990-1994	1.7* (1.2 to 2.2)	1990-1994	-1.5* (-1.8 to -1.2)
Trend2	1994-1997	2.7* (1.8 to 3.6)	1997-2001	6.4* (5.6 to 7.3)	1996-2005	1.5* (1.4 to 1.6)	1995-2000	6.3* (5.3 to 7.3)	1994-1997	3.3* (1.7 to 4.9)	1994-2001	-1.1* (-1.3 to -1)
Trend3	1997-2001	4.6* (4.1 to 5)	2001-2009	1.2* (1 to 1.4)	2005-2010	2.2* (1.8 to 2.5)	2000-2009	2.8* (2.5 to 3.1)	1997-2007	2.4* (2.3 to 2.6)	2001-2008	0.3* (0.1 to 0.4)
Trend4	2001-2005	1.2* (0.8 to 1.7)	2009-2015	3.9* (3.5 to 4.3)	2010-2019	0.9* (0.8 to 1)	2009-2016	1.3* (0.8 to 1.8)	2007-2010	3.8* (2.3 to 5.4)	2008-2015	2.0* (1.9 to 2.2)
Trend5	2005-2016	2.0* (2 to 2.1)	2015-2021	0.6* (0.3 to 0.8)	2019-2021	0.2 (-0.9 to 1.3)	2016-2019	-0.5 (-3.3 to 2.4)	2010-2015	2.9* (2.4 to 3.4)	2015-2021	1.2* (1 to 1.3)
Trend6	2016-2019	-0.2 (-1 to 0.7)	\	\	\	\	2019-2021	3.1* (0.2 to 6.2)	2015-2021	1.3* (1.1 to 1.6)	\	\
Trend7	2019-2021	1.5* (0.6 to 2.3)	\	\	\	\	\	\	\	\	\	\
AAPC*	1990-2021	1.9* (1.8 to 2.1)	1990-2021	2.6* (2.5 to 2.8)	1990-2021	1.0* (0.9 to 1.1)	1990-2021	2.5* (2.1 to 2.9)	1990-2021	2.4* (2.2 to 2.6)	1990-2021	0.3* (0.2 to 0.4)

This table presents results from Joinpoint regression analyses of age-standardized DALY rates in SSB-related CKD. It displays the AAPC with corresponding 95% CIs. Trends are classified as increasing (AAPC > 0), decreasing (AAPC< 0), or stable (95% CI includes 0). This analysis identifies periods with significant temporal shifts in age-standardized DALY rates. Asterisks (*) indicate values significantly different from 0 at α=0.05 (p<0.05). DALYs, disability adjusted life-years; SSB, sugar-sweetened beverage; CKD, chronic kidney disease; SDI, sociodemographic index; AAPC, average annual percent change; CIs, confidence intervals.

**Supplementary Appendix S2** illustrates global mortality trends for different causes of CKD attributable to high intake of SSBs, including type 2 diabetes, hypertension, glomerulonephritis, and other unspecified causes. In 2021, globally, deaths from type 2 diabetes-related CKD attributable to SSBs were the highest at 4877.33 (95% UI: 2566.43 to 7307.8), predominantly in high and middle SDI regions. Type 2 diabetes-related CKD attributable to SSBs had the highest all-age mortality rates and ASMRs in 2021, at 0.062 (95% UI: 0.033 to 0.093) and 0.059 (95% UI: 0.031 to 0.088), respectively. From 1990 to 2021, hypertension-related CKD attributable to SSBs exhibited the largest increase in mortality, rising by 489.82% (95% UI: 387.56 to 849.69), with the highest net drift of 3.66 (95% UI: 2.78 to 4.54) (**Supplementary Appendix S2**).

### 3.2 National burden of CKD attributable to diet high in SSBs

In 2021, among 204 countries and territories, 62 recorded at least one million deaths. The top five countries were the USA at 1649.5 (95% UI: 812.35 to 2690.32), Mexico at 612.68 (95% UI: 257.03 to 1122.68), China at 541.51 (95% UI: 245.08 to 931.91), India at 422.63 (95% UI: 172.5 to 741.93), and Brazil at 413.23 (95% UI: 202.32 to 683.31), collectively accounting for 53.72% of global deaths due to SSB-related CKD. The net drifts of these five countries were all greater than zero. From 1990 to 2021, the number of deaths increased in all countries, with 82 countries having mortality rates at or above the global average. The highest increase was observed in Armenia at 3,046.42% (95% UI: 1979.52 to 5187.85), with net drift at -5.66 (95% UI: -18.72 to 9.51).

Concerning all-age mortality rates, Monaco had the highest value at 0.669 (95% UI: 0.281 to 1.22), and Armenia experienced the most significant increase in all-age mortality rate, reaching 3493.22% (95% UI: 2274.82 to 5938.73). From 1990 to 2021, 11 countries experienced a decline in all-age mortality rates.

Regarding ASMRs, Saudi Arabia had the highest value at 0.746 (95% UI: 0.322 to 1.311), which was the closest to the global average level, with a net drift estimated at 2.88 (95% UI: 1.42 to 4.35). It was followed by American Samoa at 0.687 (95% UI: 0.316 to 1.18), Northern Mariana Islands at 0.536 (95% UI: 0.247 to 0.923), Qatar at 0.502 (95% UI: 0.209 to 0.895), and Mexico at 0.483 (95% UI: 0.206 to 0.877). Armenia had the most substantial increase in ASMRs at 1912.79% (95% UI: 1226.56 to 3137.4), closely followed by Ukraine at 1804.09% (95% UI: 1121.72 to 2801.75). A total of 28 countries experienced decreases in ASMRs, notably Cyprus at -53.4% (95% UI: -73.56 to -16.68) and a net drift at -2.67 (95% UI: -15.35 to 11.91).

The USA had the highest DALYs, reaching 42,962.23 (95% UI: 21090.31 to 71722.07), followed by Mexico at 20650.41 (95% UI: 8549.55 to 38361.52), India at 16412.64 (95% UI: 6655.36 to 29395.29), China at 14917.22 (95% UI: 6796.87 to 25713.21), and Brazil at 12122.21 (95% UI: 5560.9 to 20354.76). None of the countries recorded a reduction in DALYs. The highest increase was observed in Equatorial Guinea at 2275.23% (95% UI: 1177.37 to 4191.97).

Regarding age-standardized DALY rates, 79 countries reached or exceeded the global average level. Five countries, including Saudi Arabia, American Samoa, Mexico, Northern Mariana Islands, and Ecuador, had rates more than 5 times the global average. Saudi Arabia had the highest age-standardized DALY rate at 20.29 (95% UI: 8.52 to 35.89), approximately 9 times the global average. A total of 172 countries exhibited an increase in age-standardized DALY rates, with Ghana showing the most substantial increase at 620.09% (95% UI: 316.19 to 1,026.69), accompanied by the highest net drift value at 7.79 (95% UI: -1.17 to 17.56).

**Figure 2:**
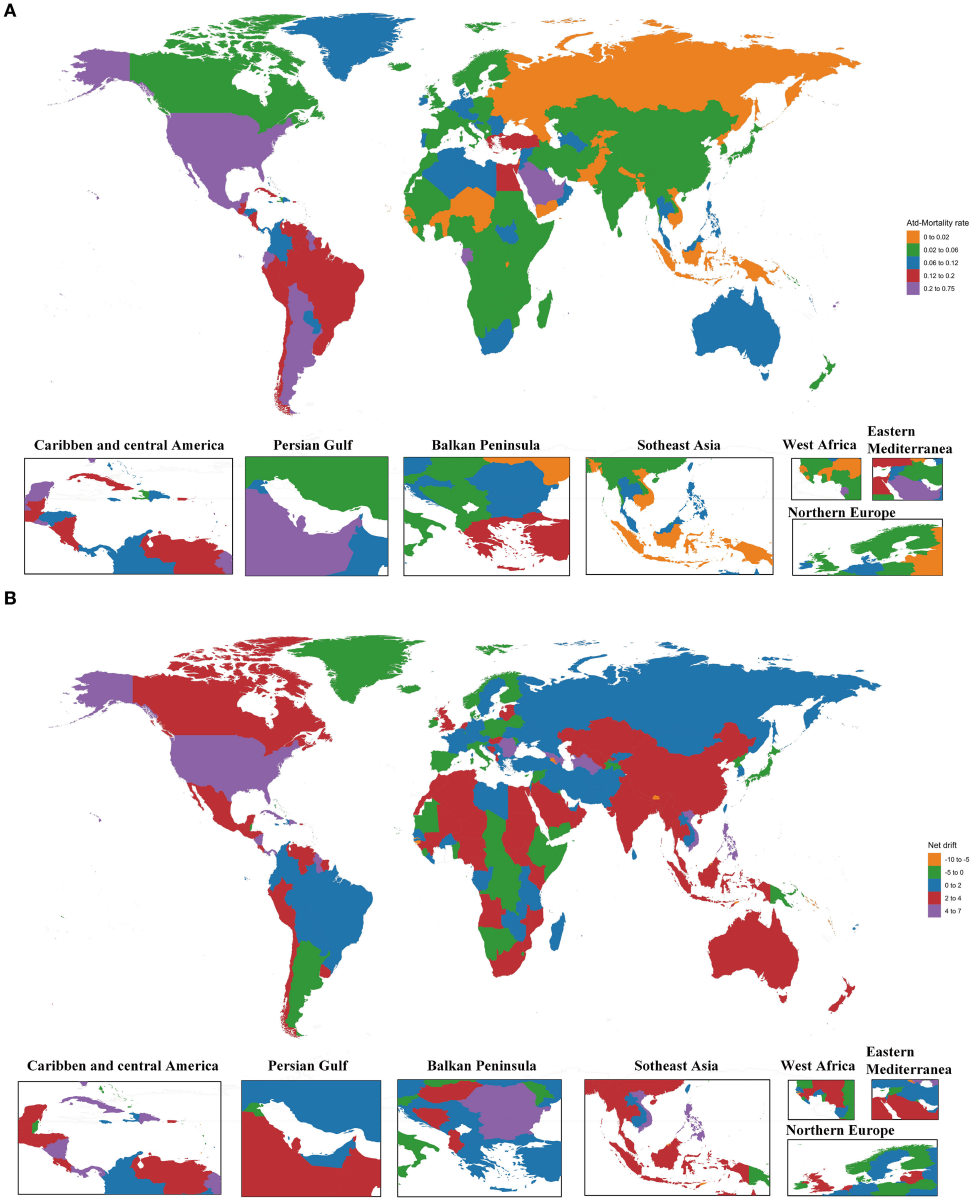
The ASMRs in 2021 and net drift of mortality from 1990 to 2021 for SSB-related CKD in 204 countries and territories. **(A)** World map of the ASMRs in 2021 for SSB-related CKD. **(B)** World map of the net drift of mortality from 1990 to 2021 for SSB-related CKD. ASMRs, age-standardized mortality rates; SSB, sugar-sweetened beverage; CKD, chronic kidney disease.

In 121 countries, the net drift was greater than 0, indicating worsening SSB-related CKD. After Ghana, Azerbaijan followed at 6.24 (95% UI: -5.56 to 19.51), Nicaragua at 6.03 (95% UI: -2.49 to 15.28), and Turkmenistan at 5.87 (95% UI: -4.25 to 17.06). Among countries with net drift less than 0, Djibouti had the smallest value at -10.16 (95% UI: -22.25 to 3.81), indicating improved SSB-related CKD mortality (**Figure 2**, **Supplementary Appendix S3**).

### 3.3 Temporal trends of CKD attributable to diet high in SSBs across different age groups

**Figure 3:**
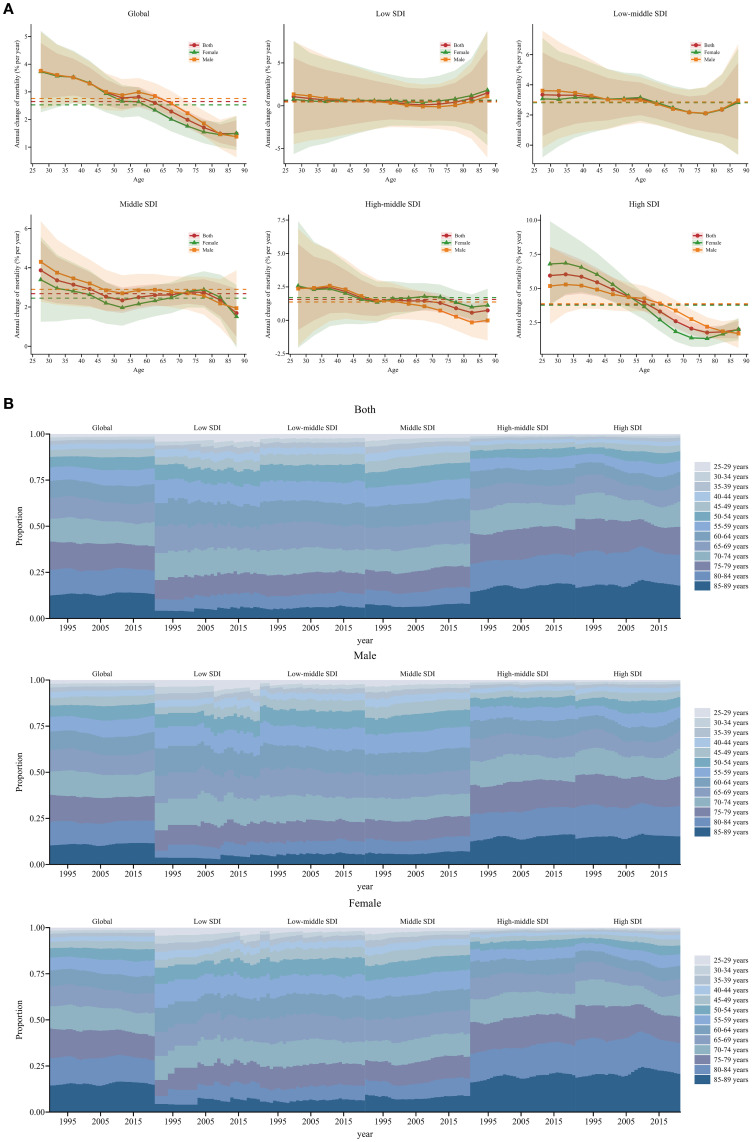
Local drift and age distribution of mortality from 1990 to 2021 for SSB-related CKD across SDI. **(A)** Local drift of mortality from 1990 to 2021 for SSB-related CKD for 13 age groups (25-29 to 85-89 years) across different SDI regions. The dots and shaded areas denote the annual percentage change of age-specific mortality and their corresponding 95% UIs. **(B)** Temporal changes in age distribution of SSB-related CKD mortality for 13 age groups (25-29 to 85-89 years) from 1990 to 2021 across different SDI regions. SSB, sugar-sweetened beverage; CKD, chronic kidney disease; SDI, sociodemographic index; UIs, uncertainty intervals.

Globally, local drifts were predominantly above zero across age groups and gradually decreased with advancing age. The annual percentage change was generally higher in males than in females. Across SDI regions, most age groups showed increasing trends in SSB-related CKD mortality, except for certain male age groups in low and high-middle SDI regions, which exhibited a downward trend. The local drift in low SDI regions fluctuated around 0, remaining relatively stable. In low-middle SDI regions, the greatest increase was observed among individuals aged 25-29 at 3.34 (95% UI: 0.61 to 6.15), then gradually decreased with age and increased slightly in the 80-84 age group; no significant gender differences were observed. In middle SDI regions, the most pronounced increase occurred in the 25-29 age group at 3.87 (95% UI: 2.39 to 5.37). The value decreased gradually until the 50-54 age group and then remained relatively constant before a significant decline was seen in the 85-89 age group. The local drift was higher in males than females from 25-29 to 65-69 age groups, but reversed from 70-74 to 80-84 age groups. In high-middle SDI regions, a decreasing trend in SSB-related CKD mortality only occurred in males aged 80-84 and 85-89, particularly in the 80-84 age group at -0.16 (95% UI: -1.24 to 0.94). Other age groups exhibited a worsening trend, with the greatest rise in the 35-39 age group at 2.47 (95% UI: 0.53 to 4.43), followed by a sharp decline with increasing age. In high SDI regions, the local drift stabilized after peaking in the 30-34 age group, with the most significant growth at 6.03 (95% UI: 4.49 to 7.59). From the 60-64 age group onwards, the value decreased significantly and then remained stable. Between 25-29 and 50-54 age groups, the annual percentage change was higher in females than males, while the opposite was true from 55-59 to 80-84 age groups (**Figure 3A**, **Supplementary Appendix S4**). The local drift of each country is displayed in **Supplementary Appendix S5**.

Temporal changes in the age distribution of SSB-related CKD mortality are illustrated in **Figure 3B**. Globally, there was a gradual shift in mortality towards the older population, especially among females, and this trend was more pronounced in high-middle and high SDI regions. Furthermore, older age groups accounted for a higher proportion of mortality, with over 50% of mortality occurring in individuals older than 70 years in high-middle and high SDI regions. In other SDI regions, more than 50% of mortality occurred in individuals older than 65 years (**Figure 3B**).

### 3.4 Age, period, and cohort effects on CKD attributable to diet high in SSBs

**Figure 4:**
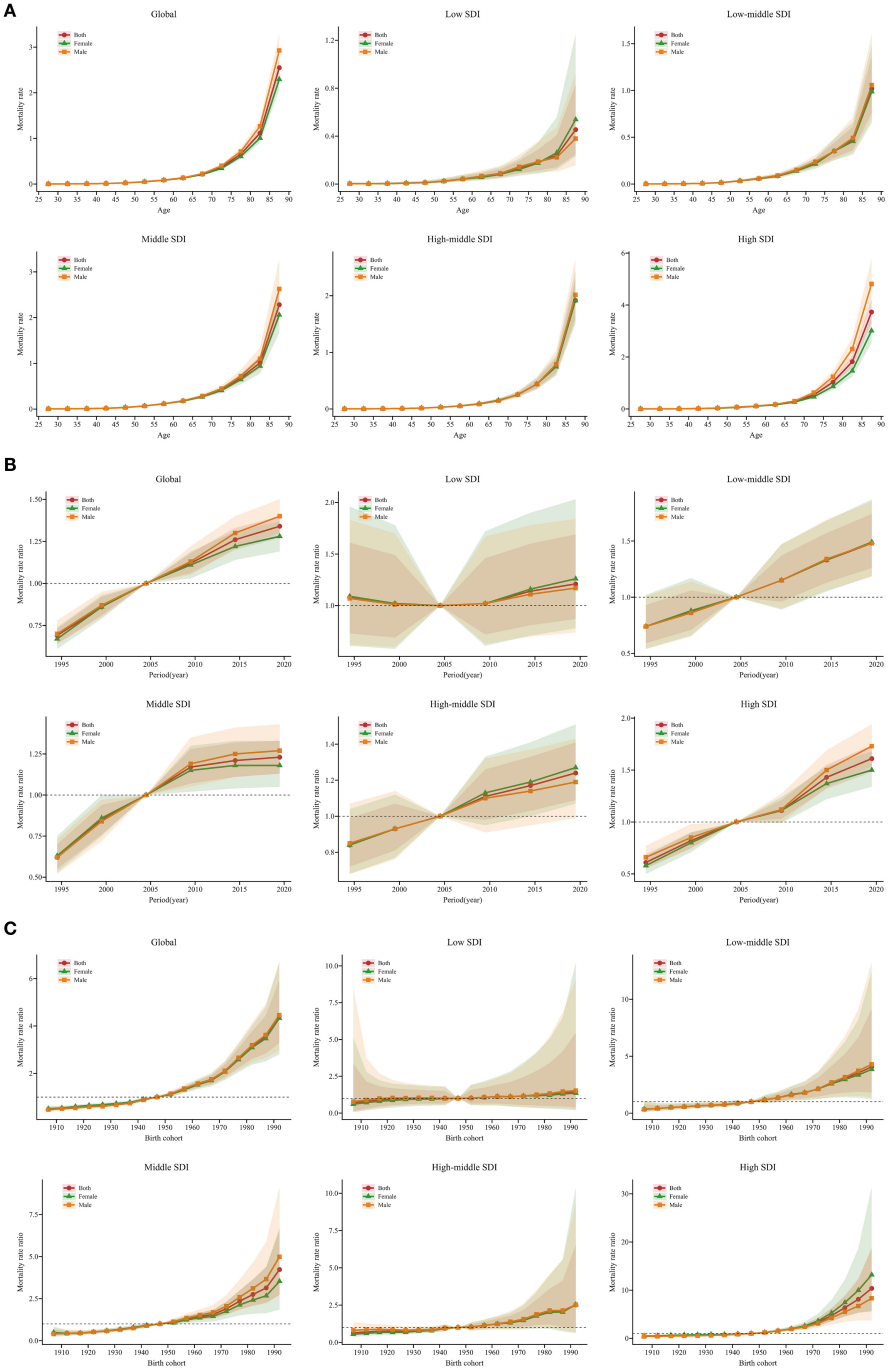
Age, period, and cohort effects on SSB-related CKD mortality across SDI from 1990 to 2021, with gender-based analysis. **(A)** Age-effect parameter estimates of SSB-related CKD mortality rate; **(B)** Period-effect parameter estimates of SSB-related CKD mortality rate; **(C)** Cohort-effect parameter estimates of SSB-related CKD mortality rate. The dots and shaded areas denote the mortality rates or rate ratios and their corresponding 95% UIs. SSB, sugar-sweetened beverage; CKD, chronic kidney disease; SDI, sociodemographic index; UIs, uncertainty intervals.

Estimates of the age, period, and birth cohort effects on SSB-related CKD mortality are presented in **Figure 4** and **Supplementary Appendix S6-S8**. Generally, the age effects exhibited similar trends globally and across SDI. The age effects remained stable up to the 55-59 age group and then increased progressively with advancing age, particularly among older populations aged 85-89. Low SDI regions showed a weaker trend compared to other SDI regions. Moreover, in the 85-89 age group, the age effects were most pronounced in high SDI regions, followed by middle SDI regions, and were weakest in low SDI regions (**Figure 4A**). Regarding period effects, low-middle, middle, high-middle, and high SDI regions exhibited similar trends, showing a positive trajectory before 2004, which then reversed into a negative trend after 2004. Among these four SDI regions, from 1992 to 1996, the most pronounced mitigation occurred in high SDI regions. The largest increase in the period effects RR was observed in high SDI regions, with relatively small increases in low, middle, and high-middle SDI regions. Additionally, in low SDI regions, the period effects fluctuated slightly above 1, especially during 2017 to 2021 (**Figure 4B**). Globally, the cohort effects showed an increasing RR with birth year: individuals born before 1945–1949 experienced declining mortality, whereas those born afterward faced worsening outcomes. Low-middle, middle, high-middle, and high SDI regions showed similar trends. In middle SDI regions, the cohort effects were more significant for males than females, while the opposite was true in high SDI regions. In low SDI regions, those born between 1905-1909 and 1925-1929 experienced a remission in SSB-related CKD mortality, whereas those born between 1950-1954 and 1990-1994 experienced a worsening trend (**Figure 4C**). Moreover, the age, period, and cohort effects on SSB-related CKD mortality for each country are shown in **Supplementary Appendix S9-S11**.

**Figure 5:**
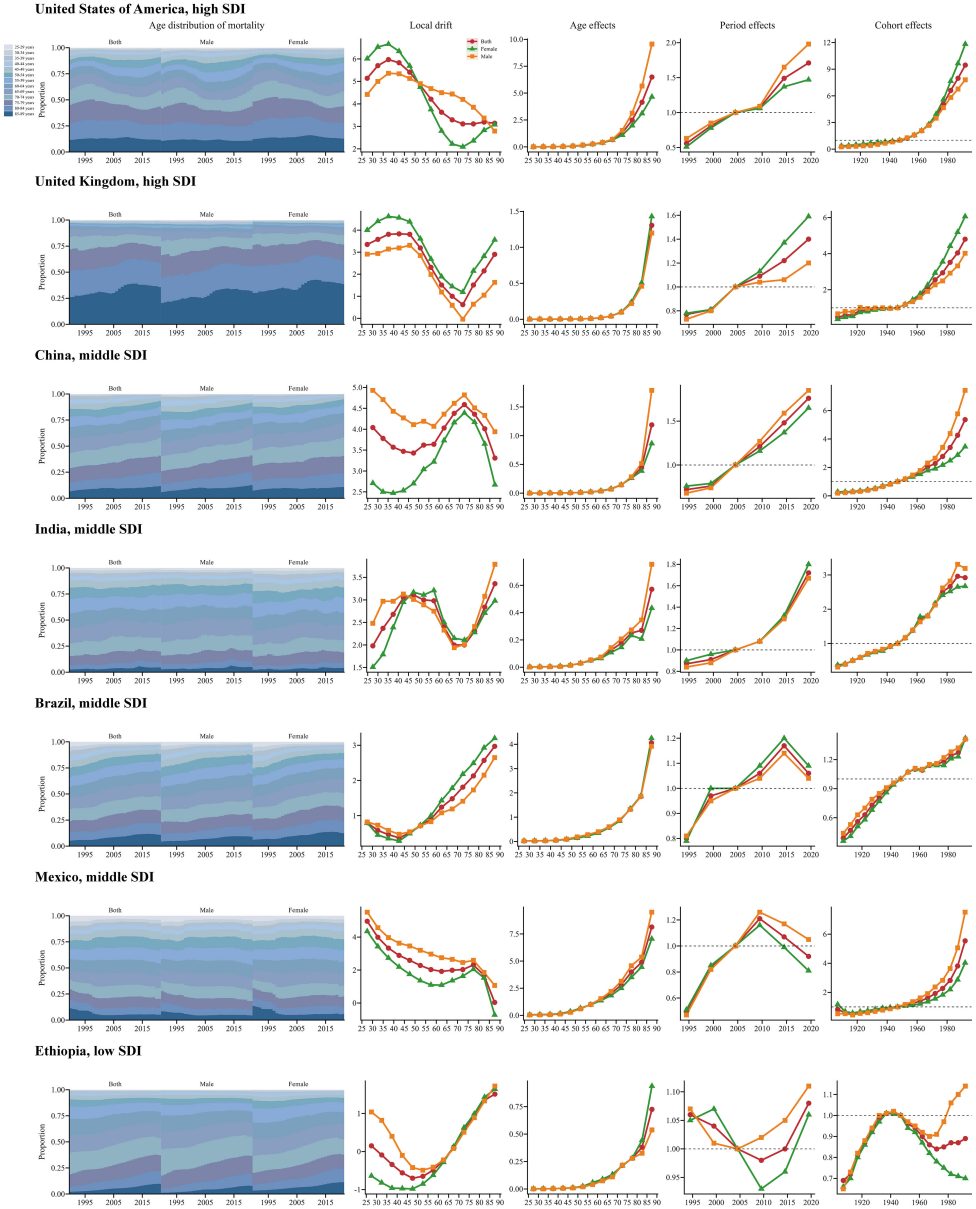
Age, period, and cohort effects on SSB-related CKD mortality in exemplary countries from 1990 to 2021, with gender-based analysis. This figure quantifies age, period, and cohort effects on SSB-related CKD mortality and depicts temporal changes in its age-distribution across 13 age groups (25-29 to 85-89 years) from 1990 to 2021. Analyses are presented for selected representative countries, including the United States of America (High SDI), the United Kingdom (High SDI), China (Middle SDI), India (Middle SDI), Brazil (Middle SDI), Mexico (Middle SDI), and Ethiopia (Low SDI). SSB, sugar-sweetened beverage; CKD, chronic kidney disease; SDI, sociodemographic index.

To more accurately characterize the global temporal trends in CKD mortality associated with SSBs, **Figure 5** highlights several representative countries across different SDI regions. The USA and the UK, as highly developed economies, exhibited mostly positive local drift values. Age effects showed a sharp increase among the elderly, while period and cohort effects progressively deteriorated over the past decade and across successive cohorts. China and India, characterized by large populations and high CKD prevalence, exhibited an unfavorable trend with no reduction in mortality across all age groups; both period and cohort effects worsened in recent years. Brazil and Mexico, despite high levels of SSB consumption, demonstrated significant improvements in period effects related to CKD mortality in recent years. Notably, in Mexico, local drift decreased overall across age groups, indicating a positive trend. Ethiopia, representing low SDI regions, exhibited a favorable local drift trend among young population, whereas the opposite trend was observed in older groups. Age effects increased with age, and period effects initially decreased but significantly worsened in recent years. However, cohort effects generally showed a positive trend (**Figure 5**).

### 3.5 The predicted results from 2022 to 2040

**Figure 6:**
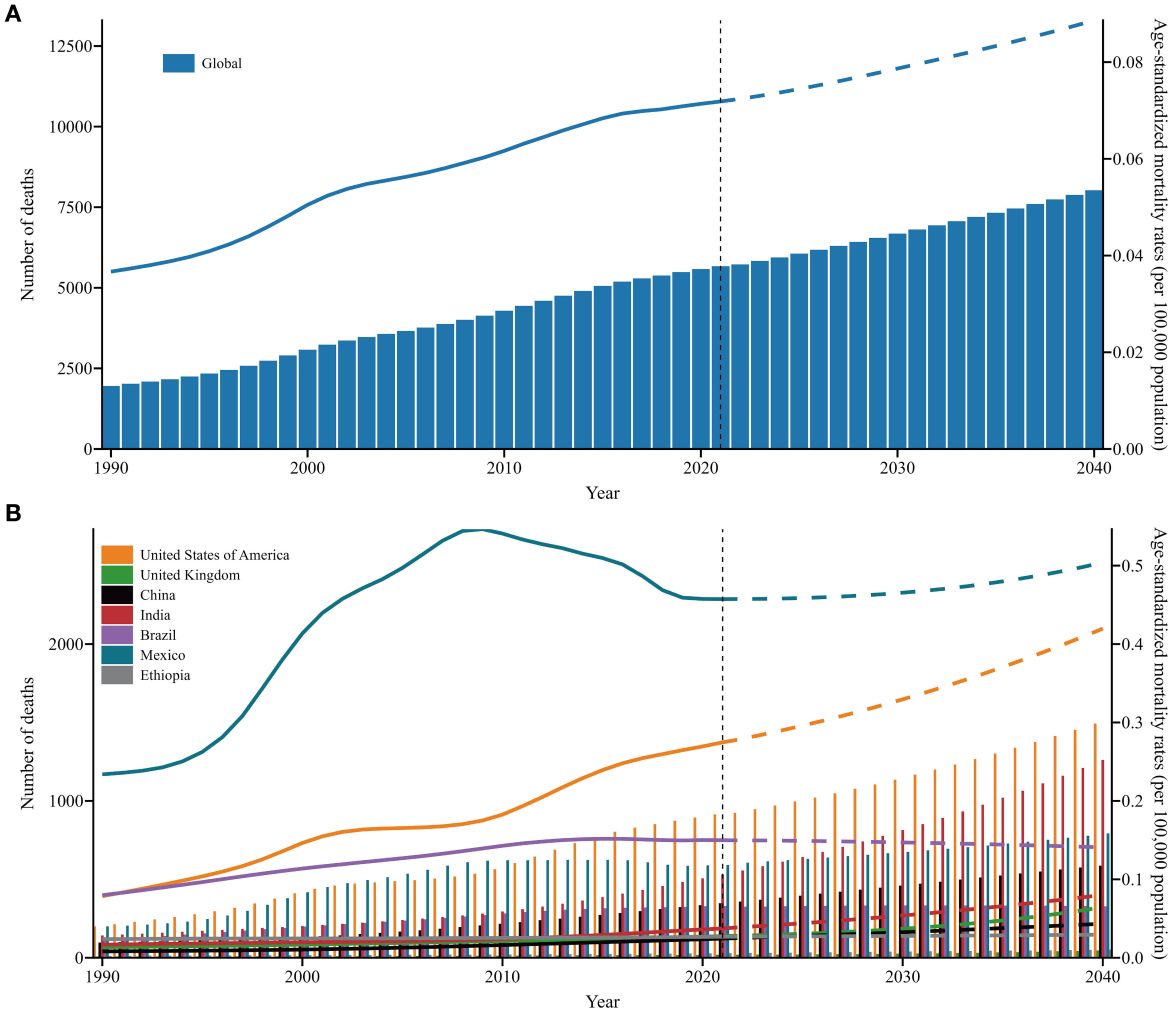
The number of deaths and ASMRs trends of SSB-related CKD across SDI and in exemplary countries from 1990 to 2021 and future forecasts from 2022 to 2040. **(A)** Global; **(B)** Exemplary countries. Y1 axis and columns correspond to number of death, and Y2 axis and lines correspond to ASMRs; the solid line represents the available data, and the dashed line represents the predicted data. ASMRs, age-standardized mortality rates; SSB, sugar-sweetened beverage; CKD, chronic kidney disease; SDI, sociodemographic index; UIs, uncertainty intervals.

Globally, projections suggest that the number of deaths and ASMRs of SSB-related CKD will continue to increase over the next two decades. Significant increases in mortality are projected in the USA and India. A comparable trend is anticipated in Mexico, China, and the UK, although the deterioration is relatively moderate. Nevertheless, the number of deaths in Brazil and Ethiopia is expected to remain stable, with no significant change over the next two decades. The ASMRs in the USA and Mexico have remained consistently high and are expected to gradually rise from 2022 to 2040, whereas those in other countries are projected to remain relatively stable (**Figure 6**, **Supplementary Appendix S12**).

## 4 Discussion

From 1990 to 2021, the mortality trend in SSB-related CKD continued to increase globally and is predicted to continue rising over the next two decades, highlighting the urgent need for effective interventions. Across different SDI regions, high and middle SDI regions emerged as the primary contributors, accounting for nearly three-quarters of the global deaths. High SDI regions had the highest ASMRs and the greatest growth, while middle SDI regions demonstrated the most significant increase in all-age mortality rates. Although mortality remained relatively stable in low SDI regions, the burden of CKD attributable to SSBs remains a significant concern. Our findings are in accordance with previous studies demonstrating an escalating burden of chronic diseases attributable to SSBs, particularly diabetes, obesity, and hyperuricemia (9, 19, 20). Notably, the burden of SSB-related CKD showed a worsening trend across all age groups, intensifying with advancing age. Additionally, period and cohort effects demonstrated adverse trends, particularly in high SDI regions. Based on these analyses, high-burden regions should formulate targeted strategies tailored to high-risk populations within their national contexts, drawing on global experiences.

It is important to note that the observed trend in CKD associated with SSBs in this study may not fully align with the prevailing assumption that higher-quality healthcare systems and medical services, typically found in higher SDI regions, can reduce disease burden. The varying trends in SSB-related CKD mortality across SDI may be attributed to differences in lifestyle and dietary patterns, as well as economic development and market transformation, leading to disparities in SSB consumption (21). Individuals in higher SDI regions are more likely to experience overnutrition, characterized by high intake of glucose, fat, and protein, which are closely associated with the onset and progression of CKD (21, 22). Additionally, in many low-middle and middle SDI countries, SSB intake is increasing as a result of widespread urbanization and economic development, which has enhanced the availability of sugary beverages (23). Despite advances in prevention, early diagnosis, management, and treatment, excessive SSB consumption remains a major driver of the burden of SSB-associated CKD.

This study examines the independent effects of age, period, and cohort on SSB-related CKD mortality. Globally, mortality from SSB-related CKD increased with age, showing a shift toward older populations. This trend is likely explained by the rapidly aging demographic structure. Epidemiological data indicate that 11% of the global population is aged 60 years or older, a figure projected to reach 22% by 2050. From 2000 to 2030, the percentage of individuals aged 65 years or older is projected to rise from 12.4% to 19.6% in the USA and from 12.6% to 20.3% in Europe (24). In China, there were 111 million individuals over 65 years old in 2010, including 19.3 million aged over 80 years. By 2050, this number is projected to reach approximately 400 million (26.9% of the total population), with those over 80 years increasing to 150 million (25). These demographic shifts are expected to drive a parallel rise in CKD prevalence among older adults (26). Aging poses challenges for the integrated management of chronic diseases, increasing the risk of CKD. Older adults have slower physiological processes, reduced metabolic rates, and declining kidney function, which limit their ability to metabolize harmful components in SSBs (27). Moreover, they may encounter chewing difficulties, making SSBs a readily accessible dietary option (28). Limited health literacy and suboptimal nutritional awareness further contribute to risk. Unfavorable period and cohort effects were also observed globally. The period effects RR has been rising over the past few years, potentially due to rapid economic growth and shifting consumption patterns. The emergence of various SSB products has fueled high consumption levels, particularly among consumers seeking trendy options (29). Advances in medical standards and diagnostic capabilities have also increased the detection rate of CKD (30). Furthermore, there was significant heterogeneity in the effects of age, period, and birth cohort on SSB-related CKD mortality across 204 countries and regions, reflecting diverse disease patterns worldwide. This heterogeneity underscores the need for tailored national health policies.

Numerous valuable lessons can be gleaned from the experiences of several exemplary countries. In Mexico and Brazil, the period effect showed a marked easing trend after 2015. In 2014, Mexico became the first country to impose a 10% SSB tax, attracting significant global attention. This policy led to a progressive decrease in SSB purchases, culminating in a 12% reduction within one year, significantly lowering the disease burden associated with SSBs and generating substantial healthcare savings (31). In the same year, the Brazilian Ministry of Health proposed implementing a standardized sugar labeling system (32). A legislative proposal to implement SSB taxation is currently under consideration in Brazil (33). Brazil’s broader health policies have garnered international recognition. Since the 1990s, Brazil has initiated the Family Health Program and the Community Health Agency program to widen access to primary healthcare and reduce health inequalities (34, 35). Additionally, the establishment of the National Health System for the comprehensive registration and identification of chronic diseases has facilitated integrated CKD management (36). In the USA, Berkeley enacted the first substantial SSB tax in 2014, resulting in a 9.6% decline in SSB sales within one year (37). More recently, in 2018, the UK introduced a tiered SSB tax based on sugar content (£0.24 per liter for drinks containing ≥8 g sugar/100 ml; £0.18 per liter for drinks containing between 5 g and<8 g sugar/100 ml) (38). The UK has also implemented labeling regulations to guide consumers toward healthier choices (39). In China, health authorities have proposed an SSB tax, while sugar-free beverages have emerged as a promising trend (40). In response to the public health threat posed by excessive SSB consumption, Indian is implementing an SSB tax, and new regulations on packaging and advertising are under consideration as part of broader health promotion strategies (41, 42). Additionally, the Indian Society of Nephrology has developed educational modules for community physicians to improve CKD management and ensure timely referral to nephrologists (43).

The present study is the first to illuminate global temporal trends in the burden of SSB-related CKD from 1990 to 2021 using the APC model. By analyzing mortality trends across different SDI regions and countries, and focusing on specific populations, this approach facilitates the formulation of tailored interventions informed by the temporal and cohort analyses. These findings provide valuable insights for policymakers worldwide, enabling them to formulate more targeted strategies to address SSB-related CKD, while also offering empirical evidence applicable to high-burden regions.

This study has several limitations. First, the exclusion of individuals under 25 years, due to GBD data constraints, may introduce selection bias and limit generalizability to younger populations. Second, our analysis was unable to differentiate CKD subtypes (e.g., obesity-related or hyperuricemic nephropathy), which limits etiological specificity. Future studies should address this heterogeneity. Third, underreporting in regions with underdeveloped vital registration systems and inter-country coding heterogeneity may affect mortality estimates, despite conducting uncertainty analyses. Fourth, residual confounding from unmeasured comorbidities and lifestyle factors remains due to the ecological design. Fifth, the APC model’s reliance on cross-sectional data rather than longitudinal data precludes causal inference for SSB-CKD associations, and any discordance with clinical studies reflects inherent methodological differences between probabilistic attribution and direct measurement. Sixth, the absence of treatment setting and disease severity stratification reduces clinical translatability. Seventh, more granular analyses by genetic profiles or beverage subtypes were unfeasible due to data limitations. Finally, although we projected CKD mortality trends up to 2040 to align with global health planning horizons, forecasting nearly two decades ahead based on 31 years of data introduces inherent uncertainty and may reduce predictive accuracy. Such projections should therefore be interpreted with caution.

In summary, our findings highlight that the global burden of SSB-related CKD remains substantial and has not been effectively alleviated. With population aging accelerating, SSB-associated CKD poses a significant burden on the elderly demographic. High-burden nations should consider formulating or optimizing SSB tax policies tailored to national contexts, aiming to relieve the disease burden. In parallel, the health sector must prioritize CKD management by enhancing medical policies and management systems to ensure standardized diagnosis and treatment, thereby effectively reducing years of life lost and preventing premature mortality. Population-level burden estimates should inform targeted clinical actions. Identifying high-risk regions and demographic groups warrants integrating systematic SSB exposure screening into primary care, enabling early detection through risk-stratified approaches. These findings should also guide updates to clinical guidelines, including strengthening SSB restriction thresholds for CKD prevention. Regions with rising mortality should be prioritized for dialysis capacity expansion. Ultimately, controlling SSB-related CKD, particularly in high-burden regions, will require sustained collaboration between policymakers and the health sector.

## Data Availability

The original contributions presented in the study are included in the article/**Supplementary Material**. Further inquiries can be directed to the corresponding authors.
