# Supplementary material for: Global temporal trends and predictions in the burden of chronic kidney disease attributable to diet high in sugar-sweetened beverages: an age-period-cohort analysis for GBD 2021
Source: Front Endocrinol (Lausanne). 2025 Sep 10;16:1660909. doi: 10.3389/fendo.2025.1660909 (PMC12457128; doi:10.3389/fendo.2025.1660909)
Supplement: Supplementary file 1 [file DataSheet1.docx]

Supplementary Material

**[Appendix 1.1](#_Toc185966437)** [The mortality number from 1990 to 2021 for SSBs-related CKD across SDI 1](#_Toc185966437)

**[Appendix 1.2](#_Toc185966438)** [The all-age mortality rate from 1990 to 2021 for SSBs-related CKD across SDI 3](#_Toc185966438)

**[Appendix 1.3](#_Toc185966439)** [The ASMRs from 1990 to 2021 for SSBs-related CKD across SDI 5](#_Toc185966439)

**[Appendix 1.4](#_Toc185966440)** [The DALYs from 1990 to 2021 for SSBs-related CKD across SDI 7](#_Toc185966440)

**[Appendix 1.5](#_Toc185966441)** [The age-standardized DALYs rate from 1990 to 2021 for SSBs-related CKD across SDI 10](#_Toc185966441)

**[Appendix 1.6](#_Toc185966442)** [The net drift of mortality from 1990 to 2021 for SSBs-related CKD across SDI 12](#_Toc185966442)

**[Appendix 2](#_Toc185966443)** [Trends in the mortality of different types of CKD attributable to excessive consumption of SSBs by SDI in 1990 and 2021 13](#_Toc185966443)

**[Appendix 3](#_Toc185966444)** [The mortality number, all-age mortality rate, ASMRs, DALYs, age-standardized DALYs rate and net drift of mortality from 1990 to 2021 for SSBs-related CKD across countries 18](#_Toc185966444)

**[Appendix 4.1](#_Toc185966445)** [The local drift of mortality from 1990 to 2021 for SSBs-related CKD across SDI ..44](#_Toc185966445)

**[Appendix 4.2](#_Toc185966446)** [The local drift of mortality from 1990 to 2021 for SSBs-related CKD across SDI in males 45](#_Toc185966446)

**[Appendix 4.3](#_Toc185966447)** [The local drift of mortality from 1990 to 2021 for SSBs-related CKD across SDI in females 46](#_Toc185966447)

**[Appendix 5](#_Toc185966448)** [The local drift of mortality from 1990 to 2021 for SSBs-related CKD across countries 47](#_Toc185966448)

**[Appendix 6.1](#_Toc185966449)** [Age effects on the mortality of SSBs-related CKD across SDI 111](#_Toc185966449)

**[Appendix 6.2](#_Toc185966450)** [Age effects on the mortality of SSBs-related CKD across SDI in males 112](#_Toc185966450)

**[Appendix 6.3](#_Toc185966451)** [Age effects on the mortality of SSBs-related CKD across SDI in females 113](#_Toc185966451)

**[Appendix 7.1](#_Toc185966452)** [Period effects on the mortality of SSBs-related CKD across SDI 114](#_Toc185966452)

**[Appendix 7.2](#_Toc185966453)** [Period effects on the mortality of SSBs-related CKD across SDI in males 115](#_Toc185966453)

**[Appendix 7.3](#_Toc185966454)** [Period effects on the mortality of SSBs-related CKD across SDI in females 116](#_Toc185966454)

**[Appendix 8.1](#_Toc185966455)** [Cohort effects on the mortality of SSBs-related CKD across SDI 117](#_Toc185966455)

**[Appendix 8.2](#_Toc185966456)** [Cohort effects on the mortality of SSBs-related CKD across SDI in males 118](#_Toc185966456)

**[Appendix 8.3](#_Toc185966457)** [Cohort effects on the mortality of SSBs-related CKD across SDI in females 119](#_Toc185966457)

**[Appendix 9](#_Toc185966458)** [Age effects on the mortality of SSBs-related CKD across countries 120](#_Toc185966458)

**[Appendix 10](#_Toc185966459)** [Period effects on the mortality of SSBs-related CKD across countries 190](#_Toc185966459)

**[Appendix 11](#_Toc185966460)** [Cohort effects on the mortality of SSBs-related CKD across countries 221](#_Toc185966460)

**[Appendix 12](#_Toc185966461)** [Prediction of mortality number and ASMRs of SSBs-related CKD from 2022 to 2040 across countries 317](#_Toc185966461)

**Appendix 1.1** The mortality number from 1990 to 2021 for SSBs-related CKD across SDI

| Year | Global | High SDI | High-middle SDI | Middle SDI | Low-middle SDI | Low SDI |
| --- | --- | --- | --- | --- | --- | --- |
| 1990 | 1483.26 (689.86, 2600.92) | 627.65 (291.53, 1071.7) | 342.5 (153.27, 603.19) | 351.72 (163.77, 610.36) | 111.38 (50.76, 198.39) | 47.75 (20.73, 84.97) |
| 1991 | 1540.91 (721.59, 2699.11) | 655.99 (305.37, 1113.79) | 352.38 (156.06, 619.27) | 365.53 (170.2, 636.81) | 116.64 (52.81, 208.54) | 48.11 (20.92, 84.82) |
| 1992 | 1607.26 (748.46, 2794.07) | 686.66 (321.53, 1164.19) | 363.33 (159.36, 641.47) | 383.68 (178.04, 664.6) | 122.72 (55.44, 217.57) | 48.59 (20.89, 84.83) |
| 1993 | 1679.16 (786.22, 2926.13) | 725.66 (342.4, 1234.04) | 371.19 (165.26, 659.68) | 402.19 (185.98, 700.76) | 128.98 (58.26, 230.42) | 48.89 (21.19, 85.92) |
| 1994 | 1750.74 (817.59, 3048.08) | 758.61 (356.98, 1290.41) | 379.77 (168.62, 675.56) | 425.19 (196.44, 739.8) | 135.66 (62.43, 239.99) | 49.24 (21.74, 86.12) |
| 1995 | 1850.73 (864.14, 3204.8) | 812.35 (379.98, 1382.11) | 391.43 (173.01, 692.12) | 450.43 (209.07, 774.03) | 144.34 (65.76, 253.58) | 49.84 (22.22, 86.94) |
| 1996 | 1950.55 (913.21, 3367.14) | 851.25 (396.09, 1456.11) | 406.02 (180.98, 717.76) | 485.82 (228.31, 835.93) | 154.47 (70.43, 273.45) | 50.57 (22.53, 87.23) |
| 1997 | 2062.56 (966.16, 3554.9) | 890.81 (414.48, 1511.88) | 428.06 (192.9, 751.58) | 526.09 (246.74, 897.75) | 164.36 (75.8, 290.79) | 50.72 (22.76, 88.22) |
| 1998 | 2235.99 (1052.42, 3844.11) | 954.08 (443.24, 1633.39) | 448.58 (205.61, 785.12) | 606.24 (280.92, 1022.52) | 172.95 (79.4, 307.27) | 51.56 (23.04, 90.65) |
| 1999 | 2420.57 (1141.55, 4149.23) | 1056.04 (489.27, 1798.95) | 468.31 (218.65, 814.35) | 661.27 (304.59, 1113.7) | 180.26 (83.17, 319.13) | 52.07 (23, 91.05) |
| 2000 | 2646.23 (1244.45, 4542.65) | 1185.95 (547.13, 2029.13) | 489.31 (227.49, 846.15) | 725.94 (335.6, 1216.27) | 189.49 (86.26, 336.46) | 52.77 (23.44, 92.84) |
| 2001 | 2807.91 (1322.65, 4812.99) | 1265.98 (587.12, 2155.02) | 510.2 (241.26, 886.36) | 773.64 (354.51, 1298.06) | 201.54 (92.57, 354.62) | 53.54 (23.64, 94.32) |
| 2002 | 2934.71 (1383.88, 5024.72) | 1318.6 (609.44, 2234.53) | 531.65 (247.89, 921.47) | 813.81 (373.64, 1364.15) | 212.76 (96.68, 371.01) | 54.79 (24.03, 97.51) |
| 2003 | 3056.26 (1444.26, 5204.13) | 1360.94 (632.47, 2301.5) | 546.74 (256.75, 939.05) | 862.39 (396.65, 1441.05) | 226.2 (103.64, 393.42) | 56.71 (25.02, 99.84) |
| 2004 | 3149.04 (1482.81, 5377.76) | 1384.36 (647.34, 2336.08) | 559.33 (259.28, 960.08) | 905.28 (416.03, 1516.16) | 238.28 (109.08, 414.68) | 58.39 (25.79, 103.16) |
| 2005 | 3280.16 (1548.68, 5563.16) | 1428.41 (673.37, 2391.1) | 579.21 (266.95, 990.16) | 958.88 (442.14, 1609.33) | 250.16 (114.69, 432.04) | 59.88 (26.6, 105.77) |
| 2006 | 3433.3 (1626.94, 5808.88) | 1482.82 (704.87, 2478.93) | 602.05 (275.76, 1028.88) | 1021.67 (474.79, 1706.27) | 261.15 (119.62, 452.87) | 61.77 (27.2, 109.53) |
| 2007 | 3621.44 (1715.86, 6106.05) | 1543.1 (733.99, 2569) | 632.42 (289.38, 1080.42) | 1101.52 (508.59, 1841.17) | 276.64 (127.25, 475.85) | 63.72 (28.08, 113.6) |
| 2008 | 3824.58 (1822.14, 6377.58) | 1605.05 (766.6, 2664.37) | 663.45 (306.17, 1134.17) | 1190.94 (550.1, 1989.95) | 295.06 (137.74, 504.03) | 65.87 (29.11, 116.93) |
| 2009 | 3998.04 (1899.27, 6726.73) | 1661.28 (786.07, 2769.12) | 693.78 (319.43, 1196.95) | 1254.29 (581.83, 2097.83) | 315.3 (147.35, 538.08) | 68.93 (30.38, 122.46) |
| 2010 | 4214.62 (2009.1, 7058.24) | 1756.74 (829.89, 2904) | 731.89 (339.74, 1267.63) | 1312.3 (607.35, 2190.51) | 336.79 (156.9, 571.5) | 72.27 (31.63, 130.96) |
| 2011 | 4483.88 (2150.19, 7469.02) | 1914.49 (899.17, 3158.88) | 760.47 (356.62, 1299.46) | 1369.19 (638.63, 2307.95) | 359.14 (168.72, 608.91) | 75.78 (33.16, 134.84) |
| 2012 | 4710.02 (2261.7, 7781.77) | 2026.19 (954.28, 3329.03) | 784.77 (369.98, 1330.36) | 1435.87 (674.54, 2406.81) | 378.51 (176.69, 643.02) | 79.71 (34.89, 141.02) |
| 2013 | 4977.79 (2399.26, 8192.74) | 2161.08 (1014.42, 3546.22) | 809.73 (383.18, 1366.31) | 1514.92 (707.28, 2521.84) | 402.45 (185.99, 678.68) | 84.51 (37.1, 148.78) |
| 2014 | 5205.84 (2500.04, 8569.82) | 2276.24 (1068.13, 3740.28) | 846.77 (404.39, 1418.97) | 1560.26 (724.48, 2595.76) | 428.25 (200.01, 721.01) | 89.11 (38.73, 156.17) |
| 2015 | 5492.85 (2657.34, 9073.03) | 2397.38 (1133.28, 3923.03) | 891.64 (420.35, 1513.29) | 1648.57 (769.33, 2731.77) | 456.49 (211.86, 772.9) | 93.31 (40.54, 162.13) |
| 2016 | 5762.99 (2774.72, 9507.6) | 2506.65 (1189.63, 4088.51) | 932.43 (448.12, 1556.89) | 1739.5 (816.33, 2882.65) | 481.51 (220.01, 815.39) | 97.19 (42.71, 168.11) |
| 2017 | 5940.76 (2839.86, 9820.74) | 2592.82 (1240.43, 4226.35) | 962.68 (464.66, 1632.8) | 1778.28 (828.98, 2954.51) | 499.52 (225.19, 850.75) | 101.52 (44.55, 175.66) |
| 2018 | 6105.72 (2941.53, 10092.78) | 2676.44 (1293.33, 4353.45) | 995.6 (483.28, 1683.23) | 1796.78 (847.3, 2968.82) | 524.74 (239.05, 886.45) | 106 (46.36, 184.68) |
| 2019 | 6366.48 (3083.49, 10522.87) | 2783.39 (1343.24, 4545.03) | 1038.45 (498.87, 1776.27) | 1875.05 (898.11, 3080.32) | 552.13 (250.99, 933.27) | 111.09 (48.63, 192.05) |
| 2020 | 6600.82 (3153.48, 10950.34) | 2845.99 (1364.21, 4727.2) | 1061.27 (506.81, 1807.2) | 1996.95 (937.28, 3272.78) | 574 (259.21, 948.27) | 116.09 (51.88, 201.62) |
| 2021 | 6782.03 (3266.15, 11232.9) | 2929.07 (1428.19, 4867.13) | 1084.17 (513.35, 1825.56) | 2063.07 (972.53, 3420.81) | 580.61 (258.71, 976.68) | 118.55 (51.74, 204.9) |

**Appendix 1.2** The all-age mortality rate from 1990 to 2021 for SSBs-related CKD across SDI

| Year | Global | High SDI | High-middle SDI | Middle SDI | Low-middle SDI | Low SDI |
| --- | --- | --- | --- | --- | --- | --- |
| 1990 | 0.028 (0.013, 0.049) | 0.071 (0.033, 0.122) | 0.032 (0.014, 0.057) | 0.02 (0.01, 0.035) | 0.01 (0.004, 0.017) | 0.01 (0.004, 0.017) |
| 1991 | 0.028 (0.013, 0.05) | 0.074 (0.034, 0.126) | 0.033 (0.015, 0.058) | 0.021 (0.01, 0.036) | 0.01 (0.004, 0.018) | 0.009 (0.004, 0.017) |
| 1992 | 0.029 (0.014, 0.051) | 0.077 (0.036, 0.13) | 0.033 (0.015, 0.059) | 0.022 (0.01, 0.037) | 0.01 (0.005, 0.018) | 0.009 (0.004, 0.016) |
| 1993 | 0.03 (0.014, 0.052) | 0.081 (0.038, 0.137) | 0.034 (0.015, 0.06) | 0.022 (0.01, 0.039) | 0.011 (0.005, 0.019) | 0.009 (0.004, 0.016) |
| 1994 | 0.031 (0.014, 0.054) | 0.084 (0.039, 0.142) | 0.034 (0.015, 0.061) | 0.023 (0.011, 0.04) | 0.011 (0.005, 0.019) | 0.009 (0.004, 0.016) |
| 1995 | 0.032 (0.015, 0.056) | 0.089 (0.042, 0.151) | 0.035 (0.016, 0.062) | 0.024 (0.011, 0.042) | 0.011 (0.005, 0.02) | 0.009 (0.004, 0.015) |
| 1996 | 0.034 (0.016, 0.058) | 0.093 (0.043, 0.159) | 0.036 (0.016, 0.064) | 0.026 (0.012, 0.045) | 0.012 (0.005, 0.021) | 0.009 (0.004, 0.015) |
| 1997 | 0.035 (0.016, 0.061) | 0.096 (0.045, 0.164) | 0.038 (0.017, 0.067) | 0.028 (0.013, 0.047) | 0.012 (0.006, 0.022) | 0.008 (0.004, 0.015) |
| 1998 | 0.038 (0.018, 0.065) | 0.102 (0.048, 0.175) | 0.039 (0.018, 0.069) | 0.032 (0.015, 0.053) | 0.013 (0.006, 0.023) | 0.008 (0.004, 0.015) |
| 1999 | 0.04 (0.019, 0.069) | 0.113 (0.052, 0.192) | 0.041 (0.019, 0.071) | 0.034 (0.016, 0.058) | 0.013 (0.006, 0.023) | 0.008 (0.004, 0.014) |
| 2000 | 0.043 (0.02, 0.074) | 0.126 (0.058, 0.215) | 0.042 (0.02, 0.073) | 0.037 (0.017, 0.062) | 0.014 (0.006, 0.024) | 0.008 (0.004, 0.014) |
| 2001 | 0.045 (0.021, 0.078) | 0.133 (0.062, 0.227) | 0.044 (0.021, 0.076) | 0.039 (0.018, 0.066) | 0.014 (0.007, 0.025) | 0.008 (0.004, 0.014) |
| 2002 | 0.047 (0.022, 0.08) | 0.138 (0.064, 0.233) | 0.046 (0.021, 0.079) | 0.041 (0.019, 0.068) | 0.015 (0.007, 0.026) | 0.008 (0.004, 0.014) |
| 2003 | 0.048 (0.023, 0.082) | 0.141 (0.066, 0.239) | 0.047 (0.022, 0.08) | 0.043 (0.02, 0.071) | 0.015 (0.007, 0.027) | 0.008 (0.004, 0.014) |
| 2004 | 0.049 (0.023, 0.084) | 0.142 (0.067, 0.24) | 0.047 (0.022, 0.081) | 0.044 (0.02, 0.074) | 0.016 (0.007, 0.028) | 0.008 (0.004, 0.014) |
| 2005 | 0.05 (0.024, 0.086) | 0.146 (0.069, 0.244) | 0.049 (0.022, 0.083) | 0.046 (0.021, 0.078) | 0.017 (0.008, 0.029) | 0.008 (0.004, 0.014) |
| 2006 | 0.052 (0.025, 0.088) | 0.15 (0.071, 0.251) | 0.05 (0.023, 0.086) | 0.049 (0.023, 0.082) | 0.017 (0.008, 0.029) | 0.008 (0.004, 0.014) |
| 2007 | 0.054 (0.026, 0.091) | 0.155 (0.074, 0.257) | 0.052 (0.024, 0.09) | 0.052 (0.024, 0.087) | 0.018 (0.008, 0.03) | 0.008 (0.004, 0.014) |
| 2008 | 0.057 (0.027, 0.094) | 0.159 (0.076, 0.264) | 0.055 (0.025, 0.093) | 0.056 (0.026, 0.093) | 0.019 (0.009, 0.032) | 0.008 (0.004, 0.014) |
| 2009 | 0.058 (0.028, 0.098) | 0.163 (0.077, 0.272) | 0.057 (0.026, 0.098) | 0.058 (0.027, 0.097) | 0.02 (0.009, 0.033) | 0.008 (0.004, 0.015) |
| 2010 | 0.061 (0.029, 0.102) | 0.171 (0.081, 0.283) | 0.06 (0.028, 0.103) | 0.06 (0.028, 0.1) | 0.02 (0.01, 0.035) | 0.008 (0.004, 0.015) |
| 2011 | 0.064 (0.031, 0.106) | 0.185 (0.087, 0.306) | 0.062 (0.029, 0.105) | 0.062 (0.029, 0.104) | 0.022 (0.01, 0.036) | 0.009 (0.004, 0.015) |
| 2012 | 0.066 (0.032, 0.109) | 0.195 (0.092, 0.32) | 0.063 (0.03, 0.107) | 0.064 (0.03, 0.107) | 0.022 (0.01, 0.038) | 0.009 (0.004, 0.016) |
| 2013 | 0.069 (0.033, 0.114) | 0.206 (0.097, 0.338) | 0.065 (0.031, 0.109) | 0.067 (0.031, 0.111) | 0.023 (0.011, 0.039) | 0.009 (0.004, 0.016) |
| 2014 | 0.071 (0.034, 0.117) | 0.215 (0.101, 0.354) | 0.067 (0.032, 0.113) | 0.068 (0.032, 0.113) | 0.024 (0.011, 0.041) | 0.009 (0.004, 0.017) |
| 2015 | 0.074 (0.036, 0.123) | 0.225 (0.107, 0.369) | 0.07 (0.033, 0.119) | 0.071 (0.033, 0.118) | 0.026 (0.012, 0.044) | 0.01 (0.004, 0.017) |
| 2016 | 0.077 (0.037, 0.127) | 0.234 (0.111, 0.382) | 0.073 (0.035, 0.122) | 0.074 (0.035, 0.123) | 0.027 (0.012, 0.045) | 0.01 (0.004, 0.017) |
| 2017 | 0.078 (0.037, 0.13) | 0.241 (0.115, 0.392) | 0.075 (0.036, 0.127) | 0.075 (0.035, 0.125) | 0.027 (0.012, 0.047) | 0.01 (0.004, 0.017) |
| 2018 | 0.08 (0.038, 0.132) | 0.247 (0.119, 0.402) | 0.077 (0.037, 0.13) | 0.075 (0.035, 0.124) | 0.028 (0.013, 0.048) | 0.01 (0.004, 0.018) |
| 2019 | 0.082 (0.04, 0.136) | 0.256 (0.124, 0.418) | 0.08 (0.038, 0.137) | 0.078 (0.037, 0.128) | 0.029 (0.013, 0.05) | 0.01 (0.005, 0.018) |
| 2020 | 0.084 (0.04, 0.14) | 0.261 (0.125, 0.433) | 0.082 (0.039, 0.139) | 0.082 (0.039, 0.135) | 0.03 (0.014, 0.05) | 0.011 (0.005, 0.018) |
| 2021 | 0.086 (0.041, 0.142) | 0.268 (0.131, 0.445) | 0.083 (0.039, 0.14) | 0.084 (0.04, 0.14) | 0.03 (0.013, 0.051) | 0.011 (0.005, 0.018) |

**Appendix 1.3** The ASMRs from 1990 to 2021 for SSBs-related CKD across SDI

| Year | Global | High SDI | High-middle SDI | Middle SDI | Low-middle SDI | Low SDI |
| --- | --- | --- | --- | --- | --- | --- |
| 1990 | 0.045 (0.021, 0.078) | 0.058 (0.027, 0.099) | 0.043 (0.019, 0.077) | 0.041 (0.02, 0.069) | 0.021 (0.01, 0.037) | 0.023 (0.011, 0.041) |
| 1991 | 0.045 (0.021, 0.079) | 0.059 (0.028, 0.101) | 0.043 (0.019, 0.077) | 0.041 (0.02, 0.069) | 0.021 (0.01, 0.037) | 0.023 (0.01, 0.04) |
| 1992 | 0.046 (0.022, 0.08) | 0.061 (0.029, 0.103) | 0.043 (0.019, 0.077) | 0.041 (0.02, 0.069) | 0.022 (0.01, 0.037) | 0.023 (0.01, 0.039) |
| 1993 | 0.047 (0.022, 0.081) | 0.063 (0.03, 0.106) | 0.043 (0.019, 0.076) | 0.042 (0.02, 0.071) | 0.022 (0.01, 0.039) | 0.023 (0.01, 0.039) |
| 1994 | 0.048 (0.023, 0.082) | 0.064 (0.03, 0.109) | 0.044 (0.02, 0.076) | 0.043 (0.02, 0.073) | 0.023 (0.01, 0.039) | 0.022 (0.01, 0.039) |
| 1995 | 0.049 (0.023, 0.084) | 0.067 (0.031, 0.114) | 0.044 (0.02, 0.077) | 0.044 (0.021, 0.075) | 0.023 (0.011, 0.041) | 0.022 (0.01, 0.039) |
| 1996 | 0.05 (0.024, 0.086) | 0.068 (0.032, 0.117) | 0.045 (0.02, 0.077) | 0.047 (0.022, 0.078) | 0.024 (0.011, 0.043) | 0.022 (0.01, 0.039) |
| 1997 | 0.052 (0.025, 0.088) | 0.07 (0.032, 0.119) | 0.046 (0.021, 0.079) | 0.049 (0.023, 0.082) | 0.025 (0.012, 0.044) | 0.022 (0.01, 0.038) |
| 1998 | 0.055 (0.026, 0.092) | 0.073 (0.034, 0.125) | 0.047 (0.022, 0.081) | 0.054 (0.026, 0.089) | 0.026 (0.012, 0.046) | 0.022 (0.01, 0.038) |
| 1999 | 0.057 (0.027, 0.097) | 0.079 (0.037, 0.135) | 0.048 (0.023, 0.083) | 0.057 (0.027, 0.094) | 0.026 (0.012, 0.046) | 0.021 (0.01, 0.037) |
| 2000 | 0.061 (0.029, 0.104) | 0.087 (0.04, 0.149) | 0.049 (0.023, 0.086) | 0.06 (0.029, 0.099) | 0.027 (0.012, 0.047) | 0.021 (0.009, 0.038) |
| 2001 | 0.063 (0.03, 0.107) | 0.091 (0.042, 0.155) | 0.05 (0.024, 0.087) | 0.062 (0.03, 0.103) | 0.028 (0.013, 0.048) | 0.021 (0.009, 0.037) |
| 2002 | 0.064 (0.031, 0.109) | 0.092 (0.043, 0.157) | 0.051 (0.024, 0.088) | 0.064 (0.03, 0.105) | 0.029 (0.013, 0.05) | 0.021 (0.009, 0.037) |
| 2003 | 0.065 (0.031, 0.11) | 0.093 (0.043, 0.158) | 0.052 (0.024, 0.088) | 0.065 (0.031, 0.108) | 0.03 (0.014, 0.051) | 0.021 (0.009, 0.037) |
| 2004 | 0.065 (0.031, 0.11) | 0.093 (0.043, 0.157) | 0.051 (0.024, 0.088) | 0.066 (0.032, 0.11) | 0.03 (0.014, 0.053) | 0.021 (0.009, 0.037) |
| 2005 | 0.066 (0.031, 0.111) | 0.093 (0.044, 0.157) | 0.052 (0.024, 0.088) | 0.068 (0.032, 0.112) | 0.031 (0.014, 0.053) | 0.021 (0.009, 0.037) |
| 2006 | 0.067 (0.032, 0.113) | 0.095 (0.045, 0.158) | 0.052 (0.024, 0.089) | 0.07 (0.033, 0.115) | 0.031 (0.014, 0.054) | 0.021 (0.009, 0.037) |
| 2007 | 0.068 (0.032, 0.115) | 0.096 (0.045, 0.159) | 0.053 (0.024, 0.09) | 0.072 (0.035, 0.119) | 0.032 (0.015, 0.055) | 0.021 (0.01, 0.037) |
| 2008 | 0.07 (0.033, 0.117) | 0.097 (0.046, 0.16) | 0.054 (0.025, 0.091) | 0.075 (0.036, 0.124) | 0.033 (0.016, 0.057) | 0.021 (0.01, 0.038) |
| 2009 | 0.071 (0.033, 0.119) | 0.098 (0.046, 0.162) | 0.054 (0.025, 0.093) | 0.077 (0.037, 0.126) | 0.034 (0.016, 0.058) | 0.022 (0.01, 0.038) |
| 2010 | 0.072 (0.034, 0.121) | 0.101 (0.048, 0.165) | 0.055 (0.026, 0.096) | 0.078 (0.037, 0.127) | 0.036 (0.017, 0.06) | 0.022 (0.01, 0.039) |
| 2011 | 0.074 (0.035, 0.123) | 0.107 (0.051, 0.176) | 0.055 (0.026, 0.094) | 0.078 (0.038, 0.129) | 0.037 (0.017, 0.062) | 0.022 (0.01, 0.04) |
| 2012 | 0.075 (0.036, 0.125) | 0.111 (0.052, 0.181) | 0.055 (0.026, 0.093) | 0.079 (0.038, 0.13) | 0.037 (0.018, 0.063) | 0.023 (0.01, 0.04) |
| 2013 | 0.077 (0.037, 0.127) | 0.116 (0.054, 0.19) | 0.055 (0.026, 0.092) | 0.08 (0.039, 0.131) | 0.039 (0.018, 0.065) | 0.024 (0.011, 0.041) |
| 2014 | 0.078 (0.037, 0.128) | 0.119 (0.056, 0.196) | 0.056 (0.027, 0.093) | 0.08 (0.038, 0.131) | 0.04 (0.019, 0.067) | 0.024 (0.011, 0.042) |
| 2015 | 0.08 (0.039, 0.131) | 0.123 (0.058, 0.2) | 0.057 (0.027, 0.096) | 0.081 (0.039, 0.134) | 0.042 (0.02, 0.069) | 0.025 (0.011, 0.043) |
| 2016 | 0.081 (0.039, 0.133) | 0.126 (0.059, 0.205) | 0.057 (0.028, 0.096) | 0.082 (0.04, 0.135) | 0.042 (0.02, 0.07) | 0.025 (0.011, 0.044) |
| 2017 | 0.081 (0.039, 0.134) | 0.127 (0.061, 0.205) | 0.057 (0.028, 0.097) | 0.081 (0.039, 0.134) | 0.042 (0.019, 0.071) | 0.026 (0.012, 0.044) |
| 2018 | 0.081 (0.039, 0.133) | 0.128 (0.062, 0.208) | 0.058 (0.028, 0.098) | 0.079 (0.038, 0.13) | 0.043 (0.02, 0.071) | 0.026 (0.012, 0.045) |
| 2019 | 0.082 (0.039, 0.134) | 0.13 (0.063, 0.21) | 0.058 (0.028, 0.1) | 0.08 (0.039, 0.129) | 0.044 (0.02, 0.072) | 0.026 (0.012, 0.045) |
| 2020 | 0.082 (0.039, 0.136) | 0.13 (0.062, 0.212) | 0.058 (0.028, 0.099) | 0.082 (0.039, 0.134) | 0.044 (0.02, 0.072) | 0.026 (0.012, 0.045) |
| 2021 | 0.082 (0.04, 0.136) | 0.131 (0.064, 0.214) | 0.057 (0.027, 0.097) | 0.082 (0.039, 0.135) | 0.043 (0.02, 0.072) | 0.026 (0.011, 0.045) |

**Appendix 1.4** The DALYs from 1990 to 2021 for SSBs-related CKD across SDI

| Year | Global | High SDI | High-middle SDI | Middle SDI | Low-middle SDI | Low SDI |
| --- | --- | --- | --- | --- | --- | --- |
| 1990 | 47997.68 (21586.6, 86844.03) | 19323.68 (8873.65, 33941.49) | 10210.33 (4551.54, 18969.47) | 12676.73 (5642.65, 22809.88) | 4057.68 (1770.43, 7614.21) | 1652.03 (713.95, 3072.5) |
| 1991 | 49508.12 (22276.45, 89527.98) | 20058.18 (9206.51, 35168.99) | 10384.28 (4636.56, 19244.01) | 13103.81 (5766.61, 23518.03) | 4221.4 (1853.49, 7877) | 1663.08 (720.22, 3080.91) |
| 1992 | 51257.64 (23032.26, 92628.57) | 20858.31 (9541.81, 36500.02) | 10575.96 (4704.96, 19732.73) | 13663.08 (5992.65, 24227.09) | 4407.14 (1919.65, 8171.02) | 1675.97 (730.69, 3094.87) |
| 1993 | 53147.24 (23996.32, 95867.61) | 21853.06 (10001.45, 37866.68) | 10688.88 (4775.88, 19959.09) | 14249.23 (6265.66, 25496.27) | 4598.59 (1989.54, 8531.59) | 1680.72 (727.82, 3063.37) |
| 1994 | 55093.35 (24882.77, 99234.76) | 22767.37 (10460.55, 39327.23) | 10804.33 (4824.9, 20212.89) | 14957.73 (6613.83, 26728.48) | 4803.04 (2099.89, 8905.54) | 1683.93 (732.83, 3041.56) |
| 1995 | 57647.47 (26051.74, 102889.7) | 24110.87 (11138.16, 41651.25) | 10982.67 (4939.51, 20407.4) | 15721.41 (6964.57, 27893.17) | 5053.03 (2191.39, 9299.14) | 1700.54 (743.67, 3084.39) |
| 1996 | 60362.17 (27301.65, 107488.22) | 25213.05 (11666.31, 43698.91) | 11231.46 (5078.08, 20691.1) | 16743.85 (7486.92, 29499.63) | 5367.38 (2325.46, 9827.95) | 1725.29 (746.31, 3099.15) |
| 1997 | 63572.9 (28709, 112882.71) | 26464.08 (12240.61, 45872.91) | 11632.58 (5281.98, 21414.42) | 17982.97 (8079.79, 31374.13) | 5683.5 (2484.09, 10391.48) | 1725.63 (752.96, 3138.58) |
| 1998 | 68459.28 (31135.9, 121301.89) | 28213.02 (12978.84, 48724.35) | 12027.03 (5485.59, 22032.05) | 20419.94 (9169.18, 35347.32) | 5962.64 (2609.69, 11025.27) | 1751.73 (766.23, 3195.11) |
| 1999 | 73563.09 (33445.72, 130306.3) | 30920.16 (14164.64, 53185.31) | 12448.58 (5696.78, 22739.19) | 22116.81 (9939.03, 38168.21) | 6220.94 (2718.6, 11481.97) | 1771.4 (774.24, 3226.28) |
| 2000 | 79020.15 (35999.05, 139767.86) | 33739.23 (15520.94, 58499.11) | 12852.71 (5928.38, 23257.46) | 24032.69 (10746.93, 41550.34) | 6512.71 (2842.53, 12078.24) | 1794.26 (775.56, 3270.36) |
| 2001 | 83282.87 (37855.73, 146973.63) | 35661.09 (16307.35, 61849.38) | 13346.8 (6158.37, 23971.68) | 25471.5 (11427.51, 44077.72) | 6887.22 (3017.04, 12695.19) | 1821.19 (788.31, 3311.64) |
| 2002 | 86909.63 (39586.62, 153044.41) | 37043.77 (17107.03, 64493.82) | 13893.04 (6330.13, 24804.54) | 26769.62 (12017.36, 46162.96) | 7245.42 (3157.15, 13304.2) | 1859.46 (795.91, 3366.32) |
| 2003 | 90668.96 (41208.25, 159288.44) | 38314.37 (17821.67, 66641.64) | 14354.02 (6551.44, 25348.82) | 28303.54 (12763.71, 48734.36) | 7668.81 (3359.63, 14112.3) | 1925.16 (826.04, 3476) |
| 2004 | 93774.91 (42408.35, 164793.68) | 39199.03 (18296.25, 67962.74) | 14787.82 (6675.38, 26027.08) | 29632.88 (13264.08, 51292.83) | 8063.46 (3534.57, 14730.64) | 1985.46 (841.17, 3626.05) |
| 2005 | 97782.86 (44343.42, 171055.31) | 40411.38 (19042.34, 70206.84) | 15419.4 (6946.86, 27091.09) | 31308.98 (13976.41, 53976.39) | 8491.34 (3751.6, 15470.93) | 2039.61 (860.42, 3715.16) |
| 2006 | 102260.09 (46423.62, 178133.24) | 41786.57 (19715.42, 72624.42) | 16085.6 (7271.44, 28184.2) | 33248.24 (14918.83, 57342.25) | 8914.73 (3932.96, 16106.55) | 2106.51 (885.09, 3885.69) |
| 2007 | 107505.33 (48960.77, 187194.54) | 43072.49 (20225.64, 74405.33) | 16982.91 (7604.78, 29602.18) | 35680.12 (15938.65, 61212.55) | 9464.72 (4110.16, 17229.78) | 2180.69 (928.78, 4044.47) |
| 2008 | 113167.8 (51623.14, 195338.17) | 44468.78 (21037.18, 76576.73) | 17801.43 (8056.49, 30951.67) | 38391.86 (17361.03, 65662.47) | 10116.57 (4471.41, 18243.07) | 2259.31 (964.75, 4281.2) |
| 2009 | 117931.36 (53715.73, 204797.23) | 45630.92 (21727.98, 78584.72) | 18644.93 (8495.28, 32408.6) | 40315.55 (17970.97, 69077.62) | 10835.01 (4765.58, 19364.97) | 2368.5 (1011.29, 4451.93) |
| 2010 | 123319.32 (56307.23, 212528.43) | 47564.97 (22714.86, 81267.54) | 19558.2 (8888.19, 33588.9) | 41995.98 (18713.83, 71320.46) | 11573.48 (5089.03, 20691.42) | 2484.81 (1073.19, 4665.3) |
| 2011 | 130219.47 (59513.61, 224372.27) | 51213.77 (24449.31, 87474.42) | 20253.07 (9237.16, 35082.2) | 43675.59 (19544.74, 74244.64) | 12323.15 (5471.69, 21994.27) | 2607.14 (1112.81, 4894.94) |
| 2012 | 136375.48 (62657.14, 234968.33) | 53828.23 (25724.48, 91275.95) | 20905.67 (9532.14, 36116.24) | 45760.16 (20527.86, 77976.01) | 12983.5 (5751.9, 22967.66) | 2746.04 (1180.92, 5202.93) |
| 2013 | 143620.5 (66148.17, 247510.84) | 57179.17 (27173.77, 96217.22) | 21538.46 (9854.6, 37360.02) | 48111.08 (21696.96, 81788.2) | 13732.19 (6015.59, 24279.96) | 2903.56 (1230.62, 5480.28) |
| 2014 | 149572.97 (69360.33, 256213.76) | 60021.86 (28376.75, 100154.21) | 22311.33 (10173.82, 38442.15) | 49500.74 (22300.02, 84251.32) | 14520.16 (6342.43, 25691.7) | 3059.42 (1285.67, 5796.38) |
| 2015 | 157144.92 (73057.95, 268747.34) | 62881.54 (29658.46, 104372.78) | 23269.24 (10614.73, 40706.96) | 52141.23 (23472.66, 88445.12) | 15481.48 (6748.2, 27392.66) | 3205.67 (1345.04, 6103.2) |
| 2016 | 163892.1 (76071.21, 278908.22) | 65049.89 (30868.15, 108177.77) | 24148.4 (11117.61, 42322.18) | 54890.14 (24709.57, 93101.37) | 16288.92 (7115.09, 28850.86) | 3343.61 (1394.79, 6373.01) |
| 2017 | 167543.8 (77423.88, 284056.21) | 66320.1 (31401.01, 109732.57) | 24791.08 (11383.12, 43348.74) | 55948.12 (25059.42, 94238.61) | 16817.99 (7298.95, 29932.01) | 3489.79 (1462.82, 6635.1) |
| 2018 | 170627.59 (79121.19, 287315.01) | 67349.97 (31832.48, 111814.39) | 25554.54 (11783.82, 44822.84) | 56394.1 (25594.28, 95583.19) | 17507.78 (7687.59, 31009.05) | 3639.78 (1511.41, 6904.39) |
| 2019 | 176471.74 (81536.37, 295827.34) | 69167.58 (32634.52, 114326.97) | 26482.44 (12325.61, 46119.3) | 58539.46 (26813.55, 97557.24) | 18287.17 (8067.68, 32034.58) | 3808.76 (1600.41, 7210.5) |
| 2020 | 183373.07 (84795.09, 309445.26) | 70214.29 (33168.58, 117196.49) | 26981.1 (12527.95, 47450.89) | 62694.64 (28713.75, 106216.46) | 19276.4 (8441.93, 33831.96) | 4018.71 (1687.35, 7515.85) |
| 2021 | 188563.04 (86483.86, 316322.19) | 72057.63 (34000.73, 120187.23) | 27601.35 (12922.43, 47955.67) | 64858.14 (29249.36, 111466.14) | 19706.18 (8439.09, 34734.65) | 4151.28 (1720.29, 7723.18) |

**Appendix 1.5** The age-standardized DALYs rate from 1990 to 2021 for SSBs-related CKD across SDI

| Year | Global | High SDI | High-middle SDI | Middle SDI | Low-middle SDI | Low SDI |
| --- | --- | --- | --- | --- | --- | --- |
| 1990 | 1.23 (0.57, 2.18) | 1.81 (0.84, 3.19) | 1.09 (0.5, 1.99) | 1.13 (0.52, 1.97) | 0.61 (0.28, 1.11) | 0.66 (0.29, 1.18) |
| 1991 | 1.24 (0.57, 2.19) | 1.85 (0.85, 3.23) | 1.08 (0.5, 1.98) | 1.13 (0.52, 1.98) | 0.62 (0.28, 1.12) | 0.65 (0.28, 1.14) |
| 1992 | 1.25 (0.58, 2.22) | 1.89 (0.87, 3.3) | 1.08 (0.49, 1.99) | 1.15 (0.52, 1.98) | 0.63 (0.29, 1.14) | 0.64 (0.28, 1.12) |
| 1993 | 1.27 (0.59, 2.24) | 1.94 (0.89, 3.4) | 1.07 (0.49, 1.97) | 1.16 (0.53, 2.03) | 0.64 (0.29, 1.16) | 0.63 (0.28, 1.1) |
| 1994 | 1.28 (0.59, 2.27) | 1.99 (0.91, 3.46) | 1.06 (0.48, 1.96) | 1.19 (0.54, 2.07) | 0.66 (0.3, 1.18) | 0.62 (0.28, 1.09) |
| 1995 | 1.32 (0.61, 2.3) | 2.06 (0.95, 3.61) | 1.06 (0.48, 1.95) | 1.22 (0.56, 2.13) | 0.68 (0.31, 1.21) | 0.61 (0.28, 1.07) |
| 1996 | 1.35 (0.62, 2.35) | 2.12 (0.97, 3.71) | 1.06 (0.48, 1.94) | 1.27 (0.59, 2.22) | 0.7 (0.31, 1.26) | 0.61 (0.27, 1.07) |
| 1997 | 1.38 (0.64, 2.41) | 2.19 (1.01, 3.81) | 1.08 (0.49, 1.96) | 1.33 (0.62, 2.3) | 0.73 (0.32, 1.3) | 0.6 (0.27, 1.04) |
| 1998 | 1.45 (0.67, 2.54) | 2.29 (1.05, 3.97) | 1.09 (0.5, 1.98) | 1.46 (0.68, 2.49) | 0.74 (0.34, 1.33) | 0.59 (0.26, 1.04) |
| 1999 | 1.52 (0.71, 2.66) | 2.47 (1.14, 4.29) | 1.11 (0.52, 1.99) | 1.54 (0.72, 2.61) | 0.76 (0.34, 1.35) | 0.58 (0.26, 1.02) |
| 2000 | 1.6 (0.74, 2.8) | 2.65 (1.21, 4.63) | 1.12 (0.53, 2) | 1.63 (0.76, 2.76) | 0.77 (0.35, 1.39) | 0.58 (0.25, 1.01) |
| 2001 | 1.65 (0.76, 2.88) | 2.76 (1.26, 4.84) | 1.14 (0.54, 2.03) | 1.69 (0.77, 2.84) | 0.79 (0.36, 1.42) | 0.57 (0.25, 1) |
| 2002 | 1.68 (0.78, 2.92) | 2.82 (1.3, 4.96) | 1.17 (0.55, 2.07) | 1.72 (0.79, 2.9) | 0.81 (0.37, 1.45) | 0.57 (0.25, 0.99) |
| 2003 | 1.7 (0.79, 2.96) | 2.87 (1.33, 5.04) | 1.18 (0.55, 2.06) | 1.77 (0.82, 2.98) | 0.84 (0.38, 1.49) | 0.57 (0.25, 1) |
| 2004 | 1.72 (0.79, 2.98) | 2.89 (1.34, 5.09) | 1.19 (0.55, 2.07) | 1.8 (0.83, 3.01) | 0.86 (0.39, 1.52) | 0.58 (0.25, 1.02) |
| 2005 | 1.74 (0.8, 3.02) | 2.93 (1.36, 5.16) | 1.21 (0.56, 2.11) | 1.85 (0.86, 3.1) | 0.88 (0.4, 1.54) | 0.58 (0.25, 1.01) |
| 2006 | 1.77 (0.82, 3.06) | 2.98 (1.39, 5.22) | 1.23 (0.57, 2.14) | 1.9 (0.89, 3.21) | 0.89 (0.4, 1.57) | 0.58 (0.25, 1.02) |
| 2007 | 1.82 (0.84, 3.13) | 3.01 (1.4, 5.29) | 1.27 (0.58, 2.19) | 1.98 (0.93, 3.34) | 0.92 (0.41, 1.62) | 0.58 (0.26, 1.04) |
| 2008 | 1.86 (0.85, 3.2) | 3.05 (1.42, 5.32) | 1.3 (0.59, 2.23) | 2.06 (0.96, 3.47) | 0.96 (0.43, 1.67) | 0.58 (0.26, 1.06) |
| 2009 | 1.89 (0.86, 3.25) | 3.07 (1.44, 5.36) | 1.32 (0.61, 2.3) | 2.1 (0.96, 3.57) | 0.99 (0.45, 1.73) | 0.59 (0.26, 1.07) |
| 2010 | 1.92 (0.88, 3.3) | 3.14 (1.47, 5.43) | 1.35 (0.62, 2.33) | 2.12 (0.98, 3.6) | 1.03 (0.46, 1.79) | 0.6 (0.27, 1.09) |
| 2011 | 1.97 (0.91, 3.4) | 3.33 (1.55, 5.72) | 1.36 (0.63, 2.37) | 2.14 (0.98, 3.64) | 1.06 (0.49, 1.85) | 0.61 (0.27, 1.11) |
| 2012 | 2.01 (0.93, 3.44) | 3.44 (1.61, 5.88) | 1.37 (0.63, 2.38) | 2.18 (0.99, 3.67) | 1.09 (0.5, 1.9) | 0.63 (0.28, 1.13) |
| 2013 | 2.06 (0.95, 3.53) | 3.6 (1.69, 6.11) | 1.38 (0.64, 2.4) | 2.22 (1.01, 3.73) | 1.12 (0.51, 1.94) | 0.64 (0.28, 1.16) |
| 2014 | 2.09 (0.97, 3.56) | 3.72 (1.74, 6.24) | 1.39 (0.64, 2.41) | 2.21 (1.01, 3.73) | 1.15 (0.52, 2) | 0.66 (0.29, 1.19) |
| 2015 | 2.14 (0.99, 3.64) | 3.84 (1.8, 6.43) | 1.42 (0.65, 2.48) | 2.26 (1.03, 3.8) | 1.19 (0.53, 2.08) | 0.67 (0.29, 1.21) |
| 2016 | 2.17 (1.01, 3.7) | 3.91 (1.83, 6.54) | 1.43 (0.66, 2.52) | 2.31 (1.05, 3.88) | 1.22 (0.54, 2.13) | 0.68 (0.3, 1.22) |
| 2017 | 2.16 (1, 3.67) | 3.92 (1.84, 6.57) | 1.44 (0.66, 2.51) | 2.28 (1.03, 3.83) | 1.22 (0.54, 2.14) | 0.69 (0.3, 1.23) |
| 2018 | 2.15 (1, 3.62) | 3.92 (1.83, 6.58) | 1.45 (0.66, 2.56) | 2.24 (1.02, 3.78) | 1.23 (0.55, 2.16) | 0.69 (0.3, 1.24) |
| 2019 | 2.17 (1, 3.64) | 3.96 (1.85, 6.67) | 1.47 (0.68, 2.56) | 2.26 (1.04, 3.74) | 1.25 (0.56, 2.19) | 0.7 (0.31, 1.25) |
| 2020 | 2.2 (1.02, 3.72) | 3.96 (1.86, 6.65) | 1.47 (0.67, 2.59) | 2.36 (1.08, 3.97) | 1.28 (0.57, 2.22) | 0.71 (0.31, 1.27) |
| 2021 | 2.22 (1.02, 3.74) | 4.01 (1.86, 6.73) | 1.47 (0.68, 2.6) | 2.39 (1.08, 4.09) | 1.28 (0.56, 2.22) | 0.72 (0.31, 1.26) |

**Appendix 1.6** The net drift of mortality from 1990 to 2021 for SSBs-related CKD across SDI

| Year | Global | High SDI | High-middle SDI | Middle SDI | Low-middle SDI | Low SDI |
| --- | --- | --- | --- | --- | --- | --- |
| 1990-2021 | 2.65 (2.42, 2.89) | 3.83 (3.4, 4.27) | 1.56 (0.95, 2.18) | 2.68 (2.29, 3.07) | 2.84 (2.12, 3.55) | 0.54 (-0.72, 1.81) |

**Appendix 2** Trends in the mortality of different types of CKD attributable to excessive consumption of SSBs by SDI in 1990 and 2021

|  | Global | | High SDI | | High-middle SDI | | Middle SDI | | Low-middle SDI | | Low SDI | |
| --- | --- | --- | --- | --- | --- | --- | --- | --- | --- | --- | --- | --- |
|  | 1990 | 2021 | 1990 | 2021 | 1990 | 2021 | 1990 | 2021 | 1990 | 2021 | 1990 | 2021 |
| **Chronic kidney disease due to hypertension** | | | | | | | | | | | | |
| **Deaths** | | | | | | | | | | | | |
| Number | 129.57(19.7, 356.11) | 764.22(167.30, 1808.45) | 55.9(7.65, 155.46) | 370.78(77.85, 852.91) | 33.63(4.7, 91.27) | 127.62(31.53, 297.77) | 29.97(5.41, 79.51) | 201.58(40.85, 513.75) | 5.85(0.54, 19.43) | 52.71(8.3, 141.43) | 4(0.66, 10.48) | 10.77(1.98, 29.07) |
| Percentage of global,% | 100 | 100 | 43.14 | 48.52 | 25.96 | 16.70 | 23.13 | 26.38 | 4.51 | 6.90 | 3.09 | 1.41 |
| Percentage change of rate 1990-2021,% | 489.82(387.56, 849.69) | | 563.28(387.77, 1192.23) | | 279.43(199.39, 620.71) | | 572.66(457.5, 806.48) | | 800.85(567.42, 1642.61) | | 169.57(89.65, 295.78) | |
| **All-age mortality rate** | | | | | | | | | | | | |
| Rate per 100,000 | 0.002(0, 0.007) | 0.01(0.002, 0.023) | 0.006(0.001, 0.018) | 0.034(0.007, 0.078) | 0.003(0, 0.009) | 0.01(0.002, 0.023) | 0.002(0, 0.005) | 0.008(0.002, 0.021) | 0.001(0, 0.002) | 0.003(0, 0.007) | 0.001(0, 0.002) | 0.001(0, 0.003) |
| Percentage change of rate 1990-2021,% | 298.65(229.53, 541.88) | | 433.23(292.13, 938.86) | | 209.46(144.18, 487.79) | | 373.32(292.29, 537.84) | | 444.61(303.49, 953.49) | | 20.94(-14.92, 77.56) | |
| **Age-standardized mortality rate** | | | | | | | | | | | | |
| Rate per 100,000 | 0.005(0.001, 0.012) | 0.01(0.002, 0.023) | 0.005(0.001, 0.015) | 0.015(0.003, 0.037) | 0.005(0.001, 0.013) | 0.007(0.002, 0.016) | 0.004(0.001, 0.011) | 0.009(0.002, 0.021) | 0.001(0, 0.004) | 0.005(0.001, 0.011) | 0.002(0, 0.006) | 0.003(0.001, 0.007) |
| Percentage change of rate 1990-2021,% | 108.72(71.55, 205.55) | | 181.6(112.54, 371.39) | | 42.16(14.83, 140.8) | | 110.18(73.62, 175.72) | | 223.26(136.38, 500.49) | | 19.55(-7.98, 66.33) | |
| **APC model estimate** | | | | | | | | | | | | |
| Net drift of mortality(% per year) | 3.66 (2.78, 4.54) | | 5.84 (4.14, 7.56) | | 2.35 (-0.11, 4.87) | | 2.96 (1.62, 4.32) | | 4.89 (1.7, 8.19) | | 0.63 (-3.77, 5.23) | |
| **Chronic kidney disease due to glomerulonephritis** | | | | | | | | | | | | |
| **Deaths** | | | | | | | | | | | | |
| Number | 94.61(17.81, 259.97) | 433.21(97.9, 1063.18) | 28.35(3.8, 82.08) | 141.05(30.25, 352.74) | 22.76(3.73, 63.06) | 61.61(13.87, 145.56) | 33.93(6.62, 87.91) | 179.95(39.51, 431.76) | 4.89(0.49, 16.6) | 38.3(5.55, 109.91) | 4.44(0.81, 11.95) | 11.78(1.92, 32.6) |
| Percentage of global,% | 100 | 100 | 29.97 | 32.56 | 24.06 | 14.22 | 35.86 | 41.54 | 5.17 | 8.84 | 4.69 | 2.72 |
| Percentage change of rate 1990-2021,% | 357.87(288.59, 542.88) | | 397.61(295.03, 759.53) | | 170.65(118.52, 364.98) | | 430.34(309.23, 662.13) | | 683.52(472.7, 1365.83) | | 165.49(59.1, 274.02) | |
| **All-age mortality rate** | | | | | | | | | | | | |
| Rate per 100,000 | 0.002(0, 0.005) | 0.005(0.001, 0.013) | 0.003(0, 0.009) | 0.013(0.003, 0.032) | 0.002(0, 0.006) | 0.005(0.001, 0.011) | 0.002(0, 0.005) | 0.007(0.002, 0.018) | 0(0, 0.001) | 0.002(0, 0.006) | 0.001(0, 0.002) | 0.001(0, 0.003) |
| Percentage change of rate 1990-2021,% | 209.47(162.64, 334.51) | | 300.04(217.58, 591) | | 120.74(78.22, 279.23) | | 273.17(187.95, 436.27) | | 373.68(246.23, 786.17) | | 19.11(-28.62, 67.8) | |
| **Age-standardized mortality rate** | | | | | | | | | | | | |
| Rate per 100,000 | 0.003(0.001, 0.007) | 0.005(0.001, 0.013) | 0.003(0, 0.008) | 0.007(0.002, 0.019) | 0.003(0, 0.007) | 0.003(0.001, 0.008) | 0.003(0.001, 0.007) | 0.007(0.002, 0.016) | 0.001(0, 0.002) | 0.003(0, 0.007) | 0.002(0, 0.005) | 0.002(0, 0.006) |
| Percentage change of rate 1990-2021,% | 103.3(74.08, 180.39) | | 175.54(117.17, 349.24) | | 24.31(4.99, 95.01) | | 128.12(83.19, 216.54) | | 252.27(180.45, 512.38) | | 19.45(-21.5, 67.01) | |
| **APC model estimate** | | | | | | | | | | | | |
| Net drift of mortality(% per year) | 2.85 (2.04, 3.66) | | 4.49 (2.7, 6.31) | | 0.82 (-1.2, 2.88) | | 2.72 (1.49, 3.97) | | 4.63 (1.4, 7.97) | | 0.78 (-3.46, 5.21) | |
| **Chronic kidney disease due to other and unspecified causes** | | | | | | | | | | | | |
| **Deaths** | | | | | | | | | | | | |
| Number | 156.86(25.61, 409.38) | 707.27(159.33, 1622.79) | 62.36(8.36, 170.5) | 285.69(63.29, 668.49) | 58.9(9.47, 147.55) | 199.55(52.38, 437.85) | 26.6(5.06, 65.14) | 161.7(32.73, 388.43) | 5.59(0.48, 18.13) | 50.54(7.14, 141.18) | 3(0.53, 8.27) | 8.66(1.35, 23.96) |
| Percentage of global,% | 100 | 100 | 39.76 | 40.39 | 37.55 | 28.21 | 16.96 | 22.86 | 3.56 | 7.15 | 1.91 | 1.22 |
| Percentage change of rate 1990-2021,% | 350.9(270.72, 617.95) | | 358.16(240.11, 755.13) | | 238.8(164.39, 541.57) | | 507.8(387.7, 704.53) | | 804.85(553.43, 1756.7) | | 189.18(90.29, 301.06) | |
| **All-age mortality rate** | | | | | | | | | | | | |
| Rate per 100,000 | 0.003(0, 0.008) | 0.009(0.002, 0.021) | 0.007(0.001, 0.019) | 0.026(0.006, 0.061) | 0.006(0.001, 0.014) | 0.015(0.004, 0.034) | 0.002(0, 0.004) | 0.007(0.001, 0.016) | 0(0, 0.002) | 0.003(0, 0.007) | 0.001(0, 0.002) | 0.001(0, 0.002) |
| Percentage change of rate 1990-2021,% | 204.76(150.56, 385.25) | | 268.33(173.42, 587.46) | | 176.32(115.63, 423.24) | | 327.68(243.16, 466.11) | | 447.03(295.03, 1022.47) | | 29.74(-14.63, 79.93) | |
| **Age-standardized mortality rate** | | | | | | | | | | | | |
| Rate per 100,000 | 0.005(0.001, 0.013) | 0.009(0.002, 0.02) | 0.006(0.001, 0.016) | 0.012(0.003, 0.029) | 0.008(0.002, 0.02) | 0.011(0.003, 0.024) | 0.003(0.001, 0.007) | 0.006(0.001, 0.015) | 0.001(0, 0.003) | 0.004(0.001, 0.01) | 0.001(0, 0.004) | 0.002(0, 0.005) |
| Percentage change of rate 1990-2021,% | 72.23(45.06, 161.54) | | 103.62(58.67, 251.67) | | 34.47(9.13, 125.75) | | 115.92(73.01, 192.29) | | 251.93(150.43, 546.86) | | 27.81(-6.98, 77.11) | |
| **APC model estimate** | | | | | | | | | | | | |
| Net drift of mortality(% per year) | 2.63 (1.86, 3.42) | | 3.14 (1.53, 4.78) | | 1.68 (0.12, 3.27) | | 3.06 (1.72, 4.41) | | 5.02 (1.99, 8.15) | | 0.89 (-4.2, 6.24) | |
| **Chronic kidney disease due to diabetes mellitus type 2** | | | | | | | | | | | | |
| **Deaths** | | | | | | | | | | | | |
| Number | 1102.22(581.76, 1752.02) | 4877.33(2566.43, 7307.8) | 481.05(249.77, 755.47) | 2131.54(1117.08, 3211.29) | 227.21(118.59, 352.28) | 695.39(365.68, 1082.19) | 261.22(134.42, 417.28) | 1519.84(767.92, 2356.93) | 95.05(48.38, 155.55) | 439.05(216.43, 702.55) | 36.32(18.3, 61.82) | 87.33(41.54, 141.91) |
| Percentage of global,% | 100 | 100 | 43.64 | 43.70 | 20.61 | 14.26 | 23.70 | 31.16 | 8.62 | 9.00 | 3.30 | 1.79 |
| Percentage change of rate 1990-2021,% | 342.5(288.04, 396.78) | | 343.1(261.07, 440.97) | | 206.06(159.1, 259.81) | | 481.83(407.42, 561.97) | | 361.91(273.6, 459.43) | | 140.42(97.85, 190.92) | |
| **All-age mortality rate** | | | | | | | | | | | | |
| Rate per 100,000 | 0.021(0.011, 0.033) | 0.062(0.033, 0.093) | 0.055(0.028, 0.086) | 0.195(0.102, 0.294) | 0.021(0.011, 0.033) | 0.053(0.028, 0.083) | 0.015(0.008, 0.024) | 0.062(0.031, 0.096) | 0.008(0.004, 0.013) | 0.023(0.011, 0.037) | 0.007(0.004, 0.012) | 0.008(0.004, 0.013) |
| Percentage change of rate 1990-2021,% | 199.08(162.27, 235.77) | | 256.22(190.28, 334.9) | | 149.62(111.32, 193.45) | | 309.4(257.04, 365.79) | | 179.25(125.86, 238.2) | | 7.86(-11.24, 30.52) | |
| **Age-standardized mortality rate** | | | | | | | | | | | | |
| Rate per 100,000 | 0.032(0.017, 0.052) | 0.059(0.031, 0.088) | 0.044(0.023, 0.069) | 0.096(0.051, 0.144) | 0.028(0.014, 0.044) | 0.036(0.019, 0.057) | 0.031(0.016, 0.048) | 0.06(0.031, 0.094) | 0.018(0.009, 0.028) | 0.033(0.016, 0.051) | 0.018(0.009, 0.03) | 0.02(0.01, 0.032) |
| Percentage change of rate 1990-2021,% | 80.78(59.75, 101.72) | | 119.2(80.91, 163.56) | | 31.67(12.55, 53.76) | | 96.82(71.65, 123.47) | | 85.89(48.01, 124.5) | | 8.84(-9.72, 30.3) | |
| **APC model estimate** | | | | | | | | | | | | |
| Net drift of mortality(% per year) | 2.46 (2.15, 2.78) | | 3.54 (2.99, 4.1) | | 1.56 (0.74, 2.4) | | 2.49 (1.96, 3.03) | | 2.37 (1.46, 3.28) | | 0.49 (-1.18, 2.19) | |

**Appendix 3** The mortality number, all-age mortality rate, ASMRs, DALYs, age-standardized DALYs rate and net drift of mortality from 1990 to 2021 for SSBs-related CKD across countries

| Country | Mortality number | | | All-age mortality rate | | | ASMRs | | | DALYs | | | Age-standardized DALYs rate | | | Net drift |
| --- | --- | --- | --- | --- | --- | --- | --- | --- | --- | --- | --- | --- | --- | --- | --- | --- |
|  | 1990 | 2021 | Percentage change | 1990 | 2021 | Percentage change | 1990 | 2021 | Percentage change | 1990 | 2021 | Percentage change | 1990 | 2021 | Percentage change | 1990 to 2021 |
| Afghanistan | 1.84 (0.69, 3.55) | 2.45 (0.77, 5.66) | 33.08 (-35.75, 149.27) | 0.019 (0.007, 0.036) | 0.008 (0.002, 0.018) | -57.62 (-79.54, -20.61) | 0.029 (0.011, 0.057) | 0.025 (0.008, 0.058) | -12.35 (-55.52, 54.33) | 56.37 (20.9, 113.36) | 87.27 (28.01, 204.41) | 54.81 (-21.12, 183.6) | 0.8 (0.31, 1.65) | 0.69 (0.24, 1.57) | -14.69 (-55.38, 51.06) | 0.96 (-6.99, 9.58) |
| Albania | 0.63 (0.24, 1.27) | 2.26 (0.96, 4.34) | 256.18 (116.86, 475.39) | 0.019 (0.007, 0.039) | 0.085 (0.036, 0.163) | 341.27 (168.67, 612.85) | 0.036 (0.014, 0.069) | 0.055 (0.023, 0.107) | 55.92 (-4.89, 148.49) | 25.43 (9.31, 50.25) | 70.5 (29.35, 133.15) | 177.26 (91.21, 300.59) | 1.16 (0.44, 2.21) | 1.79 (0.71, 3.48) | 53.9 (6.04, 120.24) | 3.98 (-9.74, 19.78) |
| Algeria | 3.62 (1.47, 7.45) | 29.17 (11.63, 52.61) | 705.1 (303.46, 1315.27) | 0.014 (0.006, 0.029) | 0.066 (0.026, 0.119) | 360.64 (130.84, 709.75) | 0.039 (0.016, 0.081) | 0.098 (0.038, 0.179) | 150.32 (23.44, 361.57) | 125.31 (50.29, 250.36) | 880.51 (352.64, 1644.44) | 602.64 (287.92, 1055.97) | 0.98 (0.42, 1.9) | 2.35 (0.98, 4.25) | 139.63 (31.53, 286.95) | 3.67 (-0.05, 7.53) |
| American Samoa | 0.04 (0.02, 0.07) | 0.29 (0.13, 0.51) | 582.66 (278.5, 1190.82) | 0.088 (0.036, 0.15) | 0.584 (0.269, 1.02) | 565.43 (268.95, 1158.25) | 0.218 (0.094, 0.378) | 0.687 (0.316, 1.18) | 215.24 (79.06, 493.77) | 1.41 (0.57, 2.49) | 7.45 (3.4, 13.31) | 429.56 (200.76, 867.28) | 5.58 (2.31, 9.46) | 15.62 (7.15, 27.14) | 179.89 (58.94, 414.08) | -2.51 (-18.04, 15.98) |
| Andorra | 0.05 (0.02, 0.11) | 0.14 (0.06, 0.27) | 157.64 (39.46, 371.28) | 0.1 (0.039, 0.2) | 0.164 (0.066, 0.312) | 63.63 (-11.43, 199.32) | 0.126 (0.048, 0.251) | 0.076 (0.031, 0.143) | -39.96 (-65.99, 5.25) | 1.69 (0.68, 3.28) | 3.34 (1.41, 6.12) | 97.22 (34.86, 198.5) | 3.12 (1.28, 5.95) | 2.17 (0.89, 4.05) | -30.39 (-52.03, 2.87) | -2.33 (-13.42, 10.18) |
| Angola | 0.51 (0.18, 1) | 3.61 (1.31, 7.43) | 609.38 (303.75, 1003.88) | 0.005 (0.002, 0.01) | 0.011 (0.004, 0.023) | 122.89 (26.86, 246.84) | 0.016 (0.006, 0.029) | 0.036 (0.014, 0.07) | 128.89 (39.16, 252.19) | 18.89 (7.08, 38.55) | 137.51 (47.06, 290.63) | 627.93 (331.82, 999.87) | 0.43 (0.17, 0.83) | 0.97 (0.36, 1.88) | 125.2 (38.81, 228.79) | 3.25 (-6.18, 13.63) |
| Antigua and Barbuda | 0.02 (0.01, 0.04) | 0.08 (0.04, 0.12) | 203.04 (98.8, 348.09) | 0.041 (0.019, 0.07) | 0.085 (0.041, 0.139) | 104.16 (33.93, 201.89) | 0.046 (0.021, 0.078) | 0.076 (0.037, 0.124) | 63.2 (10.1, 144.79) | 0.68 (0.29, 1.17) | 2.12 (0.96, 3.55) | 212.64 (110.67, 363.85) | 1.3 (0.58, 2.3) | 1.96 (0.9, 3.29) | 50.57 (1.27, 121.18) | -5.59 (-17.43, 7.94) |
| Argentina | 96.34 (43.01, 169.66) | 182.46 (82.71, 317.29) | 89.39 (35.87, 149.44) | 0.291 (0.13, 0.512) | 0.401 (0.182, 0.698) | 37.86 (-1.09, 81.57) | 0.318 (0.147, 0.564) | 0.319 (0.144, 0.55) | 0.29 (-26.16, 30.49) | 2419.88 (1041.7, 4378.81) | 4181.84 (1858.47, 7329.8) | 72.81 (32.78, 115.76) | 7.62 (3.33, 13.98) | 7.65 (3.4, 13.51) | 0.34 (-22.89, 24.29) | -0.05 (-1.26, 1.17) |
| Armenia | 0.01 (0.01, 0.03) | 0.43 (0.18, 0.82) | 3046.42 (1979.52, 5187.85) | 0 (0, 0.001) | 0.014 (0.006, 0.027) | 3493.22 (2274.82, 5938.73) | 0.001 (0, 0.001) | 0.01 (0.004, 0.02) | 1912.79 (1226.56, 3137.4) | 3.79 (1.45, 7.47) | 18.52 (7.82, 36.18) | 388.31 (233.39, 657.02) | 0.13 (0.05, 0.25) | 0.46 (0.19, 0.92) | 251.91 (145.06, 428.66) | -5.66 (-18.72, 9.51) |
| Australia | 9.17 (3.59, 17.58) | 36.66 (15.08, 67.16) | 299.82 (173.08, 519.62) | 0.054 (0.021, 0.104) | 0.142 (0.058, 0.26) | 161.35 (78.5, 305.02) | 0.051 (0.02, 0.097) | 0.068 (0.028, 0.125) | 34.1 (-6.22, 96.58) | 252.65 (102.38, 494.82) | 785.25 (336.7, 1417.39) | 210.8 (134.52, 316.82) | 1.33 (0.53, 2.57) | 1.73 (0.71, 3.21) | 30.23 (-1.31, 67.58) | 3.02 (-3.5, 9.98) |
| Austria | 3.51 (1.45, 6.51) | 29.81 (12.09, 60.19) | 750.14 (447.89, 1209.9) | 0.045 (0.019, 0.084) | 0.332 (0.135, 0.67) | 635.23 (373.84, 1032.84) | 0.029 (0.012, 0.055) | 0.121 (0.052, 0.239) | 312.71 (178.6, 540.78) | 103.25 (41.02, 189.72) | 466.28 (210.1, 882.37) | 351.62 (221.73, 548.21) | 0.89 (0.37, 1.62) | 2.28 (1.03, 4.26) | 156.18 (93.53, 243.7) | 1.69 (-8.82, 13.4) |
| Azerbaijan | 0.36 (0.14, 0.77) | 2.38 (0.9, 4.75) | 567.5 (282.34, 1017.74) | 0.005 (0.002, 0.011) | 0.023 (0.009, 0.045) | 365.75 (166.77, 679.9) | 0.007 (0.003, 0.015) | 0.023 (0.009, 0.044) | 235.1 (99.67, 460.19) | 25.8 (10.16, 55.41) | 139.25 (52.08, 266.47) | 439.64 (253.86, 729.21) | 0.46 (0.19, 0.95) | 1.21 (0.47, 2.26) | 163.65 (76.16, 285.28) | 6.24 (-5.56, 19.51) |
| Bahamas | 0.12 (0.05, 0.2) | 0.37 (0.17, 0.64) | 212.29 (102.73, 422.66) | 0.047 (0.021, 0.079) | 0.096 (0.043, 0.166) | 106.63 (34.14, 245.82) | 0.075 (0.034, 0.128) | 0.092 (0.041, 0.156) | 23.38 (-20.61, 105.06) | 4.26 (1.88, 7.38) | 12.72 (5.38, 22.17) | 198.78 (88.45, 392.99) | 2.32 (1.04, 4.03) | 2.92 (1.25, 5.13) | 25.97 (-18.7, 109.87) | -0.95 (-19.23, 21.47) |
| Bahrain | 0.15 (0.07, 0.3) | 1.38 (0.54, 2.51) | 790.16 (404.47, 1479.91) | 0.031 (0.013, 0.059) | 0.09 (0.035, 0.164) | 194.74 (67.03, 423.12) | 0.113 (0.047, 0.219) | 0.228 (0.09, 0.428) | 101.93 (9.51, 273.35) | 6.31 (2.55, 12.42) | 47.85 (18.07, 90.85) | 658.95 (334.17, 1181.51) | 2.69 (1.15, 5.09) | 4.81 (1.91, 8.66) | 78.84 (4.17, 201.3) | 0.85 (-13.86, 18.08) |
| Bangladesh | 1.45 (0.61, 2.78) | 6.74 (2.63, 12.14) | 363.2 (190.36, 619.3) | 0.001 (0.001, 0.003) | 0.004 (0.002, 0.007) | 207.01 (92.45, 376.75) | 0.003 (0.001, 0.006) | 0.006 (0.002, 0.01) | 66.49 (6.94, 155.32) | 52.54 (21.63, 103.59) | 234.05 (87.99, 438.31) | 345.49 (196.21, 584.26) | 0.1 (0.04, 0.19) | 0.17 (0.06, 0.3) | 64.18 (9.63, 144.83) | 2.18 (-4.42, 9.24) |
| Barbados | 0.45 (0.2, 0.74) | 0.82 (0.37, 1.41) | 82.43 (19.13, 186.41) | 0.177 (0.081, 0.291) | 0.273 (0.123, 0.47) | 54.6 (0.96, 142.72) | 0.162 (0.075, 0.261) | 0.167 (0.079, 0.286) | 3.39 (-32.93, 58.98) | 12.7 (5.71, 21.18) | 20.81 (9.93, 36.01) | 63.8 (9.22, 150.96) | 4.85 (2.24, 8.1) | 4.66 (2.15, 8.06) | -3.96 (-35.49, 43.19) | 0.59 (-14.12, 17.81) |
| Belarus | 0.09 (0.03, 0.21) | 0.44 (0.16, 0.91) | 379.69 (188.52, 680.36) | 0.001 (0, 0.002) | 0.005 (0.002, 0.01) | 437.3 (223.17, 774.07) | 0.001 (0, 0.002) | 0.003 (0.001, 0.006) | 267.38 (119.6, 480.68) | 24.68 (9.07, 49.31) | 38.86 (15.23, 82.13) | 57.48 (-1.33, 138.65) | 0.2 (0.07, 0.4) | 0.26 (0.1, 0.56) | 30.71 (-16.78, 87.32) | -0.17 (-15.51, 17.95) |
| Belgium | 9.83 (4.13, 18.8) | 23.73 (9.91, 43.2) | 141.54 (58.06, 275.79) | 0.098 (0.041, 0.188) | 0.207 (0.086, 0.377) | 110.15 (37.52, 226.95) | 0.064 (0.026, 0.12) | 0.074 (0.032, 0.134) | 17.31 (-20.29, 69.71) | 278.77 (122.13, 518.99) | 520.11 (228.3, 926.8) | 86.58 (42.54, 153.5) | 1.89 (0.83, 3.55) | 2.27 (0.99, 4.14) | 19.94 (-6, 54.5) | 1.21 (-6.56, 9.62) |
| Belize | 0.07 (0.03, 0.12) | 0.55 (0.26, 0.94) | 679.23 (421.28, 1057.05) | 0.038 (0.017, 0.066) | 0.128 (0.06, 0.219) | 239.46 (127.09, 404.06) | 0.074 (0.034, 0.132) | 0.176 (0.085, 0.294) | 136.12 (59.56, 250.63) | 2.11 (0.86, 3.77) | 18.84 (8.58, 33.95) | 793.24 (501.21, 1249.95) | 2.1 (0.87, 3.67) | 5.27 (2.47, 9.26) | 151.13 (70.5, 279.63) | -2.26 (-18.16, 16.74) |
| Benin | 0.17 (0.07, 0.32) | 0.96 (0.39, 1.83) | 483.3 (295.55, 771.42) | 0.003 (0.001, 0.007) | 0.007 (0.003, 0.014) | 109.6 (42.13, 213.13) | 0.009 (0.004, 0.018) | 0.022 (0.009, 0.041) | 134.2 (56.81, 243.09) | 4.59 (1.88, 9.43) | 30.54 (11.8, 59.6) | 565.58 (353, 873.46) | 0.23 (0.1, 0.44) | 0.54 (0.22, 1.03) | 137.87 (64.11, 241.57) | -1 (-14.72, 14.92) |
| Bermuda | 0.05 (0.02, 0.08) | 0.08 (0.04, 0.14) | 50.46 (-2.6, 143.34) | 0.086 (0.04, 0.143) | 0.121 (0.055, 0.221) | 40.62 (-8.98, 127.41) | 0.086 (0.041, 0.143) | 0.055 (0.025, 0.098) | -36.55 (-57.95, -0.32) | 1.46 (0.66, 2.59) | 1.74 (0.79, 3.02) | 19.34 (-19.01, 85.9) | 2.29 (1.05, 4.05) | 1.49 (0.68, 2.59) | -35.05 (-56.32, -2.61) | -4.03 (-15.12, 8.51) |
| Bhutan | 0.02 (0.01, 0.03) | 0.08 (0.03, 0.15) | 357.14 (183.77, 640.46) | 0.003 (0.001, 0.005) | 0.01 (0.004, 0.02) | 280.53 (136.21, 516.37) | 0.008 (0.003, 0.015) | 0.014 (0.005, 0.026) | 71.99 (1.77, 178.99) | 0.67 (0.26, 1.25) | 2.59 (1, 4.95) | 288.14 (159.83, 486.47) | 0.23 (0.1, 0.42) | 0.39 (0.15, 0.74) | 67.72 (10.76, 150.51) | -6.73 (-18.34, 6.54) |
| Bolivia (Plurinational State of) | 4.69 (2.02, 8.02) | 23.51 (9.94, 41.36) | 401.05 (202.87, 764.65) | 0.074 (0.032, 0.126) | 0.199 (0.084, 0.351) | 171.02 (63.82, 367.69) | 0.163 (0.075, 0.288) | 0.278 (0.118, 0.479) | 70.55 (4.16, 186.89) | 133.64 (54.97, 247.82) | 639.25 (275.01, 1168.41) | 378.36 (194.12, 724.43) | 3.97 (1.72, 7.1) | 6.72 (2.94, 12.14) | 69.52 (4.95, 189.34) | 2 (-1.76, 5.89) |
| Bosnia and Herzegovina | 0.83 (0.33, 1.77) | 2.47 (0.99, 4.97) | 196.12 (75.68, 414.41) | 0.019 (0.007, 0.039) | 0.075 (0.03, 0.15) | 303.29 (139.27, 600.59) | 0.023 (0.009, 0.047) | 0.04 (0.016, 0.078) | 71.44 (2.43, 189.91) | 32.06 (12.69, 67.43) | 71.2 (29.61, 143.37) | 122.08 (40.77, 270.16) | 0.77 (0.32, 1.57) | 1.27 (0.5, 2.61) | 65.38 (5.35, 151.91) | 2.36 (-9.78, 16.13) |
| Botswana | 0.08 (0.03, 0.17) | 0.36 (0.15, 0.78) | 352.48 (159.55, 665.1) | 0.006 (0.002, 0.013) | 0.015 (0.006, 0.033) | 149.42 (43.07, 321.74) | 0.018 (0.007, 0.035) | 0.029 (0.012, 0.06) | 64.29 (-1.96, 173.29) | 3.29 (1.33, 6.8) | 14.47 (5.86, 30.73) | 340.37 (161.12, 569.98) | 0.55 (0.23, 1.13) | 0.86 (0.37, 1.81) | 56.16 (-5.47, 137.96) | -2.99 (-19.2, 16.47) |
| Brazil | 79.37 (37.55, 138.21) | 413.23 (202.32, 683.31) | 420.64 (307.56, 565.02) | 0.053 (0.025, 0.093) | 0.188 (0.092, 0.31) | 250.88 (174.67, 348.19) | 0.094 (0.046, 0.163) | 0.168 (0.083, 0.277) | 78.84 (43.31, 122.42) | 3021.59 (1313.36, 5475.77) | 12122.21 (5560.9, 20354.76) | 301.19 (217.14, 415.76) | 2.95 (1.35, 5.23) | 4.8 (2.21, 8.07) | 62.7 (31.74, 102.07) | 1.17 (0.33, 2.02) |
| Brunei Darussalam | 0.05 (0.02, 0.08) | 0.16 (0.07, 0.29) | 238.76 (115.25, 439.64) | 0.018 (0.008, 0.033) | 0.035 (0.015, 0.065) | 94.68 (23.7, 210.12) | 0.056 (0.024, 0.103) | 0.059 (0.025, 0.114) | 5.62 (-34.7, 70.24) | 1.46 (0.61, 2.66) | 4.95 (2.02, 9.18) | 239.95 (121.9, 406.27) | 1.24 (0.53, 2.23) | 1.34 (0.58, 2.48) | 8.04 (-30.5, 62.13) | -8.12 (-22.8, 9.36) |
| Bulgaria | 7.28 (2.73, 13.42) | 16.3 (6.59, 31.16) | 123.87 (56.47, 227.83) | 0.084 (0.031, 0.155) | 0.24 (0.097, 0.459) | 186.31 (100.11, 319.28) | 0.076 (0.031, 0.138) | 0.123 (0.05, 0.233) | 62.1 (14.35, 133.28) | 307.47 (123.47, 569.49) | 486.21 (196.9, 937.64) | 58.13 (15.97, 125.99) | 2.83 (1.17, 5.27) | 4.19 (1.64, 8.29) | 48.15 (7.94, 109.36) | 4.59 (0.19, 9.18) |
| Burkina Faso | 0.29 (0.11, 0.57) | 1.71 (0.68, 3.39) | 494.77 (296.08, 845.85) | 0.003 (0.001, 0.006) | 0.007 (0.003, 0.015) | 148.98 (65.8, 295.95) | 0.008 (0.004, 0.017) | 0.022 (0.009, 0.043) | 164.15 (76.86, 309.01) | 8.61 (3.41, 17.47) | 53.9 (20.85, 111.3) | 525.83 (327.13, 874.21) | 0.2 (0.08, 0.4) | 0.54 (0.21, 1.06) | 165.6 (86.71, 304.18) | 1.83 (-10.32, 15.62) |
| Burundi | 0.54 (0.23, 1.07) | 0.94 (0.4, 1.97) | 73.69 (5.58, 185.03) | 0.01 (0.004, 0.019) | 0.007 (0.003, 0.015) | -27.04 (-55.65, 19.73) | 0.027 (0.011, 0.052) | 0.025 (0.011, 0.05) | -7.99 (-43.47, 48.59) | 14.47 (5.93, 28.64) | 26.55 (10.63, 57.53) | 83.49 (14.38, 201.39) | 0.62 (0.26, 1.21) | 0.54 (0.22, 1.09) | -12.93 (-46.18, 42.22) | -2.44 (-14.52, 11.34) |
| Cabo Verde | 0.01 (0.01, 0.03) | 0.08 (0.03, 0.16) | 499.76 (271.22, 820.06) | 0.004 (0.002, 0.007) | 0.015 (0.005, 0.028) | 279.43 (134.84, 482.06) | 0.006 (0.002, 0.011) | 0.018 (0.007, 0.034) | 208.02 (90.86, 382.54) | 0.47 (0.18, 0.93) | 2.66 (0.96, 5.25) | 469.47 (269.86, 728.51) | 0.2 (0.08, 0.39) | 0.52 (0.19, 1) | 158.75 (70.95, 278.21) | -3.93 (-13.53, 6.73) |
| Cambodia | 0.39 (0.17, 0.67) | 2.03 (0.82, 3.73) | 420.88 (211.49, 763.66) | 0.004 (0.002, 0.007) | 0.012 (0.005, 0.022) | 213.94 (87.74, 420.55) | 0.01 (0.004, 0.018) | 0.019 (0.008, 0.033) | 85.08 (13.85, 207.54) | 12.64 (5.34, 22.51) | 65.26 (26.6, 121.76) | 416.28 (222.21, 713.07) | 0.27 (0.11, 0.48) | 0.5 (0.21, 0.91) | 85.95 (19.08, 193.04) | 1.29 (-9.65, 13.57) |
| Cameroon | 0.79 (0.32, 1.49) | 4.54 (1.74, 9.01) | 473.52 (258.97, 815.41) | 0.008 (0.003, 0.014) | 0.014 (0.005, 0.028) | 88.35 (17.89, 200.63) | 0.023 (0.01, 0.043) | 0.043 (0.017, 0.082) | 88.82 (20.95, 195.59) | 22.97 (8.64, 43.85) | 156.38 (57.96, 334.62) | 580.92 (326.39, 961.51) | 0.52 (0.21, 0.97) | 1.08 (0.42, 2.07) | 107.67 (35.84, 220.05) | 3.23 (-5.28, 12.49) |
| Canada | 7.93 (3.69, 14.61) | 39.34 (18.01, 68.39) | 396.06 (247.48, 653.54) | 0.029 (0.014, 0.054) | 0.105 (0.048, 0.183) | 260.81 (152.73, 448.08) | 0.025 (0.012, 0.046) | 0.049 (0.023, 0.084) | 96.83 (40.7, 188.63) | 197.22 (88.27, 372.15) | 855.59 (395.61, 1552.9) | 333.82 (221.16, 519.06) | 0.61 (0.27, 1.16) | 1.28 (0.59, 2.35) | 109.08 (53.83, 195.97) | 3.64 (-1.16, 8.67) |
| Central African Republic | 0.49 (0.18, 0.92) | 0.97 (0.34, 2.02) | 97.87 (14.09, 227.49) | 0.018 (0.007, 0.034) | 0.018 (0.006, 0.037) | -1.48 (-43.19, 63.06) | 0.046 (0.019, 0.086) | 0.044 (0.017, 0.087) | -4.51 (-43.08, 44.4) | 18.98 (6.9, 36.31) | 41.05 (14.73, 87.24) | 116.25 (25.17, 259.78) | 1.35 (0.52, 2.54) | 1.35 (0.49, 2.76) | -0.47 (-39.91, 55.07) | -0.1 (-11.02, 12.17) |
| Chad | 0.18 (0.07, 0.35) | 0.67 (0.28, 1.35) | 280.7 (145.16, 482.35) | 0.003 (0.001, 0.006) | 0.004 (0.002, 0.008) | 29.26 (-16.76, 97.73) | 0.007 (0.003, 0.014) | 0.013 (0.006, 0.026) | 84.45 (21.7, 184.14) | 5.28 (2.15, 10.54) | 23.92 (9.54, 47.71) | 353.12 (204.7, 562.38) | 0.18 (0.08, 0.35) | 0.36 (0.15, 0.7) | 92.85 (33.09, 183.48) | -1.07 (-15.48, 15.79) |
| Chile | 5.45 (2.44, 9.75) | 34.03 (14.89, 56.92) | 524.07 (354.85, 752.02) | 0.041 (0.018, 0.073) | 0.181 (0.079, 0.303) | 341.07 (221.47, 502.17) | 0.06 (0.028, 0.107) | 0.13 (0.057, 0.217) | 115.52 (59.36, 186.83) | 164.11 (70.9, 307.06) | 795.18 (363.45, 1409.88) | 384.53 (270.72, 533.81) | 1.61 (0.69, 2.93) | 3.18 (1.43, 5.79) | 98.2 (52.42, 149.42) | 2.1 (-1.76, 6.12) |
| China | 64.85 (29.49, 114.48) | 541.51 (245.08, 931.91) | 735.03 (475.35, 1069.77) | 0.006 (0.003, 0.01) | 0.038 (0.017, 0.066) | 590.48 (375.75, 867.28) | 0.01 (0.005, 0.019) | 0.028 (0.013, 0.048) | 171.5 (85.87, 287.73) | 2062.76 (930.31, 3777.55) | 14917.22 (6796.87, 25713.21) | 623.17 (427.08, 890.37) | 0.25 (0.12, 0.45) | 0.72 (0.32, 1.26) | 186.28 (113.14, 281.13) | 3.87 (2.81, 4.94) |
| Colombia | 7.7 (3.39, 14.33) | 38.24 (16.77, 67.41) | 396.79 (266.5, 584.5) | 0.024 (0.01, 0.044) | 0.078 (0.034, 0.137) | 229.01 (142.72, 353.33) | 0.047 (0.022, 0.085) | 0.068 (0.03, 0.119) | 42.81 (3.02, 98.09) | 284.34 (122.28, 541.12) | 1209.05 (520.92, 2234.9) | 325.21 (217.37, 470.44) | 1.41 (0.62, 2.58) | 2.17 (0.94, 4.01) | 53.63 (15.15, 101.65) | 0.97 (-1.93, 3.95) |
| Comoros | 0.06 (0.03, 0.12) | 0.18 (0.07, 0.35) | 187.22 (65.2, 374.06) | 0.014 (0.006, 0.027) | 0.024 (0.009, 0.048) | 78.48 (2.66, 194.59) | 0.039 (0.016, 0.074) | 0.043 (0.016, 0.087) | 10.81 (-35.91, 82.2) | 1.8 (0.71, 3.45) | 4.82 (1.84, 9.12) | 168.24 (64.55, 340.92) | 0.9 (0.37, 1.69) | 0.97 (0.37, 1.84) | 7.47 (-34.52, 70.74) | -3.45 (-19.46, 15.74) |
| Congo | 0.44 (0.16, 0.87) | 1.33 (0.49, 2.63) | 200.21 (83.02, 391.51) | 0.018 (0.007, 0.036) | 0.025 (0.009, 0.049) | 33.73 (-18.47, 118.95) | 0.049 (0.019, 0.094) | 0.056 (0.022, 0.105) | 13.93 (-25.97, 76.04) | 14.71 (5.46, 28.42) | 48.85 (16.93, 100.97) | 232.17 (106.5, 436.18) | 1.28 (0.49, 2.42) | 1.48 (0.54, 2.9) | 15.85 (-23.89, 79.3) | 0.36 (-10.69, 12.78) |
| Cook Islands | 0.01 (0, 0.02) | 0.06 (0.03, 0.11) | 545.55 (261.4, 991.83) | 0.053 (0.024, 0.09) | 0.365 (0.165, 0.621) | 588.31 (285.34, 1064.15) | 0.091 (0.043, 0.156) | 0.257 (0.119, 0.434) | 181.25 (57.87, 377.58) | 0.32 (0.14, 0.58) | 1.56 (0.67, 2.75) | 381.63 (181.99, 709.22) | 2.49 (1.09, 4.41) | 6.29 (2.71, 11.17) | 152.69 (51.45, 308.58) | -2.97 (-12.61, 7.72) |
| Costa Rica | 1.37 (0.61, 2.56) | 8.96 (4.02, 15.55) | 552.46 (348.61, 834.96) | 0.045 (0.02, 0.084) | 0.189 (0.085, 0.327) | 317.99 (187.4, 498.97) | 0.08 (0.036, 0.148) | 0.16 (0.072, 0.282) | 99.45 (36.07, 188.69) | 51.77 (22.2, 102.24) | 276.58 (122.09, 528.3) | 434.24 (259.92, 651.88) | 2.67 (1.2, 5.16) | 5.01 (2.21, 9.58) | 87.42 (29.51, 163.58) | 2.43 (-3.88, 9.15) |
| Croatia | 2.46 (0.96, 4.66) | 7.28 (2.93, 13.23) | 196.22 (95.81, 359.83) | 0.051 (0.02, 0.096) | 0.173 (0.07, 0.314) | 242.17 (126.19, 431.15) | 0.045 (0.018, 0.085) | 0.077 (0.031, 0.143) | 71.06 (15.86, 160.38) | 92.85 (37.1, 180.33) | 177.62 (74.78, 325.22) | 91.3 (39.38, 175.72) | 1.59 (0.65, 3.04) | 2.26 (0.92, 4.3) | 42.54 (4.06, 101.89) | 1.45 (-6.19, 9.72) |
| Cuba | 7.55 (3.76, 12.35) | 34.73 (16.92, 55.35) | 359.84 (208.72, 607.83) | 0.07 (0.035, 0.114) | 0.308 (0.15, 0.491) | 342.6 (197.15, 581.29) | 0.076 (0.039, 0.124) | 0.178 (0.086, 0.283) | 134.89 (60.93, 251.08) | 268.58 (126.8, 462.51) | 952.46 (454.61, 1554.21) | 254.63 (146.58, 438.16) | 2.57 (1.22, 4.42) | 5.43 (2.61, 8.87) | 111.11 (46.8, 211.43) | 4.33 (1.23, 7.53) |
| Cyprus | 0.61 (0.24, 1.19) | 0.92 (0.37, 1.74) | 49.89 (-12.39, 147.05) | 0.079 (0.031, 0.152) | 0.068 (0.027, 0.128) | -14.11 (-49.8, 41.57) | 0.136 (0.055, 0.261) | 0.063 (0.025, 0.126) | -53.4 (-73.56, -16.68) | 16.17 (6.28, 30.6) | 23.73 (9.97, 44.82) | 46.74 (4.04, 107.23) | 2.55 (1.02, 4.78) | 1.36 (0.57, 2.59) | -46.65 (-62.89, -22.1) | -2.67 (-15.35, 11.91) |
| Czechia | 5.63 (2.01, 11.03) | 7.51 (3.31, 13.64) | 33.45 (-11.31, 103.17) | 0.055 (0.02, 0.107) | 0.071 (0.031, 0.128) | 29.21 (-14.13, 96.72) | 0.042 (0.015, 0.082) | 0.034 (0.014, 0.062) | -19.79 (-46.08, 18.97) | 212.6 (85.06, 420.57) | 238.14 (104.21, 450.94) | 12.02 (-22.33, 60.88) | 1.65 (0.67, 3.26) | 1.3 (0.53, 2.55) | -21.38 (-43.5, 10.77) | -1.54 (-7.56, 4.88) |
| Côte d'Ivoire | 0.79 (0.31, 1.58) | 3.95 (1.49, 7.58) | 401.5 (220.12, 656.47) | 0.006 (0.003, 0.013) | 0.014 (0.005, 0.027) | 119.56 (40.15, 231.19) | 0.025 (0.01, 0.048) | 0.039 (0.016, 0.07) | 55.27 (2.24, 132.49) | 28.74 (11.49, 57.66) | 150.82 (56.16, 314.34) | 424.83 (234.11, 699.9) | 0.64 (0.28, 1.27) | 1.07 (0.44, 2) | 68.04 (11.78, 148.37) | 2.08 (-5.61, 10.4) |
| Democratic People's Republic of Korea | 1.66 (0.73, 3.01) | 3.13 (1.28, 5.7) | 88.26 (15.75, 200.84) | 0.008 (0.004, 0.015) | 0.012 (0.005, 0.022) | 46.88 (-9.69, 134.72) | 0.012 (0.006, 0.023) | 0.01 (0.004, 0.018) | -18.66 (-49.47, 30.1) | 53.56 (23.56, 95.24) | 95.85 (38.63, 175.59) | 78.97 (14.35, 181.97) | 0.33 (0.15, 0.59) | 0.29 (0.12, 0.52) | -12.56 (-43.53, 33.47) | -0.32 (-8.11, 8.14) |
| Democratic Republic of the Congo | 16.19 (6.47, 29.66) | 19.83 (7.34, 38.83) | 22.52 (-26.96, 110.46) | 0.042 (0.017, 0.078) | 0.022 (0.008, 0.043) | -48.08 (-69.05, -10.81) | 0.118 (0.049, 0.207) | 0.059 (0.023, 0.117) | -50.35 (-69.57, -16.6) | 612.57 (247.98, 1186.99) | 769.64 (288.87, 1527.39) | 25.64 (-21.73, 106.56) | 3.33 (1.37, 6.13) | 1.65 (0.64, 3.19) | -50.4 (-67.75, -19.63) | -2.07 (-4.51, 0.43) |
| Denmark | 2.09 (0.87, 4.06) | 11.78 (5.07, 21.83) | 462.72 (270.03, 807.26) | 0.041 (0.017, 0.079) | 0.201 (0.087, 0.373) | 394.63 (225.26, 697.48) | 0.024 (0.01, 0.046) | 0.082 (0.035, 0.15) | 241.19 (127.87, 447.91) | 74.89 (32.47, 138.49) | 227.78 (104.62, 422.2) | 204.17 (116.88, 338.79) | 0.95 (0.41, 1.79) | 1.9 (0.86, 3.67) | 100.59 (48.45, 181.81) | -0.66 (-12.61, 12.92) |
| Djibouti | 0.01 (0.01, 0.03) | 0.2 (0.08, 0.39) | 1400.29 (775.9, 2366.29) | 0.003 (0.001, 0.006) | 0.016 (0.006, 0.031) | 393.76 (188.27, 711.68) | 0.013 (0.005, 0.025) | 0.042 (0.018, 0.084) | 218.62 (93.92, 414.55) | 0.4 (0.15, 0.78) | 5.75 (2.29, 11.78) | 1353.84 (765.99, 2258.94) | 0.29 (0.12, 0.58) | 0.89 (0.36, 1.77) | 206.73 (88.99, 388.19) | -10.16 (-22.25, 3.81) |
| Dominica | 0.08 (0.04, 0.13) | 0.2 (0.1, 0.33) | 153.17 (66.02, 283.81) | 0.108 (0.052, 0.183) | 0.295 (0.145, 0.49) | 173.34 (79.25, 314.4) | 0.138 (0.067, 0.235) | 0.249 (0.122, 0.412) | 80.01 (16.61, 167.74) | 2.2 (1.01, 3.82) | 5.75 (2.65, 9.84) | 161.8 (75.76, 280.83) | 3.85 (1.75, 6.73) | 7.17 (3.25, 12.47) | 86.19 (27.18, 173.82) | -1.18 (-19.29, 20.98) |
| Dominican Republic | 1.72 (0.78, 3.03) | 10.36 (4.34, 18.84) | 502.51 (239.82, 890.53) | 0.024 (0.011, 0.042) | 0.094 (0.039, 0.171) | 291.22 (120.65, 543.17) | 0.053 (0.024, 0.092) | 0.103 (0.043, 0.185) | 95.68 (10.53, 214.38) | 53.5 (22.89, 99.98) | 319.03 (132.29, 593.12) | 496.27 (257.04, 846.38) | 1.36 (0.61, 2.44) | 3.03 (1.26, 5.55) | 123.45 (32.78, 251.92) | 4.98 (-0.96, 11.28) |
| Ecuador | 12.91 (6.06, 21.47) | 66.27 (26.2, 118.72) | 413.3 (206.84, 745.11) | 0.129 (0.061, 0.215) | 0.367 (0.145, 0.657) | 183.51 (69.48, 366.78) | 0.254 (0.121, 0.429) | 0.416 (0.169, 0.735) | 63.79 (-0.5, 162.32) | 395.14 (177.85, 678.64) | 1871.06 (748.89, 3495.39) | 373.52 (188.47, 705.7) | 6.86 (3.21, 11.67) | 11.23 (4.49, 20.81) | 63.66 (0.52, 173.89) | 0.66 (-1.35, 2.71) |
| Egypt | 12.56 (5.18, 23.28) | 73.98 (32.51, 130.13) | 488.9 (222.93, 789.04) | 0.023 (0.009, 0.042) | 0.07 (0.031, 0.123) | 208.5 (69.17, 365.73) | 0.066 (0.026, 0.128) | 0.143 (0.062, 0.25) | 115.72 (17.18, 247.57) | 346.57 (138.59, 662.5) | 2229.11 (953.52, 3967.78) | 543.19 (278.25, 828.81) | 1.36 (0.57, 2.55) | 3.33 (1.48, 5.8) | 144.47 (39.05, 258.5) | 3.43 (1.19, 5.72) |
| El Salvador | 2.27 (0.9, 4.47) | 17.63 (6.91, 32.21) | 678.24 (290.65, 1171.43) | 0.043 (0.017, 0.084) | 0.273 (0.107, 0.499) | 540.14 (221.33, 945.82) | 0.075 (0.031, 0.146) | 0.276 (0.109, 0.505) | 267.06 (82.57, 505.4) | 74.73 (29.17, 148.52) | 518.11 (195.56, 1006.42) | 593.31 (254.78, 998.9) | 2.29 (0.91, 4.59) | 8.33 (3.15, 16.04) | 264.55 (88.04, 485.05) | 3.93 (-0.12, 8.15) |
| Equatorial Guinea | 0.07 (0.03, 0.15) | 1.47 (0.53, 2.9) | 1930.07 (969.84, 3754.06) | 0.017 (0.007, 0.035) | 0.097 (0.035, 0.192) | 467.56 (199.1, 977.49) | 0.043 (0.017, 0.081) | 0.281 (0.103, 0.568) | 549.81 (250.93, 1036.19) | 2.52 (0.94, 5.14) | 59.92 (21.54, 120.86) | 2275.23 (1177.37, 4191.97) | 1.19 (0.46, 2.37) | 7.8 (2.97, 15.37) | 554.79 (268.43, 1033.11) | 0.8 (-13.36, 17.28) |
| Eritrea | 0.16 (0.06, 0.34) | 0.68 (0.26, 1.46) | 314.25 (164.06, 550.21) | 0.005 (0.002, 0.01) | 0.01 (0.004, 0.022) | 113.84 (36.31, 235.65) | 0.018 (0.007, 0.038) | 0.032 (0.013, 0.067) | 71.52 (6.81, 175.13) | 5.44 (1.99, 11.12) | 20.93 (7.8, 43.07) | 284.43 (151.14, 493.79) | 0.45 (0.17, 0.87) | 0.72 (0.28, 1.49) | 60.17 (5.17, 148.78) | -2.22 (-15.89, 13.67) |
| Estonia | 0.16 (0.06, 0.38) | 1.54 (0.57, 3.13) | 854.12 (465.59, 1510.76) | 0.01 (0.004, 0.024) | 0.117 (0.044, 0.239) | 1041.67 (576.76, 1827.37) | 0.008 (0.003, 0.019) | 0.048 (0.017, 0.099) | 479.69 (253.4, 839.87) | 10.16 (3.91, 22.49) | 36.71 (13.69, 75.25) | 261.25 (135.08, 467.31) | 0.52 (0.2, 1.15) | 1.44 (0.52, 3.18) | 174.61 (80.64, 304.47) | 2.36 (-14.5, 22.55) |
| Eswatini | 0.13 (0.05, 0.23) | 0.41 (0.14, 0.79) | 210.23 (76.09, 411.5) | 0.016 (0.006, 0.029) | 0.035 (0.012, 0.068) | 116.55 (22.92, 257.04) | 0.052 (0.022, 0.091) | 0.075 (0.028, 0.142) | 44.34 (-17.35, 130.81) | 4.7 (1.86, 8.74) | 15.32 (5.45, 30.68) | 225.83 (83.26, 411.99) | 1.48 (0.63, 2.64) | 2.25 (0.84, 4.22) | 52.04 (-7.31, 134.02) | -1.72 (-16.81, 16.12) |
| Ethiopia | 4.6 (1.91, 8.39) | 12.54 (5.73, 22.2) | 172.72 (73.84, 328.91) | 0.009 (0.004, 0.017) | 0.012 (0.005, 0.02) | 26.6 (-19.31, 99.1) | 0.029 (0.012, 0.052) | 0.035 (0.016, 0.061) | 22.2 (-19.82, 84.54) | 125.28 (52.64, 230.03) | 299.45 (132.91, 537.82) | 139.02 (55.87, 258.43) | 0.64 (0.27, 1.16) | 0.71 (0.33, 1.25) | 11.84 (-25.49, 65.25) | 0 (-4.96, 5.21) |
| Fiji | 0.32 (0.13, 0.59) | 2.13 (0.9, 3.94) | 574.9 (206.08, 1246.06) | 0.042 (0.017, 0.078) | 0.231 (0.097, 0.426) | 453.64 (151.08, 1004.22) | 0.096 (0.039, 0.172) | 0.34 (0.147, 0.608) | 253.95 (67.03, 609.02) | 10.91 (4.25, 20.34) | 57.8 (24.19, 105.16) | 429.91 (163.95, 887.83) | 2.63 (1.04, 4.79) | 7.65 (3.21, 13.95) | 190.93 (45.12, 446.35) | 1 (-9.32, 12.48) |
| Finland | 1.2 (0.5, 2.08) | 4.32 (1.8, 8.1) | 259.22 (118.79, 481.38) | 0.024 (0.01, 0.042) | 0.078 (0.033, 0.146) | 225.09 (98.01, 426.14) | 0.017 (0.007, 0.03) | 0.025 (0.01, 0.047) | 45.83 (-6.73, 126.82) | 48.15 (20.36, 91.91) | 103.69 (43.23, 193.33) | 115.35 (53.57, 200.87) | 0.71 (0.3, 1.37) | 0.89 (0.37, 1.71) | 25.4 (-5.1, 62.43) | -0.87 (-12.79, 12.66) |
| France | 38.86 (16.38, 70.58) | 98.56 (38.86, 193.7) | 153.63 (50.12, 292.11) | 0.067 (0.028, 0.122) | 0.148 (0.059, 0.292) | 120.7 (30.63, 241.21) | 0.044 (0.019, 0.082) | 0.046 (0.018, 0.087) | 4.38 (-32.29, 56.33) | 857.17 (355.82, 1605.49) | 1816.68 (791.67, 3481.98) | 111.94 (47.99, 192.49) | 1.04 (0.43, 1.93) | 1.19 (0.52, 2.32) | 14.48 (-14, 51.48) | 0.95 (-3.87, 6.02) |
| Gabon | 0.53 (0.2, 0.99) | 2.26 (0.85, 4.24) | 329.5 (137.16, 602.32) | 0.053 (0.02, 0.1) | 0.124 (0.047, 0.234) | 132.6 (28.44, 280.36) | 0.103 (0.039, 0.197) | 0.244 (0.092, 0.441) | 137.49 (30.81, 298.13) | 16.76 (6.37, 31.67) | 78.13 (30.54, 151.88) | 366.08 (174.53, 663.04) | 2.78 (1.05, 5.16) | 6.41 (2.53, 11.79) | 130.6 (35.34, 262.05) | 2 (-7.15, 12.04) |
| Gambia | 0.05 (0.02, 0.09) | 0.25 (0.09, 0.51) | 450.16 (236.15, 773.44) | 0.005 (0.002, 0.009) | 0.01 (0.004, 0.021) | 125.57 (37.82, 258.11) | 0.016 (0.007, 0.03) | 0.028 (0.011, 0.053) | 76.53 (10.03, 174.19) | 1.48 (0.59, 3.12) | 8.32 (3, 17.35) | 463.24 (246.89, 766.64) | 0.39 (0.16, 0.76) | 0.73 (0.28, 1.42) | 84.13 (19.45, 179.52) | -5.21 (-21.71, 14.77) |
| Georgia | 0.25 (0.1, 0.53) | 1.22 (0.48, 2.37) | 380.64 (201.07, 662) | 0.005 (0.002, 0.01) | 0.034 (0.013, 0.066) | 635.9 (360.97, 1066.67) | 0.004 (0.002, 0.009) | 0.021 (0.008, 0.042) | 406.22 (228.95, 686.05) | 25.88 (11, 52.49) | 50.6 (20.52, 96.46) | 95.51 (28.91, 193.74) | 0.42 (0.18, 0.84) | 0.95 (0.36, 1.84) | 124.79 (49.35, 236.12) | 4.72 (-8.8, 20.24) |
| Germany | 60.73 (25.17, 111.9) | 217.1 (88.36, 406.2) | 257.48 (135.97, 453.48) | 0.076 (0.031, 0.14) | 0.254 (0.103, 0.476) | 234.74 (120.96, 418.27) | 0.046 (0.019, 0.086) | 0.085 (0.035, 0.158) | 86.47 (27.41, 175.83) | 1896.38 (838.25, 3591.52) | 3751.08 (1640.81, 6673.51) | 97.8 (48.52, 174.28) | 1.56 (0.68, 3.02) | 1.93 (0.83, 3.53) | 23.68 (-2.35, 58.75) | 0.03 (-2.93, 3.09) |
| Ghana | 0.45 (0.18, 0.87) | 9.17 (3.53, 17.99) | 1916.32 (1024.55, 3119.58) | 0.003 (0.001, 0.006) | 0.027 (0.01, 0.053) | 781.49 (391.63, 1307.53) | 0.009 (0.004, 0.018) | 0.063 (0.025, 0.118) | 577.92 (271.85, 982.17) | 13.42 (5.15, 26.34) | 293.7 (102.18, 617.07) | 2087.77 (1138.71, 3373.68) | 0.21 (0.09, 0.4) | 1.52 (0.57, 2.97) | 620.09 (316.19, 1026.69) | 7.79 (-1.17, 17.56) |
| Greece | 22.63 (9.75, 42.31) | 49.43 (19.78, 94.32) | 118.49 (40.01, 247.26) | 0.218 (0.094, 0.407) | 0.486 (0.194, 0.927) | 123.09 (42.96, 254.58) | 0.169 (0.074, 0.31) | 0.146 (0.058, 0.278) | -13.21 (-41.76, 34.51) | 429.65 (191.73, 812.92) | 780.43 (325.93, 1465.9) | 81.64 (28.34, 162.23) | 3.03 (1.34, 5.66) | 2.9 (1.21, 5.39) | -4.19 (-28.05, 29.65) | 0.62 (-6.47, 8.26) |
| Greenland | 0.02 (0.01, 0.04) | 0.06 (0.02, 0.1) | 199.89 (102.8, 360.44) | 0.033 (0.015, 0.065) | 0.099 (0.042, 0.177) | 196.99 (100.84, 355.98) | 0.075 (0.032, 0.149) | 0.1 (0.04, 0.181) | 32.87 (-8.8, 101.72) | 0.63 (0.28, 1.2) | 1.8 (0.79, 3.23) | 187.83 (101.83, 324.25) | 1.87 (0.85, 3.53) | 2.7 (1.19, 4.84) | 44.07 (5.29, 106.03) | -4.48 (-16.08, 8.72) |
| Grenada | 0.05 (0.03, 0.09) | 0.14 (0.06, 0.24) | 164.41 (66.08, 289.57) | 0.062 (0.031, 0.1) | 0.139 (0.062, 0.239) | 124.18 (40.81, 230.3) | 0.076 (0.035, 0.123) | 0.131 (0.06, 0.224) | 72.89 (16.12, 156.45) | 1.52 (0.69, 2.56) | 4.39 (1.93, 7.51) | 189.32 (91.93, 317.2) | 2.3 (1.03, 3.93) | 3.78 (1.65, 6.49) | 64.65 (11.25, 139.73) | -6.15 (-21.04, 11.55) |
| Guam | 0.15 (0.07, 0.27) | 0.55 (0.24, 0.95) | 263.69 (122.58, 476.52) | 0.11 (0.049, 0.2) | 0.345 (0.152, 0.598) | 212.54 (91.28, 395.44) | 0.248 (0.117, 0.434) | 0.258 (0.114, 0.452) | 3.8 (-36.53, 66.29) | 4.96 (2.15, 9.17) | 17.53 (7.58, 31.91) | 253.16 (118.07, 428.56) | 6.21 (2.78, 11.3) | 8.71 (3.69, 15.63) | 40.32 (-12.06, 110.49) | 0.59 (-14.1, 17.8) |
| Guatemala | 2.55 (1.01, 4.77) | 15.4 (6.19, 29.08) | 504.03 (302.55, 808.7) | 0.03 (0.012, 0.057) | 0.098 (0.039, 0.184) | 221.19 (114.05, 383.2) | 0.088 (0.038, 0.158) | 0.141 (0.058, 0.264) | 60.68 (2.5, 132.43) | 90.47 (36.91, 173.99) | 530.37 (201.34, 1087.79) | 486.25 (285.9, 780.77) | 2.32 (0.95, 4.33) | 4.31 (1.69, 8.53) | 85.68 (23.11, 172.48) | 3.67 (-0.8, 8.34) |
| Guinea | 0.45 (0.18, 0.87) | 1.34 (0.52, 2.7) | 196.58 (88.34, 354.46) | 0.008 (0.003, 0.015) | 0.01 (0.004, 0.02) | 32.37 (-15.94, 102.84) | 0.015 (0.006, 0.03) | 0.025 (0.01, 0.05) | 63.33 (7.18, 149.54) | 14.21 (5.65, 27.27) | 48.38 (18.12, 100.26) | 240.53 (123.51, 410.49) | 0.42 (0.17, 0.8) | 0.72 (0.29, 1.43) | 72.14 (15.67, 159.56) | 2.3 (-9.98, 16.26) |
| Guinea-Bissau | 0.05 (0.02, 0.1) | 0.12 (0.05, 0.25) | 128.84 (42.29, 272.98) | 0.005 (0.002, 0.01) | 0.006 (0.002, 0.012) | 11.66 (-30.57, 82) | 0.016 (0.006, 0.03) | 0.019 (0.007, 0.037) | 19.22 (-20.38, 83.99) | 1.74 (0.68, 3.55) | 4.66 (1.68, 10.54) | 167.99 (67.68, 324.61) | 0.4 (0.16, 0.78) | 0.5 (0.2, 1.04) | 24.03 (-19.05, 90.97) | -5.1 (-23.03, 17) |
| Guyana | 0.36 (0.17, 0.61) | 1.72 (0.79, 3.05) | 377.27 (192.25, 646.53) | 0.046 (0.022, 0.078) | 0.225 (0.104, 0.399) | 386.58 (197.96, 661.09) | 0.094 (0.045, 0.152) | 0.266 (0.121, 0.465) | 183.08 (76.73, 328.35) | 13.02 (5.95, 23.05) | 58.94 (26.57, 103.33) | 352.71 (169.66, 614.73) | 2.82 (1.31, 4.78) | 8.24 (3.71, 14.26) | 191.81 (77.95, 358.82) | 4.6 (-7.48, 18.26) |
| Haiti | 1.09 (0.46, 2.17) | 2.68 (0.97, 6.27) | 145.63 (33.15, 311.97) | 0.017 (0.007, 0.034) | 0.021 (0.008, 0.049) | 21.84 (-33.96, 104.34) | 0.035 (0.015, 0.072) | 0.036 (0.013, 0.085) | 2.1 (-42.78, 67.59) | 38.47 (15.89, 75.03) | 101.57 (36.87, 234.75) | 164.03 (47.67, 342.94) | 1.04 (0.45, 2.04) | 1.11 (0.41, 2.51) | 7.04 (-37.55, 71.45) | 1.04 (-6.6, 9.3) |
| Honduras | 0.51 (0.21, 1.03) | 4.2 (1.66, 8.51) | 720.79 (380.21, 1206.35) | 0.011 (0.004, 0.022) | 0.042 (0.016, 0.084) | 282.39 (123.72, 508.6) | 0.026 (0.011, 0.052) | 0.069 (0.028, 0.136) | 167.45 (55.01, 322.74) | 24.08 (10, 47.76) | 154.86 (63.13, 313.1) | 543.09 (299.95, 913.64) | 1.01 (0.44, 1.99) | 2.19 (0.92, 4.43) | 116.64 (39.51, 231.29) | 2.98 (-5.81, 12.59) |
| Hungary | 5.49 (2.14, 10.22) | 12.06 (4.89, 21.31) | 119.6 (54.48, 241.79) | 0.053 (0.021, 0.098) | 0.126 (0.051, 0.222) | 137.85 (67.31, 270.2) | 0.041 (0.016, 0.076) | 0.059 (0.024, 0.103) | 42.37 (2.28, 111.91) | 228.13 (91.84, 431.87) | 323.15 (141.21, 580.57) | 41.65 (8.95, 95.92) | 1.73 (0.68, 3.32) | 1.91 (0.81, 3.5) | 10.47 (-15.53, 48.83) | 2.04 (-3.7, 8.13) |
| Iceland | 0.08 (0.03, 0.17) | 0.24 (0.09, 0.45) | 192.19 (90.75, 372.69) | 0.032 (0.013, 0.066) | 0.068 (0.027, 0.13) | 111.72 (38.22, 242.52) | 0.026 (0.01, 0.054) | 0.034 (0.014, 0.065) | 27.78 (-16.29, 108.73) | 3.24 (1.43, 6.18) | 6.34 (2.72, 11.99) | 95.79 (40.42, 174.88) | 1.11 (0.49, 2.14) | 1.12 (0.47, 2.09) | 0.62 (-27.03, 39.52) | -1.3 (-12.1, 10.83) |
| India | 75.28 (34.06, 137.06) | 422.63 (172.5, 741.93) | 461.44 (319.25, 614.82) | 0.009 (0.004, 0.016) | 0.03 (0.012, 0.052) | 238.6 (152.85, 331.1) | 0.017 (0.008, 0.031) | 0.037 (0.015, 0.065) | 112.58 (60.71, 169.86) | 3166.78 (1376.84, 6128.52) | 16412.64 (6655.36, 29395.29) | 418.27 (306.6, 533.83) | 0.6 (0.27, 1.13) | 1.28 (0.53, 2.29) | 113.79 (67.33, 159.45) | 2.63 (1.79, 3.48) |
| Indonesia | 5.4 (2.36, 9.54) | 26.89 (12.2, 48.53) | 397.69 (248.45, 582.96) | 0.003 (0.001, 0.005) | 0.01 (0.004, 0.017) | 230.08 (131.1, 352.96) | 0.006 (0.003, 0.011) | 0.013 (0.006, 0.023) | 101.39 (42.41, 176.28) | 185.52 (78.95, 330.18) | 905.52 (405.01, 1623.59) | 388.09 (256.65, 534.87) | 0.18 (0.08, 0.32) | 0.35 (0.16, 0.64) | 95.18 (43.4, 155.37) | 2.99 (-0.5, 6.59) |
| Iran (Islamic Republic of) | 8.02 (3.09, 16) | 31.81 (13.78, 62.16) | 296.46 (128.77, 531.83) | 0.014 (0.005, 0.028) | 0.037 (0.016, 0.073) | 165.22 (53.04, 322.67) | 0.039 (0.015, 0.076) | 0.045 (0.02, 0.087) | 15.17 (-37.25, 80.37) | 268.97 (104.82, 521.25) | 973.75 (409.42, 1908.16) | 262.03 (120.43, 436.37) | 1 (0.39, 1.92) | 1.18 (0.51, 2.29) | 17.23 (-27.62, 69.34) | 0.27 (-2.54, 3.17) |
| Iraq | 5.57 (2.31, 10.26) | 11.23 (4.13, 22.35) | 101.77 (3.02, 241.64) | 0.03 (0.013, 0.056) | 0.027 (0.01, 0.054) | -9.86 (-53.98, 52.63) | 0.068 (0.028, 0.125) | 0.05 (0.018, 0.096) | -26.7 (-59.77, 23.06) | 188.02 (80.73, 351.78) | 388.25 (135.32, 756.41) | 106.49 (13.34, 241.22) | 2.01 (0.85, 3.73) | 1.35 (0.5, 2.63) | -32.99 (-62.88, 8.74) | 0.74 (-3.61, 5.28) |
| Ireland | 2.44 (1.08, 4.75) | 6.75 (2.83, 11.8) | 176.46 (93.33, 306.3) | 0.068 (0.03, 0.132) | 0.137 (0.057, 0.239) | 101.53 (40.93, 196.18) | 0.068 (0.031, 0.127) | 0.078 (0.033, 0.135) | 14.49 (-19.53, 65.13) | 84.73 (36.6, 159.79) | 209.16 (94.26, 385.84) | 146.86 (92.48, 212.12) | 2.2 (0.95, 4.18) | 2.79 (1.23, 5.19) | 26.89 (0.73, 58.81) | -1.19 (-12, 10.94) |
| Israel | 6.31 (2.62, 11.7) | 19.19 (7.79, 36.73) | 204.25 (106.75, 365.72) | 0.127 (0.053, 0.236) | 0.2 (0.081, 0.383) | 57.33 (6.91, 140.82) | 0.154 (0.065, 0.284) | 0.132 (0.056, 0.25) | -14.35 (-39.65, 28.35) | 143.1 (63.08, 261.95) | 376.45 (160.95, 700.19) | 163.08 (102.39, 254.67) | 3.17 (1.38, 5.77) | 3.02 (1.3, 5.69) | -4.64 (-24.24, 23.53) | 0.67 (-7.46, 9.52) |
| Italy | 32.32 (13.33, 62.04) | 88.77 (37.2, 165.67) | 174.66 (69.46, 319.14) | 0.057 (0.023, 0.109) | 0.148 (0.062, 0.277) | 160.82 (60.92, 298.03) | 0.038 (0.016, 0.073) | 0.042 (0.018, 0.079) | 10 (-27.25, 60.95) | 876.48 (364.22, 1668.46) | 1576.92 (684.19, 2922.31) | 79.91 (29.94, 146.66) | 1.04 (0.43, 2.02) | 1.02 (0.44, 1.92) | -2.15 (-26.22, 26.53) | -1.3 (-7.11, 4.86) |
| Jamaica | 1.71 (0.77, 2.91) | 3.92 (1.8, 7.1) | 129.79 (48.32, 244.88) | 0.072 (0.033, 0.123) | 0.14 (0.064, 0.253) | 94.14 (25.31, 191.38) | 0.094 (0.043, 0.162) | 0.122 (0.056, 0.22) | 29.24 (-16.5, 92.94) | 42.35 (19.25, 72.88) | 111.54 (48.61, 210.92) | 163.37 (70.18, 290.76) | 2.42 (1.11, 4.12) | 3.57 (1.55, 6.78) | 47.75 (-5.04, 119.63) | 0.31 (-6.51, 7.62) |
| Japan | 100.76 (44.48, 183.82) | 346.85 (152.67, 707.26) | 244.23 (133.04, 393.75) | 0.08 (0.035, 0.146) | 0.272 (0.12, 0.554) | 239.19 (129.63, 386.52) | 0.068 (0.03, 0.123) | 0.06 (0.027, 0.114) | -11.47 (-36.21, 21.98) | 2376.38 (1047.9, 4220.12) | 5689.69 (2618.22, 10324.09) | 139.43 (77.69, 221.03) | 1.48 (0.66, 2.6) | 1.42 (0.66, 2.43) | -4.25 (-23.75, 20.62) | -0.96 (-3.66, 1.82) |
| Jordan | 0.62 (0.26, 1.17) | 6.83 (2.78, 12.67) | 997.75 (515.89, 1786.82) | 0.017 (0.007, 0.031) | 0.055 (0.023, 0.103) | 232.74 (86.68, 471.92) | 0.051 (0.021, 0.096) | 0.107 (0.043, 0.206) | 108.15 (13.05, 258.53) | 20.7 (8.52, 40.22) | 218.87 (89.85, 399.79) | 957.48 (523.89, 1638.91) | 1.33 (0.55, 2.51) | 2.58 (1.06, 4.83) | 93.45 (13.04, 225.74) | 2.43 (-5.63, 11.18) |
| Kazakhstan | 2.29 (0.86, 4.76) | 7.19 (2.57, 13.56) | 214.02 (102.9, 389.99) | 0.014 (0.005, 0.029) | 0.038 (0.014, 0.072) | 171.57 (75.47, 323.75) | 0.017 (0.006, 0.034) | 0.043 (0.016, 0.08) | 150.34 (65.91, 275.96) | 185.29 (74.88, 382.62) | 369.51 (136.49, 720.99) | 99.42 (36.71, 189.44) | 1.34 (0.53, 2.64) | 1.95 (0.74, 3.75) | 45.76 (1.09, 109.56) | 2.33 (-2.31, 7.2) |
| Kenya | 1.67 (0.67, 3.51) | 11.31 (4.29, 22.1) | 576.65 (308.23, 963.29) | 0.007 (0.003, 0.015) | 0.023 (0.009, 0.044) | 212.86 (88.75, 391.63) | 0.024 (0.01, 0.049) | 0.06 (0.023, 0.116) | 147.75 (47.86, 300.12) | 45.07 (17.92, 96.07) | 322.81 (125.5, 636.18) | 616.25 (333.13, 991.09) | 0.54 (0.22, 1.15) | 1.34 (0.52, 2.65) | 147.68 (50.89, 276.73) | 3.8 (-2.33, 10.31) |
| Kiribati | 0.03 (0.01, 0.05) | 0.09 (0.03, 0.17) | 256.11 (72.09, 575.65) | 0.034 (0.014, 0.061) | 0.074 (0.028, 0.141) | 118.69 (5.68, 314.93) | 0.076 (0.032, 0.138) | 0.144 (0.057, 0.286) | 89.89 (-5.04, 249.48) | 0.85 (0.35, 1.54) | 2.91 (1.09, 5.76) | 243.79 (76.74, 531) | 2.08 (0.87, 3.81) | 3.68 (1.43, 7.03) | 77.17 (-10.33, 212.98) | -8.37 (-22.01, 7.66) |
| Kuwait | 0.44 (0.19, 0.84) | 2.42 (0.99, 4.45) | 444.3 (244.29, 778.31) | 0.026 (0.011, 0.049) | 0.052 (0.021, 0.096) | 101.16 (27.24, 224.6) | 0.067 (0.029, 0.119) | 0.093 (0.037, 0.177) | 38.3 (-19.95, 117.92) | 19.01 (7.49, 38.97) | 92.41 (37.53, 175.91) | 386.13 (218.4, 686.33) | 1.89 (0.8, 3.5) | 2.37 (0.98, 4.3) | 25.57 (-18.02, 95.79) | -0.45 (-10.26, 10.44) |
| Kyrgyzstan | 0.27 (0.09, 0.68) | 1.21 (0.45, 2.6) | 340.74 (168.82, 653.14) | 0.006 (0.002, 0.015) | 0.018 (0.007, 0.038) | 186.67 (74.85, 389.86) | 0.008 (0.003, 0.019) | 0.024 (0.009, 0.048) | 181.34 (77.52, 353.29) | 28.39 (10.19, 65.05) | 70.59 (26.58, 153.47) | 148.65 (62.48, 300.37) | 0.85 (0.32, 1.85) | 1.23 (0.48, 2.54) | 44.78 (-2.25, 124.96) | 1.59 (-9.06, 13.49) |
| Lao People's Democratic Republic | 0.52 (0.22, 0.99) | 1.78 (0.72, 3.38) | 242.05 (111.79, 468.74) | 0.013 (0.005, 0.024) | 0.024 (0.01, 0.046) | 93.32 (19.69, 221.43) | 0.027 (0.012, 0.051) | 0.042 (0.017, 0.077) | 53.48 (-7.09, 139.47) | 16.38 (6.8, 31.33) | 56.5 (23.2, 105.56) | 244.99 (116.77, 461.53) | 0.74 (0.31, 1.4) | 1.11 (0.45, 2.05) | 49.42 (-5.39, 138.44) | 0.46 (-9.94, 12.07) |
| Latvia | 0.15 (0.05, 0.36) | 0.71 (0.26, 1.51) | 364.08 (176.25, 827.63) | 0.006 (0.002, 0.013) | 0.038 (0.014, 0.081) | 559.54 (292.61, 1218.34) | 0.005 (0.001, 0.011) | 0.017 (0.006, 0.036) | 265.55 (123.72, 571.15) | 14.96 (5.56, 31.62) | 23.91 (9.22, 48.51) | 59.88 (6.27, 152.22) | 0.45 (0.16, 0.98) | 0.69 (0.26, 1.48) | 54.81 (2.81, 139.72) | 3.85 (-14.18, 25.67) |
| Lebanon | 1.27 (0.47, 2.53) | 4.55 (1.87, 8.47) | 256.64 (116.19, 544.02) | 0.043 (0.016, 0.085) | 0.082 (0.034, 0.153) | 92.6 (16.75, 247.79) | 0.065 (0.025, 0.127) | 0.071 (0.029, 0.13) | 8.72 (-34.14, 94.92) | 38.77 (14.86, 77.59) | 104.66 (44.77, 187.68) | 169.95 (62.22, 348.04) | 1.71 (0.65, 3.36) | 1.69 (0.71, 3) | -1.31 (-39.16, 62.69) | -0.66 (-8.43, 7.77) |
| Lesotho | 0.18 (0.07, 0.37) | 0.93 (0.32, 2.09) | 409.6 (183.77, 727.15) | 0.012 (0.004, 0.024) | 0.05 (0.017, 0.111) | 316.67 (132.02, 576.31) | 0.024 (0.009, 0.05) | 0.095 (0.033, 0.205) | 286.84 (117.61, 504.74) | 7.14 (2.85, 14.13) | 33.65 (11.63, 76.15) | 371.15 (190.97, 643.82) | 0.83 (0.33, 1.63) | 2.84 (1.05, 6.22) | 242.48 (115.3, 435.16) | 1.75 (-11.09, 16.45) |
| Liberia | 0.24 (0.09, 0.5) | 0.56 (0.2, 1.11) | 131.45 (40.09, 261.79) | 0.01 (0.004, 0.02) | 0.01 (0.004, 0.02) | 4.32 (-36.86, 63.06) | 0.024 (0.01, 0.045) | 0.026 (0.01, 0.048) | 9.65 (-32.41, 67.79) | 8 (3.02, 17.88) | 22.57 (7.8, 46.07) | 182.24 (67.77, 348.02) | 0.62 (0.25, 1.29) | 0.74 (0.28, 1.44) | 19.38 (-23.5, 82.88) | 0.56 (-16.28, 20.78) |
| Libya | 1.27 (0.51, 2.6) | 3.89 (1.36, 7.52) | 205.99 (42.36, 462.3) | 0.03 (0.012, 0.062) | 0.057 (0.02, 0.109) | 87.72 (-12.66, 244.97) | 0.069 (0.028, 0.139) | 0.075 (0.027, 0.145) | 8.23 (-49.54, 99.49) | 40.55 (15.07, 83.57) | 130.06 (41.82, 261.41) | 220.75 (59.18, 476.38) | 1.89 (0.74, 3.81) | 2.03 (0.69, 3.97) | 7.46 (-44.97, 89.11) | 1.56 (-5.26, 8.87) |
| Lithuania | 0.21 (0.07, 0.52) | 1.39 (0.53, 2.76) | 550.35 (303.64, 1093.93) | 0.006 (0.002, 0.014) | 0.051 (0.019, 0.101) | 775.86 (443.6, 1507.92) | 0.005 (0.002, 0.012) | 0.024 (0.009, 0.05) | 373.8 (207.17, 693.44) | 25.09 (9.17, 53.21) | 58.16 (23, 114.4) | 131.84 (55.05, 253.54) | 0.59 (0.21, 1.27) | 1.21 (0.44, 2.48) | 107.03 (37.95, 206.4) | 2.76 (-11.24, 18.98) |
| Luxembourg | 0.4 (0.15, 0.72) | 0.63 (0.27, 1.19) | 58.46 (-4.01, 161.25) | 0.104 (0.039, 0.19) | 0.097 (0.042, 0.184) | -6.25 (-43.21, 54.57) | 0.077 (0.03, 0.146) | 0.051 (0.022, 0.095) | -33.66 (-57.18, 7.14) | 12.35 (5.07, 22.36) | 16.57 (7.4, 31.21) | 34.13 (-3.07, 92.25) | 2.37 (0.99, 4.3) | 1.63 (0.72, 3.16) | -30.99 (-48.92, -6.19) | -2.8 (-14.99, 11.14) |
| Madagascar | 1.2 (0.46, 2.32) | 2.38 (0.9, 4.67) | 98.72 (18.51, 222.64) | 0.01 (0.004, 0.019) | 0.008 (0.003, 0.016) | -17.19 (-50.61, 34.45) | 0.028 (0.011, 0.056) | 0.028 (0.011, 0.054) | -0.65 (-40.38, 63.38) | 32.97 (12.54, 63.43) | 72.31 (28.53, 144.32) | 119.28 (29.91, 256.65) | 0.65 (0.25, 1.22) | 0.62 (0.24, 1.21) | -3.48 (-40.64, 51.59) | 0.6 (-8.91, 11.1) |
| Malawi | 0.88 (0.36, 1.66) | 3.79 (1.44, 7.46) | 329.38 (164.3, 552.43) | 0.009 (0.004, 0.017) | 0.019 (0.007, 0.038) | 116.5 (33.26, 228.96) | 0.028 (0.012, 0.054) | 0.062 (0.025, 0.12) | 118.8 (36.05, 235.56) | 24.42 (10.5, 45.37) | 106.74 (41.5, 210.14) | 337.04 (171.09, 566.83) | 0.63 (0.27, 1.17) | 1.39 (0.54, 2.72) | 118.8 (35.77, 225.91) | 2.53 (-6.06, 11.92) |
| Malaysia | 3.69 (1.74, 6.63) | 23.69 (10.42, 40.88) | 541.67 (342.89, 788.72) | 0.021 (0.01, 0.038) | 0.074 (0.033, 0.128) | 256.35 (145.96, 393.55) | 0.042 (0.02, 0.074) | 0.088 (0.039, 0.152) | 107.76 (41.15, 190.37) | 115.12 (50.19, 213.59) | 742.98 (330.09, 1338.6) | 545.42 (347.84, 784.57) | 1.18 (0.51, 2.14) | 2.49 (1.12, 4.42) | 111.71 (45.73, 191.7) | 2.87 (-1.25, 7.17) |
| Maldives | 0.03 (0.01, 0.05) | 0.08 (0.03, 0.13) | 188.87 (75.08, 354.32) | 0.012 (0.005, 0.022) | 0.015 (0.006, 0.026) | 24.13 (-24.76, 95.23) | 0.034 (0.014, 0.063) | 0.024 (0.01, 0.044) | -27.19 (-53.8, 14.38) | 0.83 (0.35, 1.55) | 2.28 (0.96, 4.17) | 174.27 (73, 327.03) | 0.86 (0.37, 1.61) | 0.59 (0.24, 1.04) | -31.69 (-56.71, 0.72) | -7.61 (-19.44, 5.95) |
| Mali | 0.46 (0.18, 0.95) | 2.4 (0.94, 4.84) | 428.15 (244.87, 703.86) | 0.005 (0.002, 0.011) | 0.01 (0.004, 0.02) | 89.81 (23.94, 188.9) | 0.015 (0.006, 0.029) | 0.032 (0.013, 0.064) | 120.57 (39.45, 228.6) | 14.54 (5.84, 30.3) | 84.57 (32.15, 178.61) | 481.49 (270.54, 814.3) | 0.36 (0.15, 0.75) | 0.85 (0.34, 1.69) | 134 (60.56, 241.21) | 3.37 (-7.48, 15.48) |
| Malta | 0.4 (0.16, 0.72) | 0.96 (0.42, 1.76) | 141.99 (54.87, 260.63) | 0.107 (0.043, 0.194) | 0.217 (0.095, 0.397) | 102.79 (29.79, 202.22) | 0.104 (0.041, 0.192) | 0.085 (0.037, 0.159) | -18.1 (-46.76, 17.88) | 12.02 (5.06, 22.65) | 23.3 (10.14, 41.7) | 93.81 (41.08, 168.78) | 2.92 (1.24, 5.45) | 2.66 (1.1, 5.02) | -8.85 (-32.73, 21.28) | -1.98 (-13.04, 10.48) |
| Marshall Islands | 0.01 (0, 0.02) | 0.06 (0.01, 0.16) | 521.26 (137.16, 1056.23) | 0.02 (0.007, 0.042) | 0.101 (0.022, 0.29) | 401.52 (91.45, 833.39) | 0.061 (0.023, 0.126) | 0.189 (0.042, 0.517) | 209.8 (16.53, 497.91) | 0.31 (0.11, 0.67) | 1.79 (0.42, 5.06) | 476.89 (138.58, 1007.4) | 1.68 (0.61, 3.37) | 4.67 (1.14, 12.91) | 178.69 (13.4, 427.54) | -3.62 (-13.65, 7.58) |
| Mauritania | 0.22 (0.09, 0.44) | 1.07 (0.4, 2.1) | 378.24 (182.72, 696.7) | 0.011 (0.005, 0.021) | 0.024 (0.009, 0.048) | 123.51 (32.13, 272.35) | 0.027 (0.011, 0.053) | 0.058 (0.022, 0.116) | 118.17 (31.04, 258.89) | 6.23 (2.48, 12.23) | 29.97 (10.96, 60.74) | 381.38 (198.76, 683.83) | 0.63 (0.26, 1.23) | 1.35 (0.51, 2.72) | 115.64 (36, 247.98) | -1.13 (-14.54, 14.38) |
| Mauritius | 0.69 (0.29, 1.22) | 7.76 (3.41, 14.03) | 1024.57 (603.94, 1672.7) | 0.063 (0.027, 0.111) | 0.61 (0.268, 1.103) | 868.95 (506.53, 1427.39) | 0.103 (0.045, 0.184) | 0.441 (0.193, 0.795) | 326.57 (165.83, 564.58) | 20.88 (8.45, 37.36) | 188.98 (80.86, 343.25) | 804.87 (486.24, 1303.69) | 2.71 (1.09, 4.8) | 10.47 (4.5, 19.1) | 285.95 (156.67, 492.25) | 4.89 (-3.61, 14.13) |
| Mexico | 110.94 (46.85, 204.71) | 612.68 (257.03, 1122.68) | 452.29 (300.33, 638.83) | 0.13 (0.055, 0.24) | 0.474 (0.199, 0.868) | 264.8 (164.43, 388.02) | 0.286 (0.13, 0.513) | 0.483 (0.206, 0.877) | 68.72 (21.52, 128.66) | 4029.16 (1685.11, 7631.69) | 20650.41 (8549.55, 38361.52) | 412.52 (272.98, 596.61) | 8.16 (3.62, 14.9) | 15.43 (6.46, 28.67) | 88.99 (38.88, 155.53) | 2.39 (1.72, 3.06) |
| Micronesia (Federated States of) | 0.04 (0.02, 0.08) | 0.11 (0.05, 0.21) | 163.81 (23.27, 417.25) | 0.039 (0.016, 0.078) | 0.104 (0.045, 0.202) | 166.13 (24.35, 421.81) | 0.093 (0.039, 0.187) | 0.17 (0.074, 0.322) | 82.11 (-14.52, 262.21) | 1.24 (0.54, 2.3) | 3.3 (1.43, 6.29) | 167.03 (32.84, 414.69) | 2.42 (1.04, 4.54) | 4.24 (1.88, 8.05) | 75.22 (-10.7, 228.85) | -6.63 (-21.9, 11.62) |
| Monaco | 0.1 (0.04, 0.19) | 0.25 (0.11, 0.46) | 141.97 (56.59, 291.03) | 0.344 (0.135, 0.63) | 0.669 (0.281, 1.22) | 94.35 (25.78, 214.09) | 0.125 (0.05, 0.228) | 0.199 (0.085, 0.364) | 58.71 (5.88, 141.26) | 2.8 (1.21, 4.96) | 5.18 (2.19, 9.11) | 84.97 (33.62, 155.28) | 4.19 (1.86, 7.75) | 5.57 (2.33, 10.08) | 32.96 (-0.74, 75.11) | -0.92 (-11.35, 10.75) |
| Mongolia | 0.24 (0.09, 0.53) | 0.6 (0.22, 1.25) | 147.7 (49.92, 298.12) | 0.011 (0.004, 0.024) | 0.018 (0.007, 0.038) | 60.21 (-3.03, 157.49) | 0.023 (0.009, 0.047) | 0.026 (0.01, 0.055) | 14.99 (-28.13, 79.67) | 12.46 (4.63, 26.99) | 33.04 (12.69, 67.53) | 165.2 (71.37, 320.01) | 1.01 (0.41, 2.11) | 1.17 (0.46, 2.36) | 15.57 (-21.62, 75.69) | 0.84 (-14.54, 18.99) |
| Montenegro | 0.28 (0.1, 0.55) | 0.46 (0.18, 0.87) | 64.62 (-1.87, 172.21) | 0.045 (0.016, 0.089) | 0.075 (0.029, 0.14) | 66.78 (-0.58, 175.79) | 0.047 (0.017, 0.093) | 0.055 (0.021, 0.105) | 15.17 (-31.78, 92.39) | 9.38 (3.68, 19.16) | 12.68 (4.88, 24.16) | 35.26 (-10.81, 104.95) | 1.49 (0.6, 2.98) | 1.49 (0.58, 2.85) | -0.34 (-32, 47.27) | 0.73 (-15.38, 19.92) |
| Morocco | 2.92 (1.09, 6.25) | 18.33 (7.52, 33.76) | 527.88 (243.7, 897.11) | 0.012 (0.004, 0.025) | 0.049 (0.02, 0.091) | 328.25 (134.43, 580.1) | 0.022 (0.008, 0.051) | 0.058 (0.024, 0.109) | 161.9 (40.09, 317.26) | 88.98 (36.75, 173.68) | 534.6 (207.99, 996.17) | 500.79 (249.31, 812.79) | 0.58 (0.23, 1.17) | 1.5 (0.59, 2.79) | 157.39 (47.04, 284.98) | 3.9 (-0.64, 8.65) |
| Mozambique | 0.56 (0.23, 1.12) | 3.08 (1.29, 6.58) | 445.37 (223.56, 772.71) | 0.004 (0.002, 0.008) | 0.01 (0.004, 0.021) | 134.49 (39.12, 275.23) | 0.012 (0.005, 0.024) | 0.035 (0.015, 0.074) | 184.35 (70.44, 380.68) | 15.8 (6.49, 31.62) | 92.77 (37.2, 202.35) | 487.19 (249.6, 828.86) | 0.27 (0.11, 0.53) | 0.79 (0.33, 1.63) | 190.88 (76.43, 355.15) | 2.74 (-6.21, 12.54) |
| Myanmar | 2.54 (1.1, 4.37) | 11.99 (5.3, 21.8) | 371.24 (189.76, 670.44) | 0.006 (0.003, 0.011) | 0.021 (0.009, 0.039) | 237.76 (107.68, 452.21) | 0.012 (0.005, 0.021) | 0.027 (0.012, 0.049) | 123.1 (42.04, 267.68) | 85.3 (35.95, 147.55) | 382.61 (168.79, 701.82) | 348.56 (179.63, 617.96) | 0.35 (0.15, 0.6) | 0.75 (0.33, 1.37) | 117.31 (38.25, 244.64) | 3.11 (-1.71, 8.16) |
| Namibia | 0.12 (0.05, 0.25) | 0.33 (0.13, 0.68) | 165.88 (56.13, 338.82) | 0.009 (0.003, 0.018) | 0.014 (0.005, 0.028) | 53.55 (-9.83, 153.43) | 0.022 (0.009, 0.044) | 0.026 (0.011, 0.053) | 19.67 (-28.38, 93.31) | 5.17 (1.96, 10.86) | 13.13 (5.07, 27.13) | 154.06 (57.89, 299.56) | 0.72 (0.29, 1.5) | 0.82 (0.32, 1.69) | 13.2 (-25.82, 73.12) | -3.43 (-20.25, 16.93) |
| Nauru | 0.01 (0, 0.02) | 0.02 (0.01, 0.03) | 101.03 (3.86, 289.38) | 0.077 (0.03, 0.152) | 0.144 (0.061, 0.268) | 86.09 (-3.86, 260.44) | 0.188 (0.072, 0.354) | 0.301 (0.125, 0.562) | 60.33 (-15.02, 197.27) | 0.27 (0.1, 0.54) | 0.49 (0.2, 0.92) | 78.98 (-6.81, 240) | 5.12 (2.03, 10.03) | 7.68 (3.26, 14.23) | 50.14 (-18.98, 178.66) | -0.58 (-8.73, 8.3) |
| Nepal | 0.43 (0.18, 0.85) | 2.57 (1.04, 4.89) | 494.68 (237.5, 875.19) | 0.002 (0.001, 0.004) | 0.008 (0.003, 0.016) | 271.92 (111.08, 509.9) | 0.005 (0.002, 0.01) | 0.012 (0.005, 0.023) | 127.61 (26.95, 276.52) | 17.86 (7.27, 35.52) | 96.78 (37.47, 186.29) | 441.86 (261.77, 713.09) | 0.18 (0.07, 0.34) | 0.39 (0.16, 0.74) | 123.09 (46.9, 238.35) | 3.4 (-7.8, 15.96) |
| Netherlands | 8.1 (3.56, 15.21) | 31.26 (12.83, 58.16) | 285.71 (167.73, 490.66) | 0.054 (0.024, 0.102) | 0.182 (0.075, 0.338) | 234.42 (132.13, 412.11) | 0.041 (0.018, 0.076) | 0.076 (0.032, 0.143) | 87.03 (31.6, 182.2) | 261.45 (110.93, 496.15) | 664.29 (288.93, 1200.73) | 154.08 (99.12, 246.85) | 1.35 (0.57, 2.6) | 1.98 (0.87, 3.68) | 46.59 (17.41, 97.68) | 2.06 (-6.17, 11.01) |
| New Zealand | 1.35 (0.56, 2.61) | 4.75 (1.96, 8.52) | 251.24 (163.48, 404.77) | 0.04 (0.016, 0.076) | 0.092 (0.038, 0.165) | 132.18 (74.17, 233.68) | 0.037 (0.015, 0.07) | 0.054 (0.022, 0.098) | 46.15 (8.72, 102.72) | 41.16 (17.63, 78.6) | 128.18 (54.44, 238.08) | 211.4 (138.84, 303.43) | 1.1 (0.47, 2.07) | 1.65 (0.69, 3.11) | 49.22 (14.67, 93.1) | 2.68 (-7.6, 14.1) |
| Nicaragua | 0.55 (0.22, 1.07) | 6.42 (2.67, 12.06) | 1073.97 (619.56, 1713.14) | 0.014 (0.006, 0.028) | 0.096 (0.04, 0.181) | 584.34 (319.45, 956.93) | 0.037 (0.015, 0.069) | 0.131 (0.055, 0.241) | 257.47 (122.55, 444.62) | 20.77 (8.15, 40.32) | 220.31 (91.13, 446.49) | 960.67 (539.31, 1519.36) | 1.16 (0.47, 2.3) | 4.03 (1.68, 7.78) | 247.37 (114.8, 429.72) | 6.03 (-2.49, 15.28) |
| Niger | 0.3 (0.12, 0.66) | 1.5 (0.54, 3.45) | 394.33 (219.04, 714.46) | 0.004 (0.001, 0.008) | 0.006 (0.002, 0.014) | 58.58 (2.35, 161.28) | 0.012 (0.005, 0.024) | 0.019 (0.007, 0.042) | 62.93 (8.63, 156.06) | 12.77 (5.04, 28.16) | 64.57 (24, 144.32) | 405.81 (227.28, 732.23) | 0.36 (0.14, 0.74) | 0.6 (0.24, 1.31) | 66.2 (15.61, 152.09) | 2.05 (-10.21, 15.99) |
| Nigeria | 4.38 (1.85, 8.63) | 23.15 (8.19, 45.93) | 428.49 (224.34, 703.73) | 0.005 (0.002, 0.01) | 0.01 (0.004, 0.02) | 105.85 (26.33, 213.06) | 0.012 (0.005, 0.023) | 0.029 (0.011, 0.054) | 148.27 (54.18, 284.56) | 155.81 (65.75, 309.61) | 963.36 (340.45, 1973.12) | 518.29 (294.71, 837.65) | 0.34 (0.14, 0.64) | 0.88 (0.34, 1.7) | 161.66 (77.27, 282.19) | 2.84 (-0.66, 6.46) |
| Niue | 0 (0, 0) | 0.01 (0, 0.02) | 213.69 (42.52, 481.44) | 0.111 (0.046, 0.21) | 0.482 (0.18, 0.97) | 332.99 (96.73, 702.56) | 0.115 (0.048, 0.212) | 0.387 (0.143, 0.778) | 237.34 (58.31, 535.97) | 0.07 (0.03, 0.12) | 0.19 (0.07, 0.39) | 191.05 (41.46, 434.7) | 3.11 (1.26, 5.7) | 9.06 (3.26, 18.42) | 191.88 (41.57, 435.24) | 0.35 (-7.87, 9.31) |
| North Macedonia | 0.53 (0.2, 1.02) | 1.53 (0.61, 3) | 187.4 (77.76, 378.02) | 0.027 (0.01, 0.051) | 0.07 (0.028, 0.138) | 163.09 (62.72, 337.58) | 0.03 (0.011, 0.058) | 0.055 (0.023, 0.104) | 80.55 (12.01, 194.85) | 24.61 (9.81, 47.07) | 58.59 (24.5, 111.72) | 138.05 (61.38, 269.99) | 1.27 (0.52, 2.43) | 1.94 (0.82, 3.76) | 52.54 (3.48, 131.4) | 1.95 (-9.98, 15.46) |
| Northern Mariana Islands | 0.08 (0.03, 0.13) | 0.23 (0.1, 0.43) | 208.95 (77.5, 423.9) | 0.167 (0.071, 0.294) | 0.481 (0.214, 0.891) | 187.48 (65.17, 387.49) | 0.542 (0.243, 0.937) | 0.536 (0.247, 0.923) | -1.14 (-39.25, 62.26) | 2.85 (1.13, 5.12) | 6.94 (3, 12.85) | 143.63 (47.04, 312.83) | 12.9 (5.48, 22.38) | 12.92 (5.96, 23.37) | 0.15 (-39.56, 61.11) | -0.68 (-20.17, 23.57) |
| Norway | 1.37 (0.59, 2.64) | 5.65 (2.25, 10.69) | 311.94 (189.2, 501.77) | 0.032 (0.014, 0.062) | 0.104 (0.042, 0.197) | 222.83 (126.65, 371.6) | 0.018 (0.008, 0.034) | 0.044 (0.018, 0.082) | 148.51 (76.8, 255.9) | 53 (24.44, 101.7) | 133.56 (60.17, 245.47) | 152 (97.24, 224.26) | 0.82 (0.37, 1.55) | 1.35 (0.6, 2.48) | 66.08 (34.57, 107.64) | -2.02 (-14.81, 12.68) |
| Oman | 0.15 (0.06, 0.3) | 1.59 (0.62, 3.13) | 971.6 (511.09, 1781.9) | 0.007 (0.003, 0.015) | 0.034 (0.013, 0.066) | 352.08 (157.81, 693.93) | 0.024 (0.01, 0.047) | 0.086 (0.036, 0.164) | 256.59 (101.04, 544.6) | 5.16 (2, 10.13) | 61.32 (23.94, 124.91) | 1089.44 (612.04, 1895.97) | 0.63 (0.25, 1.22) | 2.12 (0.85, 4.12) | 235.49 (105.92, 448.22) | 3.6 (-10.56, 20.01) |
| Pakistan | 4.99 (2.08, 9.41) | 16.57 (6.39, 32.77) | 231.97 (108.4, 414.62) | 0.004 (0.002, 0.008) | 0.007 (0.003, 0.014) | 56.62 (-1.68, 142.78) | 0.01 (0.004, 0.018) | 0.014 (0.006, 0.028) | 45.6 (-5.35, 117.73) | 158.55 (62.16, 311.04) | 624.47 (222.83, 1270.97) | 293.87 (149.23, 485.49) | 0.27 (0.11, 0.53) | 0.42 (0.16, 0.83) | 56.24 (1.5, 129.6) | 0.86 (-2.5, 4.33) |
| Palau | 0.01 (0.01, 0.02) | 0.06 (0.02, 0.12) | 408.58 (173.25, 839.88) | 0.083 (0.035, 0.146) | 0.353 (0.138, 0.653) | 326.94 (129.39, 689.01) | 0.147 (0.063, 0.255) | 0.354 (0.144, 0.648) | 141.19 (32.57, 340.66) | 0.41 (0.17, 0.73) | 1.82 (0.75, 3.3) | 342.65 (145.09, 715.66) | 3.94 (1.71, 7.05) | 8.35 (3.48, 14.71) | 112.04 (20.77, 279.3) | -2.94 (-12.88, 8.12) |
| Palestine | 0.17 (0.07, 0.32) | 0.43 (0.17, 0.8) | 144.45 (54.17, 297.29) | 0.009 (0.003, 0.016) | 0.008 (0.003, 0.016) | -2.57 (-38.55, 58.35) | 0.024 (0.009, 0.043) | 0.02 (0.009, 0.036) | -14.32 (-43.5, 36.9) | 4.77 (1.85, 8.83) | 13.57 (5.3, 27.24) | 184.62 (79.27, 340.66) | 0.54 (0.21, 0.97) | 0.47 (0.19, 0.89) | -11.99 (-43.22, 38.6) | -4.63 (-19.69, 13.25) |
| Panama | 0.43 (0.18, 0.84) | 3.84 (1.66, 6.93) | 790.11 (485.82, 1288.3) | 0.018 (0.008, 0.035) | 0.09 (0.039, 0.161) | 395.44 (226.07, 672.74) | 0.03 (0.013, 0.057) | 0.085 (0.036, 0.155) | 186.23 (86.94, 345.22) | 16.65 (6.89, 33.5) | 115.73 (46.36, 216.78) | 595.3 (361.88, 970.89) | 1.02 (0.43, 1.98) | 2.61 (1.03, 4.87) | 154.75 (65.57, 286.17) | 4.15 (-6.64, 16.19) |
| Papua New Guinea | 0.29 (0.12, 0.55) | 0.81 (0.36, 1.5) | 181.48 (49.44, 420.47) | 0.007 (0.003, 0.013) | 0.008 (0.003, 0.014) | 10.4 (-41.39, 104.13) | 0.017 (0.008, 0.032) | 0.017 (0.007, 0.031) | -2.72 (-46.3, 70.23) | 11.35 (4.45, 21.59) | 31.7 (13.49, 58.39) | 179.22 (57.66, 394.35) | 0.54 (0.23, 0.98) | 0.51 (0.22, 0.94) | -5.61 (-45.45, 62.72) | -0.55 (-13.86, 14.82) |
| Paraguay | 1.34 (0.62, 2.43) | 4.58 (1.78, 8.01) | 240.63 (111.06, 442) | 0.033 (0.015, 0.06) | 0.064 (0.025, 0.112) | 92.09 (19.02, 205.64) | 0.063 (0.029, 0.112) | 0.079 (0.031, 0.141) | 25.59 (-22.78, 102.09) | 41.77 (18.12, 74.31) | 144.06 (56.89, 256.16) | 244.86 (125.58, 435.28) | 1.75 (0.76, 3.09) | 2.29 (0.92, 3.9) | 31.22 (-14.67, 101.37) | 0.98 (-5.93, 8.41) |
| Peru | 7.88 (3.35, 13.85) | 52.65 (23.29, 92.16) | 567.87 (341.08, 894.17) | 0.036 (0.015, 0.064) | 0.145 (0.064, 0.254) | 298.36 (163.09, 492.98) | 0.071 (0.031, 0.126) | 0.156 (0.069, 0.272) | 120.39 (44.17, 224.27) | 199.53 (84.31, 363.62) | 1310.94 (549.05, 2370.67) | 557.01 (340.31, 861.84) | 1.62 (0.69, 2.89) | 3.82 (1.61, 6.89) | 134.96 (55.99, 242.94) | 3.91 (0.65, 7.27) |
| Philippines | 10.43 (4.75, 18.45) | 79.46 (33.38, 139.63) | 662.21 (466.92, 914.69) | 0.017 (0.008, 0.029) | 0.07 (0.029, 0.123) | 324.07 (215.42, 464.54) | 0.043 (0.02, 0.076) | 0.102 (0.043, 0.182) | 135.75 (71.64, 213.93) | 346.19 (153.11, 610.94) | 2493.61 (1063.97, 4255.27) | 620.31 (443.5, 855.74) | 1.09 (0.49, 1.87) | 2.78 (1.19, 4.79) | 154.19 (92.95, 233.12) | 4.13 (1.88, 6.43) |
| Poland | 25.9 (10.6, 48.46) | 34.31 (14.94, 62.25) | 32.47 (-4.58, 89.93) | 0.068 (0.028, 0.127) | 0.09 (0.039, 0.163) | 32.25 (-4.74, 89.6) | 0.063 (0.026, 0.116) | 0.047 (0.02, 0.085) | -26.2 (-46, 2.28) | 856.47 (335.14, 1608.5) | 1164.28 (511.11, 2131.45) | 35.94 (3.81, 87.19) | 2.03 (0.81, 3.79) | 1.85 (0.8, 3.44) | -9.07 (-29.44, 23.24) | -1.42 (-4.15, 1.4) |
| Portugal | 7.69 (3.36, 13.9) | 24.53 (10.05, 44.8) | 219.1 (89.46, 423.19) | 0.076 (0.033, 0.137) | 0.231 (0.095, 0.422) | 204.94 (81.05, 399.97) | 0.065 (0.029, 0.124) | 0.075 (0.032, 0.138) | 15.87 (-26.84, 86.06) | 201.14 (88.68, 365.71) | 424.36 (181.62, 754.43) | 110.98 (47.17, 193.99) | 1.6 (0.71, 2.99) | 1.74 (0.74, 3.12) | 8.78 (-17.24, 42.68) | -0.45 (-7.43, 7.05) |
| Puerto Rico | 5.17 (2.37, 9.49) | 13.23 (5.75, 23.62) | 156.09 (53.82, 323.4) | 0.143 (0.066, 0.263) | 0.402 (0.174, 0.717) | 180.81 (68.66, 364.26) | 0.151 (0.07, 0.272) | 0.188 (0.081, 0.323) | 24.73 (-22.94, 98.43) | 156.67 (70.68, 281.33) | 310.39 (134.43, 530) | 98.11 (26.05, 215.52) | 4.41 (2, 7.99) | 5.77 (2.54, 10.15) | 30.8 (-15.18, 102.33) | 2.58 (-1.94, 7.31) |
| Qatar | 0.17 (0.06, 0.34) | 2.82 (1.11, 4.89) | 1538.8 (806.66, 3271.2) | 0.039 (0.014, 0.077) | 0.095 (0.037, 0.164) | 144.87 (35.47, 403.73) | 0.188 (0.071, 0.371) | 0.502 (0.209, 0.895) | 167.55 (44.89, 431.65) | 8.46 (3.14, 16.86) | 121.07 (48.8, 214.53) | 1331.93 (762.07, 2558.85) | 4.46 (1.76, 8.29) | 10.21 (4.36, 17.66) | 128.89 (31.51, 336.92) | 2.06 (-11.13, 17.22) |
| Republic of Korea | 5.98 (2.58, 10.25) | 25.81 (11.61, 48.02) | 331.85 (177.97, 587.93) | 0.014 (0.006, 0.023) | 0.05 (0.023, 0.093) | 270.51 (138.49, 490.21) | 0.028 (0.012, 0.049) | 0.028 (0.013, 0.053) | 1.09 (-34.65, 60.52) | 161.11 (70.74, 279.4) | 555.66 (245.6, 981.03) | 244.89 (141.91, 400.41) | 0.58 (0.25, 0.99) | 0.61 (0.27, 1.1) | 6.27 (-24.81, 52.37) | 0.36 (-4.73, 5.72) |
| Republic of Moldova | 0.05 (0.02, 0.12) | 0.16 (0.05, 0.36) | 244.17 (111.35, 464.21) | 0.001 (0, 0.003) | 0.005 (0.001, 0.01) | 325.95 (161.57, 598.28) | 0.001 (0, 0.003) | 0.003 (0.001, 0.006) | 131.53 (42.6, 252.88) | 8.45 (3.1, 17.7) | 14.82 (5.49, 30.63) | 75.46 (15.17, 160.87) | 0.19 (0.07, 0.4) | 0.27 (0.1, 0.55) | 38.26 (-7.69, 96.06) | -2.45 (-19.29, 17.91) |
| Romania | 10.48 (3.42, 21.18) | 33.48 (14.13, 61.52) | 219.53 (113.75, 448.11) | 0.045 (0.015, 0.091) | 0.177 (0.075, 0.325) | 294.46 (163.88, 576.65) | 0.043 (0.015, 0.088) | 0.091 (0.038, 0.169) | 110.72 (46.38, 247.26) | 448.84 (177.53, 868.42) | 1195.47 (512.95, 2116.97) | 166.34 (94.9, 295.15) | 1.74 (0.7, 3.37) | 3.82 (1.59, 6.86) | 119.25 (63.05, 215.53) | 4.21 (0.97, 7.55) |
| Russian Federation | 13.46 (4.9, 29) | 41.13 (16.27, 75.95) | 205.51 (120.4, 345.51) | 0.009 (0.003, 0.019) | 0.028 (0.011, 0.052) | 218.44 (129.73, 364.37) | 0.008 (0.003, 0.017) | 0.017 (0.007, 0.033) | 122.32 (64.54, 220.53) | 791.32 (304.43, 1678.86) | 1434.74 (579.03, 2789.26) | 81.31 (37.43, 146.54) | 0.46 (0.18, 0.96) | 0.64 (0.25, 1.28) | 40.22 (7, 88.35) | 1.61 (-0.94, 4.23) |
| Rwanda | 0.5 (0.21, 0.98) | 1.29 (0.52, 2.53) | 156.77 (47.98, 292.5) | 0.007 (0.003, 0.014) | 0.01 (0.004, 0.019) | 39.11 (-19.83, 112.65) | 0.022 (0.009, 0.042) | 0.026 (0.011, 0.051) | 19.67 (-28.9, 81.77) | 14.18 (5.79, 27.22) | 35.02 (13.64, 66.82) | 147.06 (46.57, 272.48) | 0.5 (0.21, 0.94) | 0.56 (0.23, 1.08) | 11.97 (-32.55, 66.48) | -1.39 (-13.34, 12.2) |
| Saint Kitts and Nevis | 0.05 (0.02, 0.08) | 0.09 (0.04, 0.16) | 95.58 (27.87, 206.4) | 0.113 (0.057, 0.184) | 0.156 (0.072, 0.272) | 38.31 (-9.57, 116.68) | 0.137 (0.069, 0.223) | 0.147 (0.07, 0.248) | 6.75 (-27.61, 63.03) | 1.31 (0.64, 2.25) | 2.74 (1.23, 4.98) | 108.73 (37.63, 225.77) | 3.91 (1.87, 6.73) | 3.82 (1.74, 6.81) | -2.29 (-33.7, 49.67) | -6.23 (-18.71, 8.17) |
| Saint Lucia | 0.08 (0.04, 0.13) | 0.26 (0.12, 0.44) | 237.24 (129.14, 394.95) | 0.056 (0.027, 0.094) | 0.145 (0.065, 0.249) | 159.45 (76.29, 280.79) | 0.094 (0.047, 0.156) | 0.111 (0.05, 0.187) | 17.77 (-17.97, 70.64) | 2.36 (1.02, 4.14) | 7.57 (3.37, 13.46) | 221.27 (121.25, 378.48) | 2.63 (1.2, 4.41) | 3.28 (1.46, 5.79) | 24.46 (-13.88, 84.7) | -3.16 (-22.17, 20.48) |
| Saint Vincent and the Grenadines | 0.04 (0.02, 0.07) | 0.23 (0.12, 0.38) | 453.22 (284.3, 676.57) | 0.039 (0.018, 0.066) | 0.205 (0.104, 0.329) | 431.04 (268.9, 645.44) | 0.063 (0.029, 0.104) | 0.178 (0.09, 0.284) | 184.07 (101.41, 299.67) | 1.33 (0.61, 2.48) | 7.13 (3.41, 12.21) | 434.84 (260.96, 652.03) | 1.83 (0.83, 3.31) | 5.32 (2.55, 9.06) | 190.41 (97.49, 302.82) | -9.06 (-22.42, 6.59) |
| Samoa | 0.09 (0.03, 0.17) | 0.36 (0.15, 0.65) | 318.15 (134.51, 671.99) | 0.05 (0.02, 0.098) | 0.166 (0.072, 0.305) | 230.64 (85.43, 510.44) | 0.109 (0.045, 0.207) | 0.267 (0.119, 0.487) | 144.9 (36.32, 345.85) | 2.63 (1.06, 5.15) | 10.12 (4.18, 19.16) | 284.29 (118.39, 591.87) | 2.92 (1.19, 5.61) | 6.75 (2.85, 12.59) | 131.05 (34.17, 317.01) | -1.98 (-17.88, 17) |
| San Marino | 0.02 (0.01, 0.04) | 0.04 (0.01, 0.07) | 73 (0.52, 173.13) | 0.087 (0.034, 0.158) | 0.109 (0.041, 0.218) | 25.52 (-27.07, 98.16) | 0.054 (0.021, 0.098) | 0.031 (0.012, 0.062) | -43.35 (-67.2, -11.81) | 0.63 (0.26, 1.18) | 1.02 (0.44, 1.93) | 61.31 (15.01, 119.02) | 1.84 (0.77, 3.45) | 1.42 (0.6, 2.77) | -22.89 (-43.42, 4.18) | -2.18 (-10.99, 7.5) |
| Sao Tome and Principe | 0.01 (0.01, 0.02) | 0.05 (0.02, 0.09) | 252.58 (111.43, 437.97) | 0.012 (0.005, 0.02) | 0.023 (0.009, 0.042) | 97.4 (18.38, 201.2) | 0.025 (0.011, 0.043) | 0.053 (0.022, 0.099) | 111.86 (25.18, 221.23) | 0.34 (0.14, 0.61) | 1.41 (0.53, 2.64) | 319.54 (158.09, 529.44) | 0.54 (0.24, 0.97) | 1.19 (0.47, 2.21) | 121.05 (40.58, 224.63) | -2.71 (-11.12, 6.5) |
| Saudi Arabia | 15.05 (6.57, 27.12) | 161.41 (64.81, 287.49) | 972.5 (482.81, 1654.03) | 0.095 (0.041, 0.171) | 0.428 (0.172, 0.762) | 351.04 (145.1, 637.66) | 0.259 (0.112, 0.472) | 0.746 (0.322, 1.311) | 187.64 (63.48, 357.58) | 526.75 (218.65, 964.12) | 6467.43 (2489.9, 11944.31) | 1127.8 (611.56, 1802.63) | 7.11 (3.1, 12.68) | 20.29 (8.52, 35.89) | 185.37 (60.3, 346.19) | 2.88 (1.42, 4.35) |
| Senegal | 0.43 (0.17, 0.81) | 1.35 (0.51, 2.53) | 216.72 (108.06, 386.36) | 0.006 (0.002, 0.011) | 0.009 (0.003, 0.016) | 52.42 (0.13, 134.06) | 0.015 (0.006, 0.029) | 0.019 (0.008, 0.036) | 26.24 (-18.41, 93.08) | 12.84 (5.04, 25.05) | 42.03 (15.35, 79.91) | 227.36 (120.47, 381.41) | 0.37 (0.16, 0.71) | 0.48 (0.18, 0.9) | 29.19 (-13.03, 93.56) | 1.32 (-10.88, 15.19) |
| Serbia | 6.08 (2.38, 11.93) | 8.94 (3.81, 16.32) | 47.08 (-8.24, 134.09) | 0.063 (0.025, 0.124) | 0.1 (0.043, 0.183) | 58.78 (-0.94, 152.73) | 0.067 (0.026, 0.13) | 0.053 (0.022, 0.097) | -20.32 (-49.49, 24.61) | 184.29 (71.69, 361.2) | 215 (90.78, 398.64) | 16.67 (-23.81, 83.9) | 1.75 (0.69, 3.47) | 1.42 (0.59, 2.71) | -18.94 (-46.89, 20.45) | 0.02 (-5.66, 6.04) |
| Seychelles | 0.02 (0.01, 0.03) | 0.05 (0.02, 0.1) | 232.54 (111.06, 441.82) | 0.021 (0.009, 0.037) | 0.049 (0.02, 0.093) | 129.84 (45.87, 274.48) | 0.028 (0.011, 0.049) | 0.047 (0.019, 0.089) | 70.87 (7.01, 177.3) | 0.46 (0.2, 0.82) | 1.45 (0.59, 2.74) | 217.9 (101.22, 395.11) | 0.81 (0.35, 1.48) | 1.2 (0.49, 2.25) | 47.46 (-5.35, 128.12) | -3.53 (-13.46, 7.53) |
| Sierra Leone | 0.13 (0.05, 0.26) | 0.35 (0.13, 0.69) | 164.91 (69.09, 293.94) | 0.003 (0.001, 0.006) | 0.004 (0.001, 0.008) | 24.03 (-20.83, 84.44) | 0.007 (0.003, 0.015) | 0.01 (0.004, 0.02) | 39.69 (-7.83, 103.97) | 3.83 (1.5, 7.7) | 12.19 (4.34, 25.64) | 218.36 (103.85, 358.27) | 0.18 (0.08, 0.36) | 0.28 (0.11, 0.53) | 52.35 (0.3, 111.08) | -4.03 (-20.25, 15.49) |
| Singapore | 0.65 (0.27, 1.17) | 4.22 (1.85, 7.33) | 547.58 (344.25, 851.44) | 0.021 (0.009, 0.038) | 0.074 (0.032, 0.128) | 244.54 (136.36, 406.21) | 0.036 (0.015, 0.064) | 0.051 (0.022, 0.088) | 41.8 (-2.78, 108.51) | 18.07 (7.62, 32.83) | 94.53 (42.83, 163.82) | 423.14 (295.79, 604.43) | 0.81 (0.34, 1.45) | 1.12 (0.51, 1.95) | 37.78 (1.5, 86.42) | -0.16 (-11.98, 13.25) |
| Slovakia | 2.63 (1.01, 4.96) | 4 (1.62, 7.65) | 52.12 (-4.61, 142) | 0.05 (0.019, 0.094) | 0.074 (0.03, 0.141) | 48.03 (-7.18, 135.48) | 0.045 (0.018, 0.086) | 0.043 (0.017, 0.082) | -5.95 (-40.07, 46.36) | 81.53 (34.15, 150.8) | 118.42 (49.76, 221.03) | 45.26 (1.39, 111.56) | 1.39 (0.57, 2.6) | 1.34 (0.56, 2.52) | -3.81 (-31.19, 34.53) | -0.25 (-8.73, 9.02) |
| Slovenia | 0.67 (0.24, 1.26) | 2.13 (0.8, 4.22) | 218.99 (88.59, 477.32) | 0.034 (0.012, 0.064) | 0.103 (0.039, 0.204) | 204.18 (79.83, 450.51) | 0.028 (0.01, 0.053) | 0.04 (0.016, 0.081) | 45.47 (-8.69, 148.51) | 27.02 (9.95, 50.57) | 58.43 (23.58, 110.35) | 116.22 (44.83, 240.87) | 1.13 (0.42, 2.15) | 1.46 (0.59, 2.73) | 29.11 (-12.58, 92.93) | -0.12 (-14.53, 16.7) |
| Solomon Islands | 0.04 (0.01, 0.09) | 0.12 (0.05, 0.22) | 186.72 (51.59, 576.67) | 0.012 (0.004, 0.026) | 0.018 (0.008, 0.032) | 42.22 (-24.81, 235.64) | 0.036 (0.012, 0.072) | 0.037 (0.017, 0.062) | 3.25 (-44.08, 125.77) | 1.46 (0.45, 3.1) | 4.51 (1.92, 8.36) | 209.01 (60.14, 564.59) | 0.97 (0.33, 1.98) | 1.06 (0.48, 1.89) | 9.58 (-39.68, 126.08) | -6.54 (-25.45, 17.15) |
| Somalia | 0.54 (0.21, 1.06) | 1.35 (0.5, 2.82) | 150.82 (52.74, 287.18) | 0.007 (0.003, 0.013) | 0.006 (0.002, 0.013) | -7.84 (-43.88, 42.26) | 0.029 (0.012, 0.056) | 0.027 (0.011, 0.057) | -4.19 (-39.61, 46.49) | 16.45 (6.29, 33.4) | 41.96 (16.06, 87.46) | 155.13 (57.03, 305.04) | 0.66 (0.26, 1.26) | 0.64 (0.24, 1.33) | -3.13 (-38.47, 48.23) | -0.79 (-11.52, 11.24) |
| South Africa | 11.39 (4.47, 21.63) | 49.15 (20.64, 90.48) | 331.35 (220.43, 469.93) | 0.031 (0.012, 0.058) | 0.086 (0.036, 0.159) | 180.82 (108.61, 271.03) | 0.055 (0.023, 0.102) | 0.113 (0.049, 0.201) | 104.39 (48.46, 171.4) | 500.82 (192.38, 997.2) | 1903.33 (791.29, 3642.94) | 280.04 (196.89, 393.89) | 2.03 (0.83, 3.88) | 3.66 (1.57, 6.73) | 80.75 (43.1, 130.89) | 3.23 (0.96, 5.55) |
| South Sudan | 1.64 (0.61, 3.45) | 3.89 (1.55, 7.66) | 137.66 (19.45, 336.09) | 0.028 (0.01, 0.059) | 0.04 (0.016, 0.079) | 44.41 (-27.42, 164.99) | 0.073 (0.028, 0.147) | 0.122 (0.05, 0.236) | 66.54 (-13.82, 200.93) | 42.04 (15.82, 89.1) | 110.41 (43.58, 219.11) | 162.6 (45.21, 366.58) | 1.64 (0.65, 3.44) | 2.74 (1.13, 5.44) | 67.03 (-7.67, 195.28) | 2.39 (-5.78, 11.27) |
| Spain | 39.67 (16.55, 71.93) | 90.91 (37.1, 178.18) | 129.13 (52.71, 261.19) | 0.102 (0.043, 0.185) | 0.2 (0.081, 0.391) | 95.09 (30.02, 207.53) | 0.078 (0.033, 0.143) | 0.065 (0.027, 0.126) | -17.07 (-43.11, 23.78) | 996.4 (435.49, 1776.88) | 1757.21 (753.98, 3190.92) | 76.36 (34.25, 132.45) | 1.98 (0.86, 3.57) | 1.79 (0.76, 3.38) | -9.3 (-29.86, 15.78) | -1.31 (-4.94, 2.46) |
| Sri Lanka | 2.21 (0.94, 3.98) | 9.61 (3.6, 19.1) | 334.4 (135, 650.77) | 0.013 (0.006, 0.023) | 0.043 (0.016, 0.086) | 234.15 (80.77, 477.51) | 0.024 (0.01, 0.041) | 0.038 (0.015, 0.076) | 61.95 (-10.55, 175.33) | 70.68 (28.99, 127.08) | 282.74 (107.75, 553.09) | 300.05 (126.64, 550.54) | 0.62 (0.26, 1.12) | 1.07 (0.42, 2.08) | 71.1 (-1.28, 171.91) | 1.71 (-3.84, 7.58) |
| Sudan | 1.36 (0.51, 2.96) | 5.45 (2.23, 10.46) | 301.14 (103.77, 582.23) | 0.007 (0.003, 0.015) | 0.013 (0.005, 0.024) | 84.97 (-6.04, 214.59) | 0.016 (0.006, 0.035) | 0.028 (0.012, 0.052) | 81.37 (-2.29, 206.8) | 47.72 (17.59, 99.06) | 198.19 (78.57, 388.36) | 315.31 (129.8, 584.52) | 0.46 (0.18, 0.93) | 0.81 (0.34, 1.51) | 73.67 (-0.76, 179) | 2.39 (-4.16, 9.38) |
| Suriname | 0.37 (0.16, 0.64) | 1.27 (0.58, 2.12) | 244.47 (122.29, 452.04) | 0.095 (0.042, 0.164) | 0.219 (0.1, 0.366) | 129.97 (48.4, 268.55) | 0.139 (0.063, 0.233) | 0.203 (0.096, 0.341) | 46.15 (-7.09, 132.61) | 13.27 (5.69, 23.64) | 43.16 (18.67, 73.56) | 225.37 (112.32, 401.75) | 4.52 (1.94, 7.83) | 6.8 (2.94, 12.03) | 50.31 (2.11, 135.45) | 2.01 (-9.61, 15.12) |
| Sweden | 2.45 (0.98, 4.51) | 14.7 (5.96, 28.11) | 499.34 (284.83, 834.16) | 0.029 (0.011, 0.053) | 0.142 (0.057, 0.271) | 396.16 (218.59, 673.34) | 0.014 (0.006, 0.027) | 0.05 (0.021, 0.096) | 246.05 (130.9, 431.07) | 83.48 (35.04, 150.74) | 267.05 (115.67, 506.35) | 219.9 (129.7, 351.58) | 0.58 (0.25, 1.06) | 1.2 (0.51, 2.31) | 106.93 (53.31, 175.2) | 0.17 (-10.37, 11.94) |
| Switzerland | 3.19 (1.37, 6.06) | 10.15 (3.96, 19.69) | 217.92 (93.86, 382.07) | 0.046 (0.02, 0.088) | 0.114 (0.044, 0.221) | 144.65 (49.18, 270.97) | 0.028 (0.012, 0.054) | 0.039 (0.016, 0.073) | 36.46 (-10.63, 99.85) | 97.57 (44.47, 185.79) | 199.84 (83.09, 376.24) | 104.81 (52.58, 167.75) | 0.97 (0.43, 1.86) | 1.08 (0.46, 2.03) | 11.31 (-13.98, 40.62) | 0.75 (-12.71, 16.27) |
| Syrian Arab Republic | 4.08 (1.66, 7.96) | 8.66 (3.45, 16.88) | 112.3 (9.77, 275.55) | 0.032 (0.013, 0.063) | 0.062 (0.025, 0.12) | 92.43 (-0.5, 240.4) | 0.084 (0.034, 0.166) | 0.076 (0.031, 0.145) | -9.22 (-53.04, 59.63) | 128.31 (51.95, 254.68) | 258.98 (103.24, 489.83) | 101.83 (11.92, 249.74) | 2.18 (0.92, 4.28) | 1.95 (0.79, 3.7) | -10.58 (-49.15, 48.58) | -1.02 (-5.01, 3.13) |
| Taiwan (Province of China) | 8.82 (3.95, 14.62) | 37.27 (16.65, 65.77) | 322.41 (186.32, 517.84) | 0.043 (0.019, 0.072) | 0.158 (0.07, 0.278) | 264.4 (147, 432.99) | 0.08 (0.037, 0.137) | 0.083 (0.037, 0.143) | 3.55 (-30.92, 55.21) | 223.03 (97.44, 386.86) | 847.04 (365.99, 1493.98) | 279.78 (181.41, 413.88) | 1.58 (0.7, 2.7) | 2.01 (0.87, 3.51) | 27.08 (-6.89, 72.75) | 1.9 (-2.67, 6.68) |
| Tajikistan | 0.02 (0.01, 0.05) | 0.06 (0.02, 0.14) | 189.43 (60.93, 366.62) | 0 (0, 0.001) | 0.001 (0, 0.001) | 52.95 (-14.96, 146.58) | 0.001 (0, 0.002) | 0.001 (0, 0.002) | 34.14 (-23.35, 115.41) | 3.65 (1.54, 7.64) | 8.86 (3.39, 18.16) | 142.39 (64.21, 252.38) | 0.12 (0.05, 0.24) | 0.12 (0.05, 0.25) | 3.67 (-26.22, 42.91) | -4.18 (-14.03, 6.81) |
| Thailand | 13.1 (5.77, 23.85) | 128.32 (57.2, 226.98) | 879.69 (561.01, 1354.53) | 0.023 (0.01, 0.042) | 0.192 (0.086, 0.34) | 733.97 (462.69, 1138.18) | 0.044 (0.019, 0.081) | 0.118 (0.053, 0.209) | 169.64 (82.05, 319.46) | 403.56 (178.13, 729.83) | 3453.4 (1526.9, 6281.09) | 755.74 (479.92, 1116.47) | 1.11 (0.49, 2.01) | 3.22 (1.42, 6.07) | 190.61 (103.15, 312.26) | 3.05 (1.08, 5.05) |
| Timor-Leste | 0.03 (0.01, 0.06) | 0.1 (0.04, 0.19) | 249.57 (112.39, 441.91) | 0.004 (0.002, 0.007) | 0.007 (0.003, 0.014) | 95.36 (18.7, 202.85) | 0.013 (0.006, 0.024) | 0.014 (0.005, 0.025) | 2.6 (-35.3, 54.88) | 0.98 (0.43, 1.85) | 3.01 (1.18, 5.64) | 208.1 (88.48, 375.67) | 0.33 (0.14, 0.6) | 0.35 (0.14, 0.65) | 6.48 (-30.9, 63.35) | -7.74 (-19.82, 6.16) |
| Togo | 0.09 (0.04, 0.18) | 0.63 (0.24, 1.24) | 570.72 (326.01, 900.94) | 0.003 (0.001, 0.005) | 0.007 (0.003, 0.015) | 192.19 (85.59, 336.05) | 0.01 (0.004, 0.018) | 0.022 (0.009, 0.043) | 122.66 (41.46, 237.06) | 2.87 (1.18, 5.42) | 20.29 (7.78, 41.74) | 607.47 (364.51, 929.86) | 0.23 (0.1, 0.43) | 0.52 (0.21, 1) | 122.68 (48.84, 221.09) | -3.47 (-17.96, 13.59) |
| Tokelau | 0 (0, 0) | 0 (0, 0) | 252.44 (90.08, 593.52) | 0.043 (0.017, 0.087) | 0.175 (0.072, 0.321) | 309.92 (121.09, 706.65) | 0.056 (0.023, 0.114) | 0.162 (0.068, 0.299) | 192.29 (64.37, 464.59) | 0.02 (0.01, 0.04) | 0.06 (0.02, 0.11) | 194 (62.79, 414.76) | 1.53 (0.63, 2.98) | 3.95 (1.67, 7.35) | 158.01 (50.39, 337.29) | -0.24 (-8.42, 8.66) |
| Tonga | 0.02 (0.01, 0.04) | 0.06 (0.02, 0.11) | 227.09 (49.67, 594.48) | 0.018 (0.007, 0.039) | 0.055 (0.022, 0.106) | 204.17 (39.18, 545.82) | 0.034 (0.014, 0.07) | 0.075 (0.029, 0.144) | 119.17 (6.68, 345.73) | 0.64 (0.25, 1.3) | 1.68 (0.69, 3.09) | 161.86 (30.12, 419.59) | 1.08 (0.43, 2.16) | 2.05 (0.85, 3.83) | 90.16 (-3.96, 278.78) | -2.92 (-13.55, 9.02) |
| Trinidad and Tobago | 0.89 (0.42, 1.47) | 4.87 (2.21, 8.36) | 446.11 (228.23, 745.14) | 0.074 (0.035, 0.122) | 0.349 (0.158, 0.6) | 372.28 (183.86, 630.89) | 0.113 (0.056, 0.183) | 0.26 (0.119, 0.45) | 130.3 (39.97, 254.21) | 31.09 (13.88, 53.02) | 143.97 (63.31, 245.19) | 363.13 (187.63, 639.76) | 3.38 (1.54, 5.59) | 7.9 (3.48, 13.51) | 133.37 (49.6, 256.55) | 4.27 (-3.63, 12.83) |
| Tunisia | 2.27 (0.92, 4.44) | 12.98 (5.08, 24.81) | 471.14 (209.54, 907.52) | 0.027 (0.011, 0.053) | 0.11 (0.043, 0.209) | 302.68 (118.24, 610.34) | 0.052 (0.021, 0.101) | 0.106 (0.042, 0.206) | 102.75 (13.72, 247.31) | 74.95 (31.21, 151.58) | 361.36 (138.59, 690.06) | 382.14 (171.94, 715.86) | 1.42 (0.58, 2.84) | 2.74 (1.05, 5.23) | 92.81 (7.83, 219.93) | 2.5 (-2.44, 7.7) |
| Turkey | 39.27 (16.52, 75.11) | 141.92 (61.31, 267.38) | 261.42 (125.51, 457.13) | 0.068 (0.029, 0.131) | 0.17 (0.073, 0.32) | 148.43 (55.01, 282.96) | 0.133 (0.056, 0.248) | 0.163 (0.07, 0.31) | 22.65 (-24.55, 91.53) | 1137.56 (458.3, 2179.01) | 3746.5 (1648.06, 7019.42) | 229.35 (119.35, 384.14) | 3.18 (1.36, 5.95) | 4.02 (1.75, 7.46) | 26.3 (-15.58, 83.92) | 1.72 (0.12, 3.35) |
| Turkmenistan | 0.39 (0.14, 0.83) | 3.62 (1.32, 7.6) | 829.59 (456.67, 1391.06) | 0.011 (0.004, 0.022) | 0.07 (0.026, 0.147) | 566.64 (299.2, 969.29) | 0.018 (0.007, 0.037) | 0.084 (0.032, 0.17) | 357.85 (180.1, 632.86) | 25.06 (9.6, 54.92) | 180.04 (66.63, 385.65) | 618.34 (338.88, 994.1) | 1.1 (0.43, 2.25) | 3.78 (1.45, 7.81) | 244.98 (120.01, 411.73) | 5.87 (-4.25, 17.06) |
| Tuvalu | 0 (0, 0.01) | 0.01 (0, 0.02) | 244.65 (76.83, 552.55) | 0.036 (0.015, 0.074) | 0.094 (0.036, 0.18) | 165.09 (36.01, 401.91) | 0.056 (0.025, 0.11) | 0.122 (0.048, 0.231) | 117.25 (12.54, 293.87) | 0.11 (0.04, 0.22) | 0.33 (0.13, 0.62) | 206.45 (65.99, 429.17) | 1.54 (0.64, 3.08) | 3.13 (1.22, 5.85) | 103.23 (11.74, 244.09) | -1.43 (-9.51, 7.37) |
| Uganda | 1.16 (0.47, 2.44) | 4.34 (1.62, 8.39) | 275.15 (124.41, 515.02) | 0.007 (0.003, 0.014) | 0.01 (0.004, 0.019) | 49.76 (-10.42, 145.51) | 0.021 (0.009, 0.043) | 0.035 (0.014, 0.068) | 64.94 (0.75, 169.25) | 31.29 (12.7, 64.74) | 122.46 (45.28, 237.18) | 291.42 (133.66, 544.8) | 0.49 (0.2, 0.98) | 0.79 (0.3, 1.51) | 62.63 (-0.05, 162.28) | 1.03 (-6.76, 9.48) |
| Ukraine | 0.07 (0.02, 0.15) | 1.37 (0.46, 3.21) | 2005.69 (1288.91, 3067.89) | 0 (0, 0) | 0.003 (0.001, 0.007) | 2476.68 (1599.58, 3776.45) | 0 (0, 0) | 0.002 (0.001, 0.005) | 1804.09 (1121.72, 2801.75) | 92.04 (36.68, 179.12) | 139.34 (54.97, 279.23) | 51.39 (5.7, 122.47) | 0.14 (0.06, 0.28) | 0.2 (0.08, 0.42) | 43.64 (-1.96, 114.43) | 0.97 (-9.03, 12.08) |
| United Arab Emirates | 0.94 (0.39, 1.81) | 6.94 (2.62, 12.48) | 635.84 (304.41, 1086.97) | 0.05 (0.021, 0.097) | 0.072 (0.027, 0.13) | 42.94 (-21.44, 130.57) | 0.186 (0.083, 0.327) | 0.276 (0.104, 0.485) | 48.59 (-21.63, 144.12) | 58.07 (23.48, 115.91) | 383.32 (151.46, 677.82) | 560.13 (327.53, 880.86) | 5.83 (2.49, 10.48) | 6.64 (2.58, 11.72) | 13.8 (-27.9, 73.2) | 1.34 (-5.6, 8.79) |
| United Kingdom | 21.43 (9.47, 39.25) | 53.74 (23.32, 100.63) | 150.72 (65.16, 270.4) | 0.037 (0.017, 0.069) | 0.079 (0.034, 0.148) | 111.74 (39.48, 212.81) | 0.023 (0.01, 0.042) | 0.034 (0.015, 0.062) | 48.08 (1.13, 115.08) | 937.86 (433.06, 1749.4) | 1741.31 (780.31, 3200.3) | 85.67 (41.38, 141.83) | 1.15 (0.52, 2.14) | 1.54 (0.67, 2.89) | 34.2 (3.64, 71.02) | 2.48 (-2.1, 7.27) |
| United Republic of Tanzania | 3.73 (1.39, 7.39) | 11.94 (4.83, 23.26) | 220.46 (100.18, 413.03) | 0.014 (0.005, 0.029) | 0.02 (0.008, 0.04) | 41.66 (-11.51, 126.79) | 0.04 (0.016, 0.076) | 0.052 (0.021, 0.097) | 30.72 (-19.86, 104.04) | 113.61 (43.29, 227.64) | 376.65 (141.21, 781.47) | 231.52 (108.15, 417.01) | 0.98 (0.4, 1.91) | 1.29 (0.52, 2.56) | 31.9 (-14.32, 104.77) | 1.27 (-3.38, 6.15) |
| United States of America | 276.47 (126.08, 489.18) | 1649.5 (812.35, 2690.32) | 496.62 (351.68, 692.41) | 0.109 (0.05, 0.193) | 0.496 (0.244, 0.809) | 355.74 (245.02, 505.29) | 0.085 (0.04, 0.15) | 0.283 (0.139, 0.462) | 231.43 (157.86, 336.5) | 9168.16 (4111.42, 16528.14) | 42962.23 (21090.31, 71722.07) | 368.6 (273.38, 507.27) | 3.01 (1.36, 5.35) | 8.61 (4.13, 14.51) | 186.2 (131.28, 263.99) | 4.34 (3.78, 4.89) |
| United States Virgin Islands | 0.08 (0.04, 0.14) | 0.24 (0.1, 0.44) | 206.96 (64.25, 422.84) | 0.075 (0.035, 0.128) | 0.283 (0.12, 0.507) | 279 (102.79, 545.53) | 0.096 (0.045, 0.165) | 0.161 (0.071, 0.279) | 68.17 (-2.97, 161.55) | 3.02 (1.34, 5.14) | 6.93 (3.07, 12.08) | 129.54 (34, 282.43) | 3.13 (1.41, 5.41) | 5.57 (2.41, 9.82) | 78.04 (11.5, 176.79) | -0.49 (-20.01, 23.81) |
| Uruguay | 3.12 (1.31, 5.99) | 9.76 (4.01, 17.97) | 213.27 (115.47, 372.58) | 0.099 (0.042, 0.191) | 0.287 (0.118, 0.528) | 188.79 (98.63, 335.64) | 0.082 (0.034, 0.156) | 0.149 (0.062, 0.268) | 82.58 (30.44, 176.25) | 71.84 (29.98, 139.48) | 185.96 (78.08, 328.49) | 158.87 (90.3, 272.8) | 1.9 (0.8, 3.7) | 3.4 (1.42, 6.09) | 79.33 (31.77, 149.55) | 2.46 (-5.59, 11.21) |
| Uzbekistan | 0.99 (0.32, 2.38) | 7.45 (2.69, 15.15) | 654.65 (285.2, 1331.57) | 0.005 (0.002, 0.011) | 0.022 (0.008, 0.044) | 362.03 (135.84, 776.47) | 0.009 (0.003, 0.022) | 0.027 (0.011, 0.053) | 211 (54.11, 481.74) | 83.05 (31.8, 175.52) | 425.43 (168.79, 900.45) | 412.28 (222.65, 726.38) | 0.64 (0.26, 1.35) | 1.33 (0.53, 2.77) | 107.33 (29.28, 223.65) | 3.05 (-2.84, 9.3) |
| Vanuatu | 0.03 (0.01, 0.07) | 0.14 (0.06, 0.26) | 320.77 (137.27, 663.8) | 0.021 (0.008, 0.044) | 0.043 (0.018, 0.084) | 104.65 (15.4, 271.5) | 0.061 (0.026, 0.122) | 0.085 (0.038, 0.168) | 38.67 (-16.63, 138.66) | 1.08 (0.43, 2.22) | 4.68 (1.86, 8.99) | 334.26 (150.34, 661.67) | 1.59 (0.65, 3.15) | 2.34 (1, 4.45) | 46.79 (-13.76, 150.9) | -6.46 (-23.55, 14.46) |
| Venezuela (Bolivarian Republic of) | 6.34 (2.67, 11.67) | 38.99 (16.59, 70.66) | 514.94 (325.43, 791.73) | 0.034 (0.014, 0.062) | 0.146 (0.062, 0.265) | 334.32 (200.47, 529.81) | 0.067 (0.029, 0.121) | 0.132 (0.056, 0.242) | 97.28 (34.63, 181.91) | 263.03 (107.98, 521.58) | 1169.93 (463.8, 2146.97) | 344.79 (212.83, 553.24) | 2.31 (1, 4.36) | 3.87 (1.52, 7.1) | 67.2 (20.25, 136.79) | 2.54 (-0.27, 5.43) |
| Viet Nam | 2.72 (1.15, 4.91) | 17.49 (7.33, 30.97) | 543.35 (334.19, 837.71) | 0.004 (0.002, 0.007) | 0.017 (0.007, 0.031) | 337.76 (195.44, 538.06) | 0.007 (0.003, 0.014) | 0.02 (0.009, 0.035) | 164.06 (74.63, 285.47) | 71.16 (30.6, 127.51) | 464.15 (188.95, 822.31) | 552.25 (348.2, 846.36) | 0.18 (0.08, 0.32) | 0.46 (0.19, 0.82) | 158.27 (80.66, 270.19) | 4.32 (-1.45, 10.42) |
| Yemen | 0.51 (0.2, 1.19) | 1.16 (0.48, 2.49) | 126.53 (32.64, 277.77) | 0.004 (0.001, 0.009) | 0.003 (0.001, 0.007) | -8.19 (-46.24, 53.09) | 0.012 (0.005, 0.027) | 0.009 (0.004, 0.019) | -23.75 (-54.49, 26.73) | 16.95 (6.56, 38.74) | 40.46 (16.57, 88.48) | 138.7 (45.15, 285.39) | 0.32 (0.13, 0.72) | 0.25 (0.11, 0.52) | -22.78 (-52.38, 22) | -0.6 (-12.27, 12.61) |
| Zambia | 0.91 (0.36, 1.66) | 2.73 (1.05, 5.38) | 199.69 (78.49, 438.76) | 0.011 (0.004, 0.021) | 0.014 (0.005, 0.028) | 21.88 (-27.41, 119.1) | 0.037 (0.016, 0.068) | 0.044 (0.019, 0.09) | 18.31 (-24.21, 102.11) | 26.11 (10.04, 48.64) | 86.18 (31.94, 193.63) | 230.06 (87.37, 490.03) | 0.88 (0.35, 1.59) | 1.07 (0.42, 2.12) | 21.76 (-25.65, 108.98) | 0.69 (-8.02, 10.22) |
| Zimbabwe | 0.97 (0.38, 1.97) | 2.82 (1.01, 5.87) | 190.46 (64.54, 351.56) | 0.009 (0.004, 0.019) | 0.018 (0.006, 0.038) | 92.63 (9.12, 199.47) | 0.027 (0.011, 0.054) | 0.041 (0.016, 0.08) | 51.4 (-8.55, 130.82) | 39.95 (15.83, 83.19) | 119.7 (44.21, 260.96) | 199.59 (82.24, 376.29) | 0.86 (0.35, 1.74) | 1.32 (0.51, 2.67) | 53.61 (-5.71, 136.41) | 2 (-5.49, 10.08) |

**Appendix 4.1** The local drift of mortality from 1990 to 2021 for SSBs-related CKD across SDI

| Age | Global | Low SDI | Low-middle SDI | Middle SDI | High-middle SDI | High SDI |
| --- | --- | --- | --- | --- | --- | --- |
| 25 to 29 | 3.76 (2.75, 4.77) | 1.06 (-3.28, 5.59) | 3.34 (0.61, 6.15) | 3.87 (2.39, 5.37) | 2.44 (-0.72, 5.71) | 5.94 (3.88, 8.04) |
| 30 to 34 | 3.6 (2.81, 4.39) | 0.85 (-2.91, 4.75) | 3.31 (1.09, 5.58) | 3.36 (2.19, 4.55) | 2.35 (-0.01, 4.77) | 6.03 (4.49, 7.59) |
| 35 to 39 | 3.53 (2.87, 4.2) | 0.67 (-2.73, 4.19) | 3.31 (1.41, 5.25) | 3.14 (2.13, 4.16) | 2.47 (0.53, 4.43) | 5.86 (4.61, 7.11) |
| 40 to 44 | 3.32 (2.76, 3.88) | 0.65 (-2.38, 3.78) | 3.2 (1.56, 4.86) | 2.92 (2.05, 3.78) | 2.16 (0.57, 3.77) | 5.45 (4.42, 6.5) |
| 45 to 49 | 2.98 (2.5, 3.45) | 0.61 (-2.07, 3.36) | 3.04 (1.64, 4.45) | 2.53 (1.79, 3.27) | 1.68 (0.38, 3.01) | 4.92 (4.05, 5.81) |
| 50 to 54 | 2.78 (2.37, 3.19) | 0.54 (-1.78, 2.92) | 3.04 (1.85, 4.26) | 2.34 (1.69, 2.99) | 1.41 (0.32, 2.5) | 4.41 (3.66, 5.17) |
| 55 to 59 | 2.82 (2.45, 3.18) | 0.46 (-1.59, 2.55) | 3.07 (1.99, 4.16) | 2.5 (1.91, 3.1) | 1.52 (0.6, 2.46) | 3.97 (3.31, 4.64) |
| 60 to 64 | 2.6 (2.27, 2.93) | 0.21 (-1.68, 2.13) | 2.76 (1.75, 3.79) | 2.6 (2.04, 3.16) | 1.45 (0.65, 2.26) | 3.31 (2.72, 3.89) |
| 65 to 69 | 2.29 (1.98, 2.61) | 0.1 (-1.76, 2) | 2.43 (1.44, 3.44) | 2.62 (2.06, 3.18) | 1.45 (0.73, 2.18) | 2.6 (2.08, 3.13) |
| 70 to 74 | 1.99 (1.69, 2.3) | 0.19 (-1.74, 2.16) | 2.17 (1.12, 3.23) | 2.76 (2.17, 3.35) | 1.3 (0.62, 1.99) | 2.06 (1.59, 2.54) |
| 75 to 79 | 1.71 (1.4, 2.02) | 0.41 (-1.81, 2.68) | 2.1 (0.89, 3.33) | 2.75 (2.08, 3.42) | 0.9 (0.23, 1.58) | 1.77 (1.32, 2.23) |
| 80 to 84 | 1.49 (1.15, 1.82) | 0.85 (-2.15, 3.96) | 2.35 (0.74, 3.99) | 2.36 (1.53, 3.2) | 0.57 (-0.14, 1.28) | 1.82 (1.36, 2.29) |
| 85 to 89 | 1.49 (1.01, 1.97) | 1.5 (-3.32, 6.57) | 2.89 (0.38, 5.46) | 1.69 (0.48, 2.93) | 0.74 (-0.21, 1.7) | 1.99 (1.34, 2.64) |

**Appendix 4.2** The local drift of mortality from 1990 to 2021 for SSBs-related CKD across SDI in males

| Age | Global | Low SDI | Low-middle SDI | Middle SDI | High-middle SDI | High SDI |
| --- | --- | --- | --- | --- | --- | --- |
| 25 to 29 | 3.76 (2.38, 5.16) | 1.34 (-4.49, 7.52) | 3.61 (-0.21, 7.58) | 4.3 (2.27, 6.37) | 2.37 (-1.92, 6.85) | 5.18 (2.39, 8.04) |
| 30 to 34 | 3.61 (2.52, 4.71) | 1.15 (-4.06, 6.63) | 3.59 (0.44, 6.85) | 3.75 (2.11, 5.43) | 2.42 (-0.8, 5.75) | 5.29 (3.21, 7.4) |
| 35 to 39 | 3.52 (2.6, 4.45) | 0.87 (-3.82, 5.78) | 3.47 (0.75, 6.26) | 3.46 (2.04, 4.89) | 2.59 (-0.06, 5.31) | 5.21 (3.53, 6.93) |
| 40 to 44 | 3.3 (2.52, 4.09) | 0.72 (-3.45, 5.07) | 3.28 (0.96, 5.66) | 3.2 (1.99, 4.42) | 2.31 (0.11, 4.57) | 4.92 (3.51, 6.35) |
| 45 to 49 | 3 (2.34, 3.67) | 0.6 (-3.1, 4.45) | 3.02 (1.05, 5.03) | 2.85 (1.82, 3.89) | 1.82 (-0.01, 3.69) | 4.57 (3.38, 5.78) |
| 50 to 54 | 2.88 (2.31, 3.46) | 0.5 (-2.72, 3.81) | 3.01 (1.32, 4.72) | 2.7 (1.79, 3.62) | 1.47 (-0.07, 3.04) | 4.36 (3.32, 5.41) |
| 55 to 59 | 2.99 (2.47, 3.5) | 0.33 (-2.49, 3.23) | 2.99 (1.48, 4.52) | 2.85 (2.01, 3.69) | 1.43 (0.1, 2.78) | 4.26 (3.33, 5.19) |
| 60 to 64 | 2.85 (2.38, 3.32) | 0.05 (-2.52, 2.7) | 2.73 (1.32, 4.16) | 2.88 (2.09, 3.68) | 1.23 (0.07, 2.41) | 3.9 (3.07, 4.74) |
| 65 to 69 | 2.58 (2.14, 3.03) | -0.09 (-2.61, 2.49) | 2.4 (1.01, 3.8) | 2.77 (1.98, 3.57) | 1.05 (-0.01, 2.12) | 3.38 (2.62, 4.15) |
| 70 to 74 | 2.23 (1.79, 2.67) | -0.11 (-2.72, 2.57) | 2.18 (0.7, 3.67) | 2.7 (1.86, 3.55) | 0.73 (-0.28, 1.74) | 2.75 (2.05, 3.46) |
| 75 to 79 | 1.86 (1.4, 2.32) | 0.06 (-3.01, 3.22) | 2.12 (0.4, 3.87) | 2.57 (1.61, 3.55) | 0.27 (-0.75, 1.29) | 2.2 (1.51, 2.88) |
| 80 to 84 | 1.47 (0.95, 1.99) | 0.49 (-3.83, 4.99) | 2.38 (0.09, 4.72) | 2.18 (0.94, 3.42) | -0.16 (-1.24, 0.94) | 1.86 (1.13, 2.59) |
| 85 to 89 | 1.38 (0.63, 2.14) | 1.09 (-6.03, 8.76) | 2.97 (-0.63, 6.69) | 1.94 (0.05, 3.87) | -0.01 (-1.51, 1.51) | 1.7 (0.65, 2.75) |

**Appendix 4.3** The local drift of mortality from 1990 to 2021 for SSBs-related CKD across SDI in females

| Age | Global | Low SDI | Low-middle SDI | Middle SDI | High-middle SDI | High SDI |
| --- | --- | --- | --- | --- | --- | --- |
| 25 to 29 | 3.73 (2.26, 5.21) | 0.75 (-5.64, 7.58) | 3.07 (-0.81, 7.1) | 3.39 (1.25, 5.58) | 2.57 (-2.06, 7.41) | 6.79 (3.76, 9.92) |
| 30 to 34 | 3.57 (2.44, 4.72) | 0.55 (-4.79, 6.19) | 3.03 (-0.07, 6.22) | 2.95 (1.27, 4.66) | 2.3 (-1.15, 5.88) | 6.85 (4.58, 9.17) |
| 35 to 39 | 3.53 (2.59, 4.49) | 0.48 (-4.39, 5.59) | 3.16 (0.5, 5.88) | 2.81 (1.38, 4.26) | 2.35 (-0.44, 5.23) | 6.55 (4.73, 8.42) |
| 40 to 44 | 3.32 (2.52, 4.13) | 0.59 (-3.77, 5.15) | 3.12 (0.83, 5.46) | 2.63 (1.4, 3.86) | 2.02 (-0.27, 4.35) | 6.02 (4.49, 7.58) |
| 45 to 49 | 2.94 (2.26, 3.63) | 0.63 (-3.21, 4.62) | 3.05 (1.09, 5.05) | 2.2 (1.15, 3.25) | 1.56 (-0.29, 3.45) | 5.29 (4.01, 6.59) |
| 50 to 54 | 2.66 (2.07, 3.25) | 0.6 (-2.74, 4.05) | 3.09 (1.39, 4.81) | 1.97 (1.05, 2.9) | 1.35 (-0.17, 2.9) | 4.46 (3.36, 5.56) |
| 55 to 59 | 2.64 (2.12, 3.16) | 0.63 (-2.32, 3.66) | 3.16 (1.62, 4.73) | 2.16 (1.32, 3) | 1.62 (0.33, 2.93) | 3.66 (2.72, 4.62) |
| 60 to 64 | 2.34 (1.87, 2.8) | 0.43 (-2.33, 3.28) | 2.81 (1.35, 4.28) | 2.32 (1.53, 3.11) | 1.66 (0.56, 2.78) | 2.7 (1.87, 3.53) |
| 65 to 69 | 2.01 (1.57, 2.44) | 0.38 (-2.37, 3.2) | 2.48 (1.05, 3.93) | 2.47 (1.69, 3.26) | 1.79 (0.8, 2.78) | 1.84 (1.11, 2.57) |
| 70 to 74 | 1.76 (1.35, 2.18) | 0.56 (-2.3, 3.5) | 2.18 (0.68, 3.7) | 2.79 (1.97, 3.62) | 1.74 (0.81, 2.67) | 1.38 (0.73, 2.04) |
| 75 to 79 | 1.55 (1.14, 1.97) | 0.8 (-2.4, 4.12) | 2.09 (0.4, 3.82) | 2.89 (1.97, 3.82) | 1.33 (0.42, 2.24) | 1.33 (0.72, 1.94) |
| 80 to 84 | 1.45 (1, 1.9) | 1.19 (-2.97, 5.52) | 2.35 (0.09, 4.67) | 2.5 (1.37, 3.65) | 0.99 (0.06, 1.93) | 1.66 (1.05, 2.28) |
| 85 to 89 | 1.5 (0.89, 2.12) | 1.82 (-4.62, 8.7) | 2.82 (-0.64, 6.41) | 1.51 (-0.08, 3.12) | 1.12 (-0.1, 2.36) | 2.01 (1.19, 2.83) |

**Appendix 5** The local drift of mortality from 1990 to 2021 for SSBs-related CKD across countries

| Country | Age | Local drift | | |
| --- | --- | --- | --- | --- |
|  |  | Both | Male | Female |
| Afghanistan | 25 to 29 | 1.47 (-27.21, 41.44) | -4.24 (-46.95, 72.86) | 1.7 (-31.28, 50.5) |
| Afghanistan | 30 to 34 | 1.2 (-24.16, 35.04) | -9.13 (-39.5, 36.5) | 1.37 (-28.26, 43.23) |
| Afghanistan | 35 to 39 | 0.58 (-21.57, 28.99) | -11.02 (-38.11, 27.94) | 1.51 (-24.71, 36.86) |
| Afghanistan | 40 to 44 | 0.49 (-17.47, 22.35) | -8.06 (-33.48, 27.08) | 1.57 (-19.84, 28.69) |
| Afghanistan | 45 to 49 | 0.48 (-14.32, 17.82) | -4.56 (-29.1, 28.48) | 1.21 (-16.11, 22.12) |
| Afghanistan | 50 to 54 | 0.59 (-12.76, 15.97) | -1.05 (-25.84, 32.03) | 0.94 (-14.41, 19.03) |
| Afghanistan | 55 to 59 | 0.95 (-12.33, 16.24) | 2.33 (-22.74, 35.53) | 0.87 (-14.24, 18.64) |
| Afghanistan | 60 to 64 | 1.13 (-11.39, 15.42) | 0.81 (-21.08, 28.78) | 1.11 (-13.58, 18.3) |
| Afghanistan | 65 to 69 | 1.11 (-10.75, 14.55) | -0.14 (-19.64, 24.09) | 1.2 (-13.21, 18.01) |
| Afghanistan | 70 to 74 | 1.22 (-11.4, 15.65) | -0.09 (-19.63, 24.2) | 1.51 (-14.36, 20.34) |
| Afghanistan | 75 to 79 | 1.27 (-13.71, 18.84) | 0.23 (-21.44, 27.86) | 1.59 (-17.77, 25.51) |
| Afghanistan | 80 to 84 | 1.41 (-18.77, 26.6) | 0.13 (-26.65, 36.69) | 1.44 (-23.96, 35.31) |
| Afghanistan | 85 to 89 | 1.46 (-27.96, 42.89) | 1.13 (-38.07, 65.14) | 1.59 (-36.96, 63.7) |
| Albania | 25 to 29 | 4.43 (-42.53, 89.74) | -8.72 (-54.83, 84.45) | -8.06 (-52.2, 76.86) |
| Albania | 30 to 34 | 4.86 (-34.55, 68.02) | -7.46 (-44.75, 54.98) | -7.69 (-42.85, 49.11) |
| Albania | 35 to 39 | 5.05 (-30.83, 59.53) | -4.2 (-38.05, 48.16) | -5.45 (-37.2, 42.34) |
| Albania | 40 to 44 | 5.14 (-26.83, 51.09) | 1.57 (-30.77, 49.02) | -1.01 (-31.58, 43.23) |
| Albania | 45 to 49 | 5.25 (-22.46, 42.85) | 2.17 (-27.67, 44.33) | 2.76 (-25.95, 42.61) |
| Albania | 50 to 54 | 4.41 (-19.31, 35.09) | 2.77 (-25.39, 41.56) | 3.11 (-24.64, 41.08) |
| Albania | 55 to 59 | 4.12 (-16.05, 29.14) | 3.26 (-23.19, 38.82) | 3.01 (-22.82, 37.48) |
| Albania | 60 to 64 | 3.49 (-14.2, 24.83) | 3.26 (-20.56, 34.24) | 3.28 (-19.99, 33.31) |
| Albania | 65 to 69 | 2.99 (-13.18, 22.17) | 2.32 (-19.81, 30.55) | 2.51 (-18.83, 29.46) |
| Albania | 70 to 74 | 2.55 (-12.58, 20.29) | 1.76 (-18.95, 27.78) | 2.67 (-17.38, 27.57) |
| Albania | 75 to 79 | 3.16 (-12.17, 21.17) | 1.74 (-18.93, 27.7) | 3.64 (-16.69, 28.93) |
| Albania | 80 to 84 | 2.97 (-15.37, 25.29) | 1.08 (-23.61, 33.75) | 4.04 (-20.84, 36.73) |
| Albania | 85 to 89 | 3.12 (-22.92, 37.97) | 1.23 (-35.54, 58.99) | 5.87 (-32.02, 64.88) |
| Algeria | 25 to 29 | 4.17 (-7.46, 17.28) | 3.42 (-13.91, 24.23) | 4.62 (-10.41, 22.19) |
| Algeria | 30 to 34 | 4.09 (-5.29, 14.39) | 3.37 (-11.08, 20.16) | 4.49 (-7.48, 18.02) |
| Algeria | 35 to 39 | 3.9 (-4.33, 12.84) | 3.24 (-9.89, 18.29) | 4.3 (-6.02, 15.75) |
| Algeria | 40 to 44 | 3.7 (-3.78, 11.76) | 2.97 (-9.18, 16.73) | 4.13 (-5.19, 14.36) |
| Algeria | 45 to 49 | 3.49 (-3.37, 10.83) | 2.71 (-8.5, 15.29) | 3.94 (-4.59, 13.24) |
| Algeria | 50 to 54 | 3.13 (-3.14, 9.82) | 2.5 (-7.68, 13.79) | 3.56 (-4.29, 12.05) |
| Algeria | 55 to 59 | 3.09 (-2.75, 9.28) | 2.52 (-6.81, 12.79) | 3.54 (-3.83, 11.48) |
| Algeria | 60 to 64 | 3.17 (-2.28, 8.92) | 2.65 (-5.95, 12.02) | 3.64 (-3.3, 11.07) |
| Algeria | 65 to 69 | 3.49 (-1.97, 9.26) | 2.95 (-5.54, 12.2) | 3.98 (-3.05, 11.53) |
| Algeria | 70 to 74 | 3.87 (-1.83, 9.9) | 3.13 (-5.57, 12.62) | 4.48 (-2.97, 12.49) |
| Algeria | 75 to 79 | 4.14 (-1.74, 10.36) | 3.1 (-5.71, 12.73) | 4.97 (-2.8, 13.35) |
| Algeria | 80 to 84 | 4.07 (-2.87, 11.51) | 2.88 (-7.29, 14.16) | 4.93 (-4.3, 15.04) |
| Algeria | 85 to 89 | 5.44 (-8.06, 20.92) | 3.65 (-14.48, 25.63) | 6.96 (-13.08, 31.62) |
| American Samoa | 25 to 29 | 1.34 (-21.59, 30.98) | 2.03 (-20.89, 31.59) | 1.95 (-20.92, 31.43) |
| American Samoa | 30 to 34 | 1.34 (-17.37, 24.29) | 1.41 (-16.64, 23.38) | 1.56 (-16.5, 23.52) |
| American Samoa | 35 to 39 | -0.64 (-20.36, 23.95) | 0.19 (-17.18, 21.2) | 0.28 (-17.08, 21.28) |
| American Samoa | 40 to 44 | -3.51 (-27.83, 29.01) | -2.16 (-20.7, 20.71) | -2.05 (-20.48, 20.65) |
| American Samoa | 45 to 49 | -6.38 (-32.04, 28.98) | -4.38 (-26.14, 23.79) | -4.49 (-25.76, 22.87) |
| American Samoa | 50 to 54 | -7.48 (-32.37, 26.55) | -7.22 (-30.43, 23.71) | -7.42 (-29.67, 21.87) |
| American Samoa | 55 to 59 | -5.13 (-32.14, 32.63) | -9.12 (-33.24, 23.73) | -8.86 (-31.53, 21.31) |
| American Samoa | 60 to 64 | -1.42 (-31.44, 41.74) | -9.27 (-35.71, 28.04) | -8.7 (-33.65, 25.63) |
| American Samoa | 65 to 69 | 1.56 (-31.17, 49.85) | -9.08 (-34.94, 27.06) | -8.28 (-33.11, 25.76) |
| American Samoa | 70 to 74 | 3.91 (-32.15, 59.12) | -5.74 (-36.97, 40.96) | -10.18 (-34.1, 22.43) |
| American Samoa | 75 to 79 | 3.24 (-34.81, 63.48) | -8.93 (-35.66, 28.9) | -11.9 (-35.52, 20.37) |
| American Samoa | 80 to 84 | -5.5 (-34.63, 36.61) | -11.7 (-37.61, 24.99) | -12.98 (-38.43, 23) |
| American Samoa | 85 to 89 | -10.96 (-40.43, 33.08) | -13.93 (-42.39, 28.6) | -12.73 (-40.11, 27.18) |
| Andorra | 25 to 29 | 1.7 (-20.48, 30.07) | 1.65 (-20.43, 29.84) | 1.69 (-20.43, 29.97) |
| Andorra | 30 to 34 | 1.03 (-16.56, 22.34) | 1.14 (-16.46, 22.44) | 0.8 (-16.74, 22.03) |
| Andorra | 35 to 39 | -0.45 (-16.37, 18.5) | -0.11 (-16.07, 18.88) | -0.78 (-16.63, 18.09) |
| Andorra | 40 to 44 | -1.62 (-16.97, 16.57) | -1.29 (-16.58, 16.82) | -2.08 (-17.26, 15.89) |
| Andorra | 45 to 49 | -2.25 (-17.47, 15.77) | -1.95 (-16.97, 15.79) | -2.7 (-17.69, 15.02) |
| Andorra | 50 to 54 | -2.86 (-18.21, 15.38) | -2.68 (-17.58, 14.9) | -3.19 (-18.01, 14.3) |
| Andorra | 55 to 59 | -3.76 (-19.68, 15.31) | -3.24 (-18.19, 14.43) | -3.51 (-18.55, 14.31) |
| Andorra | 60 to 64 | -3.89 (-22.11, 18.6) | -2.85 (-18.23, 15.43) | -2.99 (-18.82, 15.92) |
| Andorra | 65 to 69 | -3.95 (-26.69, 25.86) | -2.2 (-18.83, 17.85) | -2.37 (-19.87, 18.94) |
| Andorra | 70 to 74 | -3.34 (-31.16, 35.72) | -1.86 (-21.72, 23.04) | -2.2 (-23.23, 24.58) |
| Andorra | 75 to 79 | -2.14 (-33.68, 44.4) | -1.96 (-34.89, 47.63) | -1.87 (-34.48, 46.97) |
| Andorra | 80 to 84 | -1.3 (-34.97, 49.81) | -2.16 (-43.5, 69.42) | -1.63 (-41.98, 66.8) |
| Andorra | 85 to 89 | -1.79 (-41.04, 63.57) | -3.54 (-54.16, 102.96) | -1.81 (-51.01, 96.8) |
| Angola | 25 to 29 | 4.76 (-21.16, 39.19) | 4.88 (-26.08, 48.82) | 4.3 (-33.25, 62.98) |
| Angola | 30 to 34 | 4.65 (-18.92, 35.06) | 4.88 (-23.79, 44.35) | 4.18 (-29.24, 53.38) |
| Angola | 35 to 39 | 4.15 (-17.13, 30.89) | 4.49 (-21.95, 39.89) | 3.5 (-27.5, 47.74) |
| Angola | 40 to 44 | 3.64 (-15.14, 26.57) | 3.87 (-19.73, 34.4) | 3.18 (-24.88, 41.72) |
| Angola | 45 to 49 | 3.35 (-13.45, 23.42) | 3.35 (-17.8, 29.95) | 3.01 (-22.6, 37.11) |
| Angola | 50 to 54 | 2.88 (-11.83, 20.04) | 2.87 (-15.65, 25.46) | 2.96 (-20, 32.51) |
| Angola | 55 to 59 | 2.64 (-10.89, 18.22) | 2.74 (-14.21, 23.04) | 2.82 (-18.61, 29.88) |
| Angola | 60 to 64 | 2.62 (-10.59, 17.78) | 2.7 (-13.68, 22.19) | 2.8 (-18.22, 29.21) |
| Angola | 65 to 69 | 2.71 (-11.1, 18.67) | 2.87 (-14.13, 23.24) | 2.73 (-18.77, 29.92) |
| Angola | 70 to 74 | 3.03 (-12.28, 21.01) | 3.16 (-15.86, 26.48) | 2.82 (-20.58, 33.12) |
| Angola | 75 to 79 | 3.3 (-15.12, 25.72) | 3.93 (-20.83, 36.44) | 3.05 (-23.61, 39.01) |
| Angola | 80 to 84 | 3.28 (-21.21, 35.39) | 3.71 (-29.03, 51.56) | 3.05 (-30.53, 52.87) |
| Angola | 85 to 89 | 3.34 (-33.75, 61.2) | 3.36 (-44.93, 93.97) | 3.66 (-44.65, 94.14) |
| Antigua and Barbuda | 25 to 29 | 0.63 (-21.67, 29.28) | -0.58 (-22.08, 26.85) | -0.19 (-21.75, 27.31) |
| Antigua and Barbuda | 30 to 34 | 0.11 (-17.56, 21.57) | -0.6 (-17.95, 20.42) | -0.36 (-17.66, 20.59) |
| Antigua and Barbuda | 35 to 39 | -1.15 (-17.32, 18.18) | -1.15 (-17.02, 17.76) | -1.03 (-16.83, 17.78) |
| Antigua and Barbuda | 40 to 44 | -2.96 (-19.15, 16.49) | -2.34 (-17.65, 15.82) | -2.09 (-17.26, 15.86) |
| Antigua and Barbuda | 45 to 49 | -5.4 (-22.77, 15.88) | -3.52 (-18.97, 14.86) | -3.1 (-17.93, 14.41) |
| Antigua and Barbuda | 50 to 54 | -7.29 (-26.72, 17.29) | -4.51 (-20.33, 14.45) | -3.87 (-18.47, 13.34) |
| Antigua and Barbuda | 55 to 59 | -8.4 (-28.77, 17.79) | -4.98 (-21.27, 14.68) | -4.1 (-18.65, 13.07) |
| Antigua and Barbuda | 60 to 64 | -7.77 (-28.38, 18.78) | -4.49 (-20.14, 14.24) | -3.54 (-18.19, 13.73) |
| Antigua and Barbuda | 65 to 69 | -7.49 (-27.84, 18.6) | -3.45 (-18.33, 14.16) | -2.76 (-17.64, 14.81) |
| Antigua and Barbuda | 70 to 74 | -7.02 (-27.62, 19.45) | -2.53 (-17.65, 15.38) | -2.03 (-17.21, 15.93) |
| Antigua and Barbuda | 75 to 79 | -6.85 (-29.1, 22.38) | -1.69 (-17.4, 16.99) | -1.35 (-17.1, 17.39) |
| Antigua and Barbuda | 80 to 84 | -6.01 (-29.28, 24.92) | -1.1 (-18.28, 19.69) | -0.74 (-17.98, 20.12) |
| Antigua and Barbuda | 85 to 89 | -4.65 (-29.8, 29.5) | -0.89 (-22.32, 26.45) | -0.02 (-21.62, 27.52) |
| Argentina | 25 to 29 | 1.04 (-4.79, 7.23) | 0.92 (-7.34, 9.91) | 1.16 (-6.9, 9.91) |
| Argentina | 30 to 34 | 0.7 (-3.97, 5.61) | 0.53 (-6.09, 7.61) | 0.87 (-5.61, 7.8) |
| Argentina | 35 to 39 | 0.31 (-3.62, 4.41) | 0.05 (-5.56, 5.99) | 0.56 (-4.88, 6.31) |
| Argentina | 40 to 44 | -0.1 (-3.41, 3.31) | -0.32 (-5.02, 4.61) | 0.13 (-4.46, 4.93) |
| Argentina | 45 to 49 | -0.21 (-2.93, 2.59) | -0.38 (-4.23, 3.62) | -0.01 (-3.81, 3.93) |
| Argentina | 50 to 54 | -0.19 (-2.39, 2.07) | -0.52 (-3.64, 2.7) | 0.17 (-2.93, 3.36) |
| Argentina | 55 to 59 | -0.16 (-1.97, 1.68) | -0.67 (-3.23, 1.95) | 0.36 (-2.19, 2.98) |
| Argentina | 60 to 64 | -0.28 (-1.8, 1.27) | -0.92 (-3.07, 1.28) | 0.35 (-1.8, 2.55) |
| Argentina | 65 to 69 | -0.35 (-1.68, 1.01) | -1.06 (-2.95, 0.88) | 0.32 (-1.56, 2.23) |
| Argentina | 70 to 74 | -0.38 (-1.62, 0.87) | -1.07 (-2.84, 0.73) | 0.22 (-1.49, 1.97) |
| Argentina | 75 to 79 | -0.23 (-1.45, 1) | -0.81 (-2.61, 1.02) | 0.23 (-1.42, 1.91) |
| Argentina | 80 to 84 | -0.23 (-1.54, 1.09) | -0.71 (-2.73, 1.36) | 0.11 (-1.61, 1.86) |
| Argentina | 85 to 89 | 0.81 (-1.19, 2.86) | 0.5 (-2.68, 3.79) | 1.01 (-1.56, 3.65) |
| Armenia | 25 to 29 | -11.49 (-52.89, 66.3) | -0.15 (-22.59, 28.8) | -0.54 (-22.76, 28.08) |
| Armenia | 30 to 34 | -7.6 (-40.7, 43.97) | 0.28 (-17.99, 22.62) | 0.74 (-17.26, 22.65) |
| Armenia | 35 to 39 | -1.87 (-26.8, 31.56) | 0.04 (-18.71, 23.1) | 1.05 (-16.43, 22.19) |
| Armenia | 40 to 44 | -1.71 (-29.75, 37.53) | -2.32 (-23.41, 24.58) | -0.92 (-19.73, 22.31) |
| Armenia | 45 to 49 | -5.74 (-31.06, 28.9) | -5.99 (-27.77, 22.38) | -4.9 (-26.04, 22.28) |
| Armenia | 50 to 54 | -7.31 (-30.58, 23.77) | -9.52 (-32.11, 20.58) | -8.51 (-30.5, 20.44) |
| Armenia | 55 to 59 | -7.39 (-28.9, 20.64) | -9.52 (-32.45, 21.21) | -9.37 (-32.24, 21.22) |
| Armenia | 60 to 64 | -5.16 (-27.02, 23.24) | -7.05 (-31.74, 26.59) | -7.5 (-31.91, 25.64) |
| Armenia | 65 to 69 | -3.27 (-26.01, 26.46) | -5.32 (-30.73, 29.41) | -5.73 (-30.73, 28.29) |
| Armenia | 70 to 74 | -3.29 (-26.99, 28.1) | -9.26 (-31.41, 20.03) | -7.7 (-29.34, 20.57) |
| Armenia | 75 to 79 | -7.25 (-26.4, 16.88) | -12.59 (-33.68, 15.2) | -9.91 (-29.72, 15.48) |
| Armenia | 80 to 84 | -8.33 (-27.44, 15.82) | -13.86 (-35.74, 15.47) | -11.1 (-31.15, 14.77) |
| Armenia | 85 to 89 | -7.12 (-31.56, 26.05) | -11.81 (-37.46, 24.36) | -9.7 (-34.17, 23.86) |
| Australia | 25 to 29 | 3.58 (-27, 46.99) | 2.63 (-36.37, 65.53) | 4.28 (-37.47, 73.91) |
| Australia | 30 to 34 | 3.81 (-21.55, 37.36) | 3.3 (-29.17, 50.66) | 4.32 (-29.48, 54.33) |
| Australia | 35 to 39 | 4.16 (-16.88, 30.52) | 3.89 (-24.22, 42.43) | 4.13 (-23.9, 42.47) |
| Australia | 40 to 44 | 4.21 (-12.85, 24.61) | 4.32 (-19.4, 35.02) | 4.12 (-18.78, 33.49) |
| Australia | 45 to 49 | 4.16 (-9.15, 19.43) | 4.51 (-14.02, 27.05) | 3.98 (-14.12, 25.91) |
| Australia | 50 to 54 | 3.89 (-6.75, 15.74) | 4.26 (-10.77, 21.83) | 3.73 (-10.7, 20.48) |
| Australia | 55 to 59 | 3.42 (-5.16, 12.79) | 3.65 (-8.65, 17.6) | 3.3 (-8.3, 16.36) |
| Australia | 60 to 64 | 2.7 (-4.18, 10.07) | 2.72 (-7.21, 13.72) | 2.67 (-6.61, 12.87) |
| Australia | 65 to 69 | 2.05 (-3.53, 7.94) | 1.95 (-6.23, 10.83) | 2.12 (-5.36, 10.18) |
| Australia | 70 to 74 | 1.64 (-2.97, 6.48) | 1.34 (-5.55, 8.73) | 1.93 (-4.19, 8.44) |
| Australia | 75 to 79 | 1.56 (-2.47, 5.74) | 0.95 (-5.19, 7.5) | 1.99 (-3.26, 7.53) |
| Australia | 80 to 84 | 1.61 (-2.25, 5.62) | 0.74 (-5.38, 7.25) | 2.19 (-2.73, 7.37) |
| Australia | 85 to 89 | 1.73 (-3.31, 7.03) | 0.56 (-7.72, 9.59) | 2.42 (-3.84, 9.08) |
| Austria | 25 to 29 | -1.16 (-46.27, 81.84) | -10.2 (-52.43, 69.5) | -9.49 (-51.72, 69.69) |
| Austria | 30 to 34 | -5.06 (-43.22, 58.72) | -9.5 (-43.39, 44.68) | -7.8 (-40.56, 43.01) |
| Austria | 35 to 39 | -3.17 (-37.21, 49.31) | -6.92 (-36.45, 36.35) | -3.43 (-26.61, 27.08) |
| Austria | 40 to 44 | -1.01 (-29.94, 39.85) | -1.68 (-25.56, 29.86) | -3.57 (-27.04, 27.46) |
| Austria | 45 to 49 | 0.93 (-21.34, 29.5) | -0.28 (-22.61, 28.5) | -2.61 (-25.86, 27.93) |
| Austria | 50 to 54 | 2.05 (-14.52, 21.84) | 0.71 (-18.61, 24.62) | -0.81 (-21.77, 25.76) |
| Austria | 55 to 59 | 2.35 (-10.43, 16.95) | 1.81 (-14.29, 20.93) | 1.3 (-16.66, 23.13) |
| Austria | 60 to 64 | 2.37 (-7.61, 13.43) | 2.62 (-10.35, 17.46) | 2.26 (-12.52, 19.53) |
| Austria | 65 to 69 | 2.74 (-5.24, 11.4) | 2.73 (-8.06, 14.79) | 2.78 (-8.8, 15.84) |
| Austria | 70 to 74 | 3.64 (-2.99, 10.73) | 3.62 (-5.73, 13.9) | 3.25 (-5.96, 13.36) |
| Austria | 75 to 79 | 4.73 (-1.25, 11.08) | 4.56 (-4.43, 14.41) | 4.4 (-3.5, 12.94) |
| Austria | 80 to 84 | 5.9 (0.47, 11.62) | 5.37 (-3.4, 14.93) | 5.97 (-0.87, 13.27) |
| Austria | 85 to 89 | 7.51 (0.54, 14.97) | 6.87 (-5.39, 20.72) | 7.84 (-0.48, 16.85) |
| Azerbaijan | 25 to 29 | 5.3 (-22.11, 42.37) | 6.45 (-30.8, 63.76) | 6.74 (-32.79, 69.54) |
| Azerbaijan | 30 to 34 | 5.28 (-18.37, 35.77) | 6.2 (-26.69, 53.87) | 6.58 (-27.1, 55.82) |
| Azerbaijan | 35 to 39 | 5.49 (-16.52, 33.3) | 6.07 (-25.08, 50.18) | 6.47 (-24.47, 50.07) |
| Azerbaijan | 40 to 44 | 5.66 (-15.35, 31.87) | 6.08 (-23.57, 47.24) | 6.35 (-22.69, 46.29) |
| Azerbaijan | 45 to 49 | 5.87 (-14.45, 31.02) | 6.01 (-22.32, 44.67) | 6.31 (-21.23, 43.49) |
| Azerbaijan | 50 to 54 | 5.99 (-12.94, 29.03) | 5.71 (-19.86, 39.45) | 5.8 (-19.07, 38.3) |
| Azerbaijan | 55 to 59 | 5.84 (-11.46, 26.51) | 5.34 (-17.99, 35.31) | 5.51 (-17.11, 34.31) |
| Azerbaijan | 60 to 64 | 5.61 (-11.23, 25.64) | 5.31 (-17.63, 34.64) | 5.23 (-16.8, 33.08) |
| Azerbaijan | 65 to 69 | 5.77 (-12.03, 27.18) | 6.07 (-19.03, 38.95) | 5.62 (-17.57, 35.33) |
| Azerbaijan | 70 to 74 | 6.73 (-15.69, 35.11) | 7.04 (-25.48, 53.76) | 6.25 (-20.29, 41.61) |
| Azerbaijan | 75 to 79 | 7.98 (-20.34, 46.38) | 2.16 (-28.95, 46.9) | 8.42 (-26.18, 59.24) |
| Azerbaijan | 80 to 84 | 8.78 (-25.5, 58.84) | -2.24 (-33.81, 44.39) | 8.97 (-32.09, 74.88) |
| Azerbaijan | 85 to 89 | 9.25 (-41.49, 103.96) | -5.16 (-37.65, 44.25) | 8.41 (-43.78, 109.04) |
| Bahamas | 25 to 29 | -7.65 (-52.44, 79.29) | -8.04 (-49.51, 67.51) | -8.84 (-49.98, 66.17) |
| Bahamas | 30 to 34 | -7.05 (-42.45, 50.13) | -3.55 (-29.3, 31.59) | -4.35 (-30.65, 31.94) |
| Bahamas | 35 to 39 | -4.51 (-37.04, 44.82) | -5.58 (-33.76, 34.58) | -6.62 (-34.63, 33.4) |
| Bahamas | 40 to 44 | -3.17 (-35.69, 45.81) | -7.35 (-34.68, 31.39) | -8.47 (-37.86, 34.82) |
| Bahamas | 45 to 49 | -1.19 (-32.76, 45.18) | -6.5 (-36.62, 37.94) | -11.28 (-39.02, 29.08) |
| Bahamas | 50 to 54 | 0.59 (-30.45, 45.49) | -4.04 (-36.74, 45.57) | -10.6 (-36.17, 25.23) |
| Bahamas | 55 to 59 | 0.32 (-30.32, 44.44) | -1.03 (-36.06, 53.2) | -8.27 (-38.2, 36.16) |
| Bahamas | 60 to 64 | -0.3 (-31.78, 45.72) | 0.6 (-35.97, 58.07) | -9.6 (-39.32, 34.69) |
| Bahamas | 65 to 69 | 0.71 (-33.89, 53.42) | 0.67 (-37.29, 61.62) | -10.8 (-39.58, 31.69) |
| Bahamas | 70 to 74 | 1.46 (-34.81, 57.92) | -7.6 (-41.39, 45.66) | -10.88 (-39.38, 31.02) |
| Bahamas | 75 to 79 | 1.63 (-36.3, 62.14) | -11.15 (-43.31, 39.27) | -11.16 (-38.6, 28.53) |
| Bahamas | 80 to 84 | 1.34 (-40.48, 72.53) | -12.4 (-42.75, 34.04) | -13.33 (-41.34, 28.07) |
| Bahamas | 85 to 89 | 0.64 (-51.17, 107.45) | -11.83 (-42.88, 36.09) | -11.6 (-43.38, 38.02) |
| Bahrain | 25 to 29 | 2.09 (-36.26, 63.5) | 1.88 (-40.96, 75.79) | -9.67 (-44.36, 46.62) |
| Bahrain | 30 to 34 | 1.44 (-30.94, 48.99) | 0.58 (-35.92, 57.87) | -9.62 (-39.66, 35.37) |
| Bahrain | 35 to 39 | 1.13 (-27.33, 40.72) | -0.07 (-33.17, 49.41) | -9.6 (-35.87, 27.42) |
| Bahrain | 40 to 44 | 1.01 (-25.84, 37.57) | 0 (-32, 47.06) | -9.08 (-33.72, 24.73) |
| Bahrain | 45 to 49 | 0.43 (-25.47, 35.33) | -0.13 (-32.13, 46.96) | -8.01 (-31.55, 23.61) |
| Bahrain | 50 to 54 | -0.22 (-24.49, 31.84) | -0.28 (-32.27, 46.84) | -7.2 (-30.75, 24.36) |
| Bahrain | 55 to 59 | -0.16 (-22.79, 29.11) | -0.17 (-31.14, 44.73) | -4.56 (-30, 30.12) |
| Bahrain | 60 to 64 | 0.36 (-21.28, 27.95) | 0.58 (-30.49, 45.54) | -2 (-28.33, 34.02) |
| Bahrain | 65 to 69 | 0.68 (-20.69, 27.82) | 0.77 (-30.41, 45.91) | -0.52 (-28.01, 37.46) |
| Bahrain | 70 to 74 | 1.47 (-20.85, 30.07) | 1.63 (-31.6, 51.02) | 0.68 (-28.99, 42.75) |
| Bahrain | 75 to 79 | 2.1 (-23.47, 36.21) | -2.58 (-34.4, 44.67) | 1.77 (-32.64, 53.74) |
| Bahrain | 80 to 84 | 2.31 (-34.69, 60.26) | -6.82 (-38.03, 40.09) | -4.52 (-34.91, 40.07) |
| Bahrain | 85 to 89 | 0.97 (-47.7, 94.93) | -10.43 (-41.73, 37.67) | -8.12 (-38.78, 37.89) |
| Bangladesh | 25 to 29 | 3.7 (-20.3, 34.93) | 3.78 (-27.27, 48.1) | 3.23 (-31.85, 56.38) |
| Bangladesh | 30 to 34 | 3.27 (-16.45, 27.63) | 3.73 (-22.14, 38.19) | 3.01 (-25.02, 41.5) |
| Bangladesh | 35 to 39 | 2.95 (-13.68, 22.77) | 3.33 (-18.93, 31.7) | 2.57 (-20.87, 32.95) |
| Bangladesh | 40 to 44 | 2.49 (-12.05, 19.44) | 2.72 (-16.51, 26.37) | 2.54 (-18.56, 29.11) |
| Bangladesh | 45 to 49 | 2.43 (-10.23, 16.86) | 2.15 (-14.32, 21.8) | 3 (-16.05, 26.36) |
| Bangladesh | 50 to 54 | 2.14 (-8.62, 14.17) | 1.65 (-12.09, 17.54) | 3.13 (-13.74, 23.3) |
| Bangladesh | 55 to 59 | 1.96 (-7.54, 12.43) | 1.55 (-10.27, 14.93) | 2.98 (-12.49, 21.18) |
| Bangladesh | 60 to 64 | 1.74 (-6.99, 11.29) | 1.27 (-9.44, 13.25) | 2.67 (-11.85, 19.57) |
| Bangladesh | 65 to 69 | 1.77 (-7.34, 11.78) | 1.21 (-10.03, 13.85) | 2.57 (-12.32, 19.99) |
| Bangladesh | 70 to 74 | 1.59 (-8.57, 12.87) | 1.02 (-12.17, 16.2) | 2.28 (-13.25, 20.58) |
| Bangladesh | 75 to 79 | 1.55 (-9.77, 14.3) | 0.99 (-14.64, 19.49) | 1.98 (-13.82, 20.67) |
| Bangladesh | 80 to 84 | 1.67 (-12.6, 18.28) | 1.64 (-19.82, 28.86) | 1.64 (-16.66, 23.96) |
| Bangladesh | 85 to 89 | 2.44 (-18.62, 28.95) | 2.59 (-29.39, 49.05) | 2.11 (-23.82, 36.86) |
| Barbados | 25 to 29 | 0.08 (-52.75, 111.98) | 4.53 (-34.44, 66.69) | -6.74 (-48.9, 70.2) |
| Barbados | 30 to 34 | 0.44 (-43.5, 78.57) | 4.48 (-35.81, 70.04) | -2.87 (-29.86, 34.52) |
| Barbados | 35 to 39 | 0.44 (-37.86, 62.37) | 3.07 (-35.91, 65.77) | -2.71 (-33.89, 43.19) |
| Barbados | 40 to 44 | 0.53 (-32.07, 48.77) | 1.27 (-34.76, 57.19) | -2.12 (-35.43, 48.37) |
| Barbados | 45 to 49 | 0.49 (-27.13, 38.57) | -0.79 (-34.1, 49.34) | -1.28 (-34.82, 49.51) |
| Barbados | 50 to 54 | 0.35 (-24.12, 32.71) | -0.32 (-31.13, 44.27) | -0.29 (-33.28, 49.02) |
| Barbados | 55 to 59 | 0.31 (-21.92, 28.87) | 0.03 (-28.46, 39.88) | 1.26 (-30.98, 48.57) |
| Barbados | 60 to 64 | 0.26 (-20.91, 27.09) | 0.12 (-27.18, 37.64) | 0.02 (-30.87, 44.71) |
| Barbados | 65 to 69 | 0.5 (-20.33, 26.78) | -0.11 (-26.64, 36.02) | 0.46 (-29.75, 43.66) |
| Barbados | 70 to 74 | 0.86 (-19.87, 26.95) | -0.52 (-27.04, 35.66) | 1.28 (-27.24, 40.97) |
| Barbados | 75 to 79 | 1.15 (-19.51, 27.11) | -0.48 (-28.35, 38.24) | 1.69 (-26.11, 39.96) |
| Barbados | 80 to 84 | 1.29 (-20.7, 29.39) | -0.41 (-30.77, 43.25) | 2.06 (-27.25, 43.19) |
| Barbados | 85 to 89 | 1.9 (-28.21, 44.65) | -0.38 (-40.67, 67.29) | 3.4 (-36.24, 67.7) |
| Belarus | 25 to 29 | -4.07 (-31.77, 34.87) | -0.49 (-22.9, 28.44) | 0.1 (-22.25, 28.86) |
| Belarus | 30 to 34 | -6.3 (-39.53, 45.19) | 0.08 (-17.9, 22) | 0.3 (-17.54, 22.01) |
| Belarus | 35 to 39 | -0.48 (-23.29, 29.09) | 1.44 (-16.39, 23.07) | 0.75 (-16.47, 21.51) |
| Belarus | 40 to 44 | -2.15 (-24.86, 27.43) | 0.64 (-18.17, 23.77) | 0.31 (-18.14, 22.91) |
| Belarus | 45 to 49 | -4.53 (-27.58, 25.86) | -2.92 (-23.73, 23.56) | -2.08 (-22.39, 23.55) |
| Belarus | 50 to 54 | -4.88 (-28.51, 26.56) | -6.54 (-28.63, 22.38) | -4.6 (-26.17, 23.27) |
| Belarus | 55 to 59 | -2.99 (-29.49, 33.46) | -8.81 (-32.25, 22.76) | -4.89 (-29.04, 27.49) |
| Belarus | 60 to 64 | 0.88 (-27.84, 41.02) | -7.77 (-33.56, 28.03) | -2.12 (-30.06, 36.97) |
| Belarus | 65 to 69 | 4.93 (-26.5, 49.79) | -3.01 (-34.39, 43.37) | 1.54 (-29.93, 47.13) |
| Belarus | 70 to 74 | 6.87 (-27.51, 57.54) | -5.95 (-33.37, 32.76) | 5.08 (-30.71, 59.36) |
| Belarus | 75 to 79 | 5.75 (-31.36, 62.9) | -9.77 (-34.52, 24.33) | 5.03 (-32.94, 64.48) |
| Belarus | 80 to 84 | 4.56 (-36.51, 72.21) | -11.07 (-35.96, 23.49) | 3.57 (-37.94, 72.84) |
| Belarus | 85 to 89 | 3.49 (-47.39, 103.58) | -9.96 (-38.05, 30.87) | 1.95 (-48.74, 102.76) |
| Belgium | 25 to 29 | 2.69 (-31.04, 52.93) | 2.82 (-41.86, 81.85) | 2.9 (-41.71, 81.64) |
| Belgium | 30 to 34 | 2.49 (-25.22, 40.46) | 2.63 (-35.04, 62.13) | 3.01 (-34.48, 61.94) |
| Belgium | 35 to 39 | 2.06 (-22.24, 33.97) | 2.04 (-31.41, 51.8) | 2.84 (-30.33, 51.79) |
| Belgium | 40 to 44 | 1.56 (-19.44, 28.04) | 1.37 (-27.62, 41.95) | 2.61 (-26.03, 42.33) |
| Belgium | 45 to 49 | 1.25 (-15.94, 21.95) | 0.7 (-22.64, 31.08) | 2.03 (-21.9, 33.3) |
| Belgium | 50 to 54 | 1.32 (-12.21, 16.94) | 0.7 (-17.79, 23.35) | 1.92 (-17.02, 25.19) |
| Belgium | 55 to 59 | 1.28 (-9.41, 13.23) | 0.9 (-13.71, 17.99) | 1.66 (-13.37, 19.29) |
| Belgium | 60 to 64 | 1.01 (-7.38, 10.15) | 0.82 (-10.69, 13.82) | 1.24 (-10.5, 14.51) |
| Belgium | 65 to 69 | 0.61 (-6.05, 7.73) | 0.44 (-8.81, 10.63) | 0.67 (-8.66, 10.94) |
| Belgium | 70 to 74 | 0.42 (-4.9, 6.04) | 0.28 (-7.29, 8.45) | 0.5 (-6.86, 8.45) |
| Belgium | 75 to 79 | 0.34 (-4.27, 5.17) | 0.07 (-6.8, 7.44) | 0.52 (-5.63, 7.08) |
| Belgium | 80 to 84 | 0.78 (-3.04, 4.75) | 0.48 (-5.61, 6.96) | 0.93 (-3.97, 6.07) |
| Belgium | 85 to 89 | 1 (-3.26, 5.45) | 0.69 (-6.83, 8.82) | 1.12 (-4.02, 6.52) |
| Belize | 25 to 29 | -7.04 (-43.54, 53.07) | -14.02 (-54.37, 62) | -12.42 (-53.14, 63.68) |
| Belize | 30 to 34 | -7.66 (-36.18, 33.6) | -11.12 (-43.02, 38.63) | -11.42 (-40.79, 32.52) |
| Belize | 35 to 39 | -7.61 (-33.13, 27.64) | -7.59 (-32.13, 25.84) | -11.87 (-38.53, 26.34) |
| Belize | 40 to 44 | -7.17 (-31.64, 26.05) | -9.13 (-35.2, 27.43) | -11.66 (-37.18, 24.23) |
| Belize | 45 to 49 | -4.12 (-30.18, 31.65) | -9.73 (-33.59, 22.71) | -10.15 (-35.71, 25.58) |
| Belize | 50 to 54 | -1.69 (-29.94, 37.94) | -9.78 (-32.14, 19.95) | -10.12 (-33.47, 21.43) |
| Belize | 55 to 59 | -0.2 (-29.27, 40.82) | -7.89 (-30.09, 21.36) | -10.48 (-34.21, 21.81) |
| Belize | 60 to 64 | 0.1 (-29.57, 42.29) | -4.56 (-32.53, 35.01) | -10.87 (-34.28, 20.88) |
| Belize | 65 to 69 | -0.04 (-30.16, 43.07) | -2.2 (-34.85, 46.82) | -10.18 (-36.6, 27.24) |
| Belize | 70 to 74 | -0.83 (-32.02, 44.67) | -0.71 (-36.71, 55.75) | -10.67 (-37.82, 28.33) |
| Belize | 75 to 79 | 0.56 (-35.05, 55.68) | -0.78 (-39.25, 62.06) | -9.6 (-36.25, 28.19) |
| Belize | 80 to 84 | 1.63 (-38.93, 69.11) | -6 (-38.05, 42.61) | -10.75 (-36.57, 25.58) |
| Belize | 85 to 89 | 3.23 (-48.17, 105.6) | -11.82 (-43.78, 38.31) | -10.76 (-42.29, 38) |
| Benin | 25 to 29 | -7.87 (-41.65, 45.47) | -11.71 (-45.54, 43.12) | -12.88 (-53.64, 63.71) |
| Benin | 30 to 34 | -9.81 (-40.3, 36.24) | -12.44 (-42.14, 32.51) | -10.9 (-42.53, 38.15) |
| Benin | 35 to 39 | -8.49 (-35.17, 29.15) | -11.24 (-40.28, 31.94) | -6.93 (-29.69, 23.2) |
| Benin | 40 to 44 | -5.23 (-32.58, 33.22) | -8.16 (-31.32, 22.82) | -7.75 (-33.54, 28.04) |
| Benin | 45 to 49 | -1.88 (-29.24, 36.08) | -7.55 (-31.44, 24.66) | -7.83 (-32.7, 26.22) |
| Benin | 50 to 54 | 0.66 (-25.25, 35.53) | -5.31 (-30.62, 29.23) | -4.79 (-31.14, 31.64) |
| Benin | 55 to 59 | 1.02 (-23.72, 33.8) | -2.55 (-29.61, 34.9) | -1.73 (-30.63, 39.21) |
| Benin | 60 to 64 | 1.6 (-21.72, 31.86) | 0.05 (-27.98, 38.98) | 0.44 (-29.9, 43.91) |
| Benin | 65 to 69 | 1.38 (-21.04, 30.17) | 2.22 (-26.13, 41.46) | 1.94 (-29.18, 46.73) |
| Benin | 70 to 74 | 1.54 (-20.25, 29.28) | 1.86 (-25.52, 39.32) | 2.79 (-27.88, 46.51) |
| Benin | 75 to 79 | 2.24 (-20.86, 32.09) | 1.97 (-26.74, 41.93) | 1.92 (-30, 48.41) |
| Benin | 80 to 84 | 2.93 (-25, 41.26) | 4.5 (-34.6, 66.96) | 3.02 (-36.38, 66.84) |
| Benin | 85 to 89 | 3.92 (-34.69, 65.35) | 4.97 (-45.84, 103.47) | 2.93 (-46.92, 99.62) |
| Bermuda | 25 to 29 | 2.53 (-19.94, 31.32) | 1.89 (-20.12, 29.96) | 1.94 (-20.18, 30.19) |
| Bermuda | 30 to 34 | 2.07 (-15.8, 23.73) | 1.68 (-15.98, 23.05) | 1.65 (-16.01, 23.02) |
| Bermuda | 35 to 39 | 1.3 (-14.97, 20.68) | 1.13 (-15.02, 20.34) | 1.3 (-14.88, 20.55) |
| Bermuda | 40 to 44 | 0.11 (-15.68, 18.85) | 0.21 (-15.32, 18.58) | 0.53 (-15.05, 18.96) |
| Bermuda | 45 to 49 | -1.07 (-17.14, 18.13) | -0.84 (-16.01, 17.08) | -0.58 (-15.8, 17.4) |
| Bermuda | 50 to 54 | -2.7 (-19.45, 17.53) | -1.87 (-16.77, 15.7) | -1.58 (-16.63, 16.18) |
| Bermuda | 55 to 59 | -4.56 (-22.73, 17.88) | -2.51 (-17.31, 14.94) | -2.43 (-17.34, 15.16) |
| Bermuda | 60 to 64 | -5.24 (-24.55, 19.03) | -2.66 (-17.44, 14.78) | -2.74 (-17.75, 15.02) |
| Bermuda | 65 to 69 | -5.48 (-26.59, 21.7) | -2.74 (-17.62, 14.84) | -2.88 (-18.41, 15.61) |
| Bermuda | 70 to 74 | -8.39 (-29.82, 19.57) | -3.15 (-18.16, 14.6) | -3.22 (-19.78, 16.77) |
| Bermuda | 75 to 79 | -9.67 (-33.65, 22.96) | -3.66 (-19.05, 14.64) | -3.68 (-21.8, 18.63) |
| Bermuda | 80 to 84 | -10.31 (-35.76, 25.21) | -4.03 (-20.69, 16.14) | -3.95 (-23.26, 20.22) |
| Bermuda | 85 to 89 | -10.31 (-37.58, 28.89) | -3.98 (-24.72, 22.48) | -4.28 (-26.97, 25.46) |
| Bhutan | 25 to 29 | -1.84 (-23.37, 25.76) | -2.24 (-23.35, 24.7) | -2.2 (-23.33, 24.74) |
| Bhutan | 30 to 34 | -2.17 (-19.3, 18.59) | -2.46 (-19.4, 18.05) | -2.4 (-19.35, 18.11) |
| Bhutan | 35 to 39 | -2.3 (-18, 16.41) | -2.39 (-17.97, 16.16) | -2.31 (-17.91, 16.26) |
| Bhutan | 40 to 44 | -2.33 (-17.86, 16.13) | -2.27 (-17.41, 15.64) | -2.2 (-17.35, 15.72) |
| Bhutan | 45 to 49 | -2.44 (-18.5, 16.77) | -2.23 (-17.19, 15.44) | -2.21 (-17.18, 15.46) |
| Bhutan | 50 to 54 | -3.54 (-20.58, 17.15) | -2.41 (-17.23, 15.07) | -2.51 (-17.32, 14.94) |
| Bhutan | 55 to 59 | -4.49 (-24.36, 20.6) | -2.34 (-17.17, 15.13) | -2.47 (-17.27, 14.99) |
| Bhutan | 60 to 64 | -6.74 (-28.75, 22.06) | -2.33 (-17.17, 15.16) | -2.46 (-17.27, 15.01) |
| Bhutan | 65 to 69 | -11.25 (-34.09, 19.51) | -2.62 (-17.53, 14.97) | -2.73 (-17.62, 14.85) |
| Bhutan | 70 to 74 | -13.66 (-37.3, 18.91) | -3.12 (-18.13, 14.64) | -3.23 (-18.23, 14.51) |
| Bhutan | 75 to 79 | -13.99 (-37.57, 18.48) | -4.19 (-19.49, 14.02) | -4.03 (-19.35, 14.21) |
| Bhutan | 80 to 84 | -13.4 (-36.38, 17.88) | -5.99 (-22.31, 13.77) | -5.16 (-21.63, 14.77) |
| Bhutan | 85 to 89 | -10.51 (-34.25, 21.8) | -6.88 (-26.99, 18.78) | -5.5 (-25.91, 20.54) |
| Bolivia (Plurinational State of) | 25 to 29 | 2.01 (-14.91, 22.29) | 2.62 (-21.63, 34.37) | 1.91 (-19.87, 29.62) |
| Bolivia (Plurinational State of) | 30 to 34 | 2.05 (-11.7, 17.94) | 2.2 (-17.4, 26.45) | 2.02 (-16.09, 24.02) |
| Bolivia (Plurinational State of) | 35 to 39 | 2.08 (-9.14, 14.68) | 1.7 (-14.23, 20.59) | 2.33 (-12.83, 20.13) |
| Bolivia (Plurinational State of) | 40 to 44 | 2.06 (-6.95, 11.94) | 1.51 (-11.13, 15.95) | 2.55 (-9.8, 16.6) |
| Bolivia (Plurinational State of) | 45 to 49 | 2 (-5.27, 9.82) | 1.42 (-8.82, 12.82) | 2.51 (-7.5, 13.61) |
| Bolivia (Plurinational State of) | 50 to 54 | 1.82 (-4.16, 8.17) | 1.31 (-7.21, 10.61) | 2.27 (-5.93, 11.19) |
| Bolivia (Plurinational State of) | 55 to 59 | 1.73 (-3.45, 7.19) | 1.25 (-6.3, 9.4) | 2.13 (-4.88, 9.65) |
| Bolivia (Plurinational State of) | 60 to 64 | 1.7 (-3.01, 6.65) | 1.28 (-5.75, 8.83) | 2.05 (-4.22, 8.72) |
| Bolivia (Plurinational State of) | 65 to 69 | 1.81 (-2.74, 6.58) | 1.46 (-5.43, 8.85) | 2.07 (-3.92, 8.43) |
| Bolivia (Plurinational State of) | 70 to 74 | 2.08 (-2.71, 7.11) | 1.78 (-5.5, 9.62) | 2.3 (-3.96, 8.96) |
| Bolivia (Plurinational State of) | 75 to 79 | 2.36 (-3.26, 8.32) | 2.01 (-6.49, 11.28) | 2.57 (-4.76, 10.46) |
| Bolivia (Plurinational State of) | 80 to 84 | 2.51 (-4.89, 10.5) | 2.24 (-9.15, 15.06) | 2.65 (-6.84, 13.12) |
| Bolivia (Plurinational State of) | 85 to 89 | 2.54 (-9.44, 16.11) | 2.36 (-16.15, 24.95) | 2.47 (-12.27, 19.7) |
| Bosnia and Herzegovina | 25 to 29 | 3.6 (-42.99, 88.26) | 1.49 (-52.19, 115.46) | -2.54 (-53.08, 102.46) |
| Bosnia and Herzegovina | 30 to 34 | 3.47 (-37.33, 70.81) | 1.22 (-43.81, 82.33) | -4.78 (-43.39, 60.18) |
| Bosnia and Herzegovina | 35 to 39 | 3.4 (-31.21, 55.41) | 1.21 (-39.71, 69.91) | -3.6 (-39.41, 53.38) |
| Bosnia and Herzegovina | 40 to 44 | 2.54 (-28.08, 46.19) | 1.6 (-36.1, 61.53) | -1.99 (-35.67, 49.31) |
| Bosnia and Herzegovina | 45 to 49 | 2.1 (-23.74, 36.7) | 2.23 (-31.43, 52.42) | 0.1 (-30.18, 43.51) |
| Bosnia and Herzegovina | 50 to 54 | 1.58 (-18.8, 27.07) | 1.66 (-27.34, 42.23) | 1.11 (-23.7, 34) |
| Bosnia and Herzegovina | 55 to 59 | 1.29 (-14.92, 20.6) | 1.98 (-21.43, 32.37) | 0.63 (-19.82, 26.3) |
| Bosnia and Herzegovina | 60 to 64 | 1.51 (-11.82, 16.84) | 2.28 (-17.48, 26.76) | 0.6 (-16.5, 21.2) |
| Bosnia and Herzegovina | 65 to 69 | 1.78 (-10.24, 15.42) | 2.56 (-15.58, 24.59) | 1.05 (-14.15, 18.95) |
| Bosnia and Herzegovina | 70 to 74 | 2.56 (-9.58, 16.32) | 2.8 (-16.1, 25.95) | 2.16 (-13.13, 20.15) |
| Bosnia and Herzegovina | 75 to 79 | 3.35 (-11.18, 20.26) | 3.24 (-19.39, 32.21) | 3.29 (-14.69, 25.06) |
| Bosnia and Herzegovina | 80 to 84 | 3.28 (-13.08, 22.7) | 2.98 (-20.61, 33.57) | 3.25 (-17.31, 28.92) |
| Bosnia and Herzegovina | 85 to 89 | 3.49 (-20.6, 34.88) | 3.53 (-29.35, 51.71) | 3.57 (-28.25, 49.48) |
| Botswana | 25 to 29 | -13.08 (-53.86, 63.75) | -2.84 (-24.94, 25.77) | -1.78 (-23.83, 26.64) |
| Botswana | 30 to 34 | -11.25 (-43.33, 38.98) | -5.05 (-24.22, 18.96) | -3.31 (-20.84, 18.1) |
| Botswana | 35 to 39 | -7.37 (-32.59, 27.29) | -7.17 (-30.46, 23.91) | -4.61 (-23.09, 18.31) |
| Botswana | 40 to 44 | -6.77 (-31.94, 27.72) | -7.49 (-34.1, 29.86) | -6.53 (-27.6, 20.66) |
| Botswana | 45 to 49 | -4.49 (-32.68, 35.52) | -8.1 (-34.56, 29.07) | -8.58 (-30.4, 20.09) |
| Botswana | 50 to 54 | -1.51 (-31.06, 40.7) | -6.39 (-35.73, 36.34) | -9.78 (-31.27, 18.43) |
| Botswana | 55 to 59 | 0.25 (-30.14, 43.85) | -3.46 (-35.49, 44.48) | -10.31 (-32.47, 19.14) |
| Botswana | 60 to 64 | 1.88 (-28.82, 45.82) | -0.19 (-35.3, 53.95) | -10.4 (-32.84, 19.55) |
| Botswana | 65 to 69 | 1.59 (-28.58, 44.48) | 1.65 (-35.45, 60.08) | -9.95 (-32.09, 19.41) |
| Botswana | 70 to 74 | 1.31 (-30.67, 48.04) | -0.57 (-36.85, 56.54) | -9.17 (-33.08, 23.27) |
| Botswana | 75 to 79 | 1.87 (-34.49, 58.41) | -5.2 (-40.68, 51.49) | -11.11 (-34.64, 20.88) |
| Botswana | 80 to 84 | -4.1 (-36.43, 44.66) | -9.61 (-42.01, 40.89) | -13.08 (-37.42, 20.73) |
| Botswana | 85 to 89 | -9.97 (-41.44, 38.43) | -10.5 (-42.48, 39.26) | -14.28 (-41.83, 26.31) |
| Brazil | 25 to 29 | 0.79 (-2.85, 4.57) | 0.81 (-4.43, 6.35) | 0.78 (-4.21, 6.03) |
| Brazil | 30 to 34 | 0.58 (-2.16, 3.4) | 0.73 (-3.2, 4.81) | 0.45 (-3.34, 4.39) |
| Brazil | 35 to 39 | 0.46 (-1.82, 2.79) | 0.58 (-2.64, 3.91) | 0.34 (-2.84, 3.64) |
| Brazil | 40 to 44 | 0.37 (-1.58, 2.36) | 0.47 (-2.24, 3.26) | 0.28 (-2.51, 3.15) |
| Brazil | 45 to 49 | 0.51 (-1.17, 2.21) | 0.54 (-1.76, 2.9) | 0.48 (-1.94, 2.96) |
| Brazil | 50 to 54 | 0.71 (-0.74, 2.18) | 0.7 (-1.28, 2.73) | 0.73 (-1.38, 2.89) |
| Brazil | 55 to 59 | 0.9 (-0.41, 2.23) | 0.83 (-0.97, 2.67) | 1 (-0.9, 2.93) |
| Brazil | 60 to 64 | 1.24 (0.02, 2.48) | 1.07 (-0.63, 2.81) | 1.43 (-0.31, 3.2) |
| Brazil | 65 to 69 | 1.48 (0.29, 2.69) | 1.19 (-0.5, 2.91) | 1.77 (0.08, 3.49) |
| Brazil | 70 to 74 | 1.81 (0.57, 3.06) | 1.41 (-0.36, 3.21) | 2.18 (0.44, 3.95) |
| Brazil | 75 to 79 | 2.13 (0.77, 3.51) | 1.73 (-0.25, 3.75) | 2.49 (0.62, 4.4) |
| Brazil | 80 to 84 | 2.57 (0.85, 4.32) | 2.16 (-0.4, 4.77) | 2.93 (0.6, 5.31) |
| Brazil | 85 to 89 | 2.97 (0.22, 5.78) | 2.66 (-1.5, 6.99) | 3.2 (-0.42, 6.95) |
| Brunei Darussalam | 25 to 29 | -2.1 (-24.31, 26.62) | -0.59 (-22.8, 28.01) | -0.1 (-22.44, 28.69) |
| Brunei Darussalam | 30 to 34 | -2.03 (-19.99, 19.95) | -1.5 (-18.95, 19.71) | -1.08 (-18.61, 20.23) |
| Brunei Darussalam | 35 to 39 | -2.28 (-21.69, 21.93) | -2.41 (-18.88, 17.4) | -2.34 (-18.83, 17.49) |
| Brunei Darussalam | 40 to 44 | -4.69 (-27.45, 25.21) | -3.79 (-21.06, 17.26) | -4.22 (-21.43, 16.76) |
| Brunei Darussalam | 45 to 49 | -8.97 (-32.86, 23.42) | -6.28 (-25.3, 17.58) | -6.91 (-25.82, 16.82) |
| Brunei Darussalam | 50 to 54 | -12.04 (-35.81, 20.53) | -8.31 (-28.16, 17.03) | -8.42 (-28.51, 17.32) |
| Brunei Darussalam | 55 to 59 | -14.08 (-37.73, 18.56) | -9.8 (-31.01, 17.95) | -10.03 (-31.22, 17.68) |
| Brunei Darussalam | 60 to 64 | -13.36 (-38.08, 21.23) | -10.18 (-31.58, 17.91) | -10.02 (-32.15, 19.32) |
| Brunei Darussalam | 65 to 69 | -11.81 (-36.98, 23.41) | -11.38 (-32.63, 16.57) | -12.57 (-34.26, 16.27) |
| Brunei Darussalam | 70 to 74 | -6.5 (-37.84, 40.66) | -10.33 (-32.36, 18.87) | -13.13 (-35.69, 17.34) |
| Brunei Darussalam | 75 to 79 | -2.86 (-39.27, 55.38) | -9.35 (-32.06, 20.95) | -12.58 (-35.87, 19.17) |
| Brunei Darussalam | 80 to 84 | -0.74 (-42.93, 72.65) | -8.88 (-31.88, 21.88) | -10.52 (-33.77, 20.89) |
| Brunei Darussalam | 85 to 89 | -6.91 (-44.63, 56.51) | -7.51 (-31.74, 25.33) | -7.71 (-32.4, 25.99) |
| Bulgaria | 25 to 29 | 4.86 (-16.73, 32.04) | 5 (-22.27, 41.83) | 4.41 (-27.47, 50.3) |
| Bulgaria | 30 to 34 | 4.46 (-11.74, 23.64) | 4.61 (-15.97, 30.24) | 3.7 (-21.42, 36.86) |
| Bulgaria | 35 to 39 | 4.54 (-8.59, 19.56) | 4.77 (-11.96, 24.67) | 4.15 (-15.64, 28.6) |
| Bulgaria | 40 to 44 | 4.66 (-6.03, 16.56) | 4.69 (-9.26, 20.77) | 4.49 (-11.25, 23.02) |
| Bulgaria | 45 to 49 | 5.17 (-3.88, 15.07) | 5.04 (-7.03, 18.67) | 5.19 (-7.9, 20.15) |
| Bulgaria | 50 to 54 | 5.51 (-2.24, 13.86) | 5.44 (-5.04, 17.08) | 5.45 (-5.66, 17.87) |
| Bulgaria | 55 to 59 | 5.45 (-1.17, 12.51) | 5.47 (-3.61, 15.41) | 5.34 (-4.06, 15.65) |
| Bulgaria | 60 to 64 | 5.11 (-0.72, 11.27) | 5.26 (-2.82, 14.02) | 4.9 (-3.31, 13.79) |
| Bulgaria | 65 to 69 | 4.55 (-0.74, 10.13) | 4.8 (-2.62, 12.8) | 4.34 (-3.05, 12.3) |
| Bulgaria | 70 to 74 | 3.97 (-1.16, 9.36) | 4.17 (-3, 11.88) | 3.89 (-3.3, 11.62) |
| Bulgaria | 75 to 79 | 3.52 (-2.57, 9.99) | 3.61 (-4.9, 12.89) | 3.63 (-4.93, 12.95) |
| Bulgaria | 80 to 84 | 3.29 (-3.77, 10.87) | 3.59 (-6.19, 14.39) | 3.23 (-6.71, 14.23) |
| Bulgaria | 85 to 89 | 1.98 (-7.59, 12.55) | 2.39 (-10.67, 17.35) | 1.84 (-11.71, 17.46) |
| Burkina Faso | 25 to 29 | 0.8 (-33.82, 53.52) | -7.29 (-38.68, 40.18) | -5.46 (-36.97, 41.79) |
| Burkina Faso | 30 to 34 | -1.72 (-29.99, 37.95) | -10.73 (-38.59, 29.78) | -9.44 (-35.47, 27.08) |
| Burkina Faso | 35 to 39 | -1.13 (-27.83, 35.45) | -10.75 (-36.39, 25.24) | -10.07 (-35.35, 25.09) |
| Burkina Faso | 40 to 44 | -0.09 (-26.09, 35.05) | -8.78 (-33.44, 25.01) | -8.7 (-35.07, 28.39) |
| Burkina Faso | 45 to 49 | 1.29 (-23.33, 33.8) | -5.08 (-28.05, 25.22) | -4.05 (-30.21, 31.92) |
| Burkina Faso | 50 to 54 | 2.53 (-19.58, 30.7) | -2.67 (-26.16, 28.27) | 0.19 (-24.95, 33.76) |
| Burkina Faso | 55 to 59 | 3.4 (-16.56, 28.13) | -0.69 (-23.74, 29.33) | 1.51 (-22.72, 33.33) |
| Burkina Faso | 60 to 64 | 2.56 (-15.48, 24.46) | 1.03 (-22.3, 31.35) | 2.11 (-21.78, 33.3) |
| Burkina Faso | 65 to 69 | 2.61 (-14.48, 23.12) | 2.21 (-20.93, 32.13) | 2.57 (-20.91, 33.03) |
| Burkina Faso | 70 to 74 | 2.5 (-14.13, 22.35) | 2.65 (-20.3, 32.22) | 2.11 (-21, 31.98) |
| Burkina Faso | 75 to 79 | 2.6 (-14.67, 23.36) | 3.14 (-22.23, 36.78) | 2.34 (-20.89, 32.39) |
| Burkina Faso | 80 to 84 | 3.02 (-18.44, 30.13) | 3.32 (-27.38, 47.01) | 2.93 (-23.26, 38.06) |
| Burkina Faso | 85 to 89 | 3.55 (-28.57, 50.13) | 4.65 (-44, 95.57) | 3.66 (-34.26, 63.44) |
| Burundi | 25 to 29 | -13.46 (-54.21, 63.59) | -14.25 (-54.36, 61.1) | -4.71 (-27.28, 24.88) |
| Burundi | 30 to 34 | -10.76 (-44.93, 44.61) | -10.72 (-42.77, 39.27) | -5.05 (-28.17, 25.5) |
| Burundi | 35 to 39 | -5.85 (-37.35, 41.48) | -3.76 (-29.48, 31.34) | -3.61 (-25.8, 25.22) |
| Burundi | 40 to 44 | -2.98 (-31.8, 38.01) | -1.56 (-29.8, 38.04) | -2.38 (-27.64, 31.71) |
| Burundi | 45 to 49 | -0.22 (-25.5, 33.64) | -1.03 (-29.87, 39.68) | -2.19 (-29.62, 35.92) |
| Burundi | 50 to 54 | -0.84 (-22.7, 27.21) | -0.72 (-27.73, 36.39) | -2 (-29.19, 35.65) |
| Burundi | 55 to 59 | -1.62 (-20.55, 21.83) | -1.78 (-25.98, 30.34) | -1.51 (-27.11, 33.09) |
| Burundi | 60 to 64 | -1.24 (-18.23, 19.29) | -1.5 (-23.55, 26.91) | -1.12 (-25.06, 30.47) |
| Burundi | 65 to 69 | -1.05 (-17.39, 18.51) | -1.2 (-22.65, 26.19) | -0.96 (-24.58, 30.06) |
| Burundi | 70 to 74 | -0.87 (-17.5, 19.11) | -0.94 (-22.73, 26.99) | -0.64 (-24.53, 30.81) |
| Burundi | 75 to 79 | -0.56 (-18.75, 21.7) | -0.82 (-25.4, 31.86) | -0.6 (-25.76, 33.09) |
| Burundi | 80 to 84 | 0.03 (-22.91, 29.79) | -0.25 (-33.47, 49.56) | -0.32 (-29.25, 40.46) |
| Burundi | 85 to 89 | 0.16 (-32.75, 49.2) | -0.22 (-48.96, 95.05) | -0.14 (-39.03, 63.54) |
| Cabo Verde | 25 to 29 | -1.96 (-23.45, 25.56) | -2.93 (-23.9, 23.81) | -2.18 (-23.37, 24.87) |
| Cabo Verde | 30 to 34 | -2.41 (-19.47, 18.26) | -3.17 (-19.99, 17.18) | -2.14 (-19.14, 18.43) |
| Cabo Verde | 35 to 39 | -2.85 (-18.59, 15.95) | -3.56 (-18.96, 14.76) | -1.99 (-17.64, 16.63) |
| Cabo Verde | 40 to 44 | -4.05 (-19.67, 14.62) | -4.49 (-19.29, 13.02) | -2.56 (-17.66, 15.3) |
| Cabo Verde | 45 to 49 | -5.92 (-22.03, 13.51) | -5.99 (-20.38, 11) | -4.09 (-18.77, 13.24) |
| Cabo Verde | 50 to 54 | -6.65 (-23.42, 13.8) | -6.51 (-20.71, 10.23) | -4.83 (-19.29, 12.22) |
| Cabo Verde | 55 to 59 | -4.68 (-21.55, 15.82) | -4.63 (-19.11, 12.44) | -3.27 (-18.05, 14.18) |
| Cabo Verde | 60 to 64 | -2.62 (-19.16, 17.3) | -2.25 (-17.1, 15.25) | -1.61 (-16.64, 16.14) |
| Cabo Verde | 65 to 69 | -1.54 (-17.86, 18.03) | -0.43 (-15.67, 17.56) | -0.82 (-16.16, 17.32) |
| Cabo Verde | 70 to 74 | -2.49 (-20.31, 19.32) | -0.09 (-15.57, 18.23) | -1.23 (-17.17, 17.77) |
| Cabo Verde | 75 to 79 | -4.47 (-24.8, 21.35) | -0.9 (-16.73, 17.93) | -2.52 (-19.47, 18) |
| Cabo Verde | 80 to 84 | -5.67 (-28.1, 23.75) | -1.48 (-18.59, 19.22) | -3.5 (-22.58, 20.28) |
| Cabo Verde | 85 to 89 | -4.79 (-30.6, 30.61) | -0.57 (-22.04, 26.83) | -2.74 (-25.64, 27.21) |
| Cambodia | 25 to 29 | -8.8 (-50.76, 68.94) | -4.58 (-27.55, 25.69) | -9.19 (-50.11, 65.27) |
| Cambodia | 30 to 34 | -1.84 (-34.33, 46.74) | -4.88 (-28.62, 26.75) | -3.08 (-27.08, 28.82) |
| Cambodia | 35 to 39 | 0.6 (-25.84, 36.45) | -4.5 (-28.3, 27.21) | -1.27 (-26.37, 32.38) |
| Cambodia | 40 to 44 | 2.2 (-19.69, 30.04) | -1.81 (-27.58, 33.12) | 0.03 (-25.33, 34) |
| Cambodia | 45 to 49 | 2.29 (-17.64, 27.04) | 0.4 (-25.44, 35.19) | 0.63 (-23.64, 32.63) |
| Cambodia | 50 to 54 | 2.07 (-15.87, 23.83) | 1.37 (-24.74, 36.54) | 1.13 (-21.37, 30.08) |
| Cambodia | 55 to 59 | 1.83 (-14.7, 21.55) | 2.39 (-22.97, 36.09) | 1.88 (-19.04, 28.21) |
| Cambodia | 60 to 64 | 1.78 (-14.1, 20.6) | 2.84 (-21.73, 35.14) | 1.22 (-18.7, 26.02) |
| Cambodia | 65 to 69 | 1.96 (-14.06, 20.97) | 1.79 (-22.28, 33.3) | 1.47 (-18.39, 26.16) |
| Cambodia | 70 to 74 | 2.22 (-14.92, 22.81) | 2.17 (-23.21, 35.94) | 2.19 (-19.39, 29.53) |
| Cambodia | 75 to 79 | 2.63 (-17.88, 28.25) | 2.36 (-28.2, 45.93) | 2.29 (-22.38, 34.81) |
| Cambodia | 80 to 84 | 2.63 (-23.94, 38.47) | 2.18 (-36.8, 65.19) | 2.61 (-29.98, 50.35) |
| Cambodia | 85 to 89 | 2.04 (-35.58, 61.61) | 1.15 (-48.89, 100.16) | 3.09 (-45.25, 94.11) |
| Cameroon | 25 to 29 | 5.75 (-18.84, 37.81) | 1.85 (-27.42, 42.9) | 4.98 (-27.01, 51) |
| Cameroon | 30 to 34 | 4.82 (-17.96, 33.91) | -0.51 (-24.98, 31.96) | 4.08 (-25.43, 45.27) |
| Cameroon | 35 to 39 | 3.9 (-16.86, 29.84) | -0.23 (-23.72, 30.47) | 3.2 (-24.48, 41.02) |
| Cameroon | 40 to 44 | 3.38 (-15.16, 25.97) | 0.79 (-22.27, 30.71) | 2.83 (-22.44, 36.34) |
| Cameroon | 45 to 49 | 3.16 (-13.33, 22.78) | 1.78 (-20.12, 29.69) | 2.84 (-19.38, 31.2) |
| Cameroon | 50 to 54 | 3.19 (-11.02, 19.66) | 2.81 (-16.86, 27.14) | 2.8 (-16.47, 26.5) |
| Cameroon | 55 to 59 | 3.17 (-9.33, 17.4) | 3.59 (-14.32, 25.24) | 2.91 (-13.89, 22.99) |
| Cameroon | 60 to 64 | 3.09 (-8.45, 16.09) | 3.08 (-13.29, 22.54) | 2.82 (-12.71, 21.12) |
| Cameroon | 65 to 69 | 2.92 (-8.34, 15.57) | 2.85 (-13.18, 21.83) | 2.6 (-12.45, 20.23) |
| Cameroon | 70 to 74 | 2.63 (-9.12, 15.9) | 2.46 (-14.16, 22.29) | 2.37 (-13.34, 20.92) |
| Cameroon | 75 to 79 | 2.37 (-10.63, 17.27) | 2.24 (-16.32, 24.92) | 2.2 (-15.06, 22.97) |
| Cameroon | 80 to 84 | 2.37 (-14.12, 22.02) | 2.49 (-22.14, 34.89) | 2.16 (-18.75, 28.47) |
| Cameroon | 85 to 89 | 2.35 (-20.42, 31.64) | 3.05 (-30.02, 51.73) | 2.08 (-26.76, 42.29) |
| Canada | 25 to 29 | 7.76 (-24.34, 53.48) | -2.22 (-33.82, 44.47) | -0.44 (-33.07, 48.1) |
| Canada | 30 to 34 | 6.65 (-14.53, 33.07) | 2.39 (-24.4, 38.69) | 3.3 (-21.32, 35.63) |
| Canada | 35 to 39 | 5.71 (-9.5, 23.49) | 4.02 (-16.75, 29.97) | 4.2 (-15.12, 27.92) |
| Canada | 40 to 44 | 5.2 (-6.45, 18.32) | 4.63 (-11.38, 23.53) | 4.4 (-11.19, 22.72) |
| Canada | 45 to 49 | 4.91 (-4.11, 14.78) | 4.98 (-7.29, 18.88) | 4.77 (-7.72, 18.95) |
| Canada | 50 to 54 | 4.25 (-3.09, 12.14) | 4.1 (-5.98, 15.27) | 4.66 (-5.67, 16.12) |
| Canada | 55 to 59 | 3.46 (-2.7, 10.02) | 3.05 (-5.44, 12.31) | 3.79 (-4.94, 13.33) |
| Canada | 60 to 64 | 2.7 (-2.54, 8.21) | 2.16 (-5.07, 9.94) | 3.21 (-4.22, 11.22) |
| Canada | 65 to 69 | 2.06 (-2.44, 6.78) | 1.44 (-4.82, 8.11) | 2.65 (-3.74, 9.45) |
| Canada | 70 to 74 | 1.41 (-2.48, 5.45) | 0.67 (-4.74, 6.39) | 2 (-3.49, 7.8) |
| Canada | 75 to 79 | 1.2 (-2.31, 4.85) | 0.45 (-4.53, 5.69) | 1.72 (-3.18, 6.87) |
| Canada | 80 to 84 | 1.47 (-2.04, 5.11) | 0.73 (-4.46, 6.19) | 1.93 (-2.79, 6.88) |
| Canada | 85 to 89 | 2.38 (-2.78, 7.8) | 1.55 (-6.28, 10.04) | 2.86 (-3.83, 10.02) |
| Central African Republic | 25 to 29 | 0.87 (-25.47, 36.53) | 1.33 (-31.52, 49.94) | 1.37 (-35.12, 58.38) |
| Central African Republic | 30 to 34 | 0.99 (-21.98, 30.73) | 1.26 (-28.35, 43.11) | 0.78 (-32.16, 49.71) |
| Central African Republic | 35 to 39 | 0.56 (-21.5, 28.83) | 0.52 (-27.49, 39.36) | 0.3 (-31.53, 46.94) |
| Central African Republic | 40 to 44 | 0.28 (-20.53, 26.56) | 0.08 (-26.2, 35.71) | 0.4 (-30.07, 44.14) |
| Central African Republic | 45 to 49 | 0.06 (-19.42, 24.26) | 0.12 (-24.38, 32.56) | -0.1 (-28.58, 39.73) |
| Central African Republic | 50 to 54 | 0 (-18.11, 22.1) | 0.04 (-22.84, 29.72) | 0.04 (-26.81, 36.74) |
| Central African Republic | 55 to 59 | -0.25 (-17.44, 20.52) | -0.37 (-22.16, 27.52) | -0.02 (-25.54, 34.25) |
| Central African Republic | 60 to 64 | -0.41 (-17.82, 20.68) | -0.35 (-22.27, 27.75) | -0.17 (-26.34, 35.31) |
| Central African Republic | 65 to 69 | -0.57 (-19.34, 22.57) | -0.52 (-24.51, 31.1) | -0.07 (-28.17, 39.02) |
| Central African Republic | 70 to 74 | -0.58 (-21.75, 26.33) | -0.64 (-28.25, 37.58) | -0.18 (-29.73, 41.81) |
| Central African Republic | 75 to 79 | -0.68 (-25.34, 32.13) | -0.94 (-34.03, 48.74) | -0.11 (-33.21, 49.38) |
| Central African Republic | 80 to 84 | -0.25 (-33.59, 49.82) | -6.29 (-39.67, 45.56) | -0.15 (-40.95, 68.85) |
| Central African Republic | 85 to 89 | -0.4 (-48.53, 92.72) | -10.91 (-46.18, 47.48) | -2.3 (-53.43, 104.96) |
| Chad | 25 to 29 | -4.5 (-45.17, 66.32) | -9.75 (-45.87, 50.48) | -13.04 (-53.76, 63.54) |
| Chad | 30 to 34 | -7.86 (-41.88, 46.08) | -12.04 (-42.47, 34.47) | -11.06 (-42.89, 38.51) |
| Chad | 35 to 39 | -8.04 (-36.12, 32.4) | -12.19 (-41.9, 32.71) | -6.53 (-30.44, 25.6) |
| Chad | 40 to 44 | -5.71 (-33.81, 34.31) | -10.07 (-37.25, 28.89) | -6.41 (-33.28, 31.27) |
| Chad | 45 to 49 | -2.56 (-30.59, 36.77) | -7.38 (-34.21, 30.39) | -6.97 (-32.86, 28.91) |
| Chad | 50 to 54 | 0.26 (-26.26, 36.31) | -4.8 (-32.08, 33.44) | -4.65 (-33.27, 36.24) |
| Chad | 55 to 59 | 1.12 (-24.44, 35.33) | -2.72 (-29.94, 35.08) | -2.11 (-33.1, 43.25) |
| Chad | 60 to 64 | 1.76 (-22.32, 33.31) | -0.89 (-28.16, 36.73) | -0.39 (-33.04, 48.19) |
| Chad | 65 to 69 | 1.15 (-22.6, 32.19) | 0.99 (-27.66, 40.99) | 1.34 (-33.27, 53.9) |
| Chad | 70 to 74 | 1.37 (-22.2, 32.08) | 1.34 (-27.03, 40.72) | 2.04 (-33.18, 55.82) |
| Chad | 75 to 79 | 1.23 (-22.93, 32.98) | 0.86 (-28.74, 42.76) | 0.41 (-35.87, 57.23) |
| Chad | 80 to 84 | 1.87 (-28.81, 45.76) | 2.59 (-36.94, 66.92) | 1.27 (-40.6, 72.64) |
| Chad | 85 to 89 | 1.8 (-38.18, 67.62) | 3.36 (-47.56, 103.74) | 0.81 (-50.73, 106.26) |
| Chile | 25 to 29 | 3.88 (-14.33, 25.95) | 3.72 (-20.77, 35.79) | 4.13 (-20.79, 36.91) |
| Chile | 30 to 34 | 3.44 (-10.97, 20.18) | 3.31 (-16.05, 27.14) | 3.73 (-16.02, 28.11) |
| Chile | 35 to 39 | 2.96 (-8.76, 16.18) | 2.66 (-13.42, 21.73) | 3.14 (-13.06, 22.36) |
| Chile | 40 to 44 | 2.26 (-7.46, 12.99) | 2.05 (-11.51, 17.69) | 2.44 (-10.88, 17.74) |
| Chile | 45 to 49 | 1.54 (-6.07, 9.75) | 1.57 (-9.15, 13.57) | 1.48 (-8.98, 13.14) |
| Chile | 50 to 54 | 1.25 (-4.87, 7.75) | 1.35 (-7.42, 10.94) | 1.16 (-7.17, 10.25) |
| Chile | 55 to 59 | 1.18 (-4, 6.65) | 1.44 (-6.03, 9.49) | 1 (-6.06, 8.58) |
| Chile | 60 to 64 | 1.34 (-3.27, 6.17) | 1.72 (-4.96, 8.86) | 0.99 (-5.29, 7.68) |
| Chile | 65 to 69 | 1.65 (-2.65, 6.14) | 2.01 (-4.24, 8.66) | 1.31 (-4.53, 7.51) |
| Chile | 70 to 74 | 2.21 (-1.94, 6.53) | 2.5 (-3.54, 8.92) | 1.93 (-3.7, 7.9) |
| Chile | 75 to 79 | 2.59 (-1.59, 6.93) | 2.84 (-3.34, 9.42) | 2.34 (-3.23, 8.24) |
| Chile | 80 to 84 | 3.01 (-1.76, 8) | 3.16 (-4.02, 10.88) | 2.84 (-3.42, 9.51) |
| Chile | 85 to 89 | 3.69 (-3.73, 11.68) | 3.84 (-7.43, 16.47) | 3.58 (-6.03, 14.17) |
| China | 25 to 29 | 4.03 (-2.97, 11.53) | 4.96 (-3.77, 14.49) | 2.71 (-8.6, 15.41) |
| China | 30 to 34 | 3.77 (-0.33, 8.05) | 4.71 (-0.54, 10.25) | 2.48 (-4.03, 9.44) |
| China | 35 to 39 | 3.57 (0.53, 6.71) | 4.43 (0.46, 8.56) | 2.47 (-2.25, 7.41) |
| China | 40 to 44 | 3.47 (1.12, 5.87) | 4.26 (1.11, 7.51) | 2.53 (-0.98, 6.18) |
| China | 45 to 49 | 3.44 (1.54, 5.36) | 4.11 (1.5, 6.79) | 2.7 (-0.04, 5.51) |
| China | 50 to 54 | 3.62 (2, 5.28) | 4.19 (1.89, 6.54) | 3.04 (0.74, 5.39) |
| China | 55 to 59 | 3.64 (2.15, 5.15) | 4.07 (1.93, 6.25) | 3.22 (1.15, 5.33) |
| China | 60 to 64 | 4.03 (2.68, 5.41) | 4.36 (2.39, 6.36) | 3.73 (1.87, 5.63) |
| China | 65 to 69 | 4.38 (3.1, 5.68) | 4.62 (2.75, 6.53) | 4.16 (2.41, 5.94) |
| China | 70 to 74 | 4.59 (3.3, 5.9) | 4.81 (2.91, 6.76) | 4.39 (2.63, 6.17) |
| China | 75 to 79 | 4.36 (2.95, 5.78) | 4.51 (2.42, 6.65) | 4.17 (2.28, 6.09) |
| China | 80 to 84 | 4.01 (2.21, 5.83) | 4.33 (1.63, 7.11) | 3.65 (1.27, 6.09) |
| China | 85 to 89 | 3.31 (0.65, 6.04) | 3.94 (-0.15, 8.2) | 2.67 (-0.8, 6.25) |
| Colombia | 25 to 29 | 1.99 (-7.97, 13.04) | 1.9 (-10.51, 16.02) | 1.96 (-14.01, 20.9) |
| Colombia | 30 to 34 | 1.47 (-7.34, 11.13) | 1.33 (-9.91, 13.97) | 1.48 (-12.3, 17.43) |
| Colombia | 35 to 39 | 1.02 (-7.08, 9.83) | 0.9 (-9.69, 12.75) | 1.08 (-11.12, 14.94) |
| Colombia | 40 to 44 | 0.72 (-6.53, 8.53) | 0.56 (-9.2, 11.38) | 0.9 (-9.59, 12.6) |
| Colombia | 45 to 49 | 0.66 (-5.5, 7.22) | 0.41 (-8.13, 9.73) | 0.93 (-7.77, 10.45) |
| Colombia | 50 to 54 | 0.61 (-4.53, 6.02) | 0.46 (-6.72, 8.2) | 0.79 (-6.42, 8.55) |
| Colombia | 55 to 59 | 0.55 (-3.92, 5.21) | 0.49 (-5.81, 7.22) | 0.64 (-5.56, 7.26) |
| Colombia | 60 to 64 | 0.72 (-3.32, 4.93) | 0.73 (-5, 6.81) | 0.73 (-4.88, 6.68) |
| Colombia | 65 to 69 | 0.98 (-2.9, 5.02) | 1.1 (-4.38, 6.89) | 0.89 (-4.53, 6.63) |
| Colombia | 70 to 74 | 1.16 (-2.75, 5.23) | 1.44 (-4.09, 7.3) | 0.95 (-4.5, 6.72) |
| Colombia | 75 to 79 | 1.45 (-2.75, 5.82) | 1.8 (-4.25, 8.23) | 1.19 (-4.57, 7.29) |
| Colombia | 80 to 84 | 1.48 (-3.57, 6.79) | 1.8 (-5.59, 9.77) | 1.25 (-5.55, 8.55) |
| Colombia | 85 to 89 | 1.17 (-6.03, 8.92) | 1.49 (-9.26, 13.51) | 0.93 (-8.51, 11.35) |
| Comoros | 25 to 29 | -1.67 (-23.8, 26.9) | -1.58 (-23.22, 26.16) | -1.76 (-23.36, 25.94) |
| Comoros | 30 to 34 | -2.35 (-19.75, 18.82) | -2.2 (-19.35, 18.61) | -2.1 (-19.28, 18.74) |
| Comoros | 35 to 39 | -3.21 (-19.98, 17.07) | -2.9 (-18.64, 15.89) | -2.47 (-18.29, 16.4) |
| Comoros | 40 to 44 | -4.1 (-22.59, 18.8) | -3.8 (-19.1, 14.39) | -2.94 (-18.59, 15.72) |
| Comoros | 45 to 49 | -4.49 (-27, 24.97) | -4.01 (-20.05, 15.24) | -3.03 (-19.62, 16.99) |
| Comoros | 50 to 54 | -5.98 (-30.77, 27.67) | -4.04 (-22.11, 18.23) | -4.41 (-22.83, 18.4) |
| Comoros | 55 to 59 | -4.51 (-32.99, 36.07) | -3.65 (-26.18, 25.75) | -6.12 (-30.14, 26.16) |
| Comoros | 60 to 64 | -2.9 (-34.37, 43.66) | -2.09 (-32.28, 41.56) | -8.83 (-35.16, 28.18) |
| Comoros | 65 to 69 | -0.63 (-35.4, 52.86) | -3.71 (-37.14, 47.51) | -8.05 (-39.53, 39.82) |
| Comoros | 70 to 74 | 0.67 (-36.21, 58.88) | -6.25 (-40.76, 48.36) | -12.44 (-41.97, 32.13) |
| Comoros | 75 to 79 | 1.12 (-38.25, 65.59) | -7.81 (-41.35, 44.92) | -13.71 (-42.29, 29.01) |
| Comoros | 80 to 84 | -6.87 (-42.31, 50.34) | -8.86 (-38.99, 36.15) | -13.7 (-41.59, 27.49) |
| Comoros | 85 to 89 | -11.65 (-47.25, 48) | -8.84 (-36.9, 31.69) | -12.18 (-41.09, 30.91) |
| Congo | 25 to 29 | 2.27 (-28.78, 46.86) | 0.03 (-39.06, 64.2) | 3.03 (-37.49, 69.82) |
| Congo | 30 to 34 | 2.02 (-26.14, 40.93) | 0.03 (-35.18, 54.36) | 2.18 (-33.7, 57.47) |
| Congo | 35 to 39 | 1.4 (-24.65, 36.44) | -0.46 (-33.93, 49.98) | 2.07 (-30.9, 50.75) |
| Congo | 40 to 44 | 0.86 (-22.79, 31.75) | -0.77 (-31.61, 43.97) | 1.21 (-29.55, 45.39) |
| Congo | 45 to 49 | 0.42 (-20.75, 27.24) | -0.85 (-28.94, 38.35) | 0.76 (-27.73, 40.48) |
| Congo | 50 to 54 | 0.01 (-18.95, 23.4) | -0.96 (-25.96, 32.48) | 0.66 (-25.83, 36.61) |
| Congo | 55 to 59 | -0.2 (-17.6, 20.88) | -1.04 (-23.26, 27.6) | 0.99 (-24.52, 35.13) |
| Congo | 60 to 64 | -0.26 (-16.95, 19.78) | -1.31 (-22.22, 25.22) | 0.72 (-24.64, 34.61) |
| Congo | 65 to 69 | -0.29 (-17.33, 20.27) | -1.01 (-22.79, 26.92) | 1.12 (-24.71, 35.8) |
| Congo | 70 to 74 | -0.1 (-18.03, 21.75) | -0.6 (-23.93, 29.89) | 1.09 (-24.98, 36.21) |
| Congo | 75 to 79 | 0.22 (-20.54, 26.41) | -0.49 (-27.34, 36.27) | 0.92 (-27.48, 40.44) |
| Congo | 80 to 84 | 0.38 (-26.52, 37.13) | 0.17 (-38, 61.86) | 1.83 (-36.53, 63.36) |
| Congo | 85 to 89 | 0.15 (-37.47, 60.39) | -0.71 (-50.36, 98.61) | 1.61 (-47.78, 97.71) |
| Cook Islands | 25 to 29 | 2.06 (-20.19, 30.51) | 2.05 (-20.02, 30.2) | 1.24 (-20.65, 29.17) |
| Cook Islands | 30 to 34 | 1.53 (-16.12, 22.9) | 1.91 (-15.79, 23.33) | 0.85 (-16.67, 22.04) |
| Cook Islands | 35 to 39 | 0.31 (-15.72, 19.39) | 0.75 (-15.34, 19.9) | -0.03 (-16, 18.96) |
| Cook Islands | 40 to 44 | -0.14 (-15.7, 18.29) | 0.32 (-15.22, 18.71) | -0.73 (-16.11, 17.47) |
| Cook Islands | 45 to 49 | -1.91 (-17.03, 15.97) | -1.73 (-16.78, 16.04) | -2.12 (-17.11, 15.57) |
| Cook Islands | 50 to 54 | -1.7 (-17.01, 16.45) | -1.38 (-16.46, 16.43) | -1.87 (-16.88, 15.84) |
| Cook Islands | 55 to 59 | -2.7 (-18.39, 16.01) | -2.28 (-17.12, 15.21) | -2.27 (-17.11, 15.23) |
| Cook Islands | 60 to 64 | -2.53 (-19.68, 18.28) | -1.47 (-16.61, 16.41) | -2.23 (-17.25, 15.52) |
| Cook Islands | 65 to 69 | -5.01 (-23.66, 18.19) | -2.17 (-17.57, 16.11) | -3.07 (-18.33, 15.05) |
| Cook Islands | 70 to 74 | -6.04 (-26.3, 19.79) | -1.86 (-17.8, 17.18) | -3.43 (-19.12, 15.3) |
| Cook Islands | 75 to 79 | -8.01 (-29.54, 20.09) | -3.78 (-20.18, 15.99) | -4.43 (-20.72, 15.21) |
| Cook Islands | 80 to 84 | -7.47 (-29.57, 21.56) | -3.58 (-20.44, 16.85) | -3.98 (-20.77, 16.37) |
| Cook Islands | 85 to 89 | -7.08 (-30.93, 25) | -4.54 (-25.2, 21.82) | -4.03 (-24.8, 22.47) |
| Costa Rica | 25 to 29 | 4.02 (-16.07, 28.93) | 3.78 (-21.54, 37.25) | 4.22 (-25.77, 46.33) |
| Costa Rica | 30 to 34 | 4.19 (-13.32, 25.24) | 4.11 (-18.19, 32.47) | 4.16 (-22.26, 39.54) |
| Costa Rica | 35 to 39 | 3.85 (-11.99, 22.55) | 4.16 (-16.14, 29.38) | 3.4 (-20.22, 34) |
| Costa Rica | 40 to 44 | 3.19 (-10.69, 19.24) | 3.52 (-14.6, 25.47) | 2.92 (-17.41, 28.27) |
| Costa Rica | 45 to 49 | 2.71 (-9.29, 16.3) | 3.18 (-12.72, 21.97) | 2.18 (-15.27, 23.22) |
| Costa Rica | 50 to 54 | 2.23 (-8.14, 13.76) | 2.77 (-11.29, 19.07) | 1.61 (-13.28, 19.07) |
| Costa Rica | 55 to 59 | 1.9 (-7.31, 12.02) | 2.25 (-10.5, 16.81) | 1.55 (-11.38, 16.36) |
| Costa Rica | 60 to 64 | 1.73 (-6.79, 11.02) | 2.06 (-9.82, 15.5) | 1.46 (-10.42, 14.92) |
| Costa Rica | 65 to 69 | 1.73 (-6.6, 10.81) | 1.92 (-9.6, 14.9) | 1.59 (-10.12, 14.83) |
| Costa Rica | 70 to 74 | 1.83 (-6.71, 11.16) | 1.98 (-9.72, 15.2) | 1.77 (-10.35, 15.53) |
| Costa Rica | 75 to 79 | 2.22 (-6.96, 12.31) | 2.21 (-10.23, 16.38) | 2.32 (-10.76, 17.32) |
| Costa Rica | 80 to 84 | 1.97 (-8.84, 14.06) | 1.84 (-12.6, 18.67) | 2.2 (-13.32, 20.5) |
| Costa Rica | 85 to 89 | 1.79 (-14.36, 20.99) | 1.86 (-20.11, 29.86) | 2.2 (-21.26, 32.65) |
| Croatia | 25 to 29 | 0.94 (-34.25, 54.97) | 1.33 (-40.99, 74.03) | 2.77 (-44.11, 88.99) |
| Croatia | 30 to 34 | 1.58 (-25.83, 39.14) | 1.07 (-33.15, 52.81) | 2.22 (-37.77, 67.91) |
| Croatia | 35 to 39 | 1.36 (-21.26, 30.46) | 0.94 (-28.31, 42.12) | 1.62 (-31.62, 51.02) |
| Croatia | 40 to 44 | 1.08 (-18.17, 24.87) | 0.82 (-24.26, 34.2) | 1.74 (-26.2, 40.26) |
| Croatia | 45 to 49 | 0.93 (-15.51, 20.58) | 0.39 (-21.59, 28.53) | 1.71 (-21.67, 32.06) |
| Croatia | 50 to 54 | 0.94 (-12.3, 16.18) | 0.67 (-17.25, 22.47) | 1.36 (-17.34, 24.3) |
| Croatia | 55 to 59 | 0.91 (-9.91, 13.04) | 0.93 (-13.93, 18.35) | 1.04 (-14.05, 18.77) |
| Croatia | 60 to 64 | 0.92 (-8.04, 10.75) | 1.12 (-11.59, 15.65) | 0.75 (-11.5, 14.68) |
| Croatia | 65 to 69 | 1.08 (-6.79, 9.61) | 1.42 (-10.34, 14.72) | 0.86 (-9.55, 12.46) |
| Croatia | 70 to 74 | 1.73 (-5.64, 9.68) | 2.06 (-9.53, 15.13) | 1.57 (-7.9, 12.01) |
| Croatia | 75 to 79 | 2.55 (-5.83, 11.69) | 2.05 (-11.51, 17.68) | 2.9 (-7.63, 14.63) |
| Croatia | 80 to 84 | 3.63 (-7.26, 15.8) | 1.94 (-14.79, 21.94) | 4.57 (-9.29, 20.54) |
| Croatia | 85 to 89 | 3.95 (-10.41, 20.59) | 1.83 (-19.02, 28.04) | 5.15 (-13.62, 28) |
| Cuba | 25 to 29 | 3.83 (-9.57, 19.21) | 2.89 (-14.93, 24.44) | 5.39 (-13.59, 28.54) |
| Cuba | 30 to 34 | 4.12 (-5.93, 15.23) | 3.18 (-10.13, 18.47) | 5.48 (-9.2, 22.53) |
| Cuba | 35 to 39 | 4.3 (-4.11, 13.44) | 3.57 (-7.37, 15.8) | 5.3 (-7.35, 19.68) |
| Cuba | 40 to 44 | 4.1 (-2.8, 11.49) | 3.6 (-5.36, 13.42) | 4.84 (-5.65, 16.51) |
| Cuba | 45 to 49 | 4.04 (-1.54, 9.95) | 3.59 (-3.72, 11.46) | 4.68 (-3.83, 13.93) |
| Cuba | 50 to 54 | 3.97 (-0.96, 9.14) | 3.54 (-2.95, 10.47) | 4.57 (-2.87, 12.58) |
| Cuba | 55 to 59 | 4 (-0.61, 8.82) | 3.63 (-2.49, 10.13) | 4.55 (-2.36, 11.95) |
| Cuba | 60 to 64 | 4.23 (-0.23, 8.9) | 3.81 (-2.15, 10.14) | 4.86 (-1.82, 12) |
| Cuba | 65 to 69 | 4.57 (0.16, 9.17) | 4.03 (-1.85, 10.27) | 5.33 (-1.29, 12.38) |
| Cuba | 70 to 74 | 4.96 (0.48, 9.63) | 4.2 (-1.82, 10.58) | 5.91 (-0.76, 13.04) |
| Cuba | 75 to 79 | 5.12 (0.32, 10.15) | 4.08 (-2.43, 11.02) | 6.3 (-0.76, 13.85) |
| Cuba | 80 to 84 | 4.85 (-1.14, 11.2) | 3.76 (-4.34, 12.55) | 6.01 (-2.76, 15.56) |
| Cuba | 85 to 89 | 4.82 (-4.15, 14.63) | 4 (-8.07, 17.65) | 5.59 (-7.33, 20.31) |
| Cyprus | 25 to 29 | -1.23 (-25.41, 30.79) | -2.63 (-24.06, 24.86) | -3.03 (-24.3, 24.21) |
| Cyprus | 30 to 34 | -2.46 (-20.39, 19.51) | -2.59 (-19.85, 18.39) | -2.76 (-19.98, 18.18) |
| Cyprus | 35 to 39 | -2.87 (-20.47, 18.63) | -2.1 (-18.35, 17.39) | -2.32 (-18.49, 17.06) |
| Cyprus | 40 to 44 | -2.14 (-21.58, 22.13) | -1.73 (-18.61, 18.65) | -1.67 (-18.42, 18.5) |
| Cyprus | 45 to 49 | -1.97 (-26.43, 30.63) | -1.86 (-21.17, 22.18) | -1.23 (-20.39, 22.54) |
| Cyprus | 50 to 54 | -1.89 (-28.71, 35) | -2.05 (-26.77, 31.02) | -0.74 (-26.11, 33.34) |
| Cyprus | 55 to 59 | -2.51 (-28.86, 33.59) | -2.12 (-29.72, 36.3) | -1.51 (-30.6, 39.78) |
| Cyprus | 60 to 64 | -2.81 (-26.67, 28.81) | -2.15 (-30.19, 37.16) | -2.45 (-31.57, 39.06) |
| Cyprus | 65 to 69 | -3.14 (-23.83, 23.18) | -2.77 (-28.87, 32.9) | -3.24 (-30.46, 34.64) |
| Cyprus | 70 to 74 | -2.96 (-20.49, 18.45) | -2.77 (-25.6, 27.08) | -3.29 (-26.7, 27.6) |
| Cyprus | 75 to 79 | -3.15 (-17.97, 14.36) | -2.77 (-23.04, 22.84) | -3.76 (-23.74, 21.44) |
| Cyprus | 80 to 84 | -4.06 (-17.25, 11.22) | -3.71 (-22.72, 19.98) | -4.54 (-21.97, 16.78) |
| Cyprus | 85 to 89 | -4.65 (-25.68, 22.34) | -4.15 (-34.17, 39.55) | -5.11 (-31.52, 31.48) |
| Czechia | 25 to 29 | -0.64 (-28.05, 37.23) | -1.2 (-34.44, 48.89) | -0.01 (-40.65, 68.45) |
| Czechia | 30 to 34 | -0.38 (-21.95, 27.16) | -0.78 (-26.88, 34.64) | 0.16 (-32.85, 49.4) |
| Czechia | 35 to 39 | -0.95 (-19.16, 21.35) | -1.3 (-23.78, 27.82) | -0.06 (-28.1, 38.92) |
| Czechia | 40 to 44 | -1.9 (-17.36, 16.46) | -2.41 (-21.67, 21.58) | -0.43 (-24.06, 30.55) |
| Czechia | 45 to 49 | -2.31 (-15.74, 13.25) | -2.95 (-19.97, 17.68) | -1.14 (-21.68, 24.78) |
| Czechia | 50 to 54 | -2.45 (-13.87, 10.49) | -2.82 (-17.54, 14.52) | -1.52 (-18.64, 19.2) |
| Czechia | 55 to 59 | -2.63 (-12.36, 8.18) | -2.78 (-15.68, 12.09) | -2.1 (-16.22, 14.39) |
| Czechia | 60 to 64 | -2.33 (-10.15, 6.16) | -2.01 (-12.81, 10.12) | -2.51 (-13.58, 9.96) |
| Czechia | 65 to 69 | -2.07 (-8.65, 4.97) | -1.33 (-10.86, 9.21) | -2.64 (-11.65, 7.29) |
| Czechia | 70 to 74 | -1.3 (-7.26, 5.04) | -0.56 (-9.7, 9.51) | -1.84 (-9.65, 6.65) |
| Czechia | 75 to 79 | -0.62 (-7.17, 6.4) | -0.12 (-10.9, 11.97) | -0.93 (-9.13, 8.02) |
| Czechia | 80 to 84 | 0.36 (-6.67, 7.91) | 0.48 (-11.31, 13.82) | 0.21 (-8.38, 9.61) |
| Czechia | 85 to 89 | 1.85 (-8.49, 13.36) | 1.71 (-16.44, 23.8) | 1.88 (-10.37, 15.81) |
| Côte d'Ivoire | 25 to 29 | 3.77 (-15.04, 26.75) | 3.3 (-18.76, 31.34) | 5.63 (-27.48, 53.87) |
| Côte d'Ivoire | 30 to 34 | 3.61 (-14.24, 25.17) | 3.08 (-18.08, 29.71) | 5.49 (-25.06, 48.51) |
| Côte d'Ivoire | 35 to 39 | 3.23 (-13.72, 23.51) | 2.49 (-17.68, 27.6) | 4.88 (-23.62, 44.03) |
| Côte d'Ivoire | 40 to 44 | 2.78 (-13.16, 21.64) | 2.03 (-17.02, 25.47) | 4.02 (-22.29, 39.23) |
| Côte d'Ivoire | 45 to 49 | 2.46 (-12.35, 19.78) | 1.61 (-16.22, 23.24) | 3.38 (-20.32, 34.15) |
| Côte d'Ivoire | 50 to 54 | 2.17 (-11.06, 17.37) | 1.38 (-14.57, 20.3) | 2.88 (-18.42, 29.74) |
| Côte d'Ivoire | 55 to 59 | 1.84 (-10.28, 15.6) | 1.16 (-13.46, 18.26) | 2.7 (-17.25, 27.46) |
| Côte d'Ivoire | 60 to 64 | 1.62 (-9.95, 14.67) | 1.13 (-12.69, 17.14) | 2.61 (-16.91, 26.71) |
| Côte d'Ivoire | 65 to 69 | 1.44 (-10.21, 14.6) | 1 (-13.07, 17.34) | 2.36 (-17.34, 26.77) |
| Côte d'Ivoire | 70 to 74 | 1.3 (-10.84, 15.09) | 0.95 (-13.75, 18.15) | 2.28 (-18.16, 27.82) |
| Côte d'Ivoire | 75 to 79 | 1.35 (-12.49, 17.37) | 1.14 (-16.34, 22.28) | 2 (-19.74, 29.63) |
| Côte d'Ivoire | 80 to 84 | 1.51 (-17.07, 24.25) | 1.09 (-23.07, 32.83) | 1.56 (-23.77, 35.32) |
| Côte d'Ivoire | 85 to 89 | 0.98 (-24.81, 35.61) | 1.63 (-35.24, 59.49) | 1.56 (-35.47, 59.84) |
| Democratic People's Republic of Korea | 25 to 29 | 0.19 (-49.11, 97.27) | -0.03 (-50.67, 102.6) | 0.15 (-24.03, 32.03) |
| Democratic People's Republic of Korea | 30 to 34 | 0.41 (-30.91, 45.95) | 0.48 (-36.43, 58.82) | 0.3 (-24.81, 33.81) |
| Democratic People's Republic of Korea | 35 to 39 | -0.07 (-23.72, 30.93) | -0.14 (-30.18, 42.82) | 0.39 (-23.96, 32.56) |
| Democratic People's Republic of Korea | 40 to 44 | 0.06 (-18.26, 22.49) | 0.06 (-24.55, 32.69) | 0.52 (-21.6, 28.89) |
| Democratic People's Republic of Korea | 45 to 49 | 0.06 (-14.22, 16.71) | 0 (-20.1, 25.17) | 0.18 (-18.44, 23.05) |
| Democratic People's Republic of Korea | 50 to 54 | -0.13 (-12.15, 13.55) | -0.01 (-17.17, 20.7) | 0 (-15.79, 18.76) |
| Democratic People's Republic of Korea | 55 to 59 | -0.19 (-10.77, 11.64) | -0.3 (-15.73, 17.95) | -0.19 (-14.08, 15.94) |
| Democratic People's Republic of Korea | 60 to 64 | -0.34 (-10.05, 10.41) | -0.44 (-15.28, 17) | -0.34 (-12.81, 13.91) |
| Democratic People's Republic of Korea | 65 to 69 | -0.53 (-9.87, 9.78) | -0.64 (-15.74, 17.17) | -0.5 (-12.23, 12.8) |
| Democratic People's Republic of Korea | 70 to 74 | -0.66 (-9.97, 9.61) | -0.7 (-16.78, 18.49) | -0.62 (-11.86, 12.04) |
| Democratic People's Republic of Korea | 75 to 79 | -0.81 (-11.26, 10.86) | -0.67 (-19.73, 22.93) | -0.73 (-13.09, 13.38) |
| Democratic People's Republic of Korea | 80 to 84 | -1.11 (-14.36, 14.19) | -1.09 (-24.05, 28.8) | -0.99 (-16.7, 17.68) |
| Democratic People's Republic of Korea | 85 to 89 | -1.45 (-20.22, 21.74) | -1.28 (-34.3, 48.34) | -1.06 (-23.63, 28.19) |
| Democratic Republic of the Congo | 25 to 29 | -1.55 (-8.69, 6.15) | -1.17 (-10.96, 9.7) | -1.88 (-12.08, 9.51) |
| Democratic Republic of the Congo | 30 to 34 | -1.67 (-7.96, 5.05) | -1.32 (-10.21, 8.46) | -1.96 (-10.67, 7.6) |
| Democratic Republic of the Congo | 35 to 39 | -1.78 (-7.78, 4.62) | -1.58 (-9.89, 7.48) | -1.91 (-10.4, 7.38) |
| Democratic Republic of the Congo | 40 to 44 | -1.79 (-7.44, 4.2) | -1.83 (-9.56, 6.55) | -1.73 (-9.82, 7.08) |
| Democratic Republic of the Congo | 45 to 49 | -1.88 (-7.11, 3.65) | -2.07 (-9.24, 5.67) | -1.7 (-9.17, 6.38) |
| Democratic Republic of the Congo | 50 to 54 | -1.98 (-6.66, 2.93) | -2.3 (-8.77, 4.64) | -1.71 (-8.34, 5.4) |
| Democratic Republic of the Congo | 55 to 59 | -2.16 (-6.41, 2.29) | -2.62 (-8.52, 3.67) | -1.76 (-7.78, 4.66) |
| Democratic Republic of the Congo | 60 to 64 | -2.28 (-6.36, 1.98) | -2.83 (-8.47, 3.15) | -1.77 (-7.59, 4.41) |
| Democratic Republic of the Congo | 65 to 69 | -2.41 (-6.58, 1.95) | -3.05 (-8.88, 3.16) | -1.81 (-7.7, 4.47) |
| Democratic Republic of the Congo | 70 to 74 | -2.47 (-6.85, 2.13) | -3.16 (-9.44, 3.57) | -1.79 (-7.87, 4.69) |
| Democratic Republic of the Congo | 75 to 79 | -2.41 (-7.33, 2.76) | -3.14 (-10.48, 4.8) | -1.78 (-8.37, 5.27) |
| Democratic Republic of the Congo | 80 to 84 | -2.21 (-8.63, 4.66) | -2.96 (-12.67, 7.83) | -1.76 (-10.18, 7.44) |
| Democratic Republic of the Congo | 85 to 89 | -1.87 (-12.37, 9.89) | -2.63 (-18.49, 16.31) | -1.68 (-15.28, 14.1) |
| Denmark | 25 to 29 | -2.59 (-53.51, 104.11) | 0.54 (-21.16, 28.21) | 0.51 (-21.15, 28.13) |
| Denmark | 30 to 34 | -6.05 (-44.17, 58.08) | 0.96 (-16.88, 22.63) | 0.89 (-16.99, 22.61) |
| Denmark | 35 to 39 | -6.9 (-39.55, 43.4) | 0.57 (-16.57, 21.22) | 0.4 (-16.82, 21.19) |
| Denmark | 40 to 44 | -5.4 (-36.21, 40.29) | 0.01 (-18.56, 22.81) | -1.04 (-19.29, 21.33) |
| Denmark | 45 to 49 | -2.77 (-30.89, 36.78) | 0.06 (-21.04, 26.81) | -1.06 (-22.35, 26.05) |
| Denmark | 50 to 54 | -0.34 (-23.85, 30.44) | 0.11 (-21.65, 27.91) | -0.81 (-23.28, 28.24) |
| Denmark | 55 to 59 | 1.71 (-16.08, 23.28) | 0.72 (-18.85, 25.01) | -0.21 (-21.76, 27.28) |
| Denmark | 60 to 64 | 0.96 (-12.36, 16.31) | 0.96 (-15.33, 20.4) | 0.38 (-18.12, 23.05) |
| Denmark | 65 to 69 | 0.7 (-9.47, 12.01) | 0.91 (-12.15, 15.92) | 0.99 (-13.72, 18.19) |
| Denmark | 70 to 74 | 1.03 (-6.93, 9.68) | 1.11 (-9.34, 12.76) | 0.91 (-10.55, 13.85) |
| Denmark | 75 to 79 | 2.02 (-4.86, 9.4) | 1.93 (-7.31, 12.1) | 2.06 (-7.64, 12.78) |
| Denmark | 80 to 84 | 4.01 (-2.86, 11.38) | 3.36 (-6.15, 13.83) | 4.48 (-5.13, 15.07) |
| Denmark | 85 to 89 | 5.66 (-3.72, 15.95) | 4.58 (-8.65, 19.73) | 6.15 (-6.49, 20.49) |
| Djibouti | 25 to 29 | -3.41 (-25.13, 24.61) | -3.09 (-24.55, 24.47) | -2.79 (-24.33, 24.88) |
| Djibouti | 30 to 34 | -4.63 (-21.88, 16.44) | -3.91 (-20.92, 16.77) | -4.43 (-21.14, 15.83) |
| Djibouti | 35 to 39 | -5.52 (-23.16, 16.16) | -4.96 (-20.97, 14.29) | -5.03 (-20.56, 13.54) |
| Djibouti | 40 to 44 | -7.04 (-26.72, 17.92) | -5.89 (-22.84, 14.79) | -5.74 (-21.27, 12.85) |
| Djibouti | 45 to 49 | -7.93 (-28.91, 19.24) | -7.16 (-25.89, 16.31) | -5.9 (-22.66, 14.5) |
| Djibouti | 50 to 54 | -9.57 (-31.27, 18.99) | -8.85 (-28.58, 16.33) | -7.12 (-25.73, 16.14) |
| Djibouti | 55 to 59 | -10.73 (-32.17, 17.49) | -10.84 (-31.95, 16.83) | -8.73 (-28.9, 17.16) |
| Djibouti | 60 to 64 | -11.81 (-33.11, 16.29) | -12.81 (-34.49, 16.04) | -11.36 (-32.9, 17.1) |
| Djibouti | 65 to 69 | -12.38 (-33.44, 15.35) | -13.54 (-35.59, 16.04) | -12.62 (-34.94, 17.36) |
| Djibouti | 70 to 74 | -14.39 (-35.05, 12.84) | -12.48 (-33.75, 15.62) | -12.68 (-35.15, 17.58) |
| Djibouti | 75 to 79 | -14.55 (-37.06, 16) | -10.3 (-30.71, 16.12) | -10.88 (-32.31, 17.34) |
| Djibouti | 80 to 84 | -13.79 (-38.5, 20.87) | -8.19 (-27.5, 16.27) | -8.89 (-29.6, 17.91) |
| Djibouti | 85 to 89 | -12.51 (-39.44, 26.4) | -7.02 (-27.57, 19.36) | -7.38 (-29.97, 22.5) |
| Dominica | 25 to 29 | 0.09 (-22.72, 29.63) | 0.14 (-22.47, 29.35) | 0.51 (-21.66, 28.95) |
| Dominica | 30 to 34 | 0.38 (-18.2, 23.18) | 0.71 (-17.43, 22.85) | 0.66 (-17.13, 22.27) |
| Dominica | 35 to 39 | -0.89 (-20.66, 23.8) | 0.68 (-17.14, 22.32) | 0.14 (-16.24, 19.72) |
| Dominica | 40 to 44 | -3.02 (-28.32, 31.22) | -1.76 (-21.18, 22.44) | -1.45 (-17.85, 18.23) |
| Dominica | 45 to 49 | -6.35 (-33.11, 31.12) | -6.39 (-29.24, 23.84) | -3.35 (-20.89, 18.08) |
| Dominica | 50 to 54 | -5.31 (-35.06, 38.06) | -9.38 (-35.67, 27.66) | -4.1 (-23.22, 19.77) |
| Dominica | 55 to 59 | -2.8 (-35.45, 46.38) | -13.07 (-38.28, 22.46) | -3.32 (-23.11, 21.55) |
| Dominica | 60 to 64 | 0.14 (-34.9, 54.04) | -13.72 (-40.29, 24.68) | -1.9 (-21.27, 22.23) |
| Dominica | 65 to 69 | 1.81 (-34.54, 58.36) | -8.01 (-39.91, 40.82) | -0.76 (-21.09, 24.82) |
| Dominica | 70 to 74 | 2.28 (-34, 58.5) | -5.1 (-37.6, 44.32) | -4.35 (-29.57, 29.9) |
| Dominica | 75 to 79 | 1.51 (-37.57, 65.04) | -3.15 (-34.93, 44.15) | -3.85 (-38.22, 49.64) |
| Dominica | 80 to 84 | 1.94 (-41.65, 78.08) | -2.22 (-29.84, 36.27) | -3.39 (-44.88, 69.34) |
| Dominica | 85 to 89 | 1.23 (-51.99, 113.45) | -3.33 (-29.86, 33.24) | -8.6 (-45.88, 54.35) |
| Dominican Republic | 25 to 29 | 8.99 (-11.49, 34.21) | 8.43 (-18.21, 43.74) | 9.17 (-19.62, 48.29) |
| Dominican Republic | 30 to 34 | 8.16 (-8.46, 27.8) | 7.63 (-13.49, 33.9) | 8.57 (-16.75, 41.59) |
| Dominican Republic | 35 to 39 | 7.38 (-6.68, 23.55) | 6.97 (-10.59, 27.99) | 7.68 (-14.72, 35.96) |
| Dominican Republic | 40 to 44 | 6.72 (-5.39, 20.38) | 6.45 (-8.3, 23.58) | 7.02 (-13.02, 31.67) |
| Dominican Republic | 45 to 49 | 6.09 (-4.5, 17.86) | 5.92 (-6.89, 20.48) | 6.27 (-11.44, 27.53) |
| Dominican Republic | 50 to 54 | 5.39 (-4.1, 15.81) | 5.29 (-6.23, 18.21) | 5.43 (-10.38, 24.04) |
| Dominican Republic | 55 to 59 | 4.64 (-4.19, 14.29) | 4.6 (-6.14, 16.57) | 4.53 (-10.17, 21.62) |
| Dominican Republic | 60 to 64 | 4.05 (-4.35, 13.18) | 3.97 (-6.24, 15.29) | 4.01 (-9.93, 20.12) |
| Dominican Republic | 65 to 69 | 3.58 (-4.86, 12.77) | 3.48 (-6.82, 14.92) | 3.6 (-10.37, 19.75) |
| Dominican Republic | 70 to 74 | 2.93 (-5.68, 12.33) | 2.88 (-7.92, 14.95) | 2.95 (-10.86, 18.9) |
| Dominican Republic | 75 to 79 | 2.69 (-6.54, 12.83) | 2.64 (-9.25, 16.09) | 2.73 (-11.57, 19.35) |
| Dominican Republic | 80 to 84 | 2.93 (-8.04, 15.21) | 2.55 (-11.4, 18.69) | 3.49 (-13.44, 23.72) |
| Dominican Republic | 85 to 89 | 3.05 (-12.85, 21.84) | 3.03 (-17.95, 29.36) | 2.93 (-18.8, 30.48) |
| Ecuador | 25 to 29 | -0.62 (-9.88, 9.61) | -0.25 (-10.67, 11.4) | -2.11 (-21.18, 21.57) |
| Ecuador | 30 to 34 | -0.99 (-8.36, 6.99) | -0.7 (-9.17, 8.56) | -2.05 (-16.51, 14.91) |
| Ecuador | 35 to 39 | -0.85 (-6.82, 5.5) | -0.62 (-7.53, 6.81) | -1.53 (-12.95, 11.4) |
| Ecuador | 40 to 44 | -0.51 (-5.36, 4.58) | -0.35 (-6.02, 5.65) | -0.85 (-9.94, 9.17) |
| Ecuador | 45 to 49 | 0.17 (-3.69, 4.19) | 0.31 (-4.25, 5.08) | -0.03 (-7.21, 7.7) |
| Ecuador | 50 to 54 | 1.06 (-2.13, 4.36) | 1.14 (-2.67, 5.11) | 0.95 (-4.84, 7.08) |
| Ecuador | 55 to 59 | 1.69 (-1.08, 4.53) | 1.71 (-1.68, 5.22) | 1.68 (-3.03, 6.62) |
| Ecuador | 60 to 64 | 1.77 (-0.84, 4.44) | 1.79 (-1.44, 5.12) | 1.77 (-2.58, 6.32) |
| Ecuador | 65 to 69 | 1.37 (-1.28, 4.08) | 1.38 (-1.89, 4.76) | 1.4 (-3.03, 6.03) |
| Ecuador | 70 to 74 | 1.01 (-1.84, 3.93) | 1.08 (-2.44, 4.72) | 0.97 (-3.8, 5.97) |
| Ecuador | 75 to 79 | 0.71 (-2.48, 4.01) | 0.86 (-3.11, 4.99) | 0.55 (-4.74, 6.13) |
| Ecuador | 80 to 84 | 0.55 (-3.36, 4.61) | 0.87 (-4.16, 6.16) | 0.12 (-6, 6.64) |
| Ecuador | 85 to 89 | 1.3 (-5, 8.01) | 1.83 (-6.49, 10.88) | 0.57 (-8.73, 10.82) |
| Egypt | 25 to 29 | 6.91 (-3.78, 18.79) | 7.02 (-8.12, 24.64) | 6.56 (-7.9, 23.29) |
| Egypt | 30 to 34 | 5.63 (-2.51, 14.45) | 5.5 (-6.15, 18.59) | 5.64 (-5.38, 17.93) |
| Egypt | 35 to 39 | 4.56 (-1.85, 11.39) | 4.22 (-5.03, 14.37) | 4.79 (-3.9, 14.27) |
| Egypt | 40 to 44 | 3.86 (-1.32, 9.31) | 3.38 (-4.11, 11.46) | 4.2 (-2.85, 11.76) |
| Egypt | 45 to 49 | 3.49 (-0.73, 7.89) | 3.26 (-2.92, 9.82) | 3.63 (-2.06, 9.64) |
| Egypt | 50 to 54 | 3.66 (0.15, 7.29) | 3.67 (-1.58, 9.2) | 3.58 (-1.08, 8.46) |
| Egypt | 55 to 59 | 3.55 (0.43, 6.76) | 3.79 (-0.94, 8.75) | 3.32 (-0.79, 7.59) |
| Egypt | 60 to 64 | 3.12 (0.27, 6.05) | 3.54 (-0.86, 8.14) | 2.73 (-0.98, 6.59) |
| Egypt | 65 to 69 | 2.73 (-0.02, 5.56) | 3.22 (-1.15, 7.79) | 2.48 (-1.04, 6.13) |
| Egypt | 70 to 74 | 2.16 (-0.67, 5.07) | 2.76 (-1.89, 7.64) | 2.05 (-1.51, 5.74) |
| Egypt | 75 to 79 | 1.91 (-1.29, 5.22) | 2.72 (-2.68, 8.42) | 1.61 (-2.35, 5.73) |
| Egypt | 80 to 84 | 2.33 (-1.93, 6.78) | 2.59 (-4.28, 9.96) | 2.52 (-2.86, 8.2) |
| Egypt | 85 to 89 | 2.33 (-3.96, 9.02) | 2.06 (-8.25, 13.53) | 3.27 (-4.58, 11.77) |
| El Salvador | 25 to 29 | 5.03 (-6.94, 18.54) | 5.09 (-9.06, 21.45) | 4.99 (-15.79, 30.9) |
| El Salvador | 30 to 34 | 5.41 (-5.11, 17.08) | 5.62 (-6.8, 19.68) | 4.97 (-13.65, 27.6) |
| El Salvador | 35 to 39 | 5.45 (-4.11, 15.96) | 5.79 (-5.65, 18.61) | 5.03 (-11.53, 24.68) |
| El Salvador | 40 to 44 | 5.16 (-3.47, 14.58) | 5.56 (-4.95, 17.24) | 4.78 (-9.69, 21.57) |
| El Salvador | 45 to 49 | 4.75 (-2.96, 13.07) | 5.08 (-4.46, 15.57) | 4.52 (-8.01, 18.76) |
| El Salvador | 50 to 54 | 4.35 (-2.5, 11.67) | 4.54 (-4.07, 13.94) | 4.24 (-6.63, 16.38) |
| El Salvador | 55 to 59 | 3.87 (-2.34, 10.47) | 3.95 (-4, 12.55) | 4.01 (-5.66, 14.68) |
| El Salvador | 60 to 64 | 3.42 (-2.38, 9.57) | 3.38 (-4.13, 11.47) | 3.65 (-5.27, 13.42) |
| El Salvador | 65 to 69 | 2.91 (-2.76, 8.9) | 2.76 (-4.61, 10.7) | 3.22 (-5.44, 12.67) |
| El Salvador | 70 to 74 | 2.57 (-3.2, 8.69) | 2.35 (-5.2, 10.49) | 2.93 (-5.82, 12.5) |
| El Salvador | 75 to 79 | 2.61 (-3.72, 9.36) | 2.23 (-6.04, 11.24) | 3.21 (-6.42, 13.84) |
| El Salvador | 80 to 84 | 2.75 (-5.21, 11.38) | 2.36 (-8.11, 14.02) | 3.33 (-8.54, 16.72) |
| El Salvador | 85 to 89 | 3.45 (-9.77, 18.6) | 2.88 (-13.72, 22.67) | 4.32 (-16.11, 29.72) |
| Equatorial Guinea | 25 to 29 | -1.11 (-24.82, 30.06) | -7.85 (-31.32, 23.65) | -5.85 (-29.54, 25.79) |
| Equatorial Guinea | 30 to 34 | -3.38 (-22.08, 19.8) | -9.86 (-30.97, 17.7) | -6.69 (-26.32, 18.18) |
| Equatorial Guinea | 35 to 39 | -4.04 (-22.35, 18.6) | -10.64 (-31.78, 17.05) | -7.26 (-26.43, 16.92) |
| Equatorial Guinea | 40 to 44 | -3.4 (-22.43, 20.29) | -10.21 (-31.57, 17.81) | -7.28 (-26.63, 17.17) |
| Equatorial Guinea | 45 to 49 | -2.3 (-22.17, 22.64) | -8.75 (-28.94, 17.18) | -7.08 (-25.88, 16.49) |
| Equatorial Guinea | 50 to 54 | 0.73 (-22.22, 30.44) | -6.59 (-27.49, 20.32) | -5.79 (-24.51, 17.59) |
| Equatorial Guinea | 55 to 59 | 3.2 (-22.34, 37.16) | -3.02 (-28.67, 31.86) | -4.78 (-23.8, 18.99) |
| Equatorial Guinea | 60 to 64 | 3.73 (-23.35, 40.37) | 0.2 (-29.58, 42.57) | -4.43 (-24.08, 20.3) |
| Equatorial Guinea | 65 to 69 | 4.49 (-24.24, 44.12) | 2.5 (-30.88, 52.01) | -4.28 (-24.43, 21.24) |
| Equatorial Guinea | 70 to 74 | 4.9 (-25.77, 48.24) | -1.04 (-30.22, 40.35) | -3.93 (-24.99, 23.03) |
| Equatorial Guinea | 75 to 79 | 5.25 (-29.67, 57.49) | -7.14 (-30.62, 24.27) | -5.21 (-24.21, 18.54) |
| Equatorial Guinea | 80 to 84 | 0.69 (-26.24, 37.44) | -10.72 (-34.02, 20.8) | -6.1 (-25.33, 18.07) |
| Equatorial Guinea | 85 to 89 | -3.88 (-28.54, 29.3) | -11.4 (-39.87, 30.56) | -7.01 (-30.84, 25.02) |
| Eritrea | 25 to 29 | -13.42 (-53.98, 62.87) | -9.5 (-49.82, 63.21) | -3.14 (-24.98, 25.07) |
| Eritrea | 30 to 34 | -10.6 (-42.71, 39.51) | -4.78 (-24.01, 19.3) | -3.65 (-22.68, 20.07) |
| Eritrea | 35 to 39 | -6.15 (-30.24, 26.26) | -5.76 (-29.46, 25.9) | -5.05 (-26.12, 22.04) |
| Eritrea | 40 to 44 | -4.62 (-30.03, 30.02) | -5.02 (-32.19, 33.03) | -5.89 (-28.75, 24.31) |
| Eritrea | 45 to 49 | -2.66 (-27.12, 30.01) | -3.85 (-32.62, 37.19) | -6.91 (-30.78, 25.19) |
| Eritrea | 50 to 54 | -1.29 (-25.08, 30.03) | -2.22 (-30.79, 38.13) | -5.45 (-30.83, 29.25) |
| Eritrea | 55 to 59 | 0.08 (-22.68, 29.53) | -0.15 (-28.13, 38.73) | -3.09 (-29.98, 34.11) |
| Eritrea | 60 to 64 | 1.05 (-21.07, 29.37) | 0.9 (-27.28, 39.99) | -0.6 (-28.88, 38.92) |
| Eritrea | 65 to 69 | 0.74 (-22.44, 30.84) | -0.57 (-29.32, 39.87) | 1.31 (-29.16, 44.87) |
| Eritrea | 70 to 74 | 1.35 (-24.85, 36.69) | -2.43 (-33.27, 42.64) | 2.37 (-31.56, 53.11) |
| Eritrea | 75 to 79 | 2.2 (-31.59, 52.67) | -5.62 (-35.23, 37.52) | 0.82 (-35.67, 58) |
| Eritrea | 80 to 84 | 1.28 (-38.56, 66.96) | -7.89 (-35.33, 31.2) | -5.76 (-35.91, 38.58) |
| Eritrea | 85 to 89 | -6.72 (-39.53, 43.9) | -7.84 (-34.79, 30.25) | -12.18 (-41.46, 31.74) |
| Estonia | 25 to 29 | -5.89 (-54.68, 95.46) | 0.21 (-22.02, 28.77) | -10.32 (-52.19, 68.25) |
| Estonia | 30 to 34 | -7.06 (-45.57, 58.71) | -0.06 (-18.11, 21.97) | -6.12 (-38.61, 43.56) |
| Estonia | 35 to 39 | -4.23 (-41, 55.43) | 0.14 (-17.29, 21.25) | 1.43 (-16.28, 22.9) |
| Estonia | 40 to 44 | -0.13 (-36.41, 56.84) | 0.52 (-16.88, 21.57) | 2.27 (-17.87, 27.35) |
| Estonia | 45 to 49 | 5.19 (-28.42, 54.56) | -1.16 (-20.25, 22.5) | 2.96 (-23.02, 37.7) |
| Estonia | 50 to 54 | 4.64 (-26.52, 49.01) | -0.56 (-26.28, 34.14) | 3.36 (-25.47, 43.34) |
| Estonia | 55 to 59 | 2.13 (-25.17, 39.4) | -0.09 (-29.65, 41.89) | 2.39 (-26.55, 42.72) |
| Estonia | 60 to 64 | 1.68 (-22.31, 33.09) | 0.88 (-30.26, 45.93) | 2.51 (-24.38, 38.97) |
| Estonia | 65 to 69 | 3.28 (-18.43, 30.76) | 3.12 (-28.95, 49.67) | 3.32 (-21.41, 35.82) |
| Estonia | 70 to 74 | 4.74 (-16.21, 30.93) | 5.74 (-29.26, 58.06) | 4.46 (-19.14, 34.94) |
| Estonia | 75 to 79 | 6.32 (-16.51, 35.39) | 4.78 (-31.86, 61.13) | 6.73 (-19.94, 42.29) |
| Estonia | 80 to 84 | 7.76 (-18.12, 41.82) | 5.11 (-35.57, 71.47) | 8.64 (-22.09, 51.5) |
| Estonia | 85 to 89 | 8.49 (-29.9, 67.9) | -3.03 (-34.46, 43.48) | 10.87 (-39.08, 101.81) |
| Eswatini | 25 to 29 | -11.7 (-53.33, 67.06) | -13.28 (-54.05, 63.65) | -1.64 (-24.03, 27.35) |
| Eswatini | 30 to 34 | -11.05 (-43.97, 41.2) | -13.1 (-45.69, 39.05) | -2.9 (-22.72, 21.99) |
| Eswatini | 35 to 39 | -8.6 (-36.35, 31.25) | -10.52 (-40.21, 33.91) | -5.07 (-28.54, 26.1) |
| Eswatini | 40 to 44 | -4.67 (-32.15, 33.96) | -6.5 (-32.95, 30.38) | -6.24 (-32.37, 29.97) |
| Eswatini | 45 to 49 | -0.89 (-27.75, 35.96) | -4.04 (-31.3, 34.04) | -7.29 (-33.38, 29.02) |
| Eswatini | 50 to 54 | 0.21 (-25.5, 34.79) | -2.07 (-30.94, 38.87) | -6.21 (-35.11, 35.57) |
| Eswatini | 55 to 59 | 0.55 (-25.3, 35.33) | 0.34 (-30.69, 45.27) | -3.65 (-35.13, 43.1) |
| Eswatini | 60 to 64 | 1.11 (-24.9, 36.12) | 0.82 (-31.35, 48.06) | -0.99 (-34.95, 50.7) |
| Eswatini | 65 to 69 | 0.59 (-26.71, 38.06) | 0.74 (-33.43, 52.45) | 0.58 (-35.69, 57.31) |
| Eswatini | 70 to 74 | 0.52 (-28.31, 40.93) | -1.11 (-35.56, 51.78) | 1.29 (-36.65, 61.97) |
| Eswatini | 75 to 79 | 0.86 (-31.98, 49.57) | -1.08 (-34.61, 49.63) | 0.01 (-39.76, 66.02) |
| Eswatini | 80 to 84 | 1.42 (-39.96, 71.31) | -2.87 (-30.76, 36.25) | -4.64 (-41.99, 56.73) |
| Eswatini | 85 to 89 | 1.13 (-51.66, 111.59) | -1.39 (-25.17, 29.95) | -8.37 (-45.73, 54.7) |
| Ethiopia | 25 to 29 | 0.39 (-27.4, 38.84) | 0.78 (-37.06, 61.36) | -1.04 (-40.32, 64.1) |
| Ethiopia | 30 to 34 | 0.03 (-19.95, 25.01) | 1.08 (-27.28, 40.5) | -0.41 (-25.87, 33.81) |
| Ethiopia | 35 to 39 | -0.34 (-15.72, 17.84) | 0.38 (-21.38, 28.15) | -0.82 (-20.96, 24.46) |
| Ethiopia | 40 to 44 | -0.53 (-12.79, 13.44) | -0.04 (-17.38, 20.94) | -0.9 (-17.31, 18.75) |
| Ethiopia | 45 to 49 | -0.71 (-10.91, 10.66) | -0.34 (-14.72, 16.46) | -1.11 (-14.94, 14.96) |
| Ethiopia | 50 to 54 | -0.65 (-8.9, 8.34) | -0.45 (-12.27, 12.97) | -0.89 (-11.98, 11.59) |
| Ethiopia | 55 to 59 | -0.49 (-7.18, 6.69) | -0.4 (-10.16, 10.42) | -0.66 (-9.61, 9.19) |
| Ethiopia | 60 to 64 | -0.23 (-6.01, 5.91) | -0.22 (-8.5, 8.8) | -0.28 (-8.18, 8.29) |
| Ethiopia | 65 to 69 | 0.12 (-5.3, 5.85) | 0.06 (-7.49, 8.22) | 0.15 (-7.47, 8.39) |
| Ethiopia | 70 to 74 | 0.59 (-4.84, 6.32) | 0.51 (-6.85, 8.45) | 0.64 (-7.21, 9.14) |
| Ethiopia | 75 to 79 | 0.96 (-5.24, 7.56) | 0.9 (-7.57, 10.16) | 1 (-7.86, 10.72) |
| Ethiopia | 80 to 84 | 1.34 (-7.85, 11.45) | 1.31 (-12.48, 17.27) | 1.45 (-10.57, 15.09) |
| Ethiopia | 85 to 89 | 1.58 (-14.96, 21.33) | 1.57 (-24.93, 37.44) | 1.51 (-17.74, 25.27) |
| Fiji | 25 to 29 | -5.96 (-50.34, 78.08) | -8.77 (-51.86, 72.89) | -7.52 (-49.24, 68.47) |
| Fiji | 30 to 34 | -2.14 (-36.08, 49.84) | -4.58 (-42.17, 57.44) | -3.57 (-29.48, 31.87) |
| Fiji | 35 to 39 | -0.88 (-27.32, 35.19) | -1.42 (-33.63, 46.42) | -4.24 (-34.26, 39.5) |
| Fiji | 40 to 44 | -0.37 (-22.57, 28.2) | 0.78 (-25.92, 37.09) | -1.28 (-27.3, 34.04) |
| Fiji | 45 to 49 | 0.23 (-18.92, 23.91) | 1.03 (-23.51, 33.45) | -0.36 (-25.21, 32.75) |
| Fiji | 50 to 54 | 1.19 (-15.25, 20.83) | 1.27 (-20.66, 29.25) | 0.31 (-22.74, 30.24) |
| Fiji | 55 to 59 | 1.23 (-13.56, 18.56) | 1.49 (-17.93, 25.51) | 1.53 (-19.87, 28.65) |
| Fiji | 60 to 64 | 1.86 (-12.32, 18.34) | 2.14 (-16.32, 24.67) | 1.33 (-19.23, 27.12) |
| Fiji | 65 to 69 | 2.29 (-12.14, 19.09) | 2.6 (-15.97, 25.27) | 1.69 (-19.42, 28.32) |
| Fiji | 70 to 74 | 2.66 (-12.61, 20.59) | 2.71 (-16.8, 26.8) | 2.29 (-20.87, 32.24) |
| Fiji | 75 to 79 | 2.95 (-14.41, 23.84) | 2.91 (-18.66, 30.21) | 3.12 (-23.88, 39.69) |
| Fiji | 80 to 84 | 3.61 (-19.6, 33.5) | 3.58 (-23.74, 40.68) | 4.44 (-34.02, 65.3) |
| Fiji | 85 to 89 | 3.98 (-33.45, 62.47) | 4.9 (-43.32, 94.15) | 3.9 (-46.35, 101.24) |
| Finland | 25 to 29 | -10.66 (-52.7, 68.75) | -0.15 (-21.95, 27.74) | -0.44 (-22.12, 27.28) |
| Finland | 30 to 34 | -10.09 (-43.5, 43.09) | 0.36 (-17.52, 22.13) | 0.19 (-17.67, 21.91) |
| Finland | 35 to 39 | -7.01 (-35.89, 34.88) | 0.53 (-16.13, 20.49) | 0.48 (-16.97, 21.6) |
| Finland | 40 to 44 | -1.12 (-23.44, 27.71) | 0.89 (-16.31, 21.62) | -0.56 (-19.43, 22.74) |
| Finland | 45 to 49 | 1.22 (-21.95, 31.27) | 1.6 (-20.76, 30.26) | -1.72 (-24.03, 27.14) |
| Finland | 50 to 54 | 0.88 (-23.48, 32.99) | 1.32 (-25.3, 37.42) | -3.08 (-26.75, 28.25) |
| Finland | 55 to 59 | 0.87 (-21.43, 29.52) | 0.84 (-26.78, 38.88) | -2.87 (-26.55, 28.44) |
| Finland | 60 to 64 | 0.33 (-18.68, 23.79) | 0.38 (-24.61, 33.65) | -2.08 (-23.7, 25.65) |
| Finland | 65 to 69 | 0.15 (-15.1, 18.14) | -0.35 (-21.1, 25.85) | -0.43 (-19.03, 22.45) |
| Finland | 70 to 74 | 0.35 (-12.1, 14.56) | -0.35 (-17.93, 21) | 0.65 (-15.3, 19.61) |
| Finland | 75 to 79 | 1.33 (-10.09, 14.2) | 0.48 (-16.57, 21.02) | 2.06 (-12.49, 19.03) |
| Finland | 80 to 84 | 2.39 (-8.32, 14.36) | 1.4 (-15.99, 22.4) | 2.96 (-10.31, 18.2) |
| Finland | 85 to 89 | 3.5 (-10.12, 19.18) | 2.55 (-21.38, 33.77) | 4.03 (-11.86, 22.79) |
| France | 25 to 29 | 2.08 (-20.18, 30.56) | 1.12 (-26.04, 38.27) | 3.38 (-31.39, 55.77) |
| France | 30 to 34 | 1.81 (-16.49, 24.13) | 0.73 (-21.78, 29.73) | 3.14 (-26.35, 44.44) |
| France | 35 to 39 | 1.19 (-14.83, 20.22) | 0.19 (-19.59, 24.83) | 2.84 (-23.11, 37.54) |
| France | 40 to 44 | 0.93 (-13.05, 17.17) | -0.11 (-17.33, 20.7) | 2.74 (-19.75, 31.52) |
| France | 45 to 49 | 1.07 (-10.12, 13.65) | 0.03 (-13.94, 16.28) | 2.77 (-15.03, 24.29) |
| France | 50 to 54 | 1.34 (-7.4, 10.9) | 0.36 (-10.85, 12.97) | 2.72 (-10.92, 18.44) |
| France | 55 to 59 | 1.24 (-5.53, 8.5) | 0.55 (-8.23, 10.17) | 2.3 (-8.09, 13.87) |
| France | 60 to 64 | 1.11 (-3.97, 6.45) | 0.63 (-5.98, 7.69) | 1.77 (-6, 10.19) |
| France | 65 to 69 | 0.75 (-3.16, 4.83) | 0.36 (-4.76, 5.76) | 1.22 (-4.75, 7.57) |
| France | 70 to 74 | 0.35 (-2.79, 3.6) | -0.15 (-4.31, 4.19) | 0.81 (-3.93, 5.78) |
| France | 75 to 79 | 0.07 (-2.68, 2.89) | -0.44 (-4.18, 3.44) | 0.45 (-3.56, 4.63) |
| France | 80 to 84 | 0.26 (-1.92, 2.49) | -0.23 (-3.32, 2.96) | 0.53 (-2.53, 3.7) |
| France | 85 to 89 | -0.06 (-2.22, 2.15) | -0.67 (-3.92, 2.7) | 0.18 (-2.69, 3.13) |
| Gabon | 25 to 29 | 2.24 (-21.31, 32.85) | 1.29 (-32.31, 51.56) | 3.07 (-27.21, 45.95) |
| Gabon | 30 to 34 | 2.6 (-18.34, 28.91) | 1.66 (-29.05, 45.68) | 3.53 (-23.78, 40.61) |
| Gabon | 35 to 39 | 2.61 (-17.25, 27.22) | 1.82 (-27.36, 42.73) | 3.55 (-22.82, 38.93) |
| Gabon | 40 to 44 | 2.41 (-16.32, 25.33) | 1.65 (-25.35, 38.41) | 3.36 (-21.87, 36.75) |
| Gabon | 45 to 49 | 1.96 (-15.59, 23.17) | 1.29 (-24.01, 35.02) | 2.99 (-20.78, 33.91) |
| Gabon | 50 to 54 | 1.67 (-14.35, 20.69) | 1.01 (-21.96, 30.75) | 2.72 (-19.23, 30.63) |
| Gabon | 55 to 59 | 1.51 (-13.27, 18.8) | 0.66 (-20.17, 26.94) | 2.68 (-17.6, 27.95) |
| Gabon | 60 to 64 | 1.55 (-12.59, 17.99) | 0.47 (-19.3, 25.09) | 2.65 (-16.5, 26.19) |
| Gabon | 65 to 69 | 1.68 (-12.51, 18.17) | 0.82 (-19.52, 26.28) | 2.69 (-16.17, 25.8) |
| Gabon | 70 to 74 | 2.01 (-12.72, 19.22) | 1.22 (-20.79, 29.33) | 2.88 (-16.17, 26.26) |
| Gabon | 75 to 79 | 2.35 (-13.62, 21.26) | 1.5 (-23.56, 34.78) | 2.75 (-16.87, 27.01) |
| Gabon | 80 to 84 | 2.39 (-16.48, 25.53) | 1.58 (-29.88, 47.14) | 2.23 (-19.57, 29.92) |
| Gabon | 85 to 89 | 2.21 (-22.81, 35.33) | 0.75 (-39.85, 68.77) | 2.06 (-27.2, 43.08) |
| Gambia | 25 to 29 | -11.98 (-53.35, 66.06) | -9.59 (-50.04, 63.61) | -2.37 (-24.06, 25.51) |
| Gambia | 30 to 34 | -10.68 (-42.67, 39.17) | -4.72 (-25.91, 22.53) | -2.47 (-19.75, 18.54) |
| Gambia | 35 to 39 | -7.46 (-31.12, 24.32) | -4.31 (-26.15, 24.01) | -3.12 (-18.98, 15.84) |
| Gambia | 40 to 44 | -8.36 (-34.39, 27.98) | -3.63 (-22.42, 19.71) | -3.72 (-20.01, 15.89) |
| Gambia | 45 to 49 | -7.41 (-34.36, 30.6) | -4.24 (-23.26, 19.5) | -5.67 (-23.07, 15.66) |
| Gambia | 50 to 54 | -8.1 (-33.75, 27.48) | -6.19 (-28.24, 22.63) | -7.17 (-27.35, 18.63) |
| Gambia | 55 to 59 | -5.76 (-33.58, 33.72) | -7.5 (-32.98, 27.69) | -7.93 (-30.62, 22.18) |
| Gambia | 60 to 64 | -3.03 (-33.93, 42.33) | -8.85 (-34.67, 27.15) | -9.02 (-32.11, 21.93) |
| Gambia | 65 to 69 | -1.17 (-34.89, 49.99) | -10.02 (-36.43, 27.36) | -11.15 (-34.78, 21.05) |
| Gambia | 70 to 74 | 0.7 (-35.58, 57.41) | -7.47 (-38.99, 40.32) | -12.87 (-36.78, 20.09) |
| Gambia | 75 to 79 | 1.71 (-36.15, 62.02) | -10.3 (-40.54, 35.31) | -15.87 (-39.8, 17.57) |
| Gambia | 80 to 84 | -0.45 (-41.46, 69.28) | -12.58 (-42.68, 33.32) | -16.52 (-41.69, 19.52) |
| Gambia | 85 to 89 | -8.38 (-43.44, 48.42) | -13.07 (-44.64, 36.53) | -14.88 (-43.24, 27.65) |
| Georgia | 25 to 29 | -6.8 (-49.88, 73.33) | -9.38 (-51.92, 70.8) | 0.26 (-24.05, 32.37) |
| Georgia | 30 to 34 | -3.69 (-36.54, 46.17) | -7.66 (-42.52, 48.35) | -1.47 (-27.2, 33.35) |
| Georgia | 35 to 39 | -0.17 (-30.47, 43.34) | -2.54 (-35.83, 48.01) | -2.22 (-29.14, 34.93) |
| Georgia | 40 to 44 | 3.83 (-23.97, 41.8) | 3.97 (-25.93, 45.94) | -0.25 (-28.89, 39.93) |
| Georgia | 45 to 49 | 6.21 (-19.41, 39.99) | 6.01 (-24.03, 47.93) | 2.24 (-26.88, 42.96) |
| Georgia | 50 to 54 | 6.1 (-16.72, 35.19) | 6.18 (-22.94, 46.3) | 3.11 (-25.5, 42.71) |
| Georgia | 55 to 59 | 5.24 (-15.19, 30.59) | 6.26 (-21.23, 43.35) | 4.23 (-23.06, 41.2) |
| Georgia | 60 to 64 | 5.48 (-13.82, 29.1) | 6.28 (-20.34, 41.81) | 5.86 (-20.29, 40.59) |
| Georgia | 65 to 69 | 6.01 (-12.66, 28.67) | 6.46 (-19.81, 41.32) | 6.33 (-18.88, 39.37) |
| Georgia | 70 to 74 | 7.16 (-13.57, 32.85) | 7.34 (-24.19, 51.98) | 7.68 (-19.54, 44.11) |
| Georgia | 75 to 79 | 8.01 (-16.11, 39.06) | 7.75 (-28.48, 62.32) | 8.67 (-21.67, 50.76) |
| Georgia | 80 to 84 | 8.86 (-24.44, 56.83) | 6.7 (-34.38, 73.51) | 8.91 (-30.98, 71.85) |
| Georgia | 85 to 89 | 9.35 (-40.97, 102.58) | -1.63 (-34.51, 47.76) | 0.97 (-30.35, 46.36) |
| Germany | 25 to 29 | 1.74 (-13.66, 19.89) | 0.82 (-20.28, 27.52) | 3.01 (-18.22, 29.74) |
| Germany | 30 to 34 | 1.1 (-11.05, 14.92) | 0.43 (-15.93, 19.97) | 2.23 (-14.91, 22.83) |
| Germany | 35 to 39 | 0.05 (-10.53, 11.88) | -0.61 (-14.89, 16.06) | 1.14 (-13.87, 18.77) |
| Germany | 40 to 44 | -0.91 (-9.92, 9.01) | -1.44 (-13.6, 12.44) | -0.01 (-12.93, 14.84) |
| Germany | 45 to 49 | -1.07 (-7.97, 6.34) | -1.54 (-10.72, 8.59) | -0.38 (-10.47, 10.84) |
| Germany | 50 to 54 | -1.22 (-6.21, 4.04) | -1.54 (-8.13, 5.53) | -0.81 (-8.29, 7.28) |
| Germany | 55 to 59 | -0.98 (-4.83, 3.02) | -0.99 (-6.09, 4.39) | -0.96 (-6.73, 5.18) |
| Germany | 60 to 64 | -0.89 (-3.92, 2.22) | -0.59 (-4.66, 3.65) | -1.18 (-5.67, 3.53) |
| Germany | 65 to 69 | -0.64 (-3.06, 1.84) | -0.15 (-3.55, 3.36) | -1.06 (-4.53, 2.53) |
| Germany | 70 to 74 | 0.06 (-1.95, 2.11) | 0.41 (-2.56, 3.47) | -0.27 (-3.03, 2.57) |
| Germany | 75 to 79 | 1.41 (-0.29, 3.15) | 1.28 (-1.45, 4.09) | 1.4 (-0.8, 3.66) |
| Germany | 80 to 84 | 2.91 (1.37, 4.46) | 2.12 (-0.45, 4.77) | 3.25 (1.34, 5.2) |
| Germany | 85 to 89 | 5.32 (3.12, 7.57) | 4.09 (0.23, 8.09) | 5.87 (3.2, 8.62) |
| Ghana | 25 to 29 | 9.92 (-10.72, 35.33) | 9.69 (-15.35, 42.15) | 9.52 (-21.75, 53.29) |
| Ghana | 30 to 34 | 9.27 (-10.35, 33.19) | 8.88 (-15.37, 40.07) | 9.23 (-19.23, 47.71) |
| Ghana | 35 to 39 | 8.67 (-9.45, 30.41) | 8.27 (-14.43, 37) | 8.83 (-17.46, 43.48) |
| Ghana | 40 to 44 | 8.14 (-8.42, 27.7) | 7.86 (-13.13, 33.93) | 8.45 (-15.72, 39.56) |
| Ghana | 45 to 49 | 8 (-7.14, 25.6) | 7.67 (-11.45, 30.93) | 8.3 (-14.09, 36.53) |
| Ghana | 50 to 54 | 7.99 (-5.64, 23.59) | 7.59 (-9.41, 27.77) | 8.4 (-12.29, 33.97) |
| Ghana | 55 to 59 | 7.91 (-4.54, 21.98) | 7.52 (-8, 25.67) | 8.41 (-10.96, 31.99) |
| Ghana | 60 to 64 | 7.56 (-3.99, 20.5) | 7.3 (-7.08, 23.92) | 8.17 (-10.23, 30.34) |
| Ghana | 65 to 69 | 7.26 (-4.06, 19.91) | 7.01 (-7.09, 23.25) | 7.83 (-10.3, 29.63) |
| Ghana | 70 to 74 | 6.95 (-4.65, 19.97) | 6.82 (-7.75, 23.68) | 7.56 (-11.15, 30.21) |
| Ghana | 75 to 79 | 6.7 (-6.14, 21.28) | 6.7 (-9.69, 26.06) | 7.23 (-12.98, 32.14) |
| Ghana | 80 to 84 | 6.8 (-10.64, 27.66) | 6.75 (-16.3, 36.16) | 7.18 (-17.75, 39.65) |
| Ghana | 85 to 89 | 6.86 (-21.36, 45.21) | 5.95 (-26.61, 52.96) | 7 (-30.77, 65.38) |
| Greece | 25 to 29 | -0.35 (-39.95, 65.38) | -1.29 (-51.61, 101.35) | 0.59 (-51.72, 109.59) |
| Greece | 30 to 34 | 0.77 (-29.36, 43.74) | -0.64 (-41.09, 67.59) | 0.88 (-41.13, 72.84) |
| Greece | 35 to 39 | 1.7 (-21.89, 32.42) | 1.31 (-29.7, 46.01) | 1.8 (-31.42, 51.11) |
| Greece | 40 to 44 | 1.72 (-17.37, 25.21) | 1.71 (-23.18, 34.66) | 2 (-25.32, 39.31) |
| Greece | 45 to 49 | 1.57 (-13.28, 18.96) | 1.66 (-17.43, 25.17) | 1.5 (-20.13, 28.98) |
| Greece | 50 to 54 | 1.63 (-9.61, 14.27) | 2 (-12.62, 19.08) | 0.83 (-15.84, 20.8) |
| Greece | 55 to 59 | 1.26 (-7.19, 10.47) | 1.91 (-9.18, 14.36) | 0.22 (-12.47, 14.76) |
| Greece | 60 to 64 | 0.69 (-5.59, 7.39) | 1.42 (-7.07, 10.68) | -0.17 (-9.39, 9.99) |
| Greece | 65 to 69 | 0.09 (-4.68, 5.1) | 0.81 (-5.85, 7.93) | -0.57 (-7.34, 6.69) |
| Greece | 70 to 74 | -0.07 (-3.76, 3.76) | 0.6 (-4.8, 6.3) | -0.59 (-5.6, 4.68) |
| Greece | 75 to 79 | -0.54 (-3.56, 2.57) | 0.01 (-4.6, 4.83) | -0.94 (-4.9, 3.19) |
| Greece | 80 to 84 | -1.13 (-3.63, 1.43) | -0.94 (-4.82, 3.11) | -1.29 (-4.51, 2.05) |
| Greece | 85 to 89 | -1.79 (-4.64, 1.13) | -1.89 (-6.37, 2.81) | -1.75 (-5.39, 2.03) |
| Greenland | 25 to 29 | 1.6 (-20.78, 30.3) | 0.96 (-20.85, 28.77) | 0.61 (-21.13, 28.32) |
| Greenland | 30 to 34 | 2.37 (-15.53, 24.06) | 2.22 (-15.53, 23.71) | 1.76 (-15.91, 23.15) |
| Greenland | 35 to 39 | 2.1 (-14.59, 22.07) | 2.36 (-13.98, 21.81) | 1.75 (-14.5, 21.09) |
| Greenland | 40 to 44 | 0.51 (-16.02, 20.3) | 1.25 (-14.43, 19.82) | 0.7 (-14.9, 19.16) |
| Greenland | 45 to 49 | -2.25 (-19.44, 18.61) | -0.49 (-15.72, 17.49) | -1.61 (-16.67, 16.17) |
| Greenland | 50 to 54 | -5.19 (-23.87, 18.09) | -2.26 (-17.11, 15.24) | -3.19 (-17.89, 14.15) |
| Greenland | 55 to 59 | -6.63 (-26.57, 18.72) | -2.8 (-17.56, 14.59) | -3.16 (-17.86, 14.18) |
| Greenland | 60 to 64 | -7.29 (-28.89, 20.87) | -2.88 (-17.63, 14.51) | -1.99 (-16.88, 15.56) |
| Greenland | 65 to 69 | -7.88 (-29.15, 19.78) | -3.28 (-18.08, 14.2) | -1.74 (-16.78, 16.02) |
| Greenland | 70 to 74 | -8.41 (-29, 18.14) | -4.4 (-19.21, 13.13) | -2.43 (-17.55, 15.45) |
| Greenland | 75 to 79 | -7.82 (-28.06, 18.12) | -4.63 (-19.86, 13.5) | -2.88 (-18.39, 15.58) |
| Greenland | 80 to 84 | -6.55 (-26.59, 18.96) | -4.62 (-21.19, 15.42) | -2.73 (-19.62, 17.72) |
| Greenland | 85 to 89 | -4.95 (-26.51, 22.93) | -4.27 (-24.95, 22.11) | -2.6 (-23.64, 24.23) |
| Grenada | 25 to 29 | -0.69 (-23.19, 28.4) | -0.36 (-22.42, 27.96) | -0.03 (-21.87, 27.9) |
| Grenada | 30 to 34 | -1.04 (-19.14, 21.11) | -0.57 (-18.19, 20.85) | -0.57 (-17.93, 20.46) |
| Grenada | 35 to 39 | -1.31 (-20.23, 22.1) | -1.08 (-18.16, 19.57) | -1.28 (-17.23, 17.75) |
| Grenada | 40 to 44 | -3.87 (-25.55, 24.12) | -3.08 (-21.23, 19.26) | -2.2 (-18.09, 16.77) |
| Grenada | 45 to 49 | -7.16 (-30.41, 23.85) | -5.81 (-26.39, 20.51) | -2.64 (-18.93, 16.93) |
| Grenada | 50 to 54 | -9.52 (-35.89, 27.69) | -8.57 (-30.69, 20.61) | -2.91 (-19.64, 17.29) |
| Grenada | 55 to 59 | -12.47 (-37.36, 22.31) | -11.24 (-34.14, 19.61) | -3.32 (-19.44, 16.03) |
| Grenada | 60 to 64 | -12.35 (-38.15, 24.19) | -13.39 (-36.74, 18.58) | -3.52 (-19.16, 15.15) |
| Grenada | 65 to 69 | -8.36 (-37.66, 34.7) | -12.05 (-35.18, 19.32) | -3.14 (-19.64, 16.75) |
| Grenada | 70 to 74 | -4.81 (-36.43, 42.55) | -8.01 (-30.02, 20.93) | -2.68 (-20.36, 18.93) |
| Grenada | 75 to 79 | -1.3 (-33.19, 45.82) | -1.85 (-22.41, 24.15) | -1.52 (-19.57, 20.58) |
| Grenada | 80 to 84 | 0.75 (-27.05, 39.14) | 4.88 (-14.66, 28.9) | -0.13 (-19.1, 23.27) |
| Grenada | 85 to 89 | 1.48 (-22.84, 33.47) | 10.14 (-13.94, 40.95) | 0.87 (-21.22, 29.15) |
| Guam | 25 to 29 | -6.94 (-48.87, 69.38) | -0.1 (-23.85, 31.06) | 0.96 (-21.69, 30.16) |
| Guam | 30 to 34 | -0.75 (-25.9, 32.92) | -0.52 (-25.14, 32.18) | -0.79 (-20.62, 23.98) |
| Guam | 35 to 39 | -0.92 (-26.97, 34.43) | -1.61 (-25.36, 29.7) | -3.7 (-26.04, 25.39) |
| Guam | 40 to 44 | 0.64 (-25.27, 35.55) | -3.57 (-29.52, 31.93) | -5.42 (-30.22, 28.2) |
| Guam | 45 to 49 | 1.91 (-25.24, 38.93) | -4.98 (-29.35, 27.79) | -5.91 (-30.51, 27.41) |
| Guam | 50 to 54 | 2.28 (-25.01, 39.48) | -6.24 (-29.26, 24.29) | -3.15 (-30.02, 34.03) |
| Guam | 55 to 59 | 3.06 (-23.43, 38.7) | -3.52 (-30.44, 33.81) | 0.05 (-29, 40.98) |
| Guam | 60 to 64 | 3.15 (-22.43, 37.16) | -0.3 (-31.51, 45.14) | 1.42 (-29.22, 45.3) |
| Guam | 65 to 69 | 1.15 (-23.14, 33.12) | 2.04 (-32.25, 53.7) | 1.51 (-30.57, 48.42) |
| Guam | 70 to 74 | 0.3 (-25.53, 35.1) | 3.01 (-33.93, 60.63) | 1.74 (-33.17, 54.9) |
| Guam | 75 to 79 | 1.3 (-32.35, 51.67) | -4.47 (-36, 42.59) | 0.45 (-36.77, 59.58) |
| Guam | 80 to 84 | 0.67 (-39, 66.12) | -11.29 (-39.41, 29.88) | -0.89 (-42.27, 70.16) |
| Guam | 85 to 89 | -7.34 (-40.14, 43.46) | -14.16 (-44.23, 32.11) | -10.19 (-46.47, 50.7) |
| Guatemala | 25 to 29 | 4.04 (-7.05, 16.46) | 4.42 (-10.99, 22.49) | 3.61 (-11.58, 21.4) |
| Guatemala | 30 to 34 | 3.87 (-6.32, 15.17) | 4.03 (-10.29, 20.63) | 3.49 (-10.25, 19.34) |
| Guatemala | 35 to 39 | 4.24 (-5.47, 14.95) | 4.38 (-9.46, 20.34) | 3.85 (-9.14, 18.7) |
| Guatemala | 40 to 44 | 4.52 (-4.71, 14.64) | 4.45 (-8.91, 19.76) | 4.31 (-7.95, 18.21) |
| Guatemala | 45 to 49 | 4.77 (-3.88, 14.19) | 4.6 (-8.05, 19) | 4.76 (-6.69, 17.63) |
| Guatemala | 50 to 54 | 4.85 (-3.1, 13.46) | 4.57 (-7.15, 17.77) | 5.07 (-5.47, 16.79) |
| Guatemala | 55 to 59 | 4.57 (-2.81, 12.5) | 4.11 (-6.73, 16.21) | 5 (-4.85, 15.87) |
| Guatemala | 60 to 64 | 4.21 (-2.73, 11.65) | 3.59 (-6.45, 14.71) | 4.75 (-4.65, 15.08) |
| Guatemala | 65 to 69 | 3.57 (-3.27, 10.88) | 2.97 (-6.79, 13.75) | 4.12 (-5.27, 14.43) |
| Guatemala | 70 to 74 | 2.57 (-4.47, 10.13) | 1.96 (-7.94, 12.91) | 3.14 (-6.64, 13.95) |
| Guatemala | 75 to 79 | 1.69 (-6.17, 10.2) | 0.88 (-9.8, 12.82) | 2.33 (-8.72, 14.73) |
| Guatemala | 80 to 84 | 0.83 (-8.86, 11.56) | 0.18 (-13.09, 15.47) | 1.25 (-12.11, 16.63) |
| Guatemala | 85 to 89 | 0.55 (-13.9, 17.42) | 0.24 (-19.95, 25.51) | 0.73 (-18.67, 24.76) |
| Guinea | 25 to 29 | 3.13 (-27.8, 47.32) | -0.44 (-37.24, 57.92) | 3.16 (-38.85, 74.04) |
| Guinea | 30 to 34 | 3.22 (-26.92, 45.79) | -3.37 (-34.08, 41.64) | 2.15 (-35.38, 61.47) |
| Guinea | 35 to 39 | 3.21 (-26.2, 44.34) | -2.4 (-33.21, 42.64) | 1.39 (-34.74, 57.52) |
| Guinea | 40 to 44 | 3.02 (-24.73, 40.98) | -1.26 (-32.88, 45.26) | 1.3 (-33.45, 54.18) |
| Guinea | 45 to 49 | 2.51 (-22.52, 35.62) | -0.45 (-31.58, 44.86) | 1.51 (-30.74, 48.79) |
| Guinea | 50 to 54 | 2.07 (-19.97, 30.18) | 1.39 (-27.75, 42.27) | 1.31 (-27.81, 42.16) |
| Guinea | 55 to 59 | 2.07 (-17.5, 26.29) | 2.71 (-24, 38.8) | 1.57 (-24.79, 37.17) |
| Guinea | 60 to 64 | 2.07 (-15.74, 23.65) | 1.52 (-22.05, 32.22) | 1.37 (-23.45, 34.24) |
| Guinea | 65 to 69 | 2.04 (-14.65, 21.99) | 1.74 (-20.28, 29.85) | 1.41 (-22.27, 32.31) |
| Guinea | 70 to 74 | 1.9 (-14.32, 21.19) | 2.11 (-19.39, 29.35) | 1.52 (-21.76, 31.74) |
| Guinea | 75 to 79 | 1.85 (-15.51, 22.78) | 2.36 (-20.68, 32.09) | 1.57 (-22.77, 33.57) |
| Guinea | 80 to 84 | 1.84 (-19.68, 29.12) | 3.01 (-27.02, 45.38) | 2.03 (-28.11, 44.81) |
| Guinea | 85 to 89 | 1.56 (-27.59, 42.44) | 3.25 (-36.05, 66.71) | 1.8 (-36.93, 64.29) |
| Guinea-Bissau | 25 to 29 | -8.95 (-50, 65.79) | -9.8 (-50.03, 62.81) | -3.04 (-24.02, 23.72) |
| Guinea-Bissau | 30 to 34 | -5.38 (-26.59, 21.96) | -3.7 (-23.33, 20.96) | -3.12 (-20.03, 17.37) |
| Guinea-Bissau | 35 to 39 | -6.05 (-29.63, 25.44) | -2.83 (-18.76, 16.21) | -2.92 (-18.52, 15.67) |
| Guinea-Bissau | 40 to 44 | -5.28 (-29.34, 26.97) | -2.95 (-19.34, 16.76) | -2.76 (-18.06, 15.4) |
| Guinea-Bissau | 45 to 49 | -4.82 (-31.71, 32.66) | -3.87 (-21.68, 17.99) | -2.86 (-18.35, 15.57) |
| Guinea-Bissau | 50 to 54 | -7.2 (-33.89, 30.28) | -4.79 (-25.79, 22.14) | -2.99 (-19.1, 16.33) |
| Guinea-Bissau | 55 to 59 | -5.76 (-36.93, 40.8) | -6.84 (-30.58, 25.03) | -3.09 (-19.95, 17.31) |
| Guinea-Bissau | 60 to 64 | -5.36 (-39.72, 48.59) | -9.96 (-33.33, 21.62) | -2.79 (-19.09, 16.8) |
| Guinea-Bissau | 65 to 69 | -4.15 (-40.92, 55.5) | -11.06 (-34.58, 20.92) | -2.41 (-18.38, 16.68) |
| Guinea-Bissau | 70 to 74 | -3.52 (-40.05, 55.29) | -9.72 (-32.37, 20.52) | -2.09 (-17.35, 15.98) |
| Guinea-Bissau | 75 to 79 | -2.31 (-36.47, 50.2) | -7.09 (-28.14, 20.13) | -2.02 (-17.69, 16.64) |
| Guinea-Bissau | 80 to 84 | -4.22 (-32.88, 36.67) | -2.76 (-21.5, 20.45) | -1.9 (-18.95, 18.73) |
| Guinea-Bissau | 85 to 89 | -2.85 (-26.17, 27.86) | -0.49 (-22.44, 27.67) | -2.21 (-23.35, 24.76) |
| Guyana | 25 to 29 | 3.83 (-28.8, 51.42) | 2.04 (-41.37, 77.59) | 3.94 (-37.93, 74.06) |
| Guyana | 30 to 34 | 4.05 (-23.43, 41.38) | 2.6 (-32.85, 56.77) | 4.2 (-33.41, 63.07) |
| Guyana | 35 to 39 | 4.28 (-19.97, 35.87) | 3.04 (-27.93, 47.31) | 5.06 (-28.67, 54.72) |
| Guyana | 40 to 44 | 5.1 (-16.45, 32.2) | 3.96 (-22.48, 39.42) | 5.71 (-25.57, 50.14) |
| Guyana | 45 to 49 | 5.2 (-14.41, 29.3) | 4.59 (-19.66, 36.15) | 6.14 (-23.56, 47.38) |
| Guyana | 50 to 54 | 5.35 (-13.36, 28.1) | 4.98 (-18.31, 34.92) | 6.02 (-22.84, 45.68) |
| Guyana | 55 to 59 | 5.28 (-13.06, 27.49) | 5.11 (-18.11, 34.92) | 5.67 (-23.26, 45.51) |
| Guyana | 60 to 64 | 4.73 (-13.91, 27.41) | 4.97 (-18.79, 35.7) | 5.39 (-24.44, 46.98) |
| Guyana | 65 to 69 | 4.54 (-15.08, 28.68) | 5.09 (-19.93, 37.91) | 4.82 (-25.82, 48.11) |
| Guyana | 70 to 74 | 4.57 (-16.74, 31.33) | 4.64 (-22.65, 41.56) | 4.93 (-26.97, 50.76) |
| Guyana | 75 to 79 | 3.5 (-20.25, 34.32) | 3.77 (-27.98, 49.51) | 4.64 (-30.04, 56.5) |
| Guyana | 80 to 84 | 3.07 (-27.08, 45.69) | 2.98 (-36.97, 68.26) | 4.04 (-36.45, 70.35) |
| Guyana | 85 to 89 | 4.25 (-44.19, 94.71) | 1.94 (-48.89, 103.31) | 3.01 (-47.65, 102.72) |
| Haiti | 25 to 29 | 1.9 (-21.65, 32.53) | 2.58 (-34.54, 60.77) | 2.55 (-26.78, 43.63) |
| Haiti | 30 to 34 | 1.9 (-17.86, 26.41) | 2.68 (-27.09, 44.61) | 2.58 (-23, 36.64) |
| Haiti | 35 to 39 | 1.92 (-15.47, 22.9) | 2.24 (-22.7, 35.23) | 2.06 (-21.57, 32.82) |
| Haiti | 40 to 44 | 1.64 (-13.93, 20.03) | 1.89 (-19.37, 28.75) | 1.62 (-20.26, 29.51) |
| Haiti | 45 to 49 | 1.43 (-12.66, 17.8) | 1.4 (-17.27, 24.29) | 1.39 (-18.94, 26.81) |
| Haiti | 50 to 54 | 1.17 (-11.68, 15.88) | 1.08 (-15.65, 21.13) | 1.07 (-17.81, 24.29) |
| Haiti | 55 to 59 | 0.88 (-11.2, 14.61) | 0.76 (-14.78, 19.13) | 0.96 (-17.2, 23.09) |
| Haiti | 60 to 64 | 0.64 (-11.3, 14.18) | 0.58 (-14.54, 18.38) | 0.83 (-17.47, 23.18) |
| Haiti | 65 to 69 | 0.52 (-11.81, 14.57) | 0.36 (-15.08, 18.59) | 0.9 (-18.55, 25) |
| Haiti | 70 to 74 | 0.5 (-12.94, 16.01) | 0.38 (-16.57, 20.76) | 1.03 (-19.71, 27.12) |
| Haiti | 75 to 79 | 0.64 (-15.36, 19.68) | 0.3 (-20.01, 25.77) | 1.17 (-22.65, 32.32) |
| Haiti | 80 to 84 | 0.65 (-21.11, 28.43) | -0.2 (-26.95, 36.34) | 1.5 (-31.22, 49.79) |
| Haiti | 85 to 89 | 0.19 (-31.8, 47.2) | 0.82 (-45.83, 87.65) | 1.71 (-46.94, 94.97) |
| Honduras | 25 to 29 | 0.26 (-25.5, 34.92) | -1.35 (-40.15, 62.58) | 0.79 (-30.4, 45.98) |
| Honduras | 30 to 34 | 0.66 (-22.38, 30.54) | -1.01 (-36.41, 54.09) | 1.16 (-26.79, 39.76) |
| Honduras | 35 to 39 | 1.73 (-18.77, 27.41) | 0.52 (-30.82, 46.05) | 2.02 (-23.29, 35.67) |
| Honduras | 40 to 44 | 2.3 (-15.75, 24.22) | 1.34 (-26.04, 38.87) | 2.85 (-19.63, 31.63) |
| Honduras | 45 to 49 | 2.84 (-12.95, 21.49) | 1.84 (-21.84, 32.68) | 3.64 (-16.43, 28.53) |
| Honduras | 50 to 54 | 3.2 (-10.81, 19.41) | 2.34 (-18.38, 28.31) | 3.87 (-14.27, 25.84) |
| Honduras | 55 to 59 | 3.42 (-9.42, 18.06) | 2.75 (-16.35, 26.2) | 3.91 (-12.9, 23.98) |
| Honduras | 60 to 64 | 3.59 (-8.69, 17.53) | 3.05 (-15.18, 25.18) | 4.04 (-12.1, 23.14) |
| Honduras | 65 to 69 | 3.7 (-8.62, 17.67) | 3.3 (-15.02, 25.57) | 4.14 (-12.07, 23.33) |
| Honduras | 70 to 74 | 3.87 (-9.27, 18.92) | 3.5 (-15.81, 27.23) | 4.1 (-13.01, 24.59) |
| Honduras | 75 to 79 | 3.92 (-11.3, 21.75) | 3.64 (-18.83, 32.33) | 4.37 (-15.92, 29.55) |
| Honduras | 80 to 84 | 3.73 (-15.6, 27.49) | 3.79 (-25.68, 44.95) | 4.19 (-21.58, 38.45) |
| Honduras | 85 to 89 | 3.47 (-25.09, 42.91) | 2.68 (-36.02, 64.77) | 4.25 (-33.63, 63.74) |
| Hungary | 25 to 29 | 0.03 (-27.81, 38.6) | -0.15 (-35.09, 53.59) | 1.66 (-35.28, 59.69) |
| Hungary | 30 to 34 | -0.51 (-21.45, 26.01) | -1.28 (-29.33, 37.9) | 0.84 (-28, 41.22) |
| Hungary | 35 to 39 | -0.91 (-17.85, 19.52) | -1.22 (-23.23, 27.1) | 0.23 (-23.29, 30.96) |
| Hungary | 40 to 44 | -0.23 (-13.81, 15.49) | -0.73 (-18.62, 21.08) | 0.37 (-19.08, 24.5) |
| Hungary | 45 to 49 | 0.62 (-11.2, 14.02) | 0.07 (-15.75, 18.86) | 1.29 (-15.65, 21.63) |
| Hungary | 50 to 54 | 1.39 (-8.91, 12.85) | 1.06 (-12.83, 17.16) | 1.7 (-12.99, 18.87) |
| Hungary | 55 to 59 | 2.18 (-6.85, 12.08) | 1.93 (-10.55, 16.16) | 2.34 (-10.31, 16.78) |
| Hungary | 60 to 64 | 2.62 (-5.23, 11.11) | 2.64 (-8.48, 15.11) | 2.55 (-8.22, 14.57) |
| Hungary | 65 to 69 | 3.14 (-4.04, 10.86) | 3.36 (-7.08, 14.97) | 2.91 (-6.74, 13.57) |
| Hungary | 70 to 74 | 3.92 (-2.83, 11.13) | 4.02 (-6.09, 15.22) | 3.74 (-5.14, 13.45) |
| Hungary | 75 to 79 | 4.69 (-2.65, 12.58) | 4.74 (-6.87, 17.79) | 4.62 (-4.69, 14.82) |
| Hungary | 80 to 84 | 5.28 (-2.39, 13.55) | 5.09 (-7.2, 19) | 5.41 (-4.19, 15.96) |
| Hungary | 85 to 89 | 5.51 (-5, 17.18) | 4.88 (-11.63, 24.47) | 5.89 (-7.29, 20.96) |
| Iceland | 25 to 29 | -0.88 (-22.47, 26.73) | -1.04 (-22.56, 26.46) | -0.78 (-22.36, 26.79) |
| Iceland | 30 to 34 | -0.47 (-17.8, 20.52) | -0.58 (-17.88, 20.37) | -0.38 (-17.72, 20.61) |
| Iceland | 35 to 39 | -0.56 (-16.45, 18.37) | -0.59 (-16.47, 18.31) | -0.47 (-16.37, 18.45) |
| Iceland | 40 to 44 | -0.57 (-16.08, 17.8) | -0.63 (-16.04, 17.61) | -0.7 (-16.1, 17.53) |
| Iceland | 45 to 49 | -1.19 (-16.56, 17.01) | -1.17 (-16.4, 16.84) | -1.36 (-16.57, 16.61) |
| Iceland | 50 to 54 | -2.59 (-17.77, 15.38) | -2.29 (-17.39, 15.56) | -2.35 (-17.43, 15.49) |
| Iceland | 55 to 59 | -3.64 (-19.53, 15.37) | -3.42 (-18.46, 14.4) | -3.26 (-18.33, 14.59) |
| Iceland | 60 to 64 | -3.57 (-21.39, 18.28) | -3.5 (-19.44, 15.59) | -3.29 (-19.27, 15.84) |
| Iceland | 65 to 69 | -3.03 (-24.37, 24.33) | -3.09 (-21.14, 19.11) | -2.79 (-20.9, 19.47) |
| Iceland | 70 to 74 | -1.28 (-27.58, 34.57) | -2.67 (-24.81, 25.98) | -2.27 (-24.5, 26.51) |
| Iceland | 75 to 79 | 1.5 (-30.47, 48.18) | -1.17 (-33.96, 47.88) | -5.34 (-31.22, 30.28) |
| Iceland | 80 to 84 | 2.64 (-33.05, 57.37) | -0.12 (-39.93, 66.09) | -2.91 (-39.69, 56.31) |
| Iceland | 85 to 89 | 4.23 (-44.83, 96.91) | 0.28 (-49.25, 98.15) | -0.8 (-49.47, 94.76) |
| India | 25 to 29 | 1.99 (-1.42, 5.51) | 2.48 (-2.35, 7.54) | 1.51 (-3.25, 6.5) |
| India | 30 to 34 | 2.37 (-0.23, 5.04) | 2.97 (-0.81, 6.89) | 1.79 (-1.78, 5.49) |
| India | 35 to 39 | 2.69 (0.48, 4.94) | 2.97 (-0.23, 6.26) | 2.39 (-0.64, 5.52) |
| India | 40 to 44 | 3.05 (1.17, 4.97) | 3.13 (0.41, 5.91) | 2.95 (0.34, 5.62) |
| India | 45 to 49 | 3.11 (1.51, 4.73) | 3.01 (0.74, 5.34) | 3.17 (0.94, 5.46) |
| India | 50 to 54 | 3 (1.61, 4.4) | 2.89 (0.95, 4.86) | 3.11 (1.14, 5.12) |
| India | 55 to 59 | 2.98 (1.73, 4.24) | 2.75 (1.04, 4.49) | 3.21 (1.4, 5.06) |
| India | 60 to 64 | 2.4 (1.22, 3.59) | 2.33 (0.75, 3.93) | 2.5 (0.75, 4.29) |
| India | 65 to 69 | 2 (0.81, 3.2) | 1.94 (0.37, 3.53) | 2.15 (0.34, 4) |
| India | 70 to 74 | 2 (0.67, 3.35) | 2 (0.25, 3.79) | 2.11 (0.07, 4.18) |
| India | 75 to 79 | 2.3 (0.62, 4) | 2.41 (0.17, 4.7) | 2.28 (-0.25, 4.86) |
| India | 80 to 84 | 2.84 (0.32, 5.42) | 3.08 (-0.19, 6.47) | 2.71 (-1.19, 6.76) |
| India | 85 to 89 | 3.36 (-0.52, 7.39) | 3.79 (-1.31, 9.16) | 2.98 (-2.89, 9.2) |
| Indonesia | 25 to 29 | 3.37 (-17.82, 30.02) | 4.01 (-23.21, 40.87) | 3.52 (-26.51, 45.83) |
| Indonesia | 30 to 34 | 3.48 (-9.68, 18.56) | 3.56 (-13.92, 24.58) | 3.34 (-15.6, 26.54) |
| Indonesia | 35 to 39 | 3.5 (-5.79, 13.72) | 3.49 (-9.15, 17.9) | 3.42 (-9.88, 18.69) |
| Indonesia | 40 to 44 | 3.52 (-3.81, 11.42) | 3.5 (-6.55, 14.62) | 3.59 (-6.82, 15.15) |
| Indonesia | 45 to 49 | 3.45 (-2.69, 9.99) | 3.37 (-5.11, 12.61) | 3.52 (-5.2, 13.04) |
| Indonesia | 50 to 54 | 3.27 (-2.09, 8.93) | 3.17 (-4.3, 11.23) | 3.36 (-4.2, 11.51) |
| Indonesia | 55 to 59 | 3.02 (-1.85, 8.14) | 2.96 (-3.85, 10.24) | 3.09 (-3.78, 10.44) |
| Indonesia | 60 to 64 | 2.76 (-1.86, 7.59) | 2.75 (-3.69, 9.62) | 2.75 (-3.76, 9.71) |
| Indonesia | 65 to 69 | 2.62 (-2.03, 7.5) | 2.64 (-3.86, 9.57) | 2.6 (-3.94, 9.6) |
| Indonesia | 70 to 74 | 2.49 (-2.59, 7.84) | 2.48 (-4.64, 10.14) | 2.46 (-4.61, 10.06) |
| Indonesia | 75 to 79 | 2.44 (-3.64, 8.91) | 2.41 (-6.18, 11.79) | 2.47 (-5.93, 11.62) |
| Indonesia | 80 to 84 | 2.36 (-5.63, 11.02) | 2.36 (-8.98, 15.11) | 2.39 (-8.51, 14.58) |
| Indonesia | 85 to 89 | 2.28 (-9.03, 14.99) | 2.26 (-13.67, 21.14) | 2.32 (-13.01, 20.35) |
| Iran (Islamic Republic of) | 25 to 29 | 2.13 (-11.04, 17.26) | 2.73 (-15.94, 25.55) | 1.62 (-16, 22.93) |
| Iran (Islamic Republic of) | 30 to 34 | 1.65 (-7.94, 12.23) | 2.15 (-11.55, 17.99) | 1.18 (-11.72, 15.97) |
| Iran (Islamic Republic of) | 35 to 39 | 1.25 (-6.53, 9.67) | 1.59 (-9.53, 14.09) | 0.91 (-9.61, 12.66) |
| Iran (Islamic Republic of) | 40 to 44 | 0.87 (-5.79, 8.01) | 1.16 (-8.36, 11.66) | 0.52 (-8.56, 10.49) |
| Iran (Islamic Republic of) | 45 to 49 | 0.5 (-5.17, 6.51) | 0.77 (-7.35, 9.6) | 0.22 (-7.5, 8.6) |
| Iran (Islamic Republic of) | 50 to 54 | 0.12 (-4.75, 5.23) | 0.35 (-6.62, 7.84) | -0.14 (-6.79, 6.99) |
| Iran (Islamic Republic of) | 55 to 59 | -0.17 (-4.43, 4.27) | 0 (-6.1, 6.49) | -0.36 (-6.2, 5.85) |
| Iran (Islamic Republic of) | 60 to 64 | -0.32 (-4.13, 3.64) | -0.22 (-5.7, 5.57) | -0.45 (-5.68, 5.07) |
| Iran (Islamic Republic of) | 65 to 69 | -0.36 (-4, 3.42) | -0.38 (-5.63, 5.17) | -0.41 (-5.39, 4.84) |
| Iran (Islamic Republic of) | 70 to 74 | -0.24 (-3.84, 3.5) | -0.36 (-5.53, 5.09) | -0.22 (-5.18, 5) |
| Iran (Islamic Republic of) | 75 to 79 | -0.07 (-4.02, 4.05) | -0.27 (-5.93, 5.73) | 0.06 (-5.37, 5.81) |
| Iran (Islamic Republic of) | 80 to 84 | 0.01 (-5.86, 6.26) | -0.18 (-9.19, 9.73) | 0.29 (-7.38, 8.59) |
| Iran (Islamic Republic of) | 85 to 89 | -0.16 (-9.46, 10.1) | -0.17 (-14.87, 17.06) | 0.16 (-11.73, 13.65) |
| Iraq | 25 to 29 | 0.13 (-13.9, 16.46) | -0.23 (-18.66, 22.39) | 0.45 (-19.75, 25.74) |
| Iraq | 30 to 34 | 0.49 (-10.91, 13.36) | 0.17 (-15.02, 18.06) | 0.75 (-15.68, 20.39) |
| Iraq | 35 to 39 | 0.59 (-9.42, 11.71) | 0.34 (-13.23, 16.03) | 0.91 (-13.28, 17.43) |
| Iraq | 40 to 44 | 0.6 (-8.27, 10.34) | 0.22 (-12.13, 14.31) | 0.93 (-11.48, 15.08) |
| Iraq | 45 to 49 | 0.42 (-7.58, 9.12) | -0.01 (-11.47, 12.94) | 0.77 (-10.1, 12.96) |
| Iraq | 50 to 54 | 0.36 (-7.14, 8.46) | -0.05 (-10.99, 12.23) | 0.67 (-9.33, 11.76) |
| Iraq | 55 to 59 | 0.37 (-6.93, 8.25) | 0.01 (-10.77, 12.08) | 0.63 (-9.01, 11.29) |
| Iraq | 60 to 64 | 0.36 (-6.93, 8.23) | 0.15 (-10.6, 12.19) | 0.55 (-9.11, 11.23) |
| Iraq | 65 to 69 | 0.62 (-7.01, 8.88) | 0.48 (-10.74, 13.1) | 0.78 (-9.33, 12.03) |
| Iraq | 70 to 74 | 1.1 (-7.17, 10.11) | 0.99 (-11.16, 14.81) | 1.18 (-9.72, 13.41) |
| Iraq | 75 to 79 | 1.61 (-7.9, 12.1) | 1.44 (-12.24, 17.24) | 1.63 (-10.98, 16.03) |
| Iraq | 80 to 84 | 2.06 (-9.92, 15.64) | 1.89 (-15.07, 22.24) | 1.96 (-13.83, 20.63) |
| Iraq | 85 to 89 | 2.32 (-14.2, 22.02) | 2.12 (-21.19, 32.32) | 2.34 (-19.43, 30) |
| Ireland | 25 to 29 | -0.87 (-44.41, 76.79) | -7.61 (-52.3, 78.95) | -10.64 (-52.72, 68.91) |
| Ireland | 30 to 34 | -4.91 (-36.73, 42.89) | -11.13 (-44.89, 43.29) | -12.89 (-45.51, 39.27) |
| Ireland | 35 to 39 | -7.09 (-33.77, 30.35) | -12.68 (-41.84, 31.11) | -12.94 (-41.98, 30.63) |
| Ireland | 40 to 44 | -6.98 (-32.35, 27.91) | -11.24 (-38.83, 28.8) | -10.26 (-37.56, 28.97) |
| Ireland | 45 to 49 | -4.08 (-28.79, 29.21) | -7.59 (-33.24, 27.92) | -4.71 (-29.11, 28.08) |
| Ireland | 50 to 54 | -1.11 (-24.11, 28.86) | -2.13 (-26.72, 30.7) | -2.43 (-25.61, 27.97) |
| Ireland | 55 to 59 | 1.12 (-18.29, 25.16) | -1.06 (-23.95, 28.71) | -0.46 (-22.44, 27.76) |
| Ireland | 60 to 64 | 1.57 (-14.39, 20.5) | 0.08 (-20.03, 25.24) | 1.08 (-18.95, 26.06) |
| Ireland | 65 to 69 | 1.23 (-11.58, 15.89) | 0.28 (-16.86, 20.95) | 1.9 (-15.33, 22.63) |
| Ireland | 70 to 74 | 0.97 (-9.19, 12.26) | 0.38 (-13.49, 16.47) | 1.99 (-12.08, 18.31) |
| Ireland | 75 to 79 | 1.08 (-7.38, 10.32) | 0.25 (-11.46, 13.51) | 1.88 (-9.65, 14.88) |
| Ireland | 80 to 84 | 1.38 (-6.14, 9.5) | 0.67 (-10.29, 12.96) | 1.96 (-8.01, 13.02) |
| Ireland | 85 to 89 | 1.65 (-7.7, 11.93) | 1.48 (-13.28, 18.76) | 1.52 (-10.07, 14.61) |
| Israel | 25 to 29 | 3.36 (-27.48, 47.33) | 3.23 (-34.9, 63.7) | 3.36 (-39.38, 76.24) |
| Israel | 30 to 34 | 2.54 (-26.07, 42.22) | 2.48 (-32.58, 55.77) | 2.22 (-36.24, 63.87) |
| Israel | 35 to 39 | 2.25 (-23.94, 37.45) | 2.08 (-30.56, 50.07) | 1.63 (-33.43, 55.14) |
| Israel | 40 to 44 | 1.84 (-20.42, 30.32) | 1.65 (-26.75, 41.07) | 1.06 (-29.44, 44.76) |
| Israel | 45 to 49 | 1.38 (-16.65, 23.32) | 1.44 (-21.68, 31.39) | 0.63 (-25.14, 35.28) |
| Israel | 50 to 54 | 0.8 (-13.26, 17.14) | 0.74 (-18.25, 24.15) | 0.63 (-19.17, 25.28) |
| Israel | 55 to 59 | 0 (-10.93, 12.27) | 0.44 (-14.37, 17.81) | -0.38 (-15.78, 17.85) |
| Israel | 60 to 64 | -0.36 (-8.8, 8.86) | 0.01 (-11.8, 13.39) | -0.77 (-12.47, 12.48) |
| Israel | 65 to 69 | -0.51 (-7.25, 6.72) | -0.12 (-9.74, 10.53) | -0.9 (-10.15, 9.3) |
| Israel | 70 to 74 | -0.37 (-6.1, 5.71) | 0.01 (-8.24, 9.01) | -0.78 (-8.62, 7.73) |
| Israel | 75 to 79 | -0.1 (-5.28, 5.36) | 0.19 (-7.36, 8.36) | -0.4 (-7.39, 7.11) |
| Israel | 80 to 84 | -0.03 (-4.67, 4.85) | -0.06 (-6.92, 7.32) | -0.02 (-6.24, 6.61) |
| Israel | 85 to 89 | 0.11 (-5.76, 6.36) | -0.18 (-8.82, 9.29) | 0.34 (-7.53, 8.87) |
| Italy | 25 to 29 | -0.06 (-30.96, 44.67) | -0.77 (-39.92, 63.9) | 1.02 (-42.15, 76.43) |
| Italy | 30 to 34 | -0.43 (-25.95, 33.88) | -0.87 (-32.33, 45.22) | -0.39 (-38.1, 60.28) |
| Italy | 35 to 39 | -0.85 (-22.58, 26.98) | -0.98 (-27.92, 36.03) | -1.88 (-35.64, 49.6) |
| Italy | 40 to 44 | -0.96 (-17.82, 19.35) | -0.78 (-22.13, 26.43) | -1.8 (-27.43, 32.87) |
| Italy | 45 to 49 | -1.49 (-14.31, 13.24) | -1.08 (-17.44, 18.53) | -2.48 (-21.92, 21.8) |
| Italy | 50 to 54 | -1.6 (-10.79, 8.54) | -1.19 (-13.07, 12.31) | -2.29 (-16.14, 13.85) |
| Italy | 55 to 59 | -1.78 (-8.6, 5.56) | -1.5 (-10.53, 8.44) | -2.07 (-12.12, 9.12) |
| Italy | 60 to 64 | -1.99 (-7.11, 3.41) | -1.88 (-8.77, 5.53) | -2.1 (-9.56, 5.98) |
| Italy | 65 to 69 | -2.03 (-5.9, 1.99) | -2.17 (-7.47, 3.44) | -1.92 (-7.46, 3.96) |
| Italy | 70 to 74 | -1.7 (-4.64, 1.32) | -2.01 (-6.09, 2.25) | -1.5 (-5.66, 2.85) |
| Italy | 75 to 79 | -1.28 (-3.66, 1.17) | -1.71 (-5.14, 1.84) | -1.01 (-4.31, 2.4) |
| Italy | 80 to 84 | -0.44 (-2.46, 1.62) | -0.99 (-3.96, 2.09) | -0.12 (-2.85, 2.68) |
| Italy | 85 to 89 | 0.46 (-1.93, 2.9) | -0.15 (-3.83, 3.67) | 0.79 (-2.31, 4) |
| Jamaica | 25 to 29 | 2.38 (-31.3, 52.57) | 1.71 (-40.55, 74.01) | 3.26 (-41.36, 81.84) |
| Jamaica | 30 to 34 | 1.49 (-24.35, 36.16) | 0.96 (-31.27, 48.3) | 2.74 (-32.09, 55.44) |
| Jamaica | 35 to 39 | 1.65 (-17.89, 25.85) | 0.69 (-24.21, 33.78) | 2.62 (-25.58, 41.49) |
| Jamaica | 40 to 44 | 1.44 (-14.33, 20.11) | 0.67 (-19.1, 25.26) | 2.28 (-21.68, 33.56) |
| Jamaica | 45 to 49 | 1.12 (-11.62, 15.69) | 0.49 (-15.26, 19.17) | 1.95 (-18.27, 27.18) |
| Jamaica | 50 to 54 | 0.84 (-10.12, 13.14) | 0.23 (-13.43, 16.05) | 1.83 (-15.75, 23.07) |
| Jamaica | 55 to 59 | 0.49 (-9.62, 11.74) | 0.18 (-12.31, 14.44) | 0.92 (-15.55, 20.61) |
| Jamaica | 60 to 64 | 0.04 (-9.7, 10.82) | -0.05 (-11.9, 13.38) | 0.18 (-16.02, 19.5) |
| Jamaica | 65 to 69 | -0.31 (-9.77, 10.14) | -0.41 (-11.73, 12.36) | -0.31 (-16.45, 18.95) |
| Jamaica | 70 to 74 | -0.65 (-9.65, 9.25) | -0.7 (-11.57, 11.5) | -0.92 (-16.06, 16.95) |
| Jamaica | 75 to 79 | -1.12 (-10.05, 8.69) | -1.16 (-12.29, 11.38) | -1.32 (-15.7, 15.52) |
| Jamaica | 80 to 84 | -1.74 (-11.54, 9.15) | -1.42 (-14.37, 13.49) | -2.01 (-16.73, 15.31) |
| Jamaica | 85 to 89 | -2.33 (-16.13, 13.75) | -1.75 (-20.36, 21.21) | -2.86 (-22.28, 21.42) |
| Japan | 25 to 29 | -1.77 (-22.49, 24.49) | -1.69 (-27.22, 32.78) | -1.81 (-33.38, 44.73) |
| Japan | 30 to 34 | -1.76 (-16.18, 15.14) | -1.6 (-19.86, 20.82) | -2.17 (-24.65, 27.02) |
| Japan | 35 to 39 | -1.73 (-12.41, 10.25) | -1.42 (-15.04, 14.39) | -1.96 (-18.22, 17.52) |
| Japan | 40 to 44 | -1.72 (-9.21, 6.38) | -1.3 (-10.81, 9.22) | -2.14 (-13.66, 10.92) |
| Japan | 45 to 49 | -1.27 (-6.47, 4.22) | -0.83 (-7.49, 6.31) | -1.96 (-10.11, 6.92) |
| Japan | 50 to 54 | -0.93 (-4.81, 3.12) | -0.31 (-5.27, 4.91) | -1.86 (-8.03, 4.73) |
| Japan | 55 to 59 | -0.79 (-3.76, 2.28) | -0.04 (-3.88, 3.95) | -1.87 (-6.56, 3.07) |
| Japan | 60 to 64 | -0.57 (-2.81, 1.72) | 0.25 (-2.7, 3.28) | -1.71 (-5.18, 1.89) |
| Japan | 65 to 69 | -0.37 (-2.05, 1.34) | 0.55 (-1.75, 2.9) | -1.51 (-4.02, 1.05) |
| Japan | 70 to 74 | -0.46 (-1.86, 0.96) | 0.58 (-1.46, 2.66) | -1.54 (-3.52, 0.47) |
| Japan | 75 to 79 | -0.45 (-1.66, 0.77) | 0.57 (-1.3, 2.48) | -1.32 (-2.93, 0.31) |
| Japan | 80 to 84 | -0.47 (-1.58, 0.65) | 0.46 (-1.39, 2.36) | -1.11 (-2.51, 0.32) |
| Japan | 85 to 89 | -0.83 (-2.21, 0.57) | -0.02 (-2.51, 2.54) | -1.22 (-2.89, 0.47) |
| Jordan | 25 to 29 | 4.07 (-20.01, 35.39) | 4.67 (-28.74, 53.74) | 3.24 (-28.34, 48.73) |
| Jordan | 30 to 34 | 3.26 (-16.8, 28.15) | 3.96 (-24.38, 42.93) | 2.37 (-23.95, 37.8) |
| Jordan | 35 to 39 | 2.51 (-14.87, 23.45) | 3.34 (-21.72, 36.41) | 1.72 (-21.31, 31.49) |
| Jordan | 40 to 44 | 2.24 (-13.17, 20.39) | 2.56 (-20.26, 31.92) | 2.07 (-17.85, 26.84) |
| Jordan | 45 to 49 | 1.59 (-11.89, 17.12) | 1.92 (-18.56, 27.54) | 1.39 (-15.82, 22.13) |
| Jordan | 50 to 54 | 1.16 (-10.88, 14.82) | 1.53 (-17.07, 24.31) | 0.89 (-14.34, 18.82) |
| Jordan | 55 to 59 | 1.21 (-9.98, 13.8) | 1.62 (-15.76, 22.57) | 0.92 (-13.06, 17.15) |
| Jordan | 60 to 64 | 1.54 (-9.34, 13.73) | 2.24 (-14.54, 22.3) | 1.1 (-12.58, 16.93) |
| Jordan | 65 to 69 | 2.29 (-9.07, 15.07) | 3.31 (-14.31, 24.56) | 1.63 (-12.69, 18.31) |
| Jordan | 70 to 74 | 3.5 (-8.99, 17.69) | 4.51 (-15.58, 29.39) | 2.78 (-12.6, 20.87) |
| Jordan | 75 to 79 | 3.85 (-11.32, 21.62) | 4.81 (-20.38, 37.98) | 3.34 (-14.92, 25.52) |
| Jordan | 80 to 84 | 4.14 (-16.91, 30.53) | 5.88 (-31.6, 63.89) | 3.39 (-20.75, 34.86) |
| Jordan | 85 to 89 | 4.31 (-27.17, 49.39) | 5.71 (-43.72, 98.55) | 3.64 (-33.17, 60.71) |
| Kazakhstan | 25 to 29 | -0.74 (-16.88, 18.53) | -0.94 (-24.07, 29.24) | -0.53 (-21.59, 26.18) |
| Kazakhstan | 30 to 34 | 0.29 (-12.13, 14.47) | 0.15 (-18.06, 22.41) | 0.43 (-15.59, 19.49) |
| Kazakhstan | 35 to 39 | 0.88 (-9.72, 12.72) | 0.84 (-14.56, 19.01) | 0.76 (-13.19, 16.96) |
| Kazakhstan | 40 to 44 | 0.6 (-8.67, 10.81) | 0.71 (-12.77, 16.27) | 0.32 (-12.04, 14.42) |
| Kazakhstan | 45 to 49 | 0.58 (-7.93, 9.87) | 1.03 (-11.21, 14.96) | 0.19 (-11.22, 13.07) |
| Kazakhstan | 50 to 54 | 1.03 (-6.77, 9.48) | 1.88 (-9.48, 14.66) | 0.3 (-10.15, 11.96) |
| Kazakhstan | 55 to 59 | 1.95 (-5.39, 9.87) | 3.09 (-7.7, 15.14) | 1.02 (-8.8, 11.9) |
| Kazakhstan | 60 to 64 | 2.95 (-4.71, 11.23) | 4.36 (-7.09, 17.22) | 1.87 (-8.3, 13.16) |
| Kazakhstan | 65 to 69 | 3.86 (-4.18, 12.58) | 5.41 (-6.86, 19.3) | 2.69 (-7.83, 14.4) |
| Kazakhstan | 70 to 74 | 4.61 (-4.98, 15.16) | 5.99 (-9.41, 24) | 3.46 (-8.57, 17.07) |
| Kazakhstan | 75 to 79 | 4.98 (-6.3, 17.61) | 6.05 (-12.72, 28.87) | 3.93 (-9.67, 19.59) |
| Kazakhstan | 80 to 84 | 5.08 (-8.6, 20.81) | 5.53 (-16.87, 33.98) | 4.46 (-12.08, 24.12) |
| Kazakhstan | 85 to 89 | 5.42 (-16, 32.3) | 5.77 (-31.9, 64.28) | 5.17 (-19.42, 37.26) |
| Kenya | 25 to 29 | 4.64 (-17.28, 32.37) | 4.18 (-25.52, 45.72) | 5.09 (-24.5, 46.28) |
| Kenya | 30 to 34 | 4.13 (-14.53, 26.87) | 4.02 (-21.83, 38.42) | 4.67 (-20.48, 37.76) |
| Kenya | 35 to 39 | 3.89 (-11.94, 22.56) | 3.75 (-17.94, 31.16) | 4.47 (-17.5, 32.29) |
| Kenya | 40 to 44 | 3.74 (-9.69, 19.16) | 3.66 (-14.64, 25.89) | 4.16 (-14.74, 27.24) |
| Kenya | 45 to 49 | 3.75 (-7.77, 16.71) | 3.74 (-12.17, 22.52) | 4.07 (-12.12, 23.25) |
| Kenya | 50 to 54 | 3.85 (-6.08, 14.82) | 3.88 (-10.01, 19.92) | 4.08 (-9.69, 19.95) |
| Kenya | 55 to 59 | 3.88 (-4.8, 13.34) | 3.77 (-8.5, 17.7) | 4.13 (-7.84, 17.65) |
| Kenya | 60 to 64 | 3.74 (-4.15, 12.28) | 3.5 (-7.61, 15.95) | 4.11 (-6.85, 16.36) |
| Kenya | 65 to 69 | 3.67 (-3.98, 11.93) | 3.24 (-7.47, 15.17) | 4.14 (-6.51, 16) |
| Kenya | 70 to 74 | 3.57 (-4.24, 12.01) | 2.9 (-8, 15.1) | 4.07 (-6.76, 16.16) |
| Kenya | 75 to 79 | 3.51 (-5.31, 13.15) | 2.65 (-9.97, 17.05) | 3.98 (-7.96, 17.48) |
| Kenya | 80 to 84 | 3.6 (-7.6, 16.16) | 2.62 (-13.93, 22.36) | 4.03 (-10.69, 21.17) |
| Kenya | 85 to 89 | 3.89 (-12.29, 23.05) | 2.77 (-21.9, 35.24) | 4.2 (-16.15, 29.49) |
| Kiribati | 25 to 29 | -2.07 (-23.98, 26.15) | -2.12 (-23.26, 24.84) | -1.63 (-23.02, 25.7) |
| Kiribati | 30 to 34 | -1.48 (-19.12, 20.02) | -1.7 (-18.77, 18.96) | -1.46 (-18.67, 19.4) |
| Kiribati | 35 to 39 | -2.04 (-19, 18.46) | -1.55 (-17.27, 17.16) | -1.64 (-17.43, 17.18) |
| Kiribati | 40 to 44 | -3.27 (-22.27, 20.37) | -1.89 (-17.09, 16.09) | -2.2 (-17.62, 16.1) |
| Kiribati | 45 to 49 | -6.49 (-28.51, 22.32) | -2.49 (-17.41, 15.13) | -2.88 (-18.63, 15.93) |
| Kiribati | 50 to 54 | -10.67 (-33.69, 20.34) | -2.98 (-17.72, 14.39) | -3.88 (-20.32, 15.95) |
| Kiribati | 55 to 59 | -12.32 (-36.94, 21.91) | -3.04 (-17.76, 14.31) | -5.08 (-22.91, 16.87) |
| Kiribati | 60 to 64 | -13.52 (-38.07, 20.76) | -2.49 (-17.3, 14.98) | -5.26 (-24, 18.1) |
| Kiribati | 65 to 69 | -13.34 (-37.72, 20.6) | -1.8 (-16.83, 15.95) | -4.75 (-24.02, 19.39) |
| Kiribati | 70 to 74 | -10.94 (-34.74, 21.54) | -1.47 (-16.73, 16.59) | -4.01 (-23.19, 19.97) |
| Kiribati | 75 to 79 | -8.1 (-30.84, 22.11) | -1.56 (-17.28, 17.14) | -3.1 (-20.95, 18.79) |
| Kiribati | 80 to 84 | -6.03 (-27.25, 21.4) | -1.71 (-18.78, 18.95) | -2.35 (-19.56, 18.54) |
| Kiribati | 85 to 89 | -3.39 (-24.92, 24.32) | -2.04 (-23.2, 24.95) | -2.72 (-23.85, 24.27) |
| Kuwait | 25 to 29 | 0.46 (-27.23, 38.7) | 1.69 (-32.97, 54.25) | -1.44 (-37.41, 55.18) |
| Kuwait | 30 to 34 | 0.36 (-20.67, 26.97) | 0.83 (-26.8, 38.91) | -1.52 (-29.92, 38.39) |
| Kuwait | 35 to 39 | -0.52 (-19.44, 22.85) | 0.43 (-24.2, 33.05) | -2.33 (-27.79, 32.12) |
| Kuwait | 40 to 44 | -1.13 (-19.16, 20.91) | -0.52 (-24.47, 31.02) | -2.92 (-27, 29.11) |
| Kuwait | 45 to 49 | -1.65 (-18.7, 18.99) | -1.45 (-24.05, 27.88) | -2.84 (-25.94, 27.48) |
| Kuwait | 50 to 54 | -2.05 (-18.6, 17.86) | -2 (-23.74, 25.94) | -2.88 (-25.65, 26.86) |
| Kuwait | 55 to 59 | -1.76 (-18.12, 17.86) | -2.06 (-23.39, 25.21) | -2.26 (-25.23, 27.77) |
| Kuwait | 60 to 64 | -1.16 (-17.03, 17.73) | -1.71 (-22.23, 24.21) | -1.06 (-23.79, 28.44) |
| Kuwait | 65 to 69 | -0.32 (-16.18, 18.54) | -0.73 (-21.01, 24.76) | 0.02 (-22.94, 29.83) |
| Kuwait | 70 to 74 | 0.74 (-15.2, 19.68) | 0.72 (-19.53, 26.07) | 1.13 (-22.75, 32.39) |
| Kuwait | 75 to 79 | 1.61 (-16.21, 23.22) | 1.64 (-21.05, 30.85) | 1.5 (-23.95, 35.49) |
| Kuwait | 80 to 84 | 1.68 (-20.17, 29.5) | 1.63 (-25.17, 38.03) | 1.83 (-31.14, 50.57) |
| Kuwait | 85 to 89 | 2.69 (-33.21, 57.89) | 2.79 (-43.47, 86.91) | 2.4 (-45.18, 91.27) |
| Kyrgyzstan | 25 to 29 | -0.12 (-26.68, 36.06) | 0.15 (-34.19, 52.41) | -0.61 (-38.16, 59.74) |
| Kyrgyzstan | 30 to 34 | -0.32 (-22.83, 28.76) | -0.05 (-29.4, 41.51) | -0.94 (-33.07, 46.62) |
| Kyrgyzstan | 35 to 39 | 0.01 (-20.33, 25.53) | -0.22 (-27.31, 36.96) | -0.46 (-29.24, 40.01) |
| Kyrgyzstan | 40 to 44 | -0.07 (-18.57, 22.62) | -0.26 (-24.95, 32.55) | 0.13 (-25.09, 33.83) |
| Kyrgyzstan | 45 to 49 | 0.11 (-17.44, 21.4) | -0.16 (-24.03, 31.21) | 0.6 (-23.36, 32.05) |
| Kyrgyzstan | 50 to 54 | 0.43 (-16.22, 20.39) | 0.13 (-22.85, 29.95) | 0.87 (-21.87, 30.21) |
| Kyrgyzstan | 55 to 59 | 1.02 (-15.12, 20.21) | 0.03 (-22.38, 28.92) | 1.75 (-20.27, 29.85) |
| Kyrgyzstan | 60 to 64 | 1.6 (-15.19, 21.71) | 0.71 (-22.7, 31.21) | 2.62 (-20.43, 32.35) |
| Kyrgyzstan | 65 to 69 | 2.47 (-15.71, 24.57) | 1.71 (-23.69, 35.56) | 3.39 (-21.78, 36.66) |
| Kyrgyzstan | 70 to 74 | 3.33 (-19.09, 31.97) | 2.4 (-28.67, 47) | 3.55 (-25, 42.98) |
| Kyrgyzstan | 75 to 79 | 4.9 (-25.18, 47.08) | -1.31 (-32.53, 44.34) | 4.66 (-31.01, 58.79) |
| Kyrgyzstan | 80 to 84 | 4.9 (-29.87, 56.91) | -4.28 (-37.26, 46.02) | 4.41 (-37.06, 73.21) |
| Kyrgyzstan | 85 to 89 | 5.01 (-44.55, 98.87) | -6.41 (-40.62, 47.53) | 3.68 (-47.65, 105.33) |
| Lao People's Democratic Republic | 25 to 29 | -8.77 (-50.78, 69.08) | -10.14 (-50.62, 63.52) | -11.94 (-53.29, 66) |
| Lao People's Democratic Republic | 30 to 34 | -2.66 (-34.49, 44.66) | -4.23 (-28.26, 27.85) | -8.38 (-41.34, 43.11) |
| Lao People's Democratic Republic | 35 to 39 | -0.79 (-26.74, 34.37) | -2.45 (-28.06, 32.27) | -2.36 (-27.84, 32.12) |
| Lao People's Democratic Republic | 40 to 44 | 0.55 (-21.49, 28.76) | -0.87 (-26.78, 34.22) | -1.1 (-26.67, 33.38) |
| Lao People's Democratic Republic | 45 to 49 | 1.14 (-18.07, 24.85) | 0.14 (-24.76, 33.28) | -0.41 (-24.86, 32) |
| Lao People's Democratic Republic | 50 to 54 | 1.04 (-15.92, 21.42) | 1.01 (-21.55, 30.06) | 0.15 (-22.1, 28.75) |
| Lao People's Democratic Republic | 55 to 59 | 0.74 (-14.41, 18.58) | 1.11 (-19.86, 27.56) | 1.01 (-19.23, 26.33) |
| Lao People's Democratic Republic | 60 to 64 | 0.87 (-13.61, 17.78) | 0.72 (-19.51, 26.03) | 0.64 (-18.98, 25.01) |
| Lao People's Democratic Republic | 65 to 69 | 1.17 (-13.62, 18.48) | 0.81 (-19.55, 26.34) | 1.06 (-18.82, 25.8) |
| Lao People's Democratic Republic | 70 to 74 | 1.47 (-14.43, 20.33) | 1.12 (-21.07, 29.53) | 1.6 (-19.87, 28.82) |
| Lao People's Democratic Republic | 75 to 79 | 1.83 (-17.03, 24.97) | 1.55 (-24.84, 37.2) | 1.94 (-23.14, 35.19) |
| Lao People's Democratic Republic | 80 to 84 | 2.32 (-23.98, 37.7) | 2.34 (-35.65, 62.74) | 3.04 (-34.71, 62.62) |
| Lao People's Democratic Republic | 85 to 89 | 3.51 (-43.6, 89.96) | 0.91 (-48.22, 96.67) | 2.41 (-47.1, 98.27) |
| Latvia | 25 to 29 | -7.78 (-52.19, 77.86) | 1.7 (-20.89, 30.73) | 1.55 (-21.12, 30.73) |
| Latvia | 30 to 34 | -4.38 (-41.19, 55.48) | 1.42 (-16.37, 23.01) | 1.69 (-16.57, 23.95) |
| Latvia | 35 to 39 | 2.44 (-27.8, 45.35) | 1.48 (-15.14, 21.34) | 1.27 (-16.19, 22.37) |
| Latvia | 40 to 44 | 3.67 (-28.14, 49.55) | 0.99 (-15.85, 21.19) | 0.5 (-19.12, 24.88) |
| Latvia | 45 to 49 | 3.88 (-29.28, 52.59) | -0.64 (-18.38, 20.94) | 0.91 (-25.49, 36.66) |
| Latvia | 50 to 54 | 4.23 (-28.53, 52.01) | -1.52 (-22.01, 24.37) | 2.24 (-28.36, 45.9) |
| Latvia | 55 to 59 | 3.94 (-27.18, 48.35) | 0.12 (-28.24, 39.69) | 3.14 (-28.82, 49.46) |
| Latvia | 60 to 64 | 4.05 (-24.66, 43.72) | 1.08 (-32.24, 50.78) | 4.37 (-26.48, 48.16) |
| Latvia | 65 to 69 | 4.4 (-22.61, 40.85) | 2.84 (-34.17, 60.65) | 4.99 (-24.52, 46.04) |
| Latvia | 70 to 74 | 5.92 (-21.7, 43.29) | -1.11 (-34.72, 49.8) | 5.65 (-25.22, 49.26) |
| Latvia | 75 to 79 | 8.19 (-26.42, 59.1) | -7.21 (-34.42, 31.27) | 7.16 (-28.52, 60.66) |
| Latvia | 80 to 84 | 9.92 (-31.1, 75.35) | -9.85 (-37.71, 30.48) | 8.82 (-32.21, 74.67) |
| Latvia | 85 to 89 | 9.19 (-42.22, 106.34) | -9.31 (-39.55, 36.06) | 8.43 (-42.74, 105.34) |
| Lebanon | 25 to 29 | -0.77 (-29.76, 40.19) | 1.01 (-38.52, 65.94) | -0.54 (-43.64, 75.5) |
| Lebanon | 30 to 34 | -1.09 (-24.8, 30.09) | 0.46 (-33.39, 51.51) | -1.01 (-33.57, 47.49) |
| Lebanon | 35 to 39 | -1.54 (-22.61, 25.28) | 0.18 (-30.04, 43.46) | -1.97 (-30.53, 38.33) |
| Lebanon | 40 to 44 | -1.54 (-20.25, 21.56) | -0.25 (-27.22, 36.71) | -1.88 (-26.52, 31.01) |
| Lebanon | 45 to 49 | -1.57 (-18.02, 18.19) | -0.65 (-24.58, 30.87) | -2 (-23.43, 25.43) |
| Lebanon | 50 to 54 | -1.26 (-15.48, 15.35) | -0.26 (-20.94, 25.82) | -1.73 (-20.33, 21.2) |
| Lebanon | 55 to 59 | -0.86 (-13.46, 13.56) | 0 (-18.45, 22.61) | -1.49 (-17.99, 18.32) |
| Lebanon | 60 to 64 | -0.49 (-11.91, 12.42) | 0.56 (-16.13, 20.57) | -1.4 (-16.58, 16.55) |
| Lebanon | 65 to 69 | -0.13 (-10.46, 11.41) | 1.25 (-13.96, 19.14) | -1.27 (-14.87, 14.51) |
| Lebanon | 70 to 74 | 0.1 (-9.95, 11.28) | 1.49 (-13.62, 19.25) | -1.01 (-14.04, 13.99) |
| Lebanon | 75 to 79 | 0.33 (-10.62, 12.63) | 1.68 (-15.44, 22.27) | -0.76 (-14.58, 15.29) |
| Lebanon | 80 to 84 | 0.56 (-12.77, 15.93) | 1.86 (-19.35, 28.64) | -0.39 (-16.83, 19.3) |
| Lebanon | 85 to 89 | 0.94 (-17.76, 23.91) | 1.78 (-26.32, 40.61) | -0.19 (-22.47, 28.48) |
| Lesotho | 25 to 29 | -8.7 (-42.78, 45.68) | -12.35 (-53.71, 65.96) | -12.55 (-53.58, 64.73) |
| Lesotho | 30 to 34 | -8.91 (-35.81, 29.25) | -12.97 (-45.56, 39.12) | -11.79 (-44.74, 40.81) |
| Lesotho | 35 to 39 | -6.75 (-32.2, 28.26) | -10.92 (-40.39, 33.11) | -9.12 (-37.91, 33.01) |
| Lesotho | 40 to 44 | -1.65 (-27.21, 32.9) | -6.74 (-33.23, 30.24) | -3.52 (-27, 27.52) |
| Lesotho | 45 to 49 | 2.31 (-22.04, 34.27) | -1.08 (-26.63, 33.36) | -2.54 (-25.63, 27.71) |
| Lesotho | 50 to 54 | 4.02 (-18.71, 33.11) | 0.41 (-26.22, 36.63) | 0.13 (-23.85, 31.68) |
| Lesotho | 55 to 59 | 4.83 (-17, 32.4) | 2.37 (-25.47, 40.63) | 2.36 (-23.59, 37.1) |
| Lesotho | 60 to 64 | 4.89 (-16.48, 31.73) | 3.31 (-24.78, 41.89) | 4.5 (-22.45, 40.81) |
| Lesotho | 65 to 69 | 4.49 (-16.37, 30.56) | 3.46 (-24.15, 41.11) | 5.3 (-22.75, 43.54) |
| Lesotho | 70 to 74 | 4.41 (-17.26, 31.77) | 2.86 (-26.45, 43.86) | 5.36 (-22.99, 44.14) |
| Lesotho | 75 to 79 | 4 (-18.98, 33.5) | 2.6 (-30.06, 50.52) | 4.97 (-24.37, 45.68) |
| Lesotho | 80 to 84 | 4.43 (-24.83, 45.07) | 3.32 (-38.87, 74.63) | 5.87 (-30.52, 61.32) |
| Lesotho | 85 to 89 | 6.62 (-43.04, 99.56) | 2.84 (-50.82, 115.05) | 6.9 (-44.5, 105.9) |
| Liberia | 25 to 29 | -3.3 (-38.41, 51.83) | -11.04 (-41.29, 34.81) | -11.8 (-45.71, 43.29) |
| Liberia | 30 to 34 | -5.12 (-32.99, 34.34) | -12.61 (-39.57, 26.39) | -14.32 (-43.54, 30.02) |
| Liberia | 35 to 39 | -3.5 (-33.16, 39.32) | -12.99 (-37.71, 21.54) | -14.88 (-43.42, 28.05) |
| Liberia | 40 to 44 | -1.23 (-32.49, 44.51) | -11.77 (-36.52, 22.61) | -12.98 (-40.37, 26.99) |
| Liberia | 45 to 49 | 0.23 (-31.86, 47.44) | -8.04 (-35.32, 30.74) | -8.1 (-35.01, 29.95) |
| Liberia | 50 to 54 | 2.87 (-29.08, 49.22) | -4.81 (-34.29, 37.9) | -4.53 (-33.66, 37.41) |
| Liberia | 55 to 59 | 2.18 (-28.03, 45.07) | -2.81 (-34.72, 44.69) | -1.76 (-33.77, 45.71) |
| Liberia | 60 to 64 | 1.58 (-27.56, 42.44) | -0.04 (-34.46, 52.44) | 0.96 (-34.15, 54.8) |
| Liberia | 65 to 69 | 1.53 (-26.87, 40.97) | 1.74 (-34.53, 58.1) | 2.44 (-34.84, 61.06) |
| Liberia | 70 to 74 | 1.51 (-26.61, 40.43) | 1.81 (-33.87, 56.73) | 2.28 (-35.92, 63.25) |
| Liberia | 75 to 79 | 1.11 (-27.6, 41.2) | 1.42 (-34.63, 57.34) | 1.63 (-38.16, 67.03) |
| Liberia | 80 to 84 | 1.67 (-33.13, 54.57) | 1.58 (-40.03, 72.05) | 0.65 (-42.67, 76.71) |
| Liberia | 85 to 89 | 2.85 (-46.67, 98.33) | 0.06 (-51.15, 104.98) | -1.21 (-53.34, 109.14) |
| Libya | 25 to 29 | 2.94 (-20.62, 33.48) | 2.03 (-31.18, 51.26) | 3.57 (-26.86, 46.66) |
| Libya | 30 to 34 | 3.08 (-14.71, 24.58) | 2.43 (-23.08, 36.4) | 3.34 (-19.56, 32.78) |
| Libya | 35 to 39 | 2.69 (-12.61, 20.68) | 2.32 (-20.37, 31.46) | 2.74 (-16.75, 26.8) |
| Libya | 40 to 44 | 2.08 (-11.68, 18) | 2.01 (-18.99, 28.46) | 1.92 (-15.37, 22.75) |
| Libya | 45 to 49 | 1.66 (-10.92, 16.01) | 1.7 (-17.95, 26.05) | 1.38 (-14.28, 19.9) |
| Libya | 50 to 54 | 1.24 (-10.45, 14.45) | 1.26 (-17.11, 23.72) | 0.91 (-13.64, 17.91) |
| Libya | 55 to 59 | 1 (-10.08, 13.44) | 0.93 (-16.33, 21.73) | 0.73 (-13.15, 16.83) |
| Libya | 60 to 64 | 0.86 (-9.69, 12.64) | 0.63 (-15.64, 20.05) | 0.75 (-12.61, 16.16) |
| Libya | 65 to 69 | 0.83 (-9.67, 12.54) | 0.47 (-15.36, 19.27) | 0.97 (-12.53, 16.56) |
| Libya | 70 to 74 | 0.77 (-10.12, 12.99) | 0.3 (-15.53, 19.09) | 1.12 (-13.34, 17.99) |
| Libya | 75 to 79 | 1.27 (-11.1, 15.37) | 0.94 (-16.62, 22.18) | 1.6 (-15.01, 21.46) |
| Libya | 80 to 84 | 1.97 (-13.52, 20.23) | 1.9 (-20.24, 30.18) | 2.37 (-18.78, 29.02) |
| Libya | 85 to 89 | 2.43 (-19, 29.53) | 2.16 (-27.11, 43.18) | 2.81 (-26.1, 43.03) |
| Lithuania | 25 to 29 | 0.54 (-40.4, 69.58) | 1.15 (-43.37, 80.68) | 1.28 (-52.43, 115.62) |
| Lithuania | 30 to 34 | 1.67 (-32.75, 53.71) | 1.49 (-38.94, 68.68) | 2.37 (-43.4, 85.14) |
| Lithuania | 35 to 39 | 2.36 (-29.81, 49.25) | 1.37 (-38.37, 66.74) | 2.59 (-39.49, 73.93) |
| Lithuania | 40 to 44 | 1.97 (-28.31, 45.05) | 0.69 (-38.95, 66.07) | 2.5 (-36.92, 66.56) |
| Lithuania | 45 to 49 | 1.94 (-26.36, 41.1) | 1.38 (-37.27, 63.87) | 2.35 (-33.88, 58.45) |
| Lithuania | 50 to 54 | 1.97 (-23.98, 36.79) | 2.08 (-34.69, 59.55) | 1.96 (-31.25, 51.23) |
| Lithuania | 55 to 59 | 1.98 (-21.33, 32.2) | 2.7 (-32.87, 57.11) | 2.14 (-26.52, 41.99) |
| Lithuania | 60 to 64 | 2.28 (-19.02, 29.18) | 3.2 (-30.24, 52.66) | 2.35 (-22.8, 35.71) |
| Lithuania | 65 to 69 | 3.1 (-16.53, 27.36) | 3.46 (-29.56, 51.97) | 2.8 (-20.27, 32.56) |
| Lithuania | 70 to 74 | 3.48 (-15.73, 27.07) | 3.41 (-30, 52.75) | 3.81 (-18.86, 32.81) |
| Lithuania | 75 to 79 | 4.61 (-17.44, 32.56) | 3.6 (-32.52, 59.05) | 5.17 (-21.12, 40.23) |
| Lithuania | 80 to 84 | 5.51 (-20.14, 39.39) | 3.96 (-36.6, 70.47) | 6.3 (-24.22, 49.13) |
| Lithuania | 85 to 89 | 7.34 (-30.95, 66.89) | 5.01 (-46.23, 105.08) | 6.55 (-32.67, 68.6) |
| Luxembourg | 25 to 29 | -1.24 (-22.89, 26.5) | -1.27 (-22.92, 26.44) | -0.99 (-22.81, 26.99) |
| Luxembourg | 30 to 34 | -0.98 (-18.49, 20.3) | -1.01 (-18.37, 20.05) | -1.02 (-18.36, 20) |
| Luxembourg | 35 to 39 | -1.16 (-17.56, 18.49) | -1.14 (-17.17, 17.99) | -1.34 (-17.48, 17.97) |
| Luxembourg | 40 to 44 | -1.83 (-18.65, 18.48) | -1.62 (-17.48, 17.29) | -2.02 (-18.31, 17.52) |
| Luxembourg | 45 to 49 | -3.68 (-21.98, 18.91) | -2.43 (-19.06, 17.62) | -3.07 (-20.54, 18.24) |
| Luxembourg | 50 to 54 | -5.16 (-27.21, 23.58) | -4.23 (-22.6, 18.5) | -4.57 (-24.05, 19.91) |
| Luxembourg | 55 to 59 | -4.92 (-29.56, 28.34) | -5.57 (-28.84, 25.32) | -5.1 (-27.66, 24.49) |
| Luxembourg | 60 to 64 | -4.16 (-29.15, 29.66) | -5.64 (-32.32, 31.56) | -4.24 (-29.89, 30.8) |
| Luxembourg | 65 to 69 | -2.87 (-26.6, 28.52) | -4.9 (-33.71, 36.44) | -3.06 (-29.66, 33.58) |
| Luxembourg | 70 to 74 | -1.42 (-22.74, 25.78) | -3.7 (-32.41, 37.2) | -1.74 (-27.34, 32.89) |
| Luxembourg | 75 to 79 | -1.26 (-20.51, 22.67) | -2.38 (-30.3, 36.72) | -0.78 (-24.18, 29.84) |
| Luxembourg | 80 to 84 | -1.23 (-19.4, 21.05) | -1.7 (-30.05, 38.14) | -0.51 (-22.65, 27.98) |
| Luxembourg | 85 to 89 | -1.07 (-23.49, 27.92) | -1.37 (-39.07, 59.66) | -1.24 (-27.12, 33.82) |
| Madagascar | 25 to 29 | 1.59 (-39.34, 70.15) | -4.59 (-48.07, 75.32) | -9.89 (-51.97, 69.03) |
| Madagascar | 30 to 34 | 1.1 (-32.48, 51.38) | -7.21 (-43.7, 52.93) | -5.02 (-39.95, 50.23) |
| Madagascar | 35 to 39 | 0.7 (-26.54, 38.02) | -4.07 (-35.57, 42.83) | -3.4 (-35.56, 44.79) |
| Madagascar | 40 to 44 | 0.63 (-21.38, 28.8) | -1.98 (-29.46, 36.21) | -0.83 (-28.86, 38.26) |
| Madagascar | 45 to 49 | 0.44 (-18.25, 23.4) | -0.83 (-25.59, 32.17) | 0.59 (-24.11, 33.32) |
| Madagascar | 50 to 54 | 0.37 (-15.47, 19.17) | 0.47 (-20.93, 27.66) | 0.58 (-20.72, 27.61) |
| Madagascar | 55 to 59 | 0.39 (-13.11, 15.99) | 0.19 (-18.61, 23.32) | 0.42 (-17.86, 22.76) |
| Madagascar | 60 to 64 | 0.41 (-11.86, 14.38) | -0.08 (-16.83, 20.04) | 0.48 (-16.49, 20.9) |
| Madagascar | 65 to 69 | 0.33 (-11.58, 13.85) | -0.16 (-16.39, 19.22) | 0.33 (-16.25, 20.2) |
| Madagascar | 70 to 74 | 0.39 (-11.77, 14.22) | 0.02 (-16.32, 19.56) | 0.46 (-16.63, 21.04) |
| Madagascar | 75 to 79 | 0.59 (-13.51, 16.98) | 0.06 (-18.85, 23.37) | 0.77 (-18.89, 25.2) |
| Madagascar | 80 to 84 | 0.77 (-17.11, 22.51) | 0.56 (-25.05, 34.91) | 1.42 (-22.83, 33.28) |
| Madagascar | 85 to 89 | 1.39 (-23.71, 34.76) | 0.76 (-33.52, 52.7) | 1.55 (-29.07, 45.39) |
| Malawi | 25 to 29 | 3.76 (-29.18, 52.02) | -0.02 (-37.53, 60.01) | 1.59 (-43.77, 83.55) |
| Malawi | 30 to 34 | 3.17 (-24.48, 40.94) | -2.31 (-32.63, 41.67) | 1.41 (-35.71, 59.96) |
| Malawi | 35 to 39 | 2.7 (-20.64, 32.9) | -0.8 (-26.94, 34.68) | 1.43 (-31.19, 49.53) |
| Malawi | 40 to 44 | 2.52 (-17.15, 26.87) | 0.4 (-22.68, 30.38) | 1.79 (-26.68, 41.32) |
| Malawi | 45 to 49 | 2.47 (-14.43, 22.72) | 1.36 (-19.6, 27.79) | 2.17 (-22.38, 34.5) |
| Malawi | 50 to 54 | 2.44 (-11.87, 19.07) | 2.02 (-16.61, 24.82) | 2.2 (-18.54, 28.24) |
| Malawi | 55 to 59 | 2.34 (-9.86, 16.19) | 2.53 (-13.84, 22.02) | 2.27 (-15.21, 23.36) |
| Malawi | 60 to 64 | 2.25 (-8.54, 14.3) | 1.84 (-12.46, 18.48) | 2.37 (-13.03, 20.48) |
| Malawi | 65 to 69 | 2.28 (-7.81, 13.48) | 1.81 (-11.86, 17.6) | 2.42 (-11.98, 19.17) |
| Malawi | 70 to 74 | 2.32 (-7.85, 13.62) | 1.85 (-12.08, 17.98) | 2.6 (-11.74, 19.26) |
| Malawi | 75 to 79 | 2.49 (-9.57, 16.16) | 1.95 (-15.31, 22.73) | 2.92 (-13.39, 22.3) |
| Malawi | 80 to 84 | 2.71 (-13.86, 22.46) | 2.02 (-22.83, 34.87) | 3.33 (-17.81, 29.92) |
| Malawi | 85 to 89 | 2.66 (-22.96, 36.79) | 1.82 (-36.03, 62.07) | 3.31 (-28.44, 49.16) |
| Malaysia | 25 to 29 | 3.91 (-18.22, 32.02) | 3.39 (-26.6, 45.63) | 4.55 (-25.74, 47.22) |
| Malaysia | 30 to 34 | 4.03 (-11.15, 21.79) | 3.28 (-17.36, 29.07) | 4.6 (-16.35, 30.79) |
| Malaysia | 35 to 39 | 4.3 (-7.15, 17.15) | 3.52 (-12.13, 21.96) | 4.88 (-11.12, 23.76) |
| Malaysia | 40 to 44 | 4.5 (-4.76, 14.66) | 3.81 (-8.94, 18.35) | 5.04 (-7.85, 19.74) |
| Malaysia | 45 to 49 | 4.28 (-3.34, 12.5) | 3.85 (-6.72, 15.62) | 4.58 (-6.04, 16.4) |
| Malaysia | 50 to 54 | 3.56 (-2.81, 10.35) | 3.28 (-5.71, 13.12) | 3.75 (-5.03, 13.36) |
| Malaysia | 55 to 59 | 2.64 (-2.88, 8.47) | 2.59 (-5.35, 11.19) | 2.71 (-4.83, 10.84) |
| Malaysia | 60 to 64 | 1.85 (-3.25, 7.23) | 1.99 (-5.55, 10.12) | 1.8 (-5.05, 9.14) |
| Malaysia | 65 to 69 | 1.55 (-3.6, 6.98) | 1.57 (-6.18, 9.94) | 1.58 (-5.23, 8.88) |
| Malaysia | 70 to 74 | 0.94 (-4.52, 6.7) | 0.74 (-7.69, 9.93) | 1.14 (-5.9, 8.72) |
| Malaysia | 75 to 79 | 1.44 (-5.17, 8.5) | 0.9 (-9.34, 12.29) | 1.87 (-6.59, 11.1) |
| Malaysia | 80 to 84 | 2.45 (-6.68, 12.48) | 1.44 (-12.38, 17.44) | 3.06 (-8.62, 16.24) |
| Malaysia | 85 to 89 | 2.82 (-9.76, 17.14) | 1.91 (-17.12, 25.32) | 3.36 (-12.64, 22.3) |
| Maldives | 25 to 29 | -4.69 (-25.86, 22.51) | -6.9 (-27.01, 18.76) | -3.32 (-24.2, 23.32) |
| Maldives | 30 to 34 | -5.68 (-22.2, 14.33) | -7.13 (-23.26, 12.39) | -3.92 (-20.61, 16.27) |
| Maldives | 35 to 39 | -5.55 (-20.89, 12.75) | -6.43 (-21.38, 11.35) | -4.06 (-19.38, 14.17) |
| Maldives | 40 to 44 | -6.06 (-21.43, 12.33) | -6.21 (-20.74, 10.99) | -4.69 (-19.45, 12.78) |
| Maldives | 45 to 49 | -7.18 (-23.26, 12.27) | -6.25 (-20.6, 10.69) | -5.16 (-19.68, 11.97) |
| Maldives | 50 to 54 | -6.69 (-24.5, 15.34) | -5.45 (-19.81, 11.48) | -4.77 (-19.23, 12.29) |
| Maldives | 55 to 59 | -4.44 (-25.12, 21.95) | -3.92 (-18.5, 13.28) | -3.88 (-18.47, 13.33) |
| Maldives | 60 to 64 | -3.01 (-24.92, 25.29) | -2.42 (-17.24, 15.06) | -3.02 (-17.75, 14.34) |
| Maldives | 65 to 69 | -7.21 (-30.22, 23.38) | -1.79 (-16.82, 15.96) | -2.99 (-17.84, 14.54) |
| Maldives | 70 to 74 | -11.45 (-36.38, 23.25) | -2.83 (-17.88, 14.99) | -4.72 (-19.48, 12.74) |
| Maldives | 75 to 79 | -15.08 (-40.22, 20.64) | -4.88 (-20.07, 13.19) | -6.7 (-21.6, 11.02) |
| Maldives | 80 to 84 | -15.94 (-40.16, 18.08) | -6.43 (-22.68, 13.23) | -8.25 (-24.19, 11.03) |
| Maldives | 85 to 89 | -12.94 (-37.06, 20.43) | -6.58 (-26.76, 19.16) | -8.89 (-28.57, 16.21) |
| Mali | 25 to 29 | 4.63 (-25.49, 46.92) | -1.15 (-35.68, 51.91) | -0.37 (-36.62, 56.61) |
| Mali | 30 to 34 | 4.37 (-24.25, 43.8) | -3.38 (-32.67, 38.64) | -2.76 (-32.05, 39.17) |
| Mali | 35 to 39 | 4.25 (-22.35, 39.96) | -2.45 (-30.81, 37.52) | -1.94 (-30.36, 38.1) |
| Mali | 40 to 44 | 3.85 (-19.93, 34.69) | -0.4 (-28.93, 39.6) | -0.42 (-27.62, 36.99) |
| Mali | 45 to 49 | 3.69 (-17.16, 29.79) | 1.77 (-25.54, 39.11) | 1.02 (-24.6, 35.35) |
| Mali | 50 to 54 | 3.29 (-14.7, 25.08) | 3.59 (-20.97, 35.79) | 2.18 (-21.47, 32.94) |
| Mali | 55 to 59 | 3.05 (-12.9, 21.93) | 3.52 (-18.94, 32.2) | 3.18 (-18.62, 30.81) |
| Mali | 60 to 64 | 2.92 (-11.81, 20.11) | 2.86 (-17.21, 27.8) | 2.38 (-18.04, 27.89) |
| Mali | 65 to 69 | 2.97 (-11.56, 19.89) | 2.87 (-16.76, 27.14) | 2.38 (-18.34, 28.35) |
| Mali | 70 to 74 | 2.81 (-12.23, 20.43) | 2.8 (-16.9, 27.17) | 2.45 (-19.31, 30.08) |
| Mali | 75 to 79 | 2.94 (-13.56, 22.59) | 2.58 (-18.07, 28.43) | 2.4 (-21.14, 32.96) |
| Mali | 80 to 84 | 2.94 (-17.86, 29.02) | 3.31 (-25.49, 43.22) | 2.99 (-26.63, 44.56) |
| Mali | 85 to 89 | 3.17 (-28.65, 49.17) | 2.95 (-35.55, 64.44) | 4.87 (-43.55, 94.81) |
| Malta | 25 to 29 | -0.92 (-22.67, 26.95) | -1.43 (-23.08, 26.33) | -0.88 (-22.48, 26.75) |
| Malta | 30 to 34 | -0.65 (-18.39, 20.95) | -0.83 (-18.42, 20.54) | -0.7 (-18.12, 20.44) |
| Malta | 35 to 39 | -0.17 (-17.46, 20.74) | -0.07 (-16.59, 19.73) | -0.26 (-16.45, 19.07) |
| Malta | 40 to 44 | -0.2 (-19.23, 23.33) | 0.37 (-16.56, 20.73) | 0.47 (-16.31, 20.61) |
| Malta | 45 to 49 | -0.86 (-21.82, 25.71) | 0.64 (-18.33, 24) | 0.85 (-17.86, 23.81) |
| Malta | 50 to 54 | -2.3 (-24.34, 26.17) | -1.49 (-21.94, 24.32) | -0.4 (-20.16, 24.25) |
| Malta | 55 to 59 | -3.64 (-26.54, 26.4) | -4.83 (-27.71, 25.3) | -2.83 (-24.62, 25.27) |
| Malta | 60 to 64 | -3.82 (-25.39, 23.99) | -5.91 (-29.73, 26) | -3.94 (-26.65, 25.81) |
| Malta | 65 to 69 | -3.52 (-22.9, 20.74) | -6.14 (-29.59, 25.11) | -4.55 (-27.09, 24.94) |
| Malta | 70 to 74 | -2.72 (-19.68, 17.83) | -5.06 (-27.63, 24.55) | -3.95 (-24.83, 22.74) |
| Malta | 75 to 79 | -1.8 (-18.07, 17.7) | -2.43 (-25.71, 28.14) | -2.21 (-22.84, 23.93) |
| Malta | 80 to 84 | -1.59 (-17.97, 18.07) | -1.34 (-25.01, 29.79) | -0.8 (-22.24, 26.54) |
| Malta | 85 to 89 | -0.3 (-23.14, 29.32) | -0.61 (-33.09, 47.63) | 0.24 (-28.66, 40.83) |
| Marshall Islands | 25 to 29 | -0.71 (-22.25, 26.8) | -1.15 (-22.5, 26.09) | -0.82 (-22.24, 26.51) |
| Marshall Islands | 30 to 34 | -1.42 (-18.63, 19.44) | -1.74 (-18.8, 18.91) | -1.45 (-18.57, 19.26) |
| Marshall Islands | 35 to 39 | -1.62 (-17.56, 17.39) | -1.86 (-17.53, 16.79) | -1.8 (-17.48, 16.86) |
| Marshall Islands | 40 to 44 | -1.82 (-17.41, 16.71) | -1.64 (-16.87, 16.4) | -1.97 (-17.15, 16.01) |
| Marshall Islands | 45 to 49 | -2.77 (-18.75, 16.37) | -1.89 (-16.9, 15.84) | -2.54 (-17.46, 15.07) |
| Marshall Islands | 50 to 54 | -5.06 (-22.22, 15.89) | -2.9 (-17.65, 14.49) | -3.59 (-18.23, 13.68) |
| Marshall Islands | 55 to 59 | -6.9 (-26.07, 17.23) | -3.86 (-18.45, 13.35) | -4.31 (-18.83, 12.82) |
| Marshall Islands | 60 to 64 | -7.27 (-27.09, 17.94) | -4.13 (-18.69, 13.04) | -4.19 (-18.74, 12.97) |
| Marshall Islands | 65 to 69 | -5.99 (-24.82, 17.57) | -3.69 (-18.43, 13.72) | -3.34 (-18.13, 14.13) |
| Marshall Islands | 70 to 74 | -3.43 (-20.71, 17.62) | -2.45 (-17.56, 15.43) | -1.47 (-16.73, 16.59) |
| Marshall Islands | 75 to 79 | -0.82 (-16.84, 18.3) | -1.2 (-16.98, 17.57) | 0.25 (-15.76, 19.3) |
| Marshall Islands | 80 to 84 | 0.12 (-17.33, 21.26) | -0.49 (-17.77, 20.42) | 1.06 (-16.49, 22.3) |
| Marshall Islands | 85 to 89 | -0.21 (-21.82, 27.38) | -1.23 (-22.57, 25.98) | 1.15 (-20.7, 29.01) |
| Mauritania | 25 to 29 | -8.1 (-45.09, 53.83) | -9.15 (-49.8, 64.42) | -13.53 (-54.11, 62.94) |
| Mauritania | 30 to 34 | -9.91 (-41.42, 38.53) | -4.85 (-26.29, 22.84) | -12.69 (-45.4, 39.6) |
| Mauritania | 35 to 39 | -9.76 (-39.99, 35.7) | -5.88 (-28.96, 24.71) | -10.27 (-39.26, 32.55) |
| Mauritania | 40 to 44 | -5.63 (-34.93, 36.87) | -6.36 (-31.46, 27.93) | -5.28 (-30.65, 29.36) |
| Mauritania | 45 to 49 | -2.06 (-29.97, 36.97) | -5.96 (-31.58, 29.25) | -3.73 (-30.56, 33.46) |
| Mauritania | 50 to 54 | 0.86 (-24.53, 34.79) | -4.01 (-30.79, 33.15) | -1.46 (-28.48, 35.78) |
| Mauritania | 55 to 59 | 1.26 (-22.37, 32.09) | -1.23 (-29.5, 38.36) | 0.36 (-27.28, 38.51) |
| Mauritania | 60 to 64 | 1.82 (-20.07, 29.7) | 0.93 (-28.1, 41.67) | 1.87 (-24.7, 37.82) |
| Mauritania | 65 to 69 | 1.38 (-19.56, 27.78) | 2.21 (-27.77, 44.62) | 2.08 (-23.94, 37) |
| Mauritania | 70 to 74 | 1.57 (-18.61, 26.74) | 2.52 (-26.83, 43.64) | 1.81 (-23.86, 36.14) |
| Mauritania | 75 to 79 | 1.91 (-18.25, 27.05) | 2.2 (-27.19, 43.46) | 2.39 (-23.24, 36.59) |
| Mauritania | 80 to 84 | 2.61 (-22.56, 35.98) | 2.85 (-35.71, 64.52) | 2.7 (-27.9, 46.3) |
| Mauritania | 85 to 89 | 3.61 (-34.13, 62.96) | 2.05 (-47.11, 96.92) | 3.7 (-44.53, 93.89) |
| Mauritius | 25 to 29 | 4.37 (-38.63, 77.49) | -3.97 (-40.65, 55.37) | -6.54 (-49.84, 74.16) |
| Mauritius | 30 to 34 | 4.66 (-24.88, 45.83) | -0.03 (-34.28, 52.07) | -0.35 (-35.09, 52.97) |
| Mauritius | 35 to 39 | 4.03 (-17.52, 31.23) | 2.25 (-24.48, 38.44) | 1.92 (-25.29, 39.05) |
| Mauritius | 40 to 44 | 3.6 (-13.38, 23.92) | 3.38 (-18.81, 31.64) | 2.93 (-18.64, 30.23) |
| Mauritius | 45 to 49 | 3.72 (-10.56, 20.28) | 4.65 (-14.47, 28.05) | 2.94 (-16.45, 26.81) |
| Mauritius | 50 to 54 | 3.89 (-8.51, 17.96) | 4.85 (-12.56, 25.74) | 2.92 (-13.93, 23.06) |
| Mauritius | 55 to 59 | 4.44 (-6.94, 17.2) | 5.08 (-10.99, 24.06) | 3.5 (-11.77, 21.42) |
| Mauritius | 60 to 64 | 5.4 (-5.3, 17.3) | 5.81 (-9.51, 23.73) | 4.64 (-9.58, 21.1) |
| Mauritius | 65 to 69 | 5.84 (-4.51, 17.33) | 5.88 (-8.88, 23.04) | 5.4 (-8.33, 21.19) |
| Mauritius | 70 to 74 | 6.19 (-4.67, 18.3) | 5.9 (-9.43, 23.83) | 6.18 (-8.4, 23.08) |
| Mauritius | 75 to 79 | 6.14 (-6.49, 20.48) | 5.68 (-12.51, 27.66) | 6.18 (-10.26, 25.64) |
| Mauritius | 80 to 84 | 6.04 (-10.49, 25.61) | 5.57 (-18.95, 37.51) | 5.87 (-14.46, 31.02) |
| Mauritius | 85 to 89 | 6.37 (-21.53, 44.19) | 6.73 (-40.88, 92.67) | 6.24 (-25.56, 51.61) |
| Mexico | 25 to 29 | 4.95 (2.87, 7.08) | 5.51 (2.62, 8.48) | 4.36 (1.34, 7.46) |
| Mexico | 30 to 34 | 3.99 (2.22, 5.79) | 4.57 (2.07, 7.14) | 3.43 (0.94, 5.98) |
| Mexico | 35 to 39 | 3.33 (1.75, 4.94) | 3.97 (1.7, 6.29) | 2.73 (0.54, 4.98) |
| Mexico | 40 to 44 | 2.89 (1.5, 4.3) | 3.63 (1.63, 5.67) | 2.19 (0.26, 4.14) |
| Mexico | 45 to 49 | 2.59 (1.38, 3.82) | 3.46 (1.71, 5.24) | 1.74 (0.07, 3.45) |
| Mexico | 50 to 54 | 2.28 (1.19, 3.38) | 3.2 (1.63, 4.79) | 1.36 (-0.15, 2.89) |
| Mexico | 55 to 59 | 2.03 (1.03, 3.05) | 2.97 (1.52, 4.45) | 1.1 (-0.3, 2.52) |
| Mexico | 60 to 64 | 1.92 (0.94, 2.9) | 2.75 (1.34, 4.17) | 1.09 (-0.25, 2.46) |
| Mexico | 65 to 69 | 1.99 (1, 3) | 2.65 (1.2, 4.12) | 1.36 (-0.02, 2.76) |
| Mexico | 70 to 74 | 2.03 (0.96, 3.11) | 2.46 (0.92, 4.03) | 1.63 (0.16, 3.12) |
| Mexico | 75 to 79 | 2.31 (1.06, 3.57) | 2.6 (0.78, 4.45) | 2.05 (0.34, 3.79) |
| Mexico | 80 to 84 | 1.68 (0.16, 3.23) | 1.86 (-0.38, 4.15) | 1.53 (-0.53, 3.64) |
| Mexico | 85 to 89 | 0.04 (-1.99, 2.12) | 1.07 (-2.22, 4.47) | -0.72 (-3.32, 1.95) |
| Micronesia (Federated States of) | 25 to 29 | -0.54 (-23.08, 28.6) | -0.25 (-21.84, 27.3) | 0.1 (-21.73, 28.02) |
| Micronesia (Federated States of) | 30 to 34 | -0.44 (-18.65, 21.83) | -0.29 (-17.61, 20.67) | 0.2 (-17.33, 21.45) |
| Micronesia (Federated States of) | 35 to 39 | -0.18 (-19.26, 23.42) | -0.13 (-16.17, 18.97) | 0.11 (-16.11, 19.47) |
| Micronesia (Federated States of) | 40 to 44 | -2.36 (-24.12, 25.65) | -0.53 (-16.03, 17.84) | -0.36 (-16.41, 18.77) |
| Micronesia (Federated States of) | 45 to 49 | -6.35 (-29.58, 24.55) | -1.5 (-16.75, 16.55) | -2.31 (-18.43, 16.99) |
| Micronesia (Federated States of) | 50 to 54 | -10.31 (-34.83, 23.44) | -3.03 (-18.43, 15.29) | -4.76 (-22.44, 16.95) |
| Micronesia (Federated States of) | 55 to 59 | -12.72 (-37.92, 22.7) | -3.88 (-19.81, 15.22) | -6.64 (-27.21, 19.73) |
| Micronesia (Federated States of) | 60 to 64 | -12.44 (-39.27, 26.23) | -3.2 (-19.82, 16.87) | -6.83 (-29.19, 22.58) |
| Micronesia (Federated States of) | 65 to 69 | -8.67 (-38.77, 36.22) | -1.46 (-17.66, 17.93) | -6.04 (-28.14, 22.86) |
| Micronesia (Federated States of) | 70 to 74 | -4.88 (-36.92, 43.44) | 0.37 (-15.3, 18.94) | -4.54 (-24.96, 21.42) |
| Micronesia (Federated States of) | 75 to 79 | -4.76 (-33.16, 35.7) | 1.26 (-14.94, 20.56) | -3.3 (-22.91, 21.31) |
| Micronesia (Federated States of) | 80 to 84 | -4.18 (-28.89, 29.11) | 0.61 (-16.88, 21.77) | -2.2 (-20.75, 20.69) |
| Micronesia (Federated States of) | 85 to 89 | -3.23 (-26.09, 26.7) | -0.71 (-22.17, 26.66) | -1.86 (-23.35, 25.66) |
| Monaco | 25 to 29 | 0.34 (-21.53, 28.31) | 0.55 (-21.33, 28.53) | 0.55 (-21.32, 28.52) |
| Monaco | 30 to 34 | 0.77 (-16.78, 22.03) | 1.07 (-16.53, 22.37) | 0.64 (-16.88, 21.86) |
| Monaco | 35 to 39 | 0.47 (-15.6, 19.59) | 0.84 (-15.27, 20.02) | 0.14 (-15.86, 19.19) |
| Monaco | 40 to 44 | -0.36 (-15.9, 18.06) | -0.29 (-15.75, 18.02) | -0.49 (-15.92, 17.78) |
| Monaco | 45 to 49 | -0.72 (-16.16, 17.55) | -0.74 (-16.04, 17.36) | -0.73 (-16.04, 17.36) |
| Monaco | 50 to 54 | -1.39 (-16.92, 17.06) | -1.5 (-16.73, 16.51) | -1.14 (-16.41, 16.92) |
| Monaco | 55 to 59 | -1.38 (-17.89, 18.46) | -1.56 (-17.11, 16.92) | -1.24 (-16.84, 17.29) |
| Monaco | 60 to 64 | -1.46 (-20.32, 21.87) | -1.56 (-18.18, 18.45) | -1.17 (-17.79, 18.81) |
| Monaco | 65 to 69 | -3.06 (-25.01, 25.32) | -1.25 (-20.78, 23.09) | -0.99 (-20.37, 23.1) |
| Monaco | 70 to 74 | -2.45 (-27.44, 31.16) | -0.91 (-28.83, 37.96) | -0.79 (-27.17, 35.14) |
| Monaco | 75 to 79 | -0.92 (-28.48, 37.25) | -0.54 (-34.6, 51.24) | 0.18 (-31.88, 47.32) |
| Monaco | 80 to 84 | 0.46 (-27.65, 39.48) | -0.16 (-38.1, 61.04) | 0.64 (-32.85, 50.84) |
| Monaco | 85 to 89 | 2.26 (-31.85, 53.44) | 1.22 (-48.93, 100.6) | 1.52 (-39.04, 69.05) |
| Mongolia | 25 to 29 | 1.94 (-35.18, 60.33) | 2.86 (-40.93, 79.08) | -5.81 (-54.96, 96.98) |
| Mongolia | 30 to 34 | 2.46 (-30.26, 50.52) | 2.44 (-36.17, 64.41) | -7.52 (-44.92, 55.26) |
| Mongolia | 35 to 39 | 2.27 (-28.34, 45.96) | 2.14 (-34.34, 58.88) | -4.52 (-40.1, 52.2) |
| Mongolia | 40 to 44 | 1.73 (-27.56, 42.87) | 1.95 (-33.65, 56.65) | -2 (-36.21, 50.56) |
| Mongolia | 45 to 49 | 1.26 (-27.49, 41.42) | 1.4 (-33.97, 55.72) | -0.12 (-33.74, 50.55) |
| Mongolia | 50 to 54 | 0.78 (-25.79, 36.86) | 1.71 (-32.96, 54.3) | -0.46 (-33.43, 48.85) |
| Mongolia | 55 to 59 | 0.39 (-25.19, 34.72) | 1.45 (-33.59, 54.98) | -0.46 (-33.83, 49.73) |
| Mongolia | 60 to 64 | 0.23 (-25.23, 34.35) | 3.03 (-34.1, 61.07) | -0.04 (-33.05, 49.26) |
| Mongolia | 65 to 69 | 0.14 (-26.68, 36.77) | 1.58 (-35.85, 60.86) | 1.08 (-34.55, 56.1) |
| Mongolia | 70 to 74 | -0.15 (-28.46, 39.37) | -0.41 (-37.84, 59.57) | -1.2 (-36.37, 53.4) |
| Mongolia | 75 to 79 | 0.37 (-31.4, 46.86) | -2.46 (-38.17, 53.88) | -3.05 (-37.83, 51.19) |
| Mongolia | 80 to 84 | 0.31 (-35.39, 55.73) | -3.01 (-37.12, 49.61) | -3.77 (-38.37, 50.25) |
| Mongolia | 85 to 89 | 0.64 (-49.68, 101.3) | -3.18 (-34.76, 43.69) | -4.35 (-38.01, 47.61) |
| Montenegro | 25 to 29 | -10.11 (-52.17, 68.93) | 0.56 (-22.16, 29.9) | 0.39 (-22.05, 29.29) |
| Montenegro | 30 to 34 | -6.45 (-39.56, 44.8) | 0.32 (-17.99, 22.71) | 0.04 (-17.76, 21.7) |
| Montenegro | 35 to 39 | 0.14 (-23.07, 30.36) | -0.42 (-19.03, 22.46) | -0.06 (-17.16, 20.55) |
| Montenegro | 40 to 44 | 1.35 (-26.23, 39.25) | -1.53 (-23.18, 26.23) | -0.15 (-18.54, 22.4) |
| Montenegro | 45 to 49 | 1.85 (-29.44, 47.04) | -4.08 (-27.21, 26.4) | -1.88 (-22.5, 24.21) |
| Montenegro | 50 to 54 | 1.73 (-30.08, 48.03) | -3.13 (-31.53, 37.06) | -3.21 (-27.21, 28.71) |
| Montenegro | 55 to 59 | 1.52 (-28.55, 44.23) | -1.81 (-33.43, 44.82) | -3.11 (-31.12, 36.28) |
| Montenegro | 60 to 64 | 1.46 (-26.14, 39.36) | 0.21 (-33.35, 50.65) | -1.84 (-32.93, 43.67) |
| Montenegro | 65 to 69 | 1.3 (-25.12, 37.03) | 2.01 (-32.17, 53.43) | -0.44 (-32.53, 46.91) |
| Montenegro | 70 to 74 | 1.82 (-25, 38.22) | 3.74 (-32.97, 60.54) | 1.72 (-31.77, 51.64) |
| Montenegro | 75 to 79 | 2.85 (-27.81, 46.53) | 0.93 (-36.45, 60.32) | 2.33 (-34.03, 58.72) |
| Montenegro | 80 to 84 | 3.38 (-28.77, 50.03) | 1.72 (-39.54, 71.14) | 3.08 (-37.94, 71.19) |
| Montenegro | 85 to 89 | 3.23 (-36.58, 68.03) | 1.74 (-48.95, 102.75) | 3.22 (-47.73, 103.82) |
| Morocco | 25 to 29 | 2.91 (-13.4, 22.28) | 1.39 (-24.16, 35.56) | 3.8 (-16.59, 29.17) |
| Morocco | 30 to 34 | 3.64 (-9.33, 18.46) | 1.86 (-18.59, 27.44) | 4.71 (-11.42, 23.78) |
| Morocco | 35 to 39 | 4.18 (-6.98, 16.67) | 2.26 (-15.52, 23.77) | 5.19 (-8.68, 21.16) |
| Morocco | 40 to 44 | 4.55 (-5.23, 15.32) | 2.6 (-13, 20.99) | 5.5 (-6.7, 19.3) |
| Morocco | 45 to 49 | 4.57 (-4.01, 13.92) | 2.79 (-10.95, 18.64) | 5.48 (-5.3, 17.49) |
| Morocco | 50 to 54 | 4.24 (-3.36, 12.43) | 2.82 (-9.25, 16.49) | 5.08 (-4.54, 15.67) |
| Morocco | 55 to 59 | 3.9 (-3.05, 11.35) | 2.89 (-7.95, 15.02) | 4.59 (-4.34, 14.34) |
| Morocco | 60 to 64 | 3.69 (-2.85, 10.67) | 3.01 (-6.96, 14.04) | 4.17 (-4.34, 13.44) |
| Morocco | 65 to 69 | 3.64 (-2.83, 10.55) | 3.18 (-6.43, 13.78) | 3.96 (-4.6, 13.28) |
| Morocco | 70 to 74 | 3.69 (-2.94, 10.78) | 3.26 (-6.43, 13.95) | 4.02 (-4.89, 13.76) |
| Morocco | 75 to 79 | 3.72 (-3.45, 11.42) | 3.17 (-6.87, 14.31) | 4.17 (-5.84, 15.25) |
| Morocco | 80 to 84 | 3.53 (-5, 12.82) | 2.77 (-8.52, 15.44) | 4.27 (-8.22, 18.47) |
| Morocco | 85 to 89 | 3.54 (-8.6, 17.28) | 2.7 (-13.14, 21.44) | 4.49 (-13.36, 26.02) |
| Mozambique | 25 to 29 | -3.15 (-30.83, 35.62) | -5.29 (-35.4, 38.87) | -13.2 (-53.84, 63.22) |
| Mozambique | 30 to 34 | -2.88 (-26.14, 27.69) | -4.86 (-29.81, 28.94) | -11.13 (-43.76, 40.43) |
| Mozambique | 35 to 39 | -0.08 (-21.55, 27.28) | -1.36 (-26.34, 32.08) | -6.93 (-33.99, 31.24) |
| Mozambique | 40 to 44 | 2.07 (-17.86, 26.84) | 1.14 (-23.09, 33) | -1.12 (-25.02, 30.41) |
| Mozambique | 45 to 49 | 3.31 (-14.64, 25.04) | 2.91 (-19.69, 31.87) | 1.09 (-22.13, 31.24) |
| Mozambique | 50 to 54 | 4.17 (-11.9, 23.18) | 4.38 (-16.21, 30.01) | 2.92 (-18.97, 30.73) |
| Mozambique | 55 to 59 | 4.12 (-10.1, 20.59) | 3.87 (-14.75, 26.57) | 3.77 (-16.84, 29.48) |
| Mozambique | 60 to 64 | 3.69 (-9.46, 18.74) | 3.61 (-13.68, 24.35) | 4.12 (-15.15, 27.77) |
| Mozambique | 65 to 69 | 3.58 (-9.4, 18.41) | 3.64 (-13.72, 24.48) | 3.54 (-14.92, 26.01) |
| Mozambique | 70 to 74 | 3.59 (-9.67, 18.8) | 3.55 (-14.31, 25.14) | 3.56 (-15.07, 26.27) |
| Mozambique | 75 to 79 | 3.68 (-10.89, 20.62) | 3.83 (-16.65, 29.35) | 3.83 (-16.17, 28.61) |
| Mozambique | 80 to 84 | 3.91 (-14.39, 26.13) | 4.5 (-22.95, 41.73) | 4.36 (-19.8, 35.79) |
| Mozambique | 85 to 89 | 4.5 (-21.95, 39.93) | 4.66 (-34.52, 67.31) | 5.01 (-27.87, 52.89) |
| Myanmar | 25 to 29 | 3.05 (-21.16, 34.71) | 4.1 (-33.39, 62.68) | 2.28 (-26.74, 42.77) |
| Myanmar | 30 to 34 | 3.28 (-12.7, 22.18) | 4.15 (-21.18, 37.62) | 2.71 (-16.99, 27.08) |
| Myanmar | 35 to 39 | 3.25 (-8.78, 16.86) | 3.73 (-15.64, 27.54) | 2.96 (-12.01, 20.48) |
| Myanmar | 40 to 44 | 3.29 (-6.6, 14.24) | 3.69 (-11.87, 21.99) | 3.14 (-9.31, 17.3) |
| Myanmar | 45 to 49 | 3.11 (-5.47, 12.46) | 3.25 (-9.95, 18.39) | 3 (-7.98, 15.29) |
| Myanmar | 50 to 54 | 2.83 (-4.78, 11.05) | 2.95 (-8.56, 15.91) | 2.7 (-7.21, 13.67) |
| Myanmar | 55 to 59 | 2.58 (-4.38, 10.04) | 2.7 (-7.56, 14.1) | 2.53 (-6.68, 12.66) |
| Myanmar | 60 to 64 | 2.49 (-4.14, 9.57) | 2.54 (-7.15, 13.24) | 2.5 (-6.38, 12.22) |
| Myanmar | 65 to 69 | 2.71 (-3.95, 9.84) | 2.68 (-7.03, 13.4) | 2.8 (-6.15, 12.61) |
| Myanmar | 70 to 74 | 3.24 (-4.07, 11.1) | 3.13 (-7.6, 15.09) | 3.36 (-6.39, 14.12) |
| Myanmar | 75 to 79 | 3.76 (-5.1, 13.44) | 3.59 (-9.64, 18.76) | 3.97 (-7.69, 17.11) |
| Myanmar | 80 to 84 | 4.17 (-8.25, 18.28) | 3.84 (-14.07, 25.48) | 4.47 (-12.01, 24.05) |
| Myanmar | 85 to 89 | 4.26 (-15.31, 28.36) | 3.96 (-23.97, 42.17) | 4.52 (-20.89, 38.1) |
| Namibia | 25 to 29 | -9.5 (-53.56, 76.34) | -9.35 (-53.59, 77.03) | -1.84 (-23.95, 26.69) |
| Namibia | 30 to 34 | -9.94 (-44.82, 46.98) | -10.15 (-45.18, 47.26) | -2.24 (-19.67, 18.97) |
| Namibia | 35 to 39 | -9.82 (-40.71, 37.17) | -10.76 (-42.28, 37.96) | -2.61 (-19.32, 17.55) |
| Namibia | 40 to 44 | -6.91 (-37.71, 39.11) | -10.51 (-40.52, 34.64) | -3.11 (-21.18, 19.11) |
| Namibia | 45 to 49 | -4.88 (-36.22, 41.87) | -6.87 (-38.23, 40.41) | -5.05 (-25.52, 21.06) |
| Namibia | 50 to 54 | -2 (-33.13, 43.61) | -4.1 (-36.69, 45.27) | -6.78 (-31.31, 26.52) |
| Namibia | 55 to 59 | -0.66 (-31.99, 45.09) | -1.83 (-36.04, 50.69) | -9.64 (-36.03, 27.65) |
| Namibia | 60 to 64 | -0.59 (-30.03, 41.24) | 0.02 (-34.1, 51.8) | -11.1 (-38.36, 28.2) |
| Namibia | 65 to 69 | -1.01 (-30.2, 40.4) | 0.38 (-32.68, 49.7) | -8.85 (-40.61, 39.92) |
| Namibia | 70 to 74 | -0.58 (-30.61, 42.46) | -0.04 (-36.37, 57.03) | -5.61 (-41.6, 52.55) |
| Namibia | 75 to 79 | 0.37 (-35.46, 56.1) | -3.47 (-39.13, 53.08) | -8.12 (-42.91, 47.87) |
| Namibia | 80 to 84 | 0.15 (-41.38, 71.09) | -7.59 (-44.26, 53.22) | -10.52 (-42.71, 39.74) |
| Namibia | 85 to 89 | -6.39 (-42.39, 52.12) | -8.24 (-45.42, 54.24) | -12.47 (-44.73, 38.63) |
| Nauru | 25 to 29 | -0.22 (-21.77, 27.27) | -0.2 (-21.76, 27.3) | -0.28 (-21.82, 27.2) |
| Nauru | 30 to 34 | -0.25 (-17.58, 20.71) | -0.28 (-17.6, 20.68) | -0.25 (-17.57, 20.72) |
| Nauru | 35 to 39 | -0.09 (-16.05, 18.89) | -0.14 (-16.09, 18.83) | -0.05 (-16.01, 18.95) |
| Nauru | 40 to 44 | -0.12 (-15.6, 18.19) | -0.18 (-15.64, 18.12) | -0.07 (-15.55, 18.25) |
| Nauru | 45 to 49 | -0.3 (-15.56, 17.72) | -0.17 (-15.45, 17.87) | -0.42 (-15.66, 17.57) |
| Nauru | 50 to 54 | -0.66 (-15.75, 17.13) | -0.21 (-15.37, 17.66) | -1.11 (-16.13, 16.6) |
| Nauru | 55 to 59 | -1.08 (-16.1, 16.62) | -0.23 (-15.37, 17.63) | -1.92 (-16.81, 15.64) |
| Nauru | 60 to 64 | -1.05 (-16.08, 16.67) | -0.01 (-15.2, 17.89) | -2.03 (-16.91, 15.51) |
| Nauru | 65 to 69 | -0.51 (-15.74, 17.47) | 0.51 (-14.87, 18.67) | -1.45 (-16.54, 16.35) |
| Nauru | 70 to 74 | -0.1 (-15.58, 18.21) | 0.96 (-14.68, 19.47) | -1 (-16.34, 17.14) |
| Nauru | 75 to 79 | -0.26 (-16.18, 18.7) | 0.84 (-15.27, 20) | -1.18 (-16.96, 17.6) |
| Nauru | 80 to 84 | -0.93 (-18.13, 19.9) | 0.47 (-16.98, 21.58) | -2.12 (-19.12, 18.45) |
| Nauru | 85 to 89 | -2.01 (-23.18, 24.99) | -0.07 (-21.65, 27.47) | -4.07 (-24.79, 22.36) |
| Nepal | 25 to 29 | 4.45 (-34.05, 65.43) | -6.1 (-42.86, 54.32) | -2.91 (-45.56, 73.14) |
| Nepal | 30 to 34 | 4.73 (-27.87, 52.05) | -5.86 (-35.24, 36.86) | -4.87 (-36.02, 41.44) |
| Nepal | 35 to 39 | 4.45 (-24.75, 44.99) | -3.56 (-33.08, 39) | -4.7 (-31.09, 31.8) |
| Nepal | 40 to 44 | 4.41 (-20.56, 37.22) | -0.98 (-29.65, 39.38) | -2.04 (-29.09, 35.31) |
| Nepal | 45 to 49 | 3.97 (-17.33, 30.75) | 1.09 (-25.42, 37.01) | 0.15 (-25.89, 35.33) |
| Nepal | 50 to 54 | 3.59 (-14.46, 25.46) | 3.08 (-20.27, 33.25) | 1.61 (-22.38, 33.01) |
| Nepal | 55 to 59 | 3.19 (-12.68, 21.95) | 2.98 (-17.71, 28.86) | 2.62 (-19.66, 31.08) |
| Nepal | 60 to 64 | 2.8 (-11.61, 19.57) | 2.09 (-16.44, 24.72) | 2.36 (-18.65, 28.8) |
| Nepal | 65 to 69 | 2.53 (-11.36, 18.59) | 1.97 (-15.68, 23.32) | 1.96 (-19.12, 28.54) |
| Nepal | 70 to 74 | 2.52 (-12.2, 19.7) | 2.09 (-16.21, 24.39) | 2.34 (-20.53, 31.79) |
| Nepal | 75 to 79 | 2.53 (-14.91, 23.54) | 2.23 (-18.98, 28.99) | 3.52 (-26.07, 44.97) |
| Nepal | 80 to 84 | 2.8 (-21.43, 34.52) | 2.73 (-26.18, 42.96) | 3.49 (-35.18, 65.21) |
| Nepal | 85 to 89 | 3.27 (-33.82, 61.14) | 4.68 (-43.29, 93.23) | 3.19 (-46.66, 99.63) |
| Netherlands | 25 to 29 | 3.69 (-32.02, 58.14) | 3.15 (-45.17, 94.03) | 4.94 (-38.09, 77.9) |
| Netherlands | 30 to 34 | 3.78 (-26.89, 47.32) | 1.51 (-42.7, 79.84) | 4.66 (-34.17, 66.41) |
| Netherlands | 35 to 39 | 3.44 (-24.04, 40.86) | 2.02 (-36.58, 64.14) | 4.55 (-29.79, 55.68) |
| Netherlands | 40 to 44 | 2.85 (-20.37, 32.82) | 2.13 (-29.87, 48.72) | 3.68 (-25.85, 44.98) |
| Netherlands | 45 to 49 | 2.46 (-15.43, 24.12) | 1.83 (-23.01, 34.69) | 2.83 (-20.86, 33.61) |
| Netherlands | 50 to 54 | 2.26 (-11.13, 17.66) | 1.91 (-16.55, 24.45) | 2.53 (-15.73, 24.75) |
| Netherlands | 55 to 59 | 1.86 (-8.46, 13.36) | 1.55 (-12.83, 18.29) | 2.19 (-12.02, 18.69) |
| Netherlands | 60 to 64 | 1.36 (-6.68, 10.1) | 1.01 (-10.42, 13.89) | 1.73 (-9.29, 14.09) |
| Netherlands | 65 to 69 | 1.04 (-5.33, 7.83) | 0.87 (-8.27, 10.92) | 1.24 (-7.46, 10.76) |
| Netherlands | 70 to 74 | 0.97 (-4.11, 6.33) | 0.84 (-6.53, 8.79) | 1.07 (-5.84, 8.48) |
| Netherlands | 75 to 79 | 1.23 (-3.12, 5.77) | 1.1 (-5.38, 8.02) | 1.22 (-4.59, 7.39) |
| Netherlands | 80 to 84 | 1.62 (-2.2, 5.58) | 1.43 (-4.56, 7.8) | 1.61 (-3.31, 6.78) |
| Netherlands | 85 to 89 | 1.89 (-2.64, 6.62) | 1.63 (-5.97, 9.85) | 1.92 (-3.65, 7.81) |
| New Zealand | 25 to 29 | 3.67 (-41.57, 83.94) | -8.87 (-49.82, 65.5) | -10.85 (-51.82, 64.93) |
| New Zealand | 30 to 34 | 4.05 (-33.23, 62.16) | -3.17 (-27.7, 29.69) | -8.01 (-38.13, 36.78) |
| New Zealand | 35 to 39 | 4.17 (-27.35, 49.37) | -3.3 (-29.09, 31.86) | -5.28 (-33.16, 34.23) |
| New Zealand | 40 to 44 | 3.91 (-21.55, 37.64) | -1.43 (-27.22, 33.5) | -0.38 (-25.95, 34.03) |
| New Zealand | 45 to 49 | 3.33 (-16.61, 28.04) | 0.31 (-23.34, 31.26) | 0.9 (-23.06, 32.33) |
| New Zealand | 50 to 54 | 2.41 (-13.64, 21.44) | 1.6 (-18.83, 27.17) | 1.69 (-19.27, 28.09) |
| New Zealand | 55 to 59 | 1.94 (-11.84, 17.86) | 1.57 (-16.72, 23.88) | 1.72 (-17.26, 25.06) |
| New Zealand | 60 to 64 | 2.07 (-10.08, 15.86) | 1.74 (-14.76, 21.44) | 2.32 (-14.46, 22.38) |
| New Zealand | 65 to 69 | 2.5 (-8.61, 14.97) | 1.75 (-13.44, 19.61) | 2.52 (-12.76, 20.49) |
| New Zealand | 70 to 74 | 2.81 (-7.53, 14.3) | 2.12 (-12.21, 18.77) | 2.89 (-11.25, 19.3) |
| New Zealand | 75 to 79 | 2.59 (-7.36, 13.61) | 1.64 (-12.33, 17.83) | 3.06 (-10.51, 18.68) |
| New Zealand | 80 to 84 | 1.3 (-8.96, 12.72) | 0.27 (-14.76, 17.95) | 2.17 (-11.5, 17.95) |
| New Zealand | 85 to 89 | 0.02 (-12.77, 14.69) | -1.47 (-20.08, 21.48) | 1.24 (-15.42, 21.19) |
| Nicaragua | 25 to 29 | 7.77 (-14.67, 36.11) | 8.45 (-19.1, 45.39) | 7.03 (-29.53, 62.55) |
| Nicaragua | 30 to 34 | 7.32 (-12.1, 31.03) | 8.01 (-15.78, 38.51) | 6.1 (-25.71, 51.53) |
| Nicaragua | 35 to 39 | 6.78 (-10.38, 27.22) | 7.31 (-13.69, 33.43) | 5.49 (-22.41, 43.42) |
| Nicaragua | 40 to 44 | 6.35 (-8.87, 24.12) | 6.61 (-12.09, 29.28) | 5.37 (-18.91, 36.92) |
| Nicaragua | 45 to 49 | 6.13 (-7.47, 21.74) | 6.22 (-10.54, 26.13) | 5.55 (-15.73, 32.2) |
| Nicaragua | 50 to 54 | 6.08 (-6.33, 20.14) | 6.01 (-9.48, 24.15) | 6 (-13.08, 29.28) |
| Nicaragua | 55 to 59 | 6.1 (-5.58, 19.22) | 5.98 (-8.88, 23.25) | 6.13 (-11.55, 27.34) |
| Nicaragua | 60 to 64 | 6.13 (-5.33, 18.98) | 6.09 (-8.85, 23.48) | 6.09 (-10.85, 26.26) |
| Nicaragua | 65 to 69 | 6.01 (-5.8, 19.3) | 6.18 (-9.44, 24.49) | 5.83 (-11.54, 26.61) |
| Nicaragua | 70 to 74 | 5.76 (-6.88, 20.11) | 5.96 (-10.73, 25.77) | 5.54 (-12.9, 27.88) |
| Nicaragua | 75 to 79 | 5.28 (-9.11, 21.94) | 5.16 (-13.35, 27.62) | 5.2 (-15.4, 30.82) |
| Nicaragua | 80 to 84 | 4.42 (-13.6, 26.2) | 3.63 (-18.93, 32.46) | 5.19 (-21.78, 41.44) |
| Nicaragua | 85 to 89 | 3.87 (-23.95, 41.85) | 1.71 (-29.84, 47.44) | 4.74 (-33.06, 63.89) |
| Niger | 25 to 29 | 2.57 (-21.05, 33.27) | 3.39 (-27.49, 47.44) | 1.84 (-32.95, 54.68) |
| Niger | 30 to 34 | 2.33 (-20.75, 32.14) | 2.93 (-27.68, 46.5) | 1.97 (-31.15, 51.03) |
| Niger | 35 to 39 | 2.22 (-20.96, 32.21) | 2.92 (-28.33, 47.8) | 1.76 (-30.58, 49.18) |
| Niger | 40 to 44 | 2.03 (-21.13, 31.97) | 2.64 (-29, 48.38) | 1.38 (-30.17, 47.18) |
| Niger | 45 to 49 | 2.18 (-20.39, 31.15) | 2.24 (-28.61, 46.42) | 1.35 (-28.55, 43.76) |
| Niger | 50 to 54 | 2.03 (-18.93, 28.4) | 2.19 (-26.71, 42.47) | 0.96 (-26.49, 38.67) |
| Niger | 55 to 59 | 1.92 (-17.81, 26.38) | 2.37 (-24.72, 39.22) | 0.81 (-25.4, 36.22) |
| Niger | 60 to 64 | 1.81 (-17.6, 25.78) | 2.09 (-23.56, 36.35) | 0.84 (-26.31, 38) |
| Niger | 65 to 69 | 1.73 (-17.51, 25.45) | 2.22 (-22.77, 35.28) | 0.93 (-26.71, 38.98) |
| Niger | 70 to 74 | 1.59 (-18.1, 26.01) | 1.94 (-22.57, 34.22) | 1.24 (-28.54, 43.44) |
| Niger | 75 to 79 | 1.78 (-20.93, 31) | 1.81 (-25.57, 39.26) | 2.11 (-33.15, 55.97) |
| Niger | 80 to 84 | 2.3 (-27.21, 43.77) | 2.74 (-35.49, 63.63) | 2.04 (-38.42, 69.08) |
| Niger | 85 to 89 | 3.36 (-44.31, 91.83) | 2.01 (-47.23, 97.18) | 1.15 (-48.94, 100.35) |
| Nigeria | 25 to 29 | 3.76 (-5.37, 13.77) | 4.38 (-6.95, 17.08) | 2.96 (-12.09, 20.58) |
| Nigeria | 30 to 34 | 3.89 (-4.79, 13.37) | 4.54 (-6.5, 16.88) | 3.01 (-10.66, 18.78) |
| Nigeria | 35 to 39 | 3.76 (-4.56, 12.81) | 4.36 (-6.35, 16.31) | 2.92 (-9.94, 17.62) |
| Nigeria | 40 to 44 | 3.38 (-4.41, 11.8) | 3.9 (-6.28, 15.19) | 2.82 (-9, 16.17) |
| Nigeria | 45 to 49 | 3.01 (-4.14, 10.69) | 3.44 (-6, 13.83) | 2.74 (-7.99, 14.71) |
| Nigeria | 50 to 54 | 2.7 (-3.56, 9.36) | 2.98 (-5.2, 11.87) | 2.74 (-6.85, 13.31) |
| Nigeria | 55 to 59 | 2.52 (-3.14, 8.5) | 2.7 (-4.61, 10.56) | 2.82 (-6.06, 12.53) |
| Nigeria | 60 to 64 | 2.33 (-2.92, 7.86) | 2.45 (-4.19, 9.55) | 2.81 (-5.69, 12.08) |
| Nigeria | 65 to 69 | 2.34 (-2.75, 7.68) | 2.39 (-4.04, 9.26) | 2.69 (-5.51, 11.6) |
| Nigeria | 70 to 74 | 2.4 (-2.66, 7.72) | 2.34 (-4.18, 9.31) | 2.58 (-5.35, 11.18) |
| Nigeria | 75 to 79 | 2.6 (-2.6, 8.07) | 2.5 (-4.51, 10.03) | 2.57 (-5.11, 10.87) |
| Nigeria | 80 to 84 | 2.71 (-3.54, 9.37) | 2.69 (-6.45, 12.72) | 2.6 (-5.95, 11.92) |
| Nigeria | 85 to 89 | 2.81 (-6.51, 13.06) | 2.75 (-10.79, 18.36) | 2.73 (-9.78, 16.96) |
| Niue | 25 to 29 | 1.18 (-20.67, 29.06) | 1.39 (-20.51, 29.32) | 0.95 (-20.86, 28.76) |
| Niue | 30 to 34 | 1.3 (-16.3, 22.59) | 1.51 (-16.12, 22.84) | 1.08 (-16.47, 22.33) |
| Niue | 35 to 39 | 0.97 (-15.15, 20.16) | 1.05 (-15.09, 20.25) | 0.91 (-15.21, 20.08) |
| Niue | 40 to 44 | 0.65 (-14.95, 19.1) | 0.54 (-15.03, 18.98) | 0.78 (-14.83, 19.26) |
| Niue | 45 to 49 | 0.11 (-15.21, 18.21) | -0.15 (-15.43, 17.9) | 0.42 (-14.95, 18.56) |
| Niue | 50 to 54 | -0.21 (-15.37, 17.66) | -0.61 (-15.71, 17.19) | 0.21 (-15.01, 18.15) |
| Niue | 55 to 59 | -0.1 (-15.26, 17.78) | -0.32 (-15.45, 17.52) | 0.18 (-15.03, 18.11) |
| Niue | 60 to 64 | 0.34 (-14.9, 18.31) | 0.46 (-14.8, 18.45) | 0.38 (-14.86, 18.36) |
| Niue | 65 to 69 | 0.67 (-14.74, 18.86) | 1.06 (-14.41, 19.32) | 0.4 (-14.97, 18.54) |
| Niue | 70 to 74 | 0.32 (-15.22, 18.72) | 0.85 (-14.77, 19.34) | -0.05 (-15.54, 18.27) |
| Niue | 75 to 79 | -0.16 (-16.11, 18.81) | -0.11 (-16.06, 18.87) | -0.19 (-16.13, 18.77) |
| Niue | 80 to 84 | -0.12 (-17.46, 20.88) | -0.99 (-18.19, 19.82) | 0.22 (-17.19, 21.28) |
| Niue | 85 to 89 | 0.58 (-21.15, 28.29) | -0.67 (-22.13, 26.69) | 1.23 (-20.64, 29.12) |
| North Macedonia | 25 to 29 | 2.4 (-42.13, 81.2) | 0.22 (-52.37, 110.9) | -2.65 (-53.55, 104.01) |
| North Macedonia | 30 to 34 | 2.03 (-34.61, 59.2) | 0.25 (-42.66, 75.26) | -5.32 (-43.95, 59.95) |
| North Macedonia | 35 to 39 | 1.66 (-29.38, 46.35) | 0.95 (-35.87, 58.91) | -4.08 (-39.19, 51.32) |
| North Macedonia | 40 to 44 | 1.31 (-25.43, 37.66) | 1.28 (-31.61, 49.98) | -2.13 (-34.24, 45.66) |
| North Macedonia | 45 to 49 | 1.21 (-21.8, 30.99) | 1.4 (-27.77, 42.34) | -0.39 (-29.92, 41.57) |
| North Macedonia | 50 to 54 | 1.44 (-18.33, 26) | 1.54 (-24.17, 35.95) | 0.72 (-25.97, 37.03) |
| North Macedonia | 55 to 59 | 1.7 (-15.78, 22.82) | 1.71 (-21.3, 31.45) | 1.9 (-22.15, 33.38) |
| North Macedonia | 60 to 64 | 1.95 (-13.86, 20.66) | 1.69 (-19.25, 28.06) | 1.57 (-20.11, 29.14) |
| North Macedonia | 65 to 69 | 2.29 (-12.7, 19.87) | 2.09 (-18, 27.11) | 2.02 (-18.52, 27.72) |
| North Macedonia | 70 to 74 | 2.2 (-12.89, 19.9) | 2.18 (-18.16, 27.58) | 2.01 (-18.89, 28.29) |
| North Macedonia | 75 to 79 | 2.76 (-14.72, 23.82) | 2.71 (-20.62, 32.88) | 3.17 (-22.64, 37.57) |
| North Macedonia | 80 to 84 | 2.29 (-17.52, 26.87) | 2.16 (-22.83, 35.24) | 3.14 (-27.1, 45.9) |
| North Macedonia | 85 to 89 | 3.7 (-28.03, 49.44) | 3.36 (-34.58, 63.31) | 5.39 (-43.12, 95.26) |
| Northern Mariana Islands | 25 to 29 | 4.55 (-19.99, 36.61) | 3.33 (-19.78, 33.08) | 4.84 (-18.85, 35.43) |
| Northern Mariana Islands | 30 to 34 | 5.82 (-16.95, 34.83) | 4.42 (-14.68, 27.8) | 4.05 (-15, 27.37) |
| Northern Mariana Islands | 35 to 39 | 5.28 (-24.05, 45.93) | 1.94 (-16.46, 24.38) | 3.53 (-16.76, 28.76) |
| Northern Mariana Islands | 40 to 44 | 2.04 (-30.89, 50.66) | -0.16 (-21.34, 26.71) | 0 (-22.43, 28.92) |
| Northern Mariana Islands | 45 to 49 | 0.54 (-34.03, 53.21) | -9.55 (-31.16, 18.85) | -4.4 (-24.84, 21.59) |
| Northern Mariana Islands | 50 to 54 | -0.63 (-35.2, 52.36) | -10.41 (-35.13, 23.73) | -10.56 (-33.51, 20.3) |
| Northern Mariana Islands | 55 to 59 | -1.07 (-35.77, 52.38) | -15.14 (-38.04, 16.21) | -13.39 (-35.95, 17.11) |
| Northern Mariana Islands | 60 to 64 | -1.09 (-36.38, 53.79) | -14.44 (-36.99, 16.19) | -12.92 (-37.7, 21.72) |
| Northern Mariana Islands | 65 to 69 | -1.62 (-37.67, 55.28) | -12.91 (-34.47, 15.75) | -14.07 (-37.71, 18.54) |
| Northern Mariana Islands | 70 to 74 | -1.44 (-39.66, 61) | -7.03 (-28.77, 21.34) | -11.79 (-37.55, 24.62) |
| Northern Mariana Islands | 75 to 79 | -2.06 (-41.71, 64.57) | -7.95 (-29.63, 20.41) | -11.5 (-36.31, 22.99) |
| Northern Mariana Islands | 80 to 84 | -8.07 (-45.12, 53.98) | -4.36 (-23.7, 19.87) | -9.71 (-34.28, 24.04) |
| Northern Mariana Islands | 85 to 89 | -11.84 (-48.09, 49.7) | -5.45 (-26.94, 22.37) | -9.36 (-34.67, 25.75) |
| Norway | 25 to 29 | -7.33 (-52.19, 79.63) | -9.78 (-52.31, 70.68) | -6.37 (-48.1, 68.91) |
| Norway | 30 to 34 | -10.13 (-44.37, 45.2) | -11.51 (-44.75, 41.74) | -1.18 (-21.74, 24.78) |
| Norway | 35 to 39 | -10.89 (-40.92, 34.4) | -12.4 (-41.81, 31.86) | -0.94 (-18.87, 20.96) |
| Norway | 40 to 44 | -9.62 (-38, 31.75) | -11.83 (-39.84, 29.22) | -2.52 (-21.25, 20.66) |
| Norway | 45 to 49 | -5.52 (-33.56, 34.35) | -9.21 (-35.45, 27.69) | -4.7 (-26.8, 24.09) |
| Norway | 50 to 54 | -0.91 (-27.2, 34.87) | -7.07 (-30.6, 24.44) | -6.42 (-29.53, 24.27) |
| Norway | 55 to 59 | 1.48 (-21.86, 31.8) | -3.85 (-26.21, 25.29) | -6.59 (-29.38, 23.55) |
| Norway | 60 to 64 | 1.88 (-17.58, 25.92) | -1.31 (-21.72, 24.42) | -4.65 (-26.34, 23.43) |
| Norway | 65 to 69 | 2.09 (-13.5, 20.47) | 0.27 (-17.7, 22.16) | -1.66 (-21.31, 22.9) |
| Norway | 70 to 74 | 1.8 (-10.1, 15.28) | 1.05 (-13.67, 18.26) | 0.68 (-16.27, 21.08) |
| Norway | 75 to 79 | 2.01 (-7.84, 12.91) | 1.24 (-11.21, 15.45) | 2.15 (-12.06, 18.66) |
| Norway | 80 to 84 | 2.28 (-6.23, 11.56) | 1.43 (-9.79, 14.06) | 2.82 (-9.24, 16.47) |
| Norway | 85 to 89 | 2.73 (-7.44, 14) | 2.04 (-12.29, 18.71) | 3.34 (-10.32, 19.07) |
| Oman | 25 to 29 | -1.22 (-28.57, 36.6) | -4.29 (-33.31, 37.36) | -11.25 (-44.39, 41.65) |
| Oman | 30 to 34 | 0.63 (-24.49, 34.11) | -3.82 (-27, 26.72) | -12.03 (-37.67, 24.17) |
| Oman | 35 to 39 | 1.49 (-23.52, 34.68) | -1.86 (-27.34, 32.58) | -10.49 (-34.26, 21.88) |
| Oman | 40 to 44 | 2.17 (-22.74, 35.11) | -0.26 (-27.93, 38.03) | -7.93 (-31.88, 24.43) |
| Oman | 45 to 49 | 2.82 (-21.58, 34.83) | 1.39 (-27.45, 41.68) | -4.28 (-27.16, 25.79) |
| Oman | 50 to 54 | 3.79 (-19.33, 33.52) | 2.72 (-26.22, 43.01) | -2.93 (-26.11, 27.53) |
| Oman | 55 to 59 | 3.59 (-18.41, 31.54) | 2.95 (-25.51, 42.29) | -0.31 (-25.79, 33.92) |
| Oman | 60 to 64 | 3.96 (-17.75, 31.39) | 2.79 (-25.59, 41.99) | 1.52 (-25.79, 38.88) |
| Oman | 65 to 69 | 4.69 (-18.3, 34.15) | 3.68 (-26.46, 46.18) | 4.18 (-27.36, 49.42) |
| Oman | 70 to 74 | 5.22 (-20.17, 38.7) | 4.33 (-28.77, 52.8) | 5.2 (-29.41, 56.79) |
| Oman | 75 to 79 | 5.64 (-23.09, 45.11) | 4.92 (-31.59, 60.91) | 4.87 (-32.03, 61.79) |
| Oman | 80 to 84 | 6.6 (-32.49, 68.33) | 4.39 (-36.67, 72.06) | 4.58 (-37.03, 73.69) |
| Oman | 85 to 89 | 6.26 (-44.66, 104.04) | -3.12 (-36.96, 48.88) | 3.86 (-47.78, 106.57) |
| Pakistan | 25 to 29 | 3.08 (-8.64, 16.3) | 2.6 (-14.96, 23.78) | 3.43 (-11.67, 21.11) |
| Pakistan | 30 to 34 | 2.92 (-7.02, 13.92) | 2.44 (-12.48, 19.91) | 3.25 (-9.61, 17.93) |
| Pakistan | 35 to 39 | 2.44 (-6.32, 12.02) | 1.89 (-11.22, 16.95) | 2.77 (-8.63, 15.59) |
| Pakistan | 40 to 44 | 1.92 (-5.81, 10.29) | 1.33 (-10.24, 14.39) | 2.3 (-7.83, 13.54) |
| Pakistan | 45 to 49 | 1.44 (-5.32, 8.69) | 0.91 (-9.07, 11.99) | 1.84 (-7.15, 11.7) |
| Pakistan | 50 to 54 | 1.05 (-4.88, 7.34) | 0.58 (-7.95, 9.9) | 1.45 (-6.63, 10.22) |
| Pakistan | 55 to 59 | 0.69 (-4.69, 6.37) | 0.3 (-7.27, 8.49) | 1.06 (-6.44, 9.15) |
| Pakistan | 60 to 64 | 0.29 (-4.8, 5.66) | -0.08 (-7.19, 7.57) | 0.69 (-6.48, 8.4) |
| Pakistan | 65 to 69 | -0.1 (-5.09, 5.16) | -0.51 (-7.41, 6.91) | 0.31 (-6.78, 7.94) |
| Pakistan | 70 to 74 | -0.37 (-5.5, 5.03) | -0.81 (-7.81, 6.71) | 0.03 (-7.35, 7.99) |
| Pakistan | 75 to 79 | -0.45 (-6.18, 5.63) | -0.89 (-8.63, 7.52) | -0.05 (-8.41, 9.06) |
| Pakistan | 80 to 84 | -0.35 (-7.76, 7.66) | -0.69 (-10.33, 9.99) | -0.01 (-11.29, 12.71) |
| Pakistan | 85 to 89 | -0.14 (-11.13, 12.21) | -0.44 (-14.48, 15.91) | 0.22 (-16.55, 20.36) |
| Palau | 25 to 29 | 2.54 (-19.75, 31.03) | 1.5 (-20.42, 29.47) | 3.05 (-19.26, 31.52) |
| Palau | 30 to 34 | 1.67 (-16.09, 23.19) | 1.01 (-16.53, 22.24) | 2.04 (-15.69, 23.48) |
| Palau | 35 to 39 | 0.54 (-15.61, 19.77) | 0.17 (-15.83, 19.2) | 0.8 (-15.3, 19.97) |
| Palau | 40 to 44 | -0.77 (-16.41, 17.8) | -0.79 (-16.16, 17.4) | -0.25 (-15.79, 18.17) |
| Palau | 45 to 49 | -2.22 (-18.08, 16.71) | -1.97 (-16.97, 15.75) | -1.71 (-16.85, 16.19) |
| Palau | 50 to 54 | -4.02 (-20.44, 15.79) | -3.46 (-18.12, 13.83) | -3.34 (-18.19, 14.2) |
| Palau | 55 to 59 | -6.23 (-23.84, 15.46) | -4.71 (-19.17, 12.35) | -4.53 (-19.71, 13.5) |
| Palau | 60 to 64 | -6.14 (-24.7, 17) | -3.95 (-18.54, 13.25) | -4.14 (-20.05, 14.93) |
| Palau | 65 to 69 | -5.07 (-24.27, 19) | -2.62 (-17.53, 14.97) | -3.21 (-19.85, 16.87) |
| Palau | 70 to 74 | -3.62 (-22.88, 20.45) | -1.24 (-16.54, 16.86) | -1.9 (-18.23, 17.7) |
| Palau | 75 to 79 | -2 (-20.06, 20.14) | -0.24 (-16.17, 18.71) | -0.88 (-16.84, 18.15) |
| Palau | 80 to 84 | -1.42 (-18.8, 19.67) | -0.99 (-18.19, 19.81) | -0.83 (-18.1, 20.08) |
| Palau | 85 to 89 | -2.94 (-24.02, 23.99) | -3.47 (-24.32, 23.12) | -1.63 (-22.9, 25.5) |
| Palestine | 25 to 29 | -14.39 (-54.61, 61.46) | -10.15 (-50.54, 63.22) | -10.19 (-50.54, 63.07) |
| Palestine | 30 to 34 | -13.52 (-45.93, 38.31) | -4.38 (-24.27, 20.74) | -5.76 (-29.52, 26) |
| Palestine | 35 to 39 | -10.91 (-40.41, 33.18) | -4.39 (-23.51, 19.5) | -6.28 (-28.35, 22.59) |
| Palestine | 40 to 44 | -7.31 (-33.2, 28.62) | -6.88 (-29.2, 22.48) | -8.04 (-30.92, 22.44) |
| Palestine | 45 to 49 | -6.72 (-33.87, 31.58) | -8.73 (-34.2, 26.59) | -9.04 (-34.54, 26.38) |
| Palestine | 50 to 54 | -5.28 (-32.57, 33.05) | -10.47 (-36.07, 25.37) | -7.29 (-35.23, 32.71) |
| Palestine | 55 to 59 | -3.23 (-30.17, 34.09) | -9.22 (-38.06, 33.04) | -4.9 (-34.55, 38.18) |
| Palestine | 60 to 64 | -1.43 (-28.14, 35.2) | -6.42 (-38.17, 41.65) | -2.84 (-33.17, 41.27) |
| Palestine | 65 to 69 | -0.43 (-26.5, 34.89) | -2.69 (-38.28, 53.45) | -1.05 (-31.78, 43.53) |
| Palestine | 70 to 74 | -1 (-27.58, 35.34) | -0.58 (-38.64, 61.07) | 0.14 (-34.29, 52.62) |
| Palestine | 75 to 79 | -0.82 (-29.72, 39.96) | 0.32 (-39.92, 67.54) | -0.6 (-38.09, 59.6) |
| Palestine | 80 to 84 | -0.25 (-34.74, 52.47) | -1.67 (-45.31, 76.79) | -0.74 (-42.21, 70.49) |
| Palestine | 85 to 89 | 0.89 (-47.81, 95.04) | -2.11 (-53.99, 108.29) | -1.47 (-51.88, 101.75) |
| Panama | 25 to 29 | 4.16 (-26.76, 48.15) | 3.21 (-35.09, 64.11) | -0.61 (-40.22, 65.24) |
| Panama | 30 to 34 | 4.7 (-22.78, 41.96) | 3.95 (-30.09, 54.56) | -2.79 (-32.05, 39.08) |
| Panama | 35 to 39 | 5.01 (-20.33, 38.41) | 4.32 (-27.1, 49.29) | -3.57 (-29.24, 31.4) |
| Panama | 40 to 44 | 5.19 (-17.74, 34.51) | 4.47 (-23.73, 43.1) | -1.41 (-26.84, 32.85) |
| Panama | 45 to 49 | 5.08 (-14.81, 29.6) | 4.43 (-19.85, 36.07) | 0.24 (-24.64, 33.34) |
| Panama | 50 to 54 | 4.7 (-12.67, 25.54) | 4.11 (-17.61, 31.55) | 2.05 (-21.69, 32.99) |
| Panama | 55 to 59 | 4.44 (-11.17, 22.79) | 4.01 (-15.72, 28.35) | 3.43 (-19.57, 33) |
| Panama | 60 to 64 | 3.88 (-10.66, 20.79) | 3.43 (-15.16, 26.09) | 3.15 (-18.28, 30.2) |
| Panama | 65 to 69 | 3.44 (-10.82, 19.99) | 3.05 (-15.1, 25.07) | 2.23 (-18.6, 28.39) |
| Panama | 70 to 74 | 3.18 (-11.07, 19.72) | 2.81 (-15.53, 25.14) | 2.19 (-18.91, 28.78) |
| Panama | 75 to 79 | 3.06 (-12, 20.7) | 2.65 (-16.84, 26.7) | 2.31 (-19.6, 30.2) |
| Panama | 80 to 84 | 3.01 (-15, 24.84) | 3.01 (-20.74, 33.88) | 3.11 (-24.02, 39.93) |
| Panama | 85 to 89 | 3.37 (-24.54, 41.6) | 3.95 (-33.15, 61.65) | 4.2 (-33.74, 63.86) |
| Papua New Guinea | 25 to 29 | -1.79 (-51.37, 98.33) | -10.14 (-52.02, 68.31) | -9.77 (-50.52, 64.55) |
| Papua New Guinea | 30 to 34 | -0.04 (-35.58, 55.09) | -3.45 (-37.52, 49.22) | -5.03 (-30.39, 29.58) |
| Papua New Guinea | 35 to 39 | -0.28 (-29.3, 40.66) | -0.95 (-29.68, 39.52) | -4.3 (-32.85, 36.38) |
| Papua New Guinea | 40 to 44 | -0.27 (-25.42, 33.37) | -0.74 (-29.78, 40.3) | -3.85 (-34.27, 40.64) |
| Papua New Guinea | 45 to 49 | -0.56 (-23.42, 29.11) | -0.76 (-28.9, 38.53) | -2.61 (-32.91, 41.37) |
| Papua New Guinea | 50 to 54 | -0.67 (-21.73, 26.05) | -0.23 (-27.68, 37.64) | -2.16 (-31.37, 39.47) |
| Papua New Guinea | 55 to 59 | -0.61 (-21.19, 25.36) | -0.1 (-27.26, 37.19) | -0.59 (-29.61, 40.4) |
| Papua New Guinea | 60 to 64 | -0.47 (-21.46, 26.14) | 0.09 (-27.3, 37.82) | -1.64 (-31.39, 40.99) |
| Papua New Guinea | 65 to 69 | -0.57 (-22.42, 27.43) | 0.34 (-28.11, 40.06) | -1.26 (-32.1, 43.59) |
| Papua New Guinea | 70 to 74 | -0.39 (-24, 30.55) | 0.25 (-29.95, 43.47) | -0.62 (-33.71, 48.99) |
| Papua New Guinea | 75 to 79 | -0.16 (-28.99, 40.36) | 0.17 (-36.03, 56.86) | -4.11 (-38.2, 48.77) |
| Papua New Guinea | 80 to 84 | -0.22 (-38.99, 63.18) | -6.08 (-40.89, 49.24) | -8.2 (-43.9, 50.21) |
| Papua New Guinea | 85 to 89 | -1.68 (-50.96, 97.12) | -11.27 (-46.74, 47.82) | -10.65 (-46.6, 49.5) |
| Paraguay | 25 to 29 | 2.08 (-21.51, 32.74) | 1.6 (-31.24, 50.11) | 2.46 (-28.25, 46.32) |
| Paraguay | 30 to 34 | 1.87 (-18.28, 26.99) | 1.75 (-26.09, 40.08) | 1.96 (-24.87, 38.37) |
| Paraguay | 35 to 39 | 1.94 (-15.8, 23.41) | 1.78 (-22.43, 33.54) | 2.04 (-22.18, 33.8) |
| Paraguay | 40 to 44 | 1.62 (-14.22, 20.38) | 1.93 (-19.34, 28.81) | 1.68 (-20.63, 30.26) |
| Paraguay | 45 to 49 | 1.34 (-12.62, 17.52) | 1.86 (-16.59, 24.4) | 0.9 (-19.36, 26.25) |
| Paraguay | 50 to 54 | 1.09 (-11.07, 14.91) | 1.57 (-14.3, 20.38) | 0.63 (-17.43, 22.64) |
| Paraguay | 55 to 59 | 0.87 (-10.07, 13.13) | 1.2 (-13.06, 17.81) | 0.55 (-15.68, 19.91) |
| Paraguay | 60 to 64 | 0.47 (-9.53, 11.57) | 0.69 (-12.32, 15.62) | 0.26 (-14.65, 17.77) |
| Paraguay | 65 to 69 | 0.12 (-9.49, 10.74) | 0.16 (-12.32, 14.42) | 0.14 (-14.3, 17.01) |
| Paraguay | 70 to 74 | 0.22 (-9.47, 10.96) | 0.14 (-12.35, 14.4) | 0.52 (-14.18, 17.74) |
| Paraguay | 75 to 79 | 0.46 (-9.91, 12.03) | 0.49 (-13.04, 16.11) | 0.61 (-14.8, 18.81) |
| Paraguay | 80 to 84 | 0.76 (-11.45, 14.64) | 0.8 (-15.28, 19.93) | 0.67 (-16.76, 21.75) |
| Paraguay | 85 to 89 | 1.25 (-16.44, 22.69) | 1.39 (-22.55, 32.72) | 1.02 (-23.16, 32.81) |
| Peru | 25 to 29 | 5.44 (-9.35, 22.63) | 5.19 (-14.62, 29.59) | 5.91 (-14.82, 31.68) |
| Peru | 30 to 34 | 4.92 (-7.07, 18.45) | 4.61 (-11.49, 23.65) | 5.19 (-11.93, 25.63) |
| Peru | 35 to 39 | 4.58 (-5.14, 15.29) | 4.39 (-8.68, 19.33) | 4.8 (-9.16, 20.91) |
| Peru | 40 to 44 | 4.45 (-3.42, 12.96) | 4.32 (-6.3, 16.15) | 4.59 (-6.76, 17.32) |
| Peru | 45 to 49 | 4.38 (-1.93, 11.09) | 4.32 (-4.33, 13.75) | 4.49 (-4.51, 14.33) |
| Peru | 50 to 54 | 4.31 (-0.87, 9.76) | 4.28 (-2.93, 12.02) | 4.37 (-2.95, 12.24) |
| Peru | 55 to 59 | 4.04 (-0.41, 8.69) | 3.95 (-2.33, 10.64) | 4.14 (-2.08, 10.76) |
| Peru | 60 to 64 | 3.98 (0.02, 8.1) | 3.7 (-1.99, 9.72) | 4.24 (-1.22, 9.99) |
| Peru | 65 to 69 | 3.75 (-0.03, 7.66) | 3.39 (-2.05, 9.14) | 4.06 (-1.11, 9.51) |
| Peru | 70 to 74 | 3.31 (-0.4, 7.17) | 2.82 (-2.54, 8.48) | 3.74 (-1.35, 9.09) |
| Peru | 75 to 79 | 2.75 (-1.16, 6.8) | 2.25 (-3.42, 8.26) | 3.15 (-2.16, 8.74) |
| Peru | 80 to 84 | 2.11 (-2.38, 6.8) | 1.73 (-5.04, 8.98) | 2.38 (-3.53, 8.65) |
| Peru | 85 to 89 | 1.95 (-4.46, 8.79) | 1.89 (-8.35, 13.28) | 2.03 (-6.03, 10.79) |
| Philippines | 25 to 29 | 4.29 (-8.31, 18.62) | 4.16 (-12.82, 24.44) | 4.57 (-13.25, 26.06) |
| Philippines | 30 to 34 | 4.44 (-3.38, 12.9) | 4.24 (-6.35, 16.02) | 4.74 (-6.44, 17.27) |
| Philippines | 35 to 39 | 4.54 (-1.1, 10.51) | 4.29 (-3.36, 12.55) | 4.8 (-3.37, 13.66) |
| Philippines | 40 to 44 | 4.53 (0.04, 9.22) | 4.36 (-1.7, 10.79) | 4.74 (-1.83, 11.74) |
| Philippines | 45 to 49 | 4.49 (0.71, 8.42) | 4.32 (-0.78, 9.67) | 4.68 (-0.9, 10.57) |
| Philippines | 50 to 54 | 4.39 (1.02, 7.89) | 4.26 (-0.29, 9.01) | 4.54 (-0.44, 9.77) |
| Philippines | 55 to 59 | 4.38 (1.22, 7.64) | 4.27 (-0.01, 8.73) | 4.51 (-0.14, 9.37) |
| Philippines | 60 to 64 | 4.33 (1.25, 7.51) | 4.33 (0.09, 8.76) | 4.37 (-0.09, 9.03) |
| Philippines | 65 to 69 | 4.35 (1.16, 7.65) | 4.37 (-0.09, 9.03) | 4.4 (-0.15, 9.17) |
| Philippines | 70 to 74 | 4.06 (0.61, 7.62) | 4.16 (-0.77, 9.33) | 4.07 (-0.72, 9.1) |
| Philippines | 75 to 79 | 3.45 (-0.42, 7.47) | 3.77 (-1.99, 9.86) | 3.28 (-1.92, 8.76) |
| Philippines | 80 to 84 | 2.59 (-2.06, 7.46) | 2.97 (-4.18, 10.66) | 2.34 (-3.72, 8.78) |
| Philippines | 85 to 89 | 2.23 (-4.39, 9.32) | 2.7 (-7.88, 14.51) | 1.79 (-6.57, 10.9) |
| Poland | 25 to 29 | 0.03 (-14.49, 17.01) | 0.13 (-19.47, 24.49) | -0.1 (-20.39, 25.37) |
| Poland | 30 to 34 | -0.49 (-11.24, 11.57) | -0.16 (-14.2, 16.18) | -0.96 (-16.9, 18.04) |
| Poland | 35 to 39 | -1.09 (-9.74, 8.39) | -0.68 (-11.72, 11.73) | -1.73 (-15.11, 13.77) |
| Poland | 40 to 44 | -1.32 (-8.6, 6.54) | -0.92 (-10.07, 9.15) | -2.03 (-13.64, 11.14) |
| Poland | 45 to 49 | -1.35 (-7.62, 5.35) | -1.03 (-8.97, 7.61) | -1.92 (-11.81, 9.08) |
| Poland | 50 to 54 | -1.4 (-6.66, 4.15) | -1.1 (-7.87, 6.18) | -1.96 (-10.11, 6.94) |
| Poland | 55 to 59 | -1.58 (-5.82, 2.85) | -1.27 (-6.87, 4.66) | -2.05 (-8.46, 4.81) |
| Poland | 60 to 64 | -1.8 (-5.24, 1.78) | -1.46 (-6.17, 3.49) | -2.24 (-7.27, 3.07) |
| Poland | 65 to 69 | -1.87 (-4.88, 1.23) | -1.62 (-5.9, 2.86) | -2.17 (-6.38, 2.24) |
| Poland | 70 to 74 | -1.81 (-4.65, 1.11) | -1.9 (-6.08, 2.47) | -1.84 (-5.7, 2.17) |
| Poland | 75 to 79 | -1.57 (-4.52, 1.48) | -1.98 (-6.55, 2.82) | -1.36 (-5.22, 2.65) |
| Poland | 80 to 84 | -1.49 (-4.52, 1.64) | -2.32 (-7.18, 2.79) | -1.04 (-4.9, 2.96) |
| Poland | 85 to 89 | -1.4 (-5.45, 2.83) | -2.5 (-9.2, 4.71) | -0.85 (-5.88, 4.46) |
| Portugal | 25 to 29 | -0.17 (-33.64, 50.17) | -0.53 (-40.16, 65.36) | 2.01 (-40.32, 74.36) |
| Portugal | 30 to 34 | -0.4 (-26.87, 35.66) | -1.61 (-35.23, 49.45) | 1.23 (-35.9, 59.88) |
| Portugal | 35 to 39 | -0.37 (-22.52, 28.12) | -1.35 (-29.28, 37.59) | 0.93 (-31.6, 48.93) |
| Portugal | 40 to 44 | -0.11 (-18.63, 22.64) | -0.99 (-24.71, 30.22) | 1 (-26.55, 38.9) |
| Portugal | 45 to 49 | 0.36 (-15.24, 18.84) | -0.25 (-20.48, 25.12) | 1.14 (-22.22, 31.53) |
| Portugal | 50 to 54 | 0.22 (-12.29, 14.51) | 0.26 (-15.92, 19.55) | 0.45 (-18.01, 23.07) |
| Portugal | 55 to 59 | -0.27 (-10.22, 10.79) | -0.15 (-13.24, 14.92) | -0.38 (-14.95, 16.69) |
| Portugal | 60 to 64 | -0.83 (-8.64, 7.66) | -0.78 (-11.3, 10.99) | -0.88 (-12.2, 11.9) |
| Portugal | 65 to 69 | -1.27 (-7.54, 5.42) | -1.35 (-9.96, 8.08) | -1.14 (-10.03, 8.63) |
| Portugal | 70 to 74 | -1.27 (-6.33, 4.06) | -1.59 (-8.69, 6.07) | -0.99 (-8.05, 6.62) |
| Portugal | 75 to 79 | -0.93 (-5.18, 3.51) | -1.56 (-7.74, 5.03) | -0.42 (-6.21, 5.74) |
| Portugal | 80 to 84 | -0.55 (-4.34, 3.39) | -1.41 (-7.19, 4.74) | 0.02 (-4.97, 5.28) |
| Portugal | 85 to 89 | -0.41 (-5.1, 4.52) | -1.58 (-9.05, 6.51) | 0.19 (-5.74, 6.49) |
| Puerto Rico | 25 to 29 | 2.6 (-13.77, 22.08) | 3.22 (-16.6, 27.74) | 1.68 (-25.03, 37.91) |
| Puerto Rico | 30 to 34 | 2.72 (-10.21, 17.52) | 3.25 (-12.66, 22.07) | 1.83 (-19, 28) |
| Puerto Rico | 35 to 39 | 2.67 (-8.7, 15.46) | 3.16 (-10.44, 18.83) | 1.35 (-18.92, 26.69) |
| Puerto Rico | 40 to 44 | 2.45 (-7.85, 13.9) | 2.75 (-9.09, 16.13) | 1.29 (-19.12, 26.83) |
| Puerto Rico | 45 to 49 | 2.26 (-7.02, 12.47) | 2.41 (-7.91, 13.89) | 1.43 (-18.74, 26.62) |
| Puerto Rico | 50 to 54 | 2.11 (-6.23, 11.19) | 2.19 (-6.9, 12.17) | 1.42 (-18.15, 25.67) |
| Puerto Rico | 55 to 59 | 2.09 (-5.55, 10.34) | 2.18 (-6.06, 11.16) | 1.43 (-17.3, 24.4) |
| Puerto Rico | 60 to 64 | 2.36 (-4.7, 9.95) | 2.47 (-5.13, 10.69) | 1.65 (-15.96, 22.96) |
| Puerto Rico | 65 to 69 | 2.67 (-4.03, 9.85) | 2.74 (-4.5, 10.54) | 2.19 (-14.32, 21.88) |
| Puerto Rico | 70 to 74 | 3.12 (-3.41, 10.11) | 3.16 (-3.99, 10.84) | 2.79 (-12.6, 20.89) |
| Puerto Rico | 75 to 79 | 3.45 (-3.35, 10.72) | 3.64 (-3.96, 11.83) | 3.01 (-11.8, 20.31) |
| Puerto Rico | 80 to 84 | 3.24 (-4.49, 11.6) | 3.62 (-5.19, 13.25) | 2.58 (-12.87, 20.78) |
| Puerto Rico | 85 to 89 | 2.72 (-8.03, 14.72) | 3.21 (-9.09, 17.18) | 2.01 (-18.56, 27.77) |
| Qatar | 25 to 29 | 3.24 (-25.63, 43.31) | 2.51 (-29.96, 50.03) | -11.29 (-40.15, 31.49) |
| Qatar | 30 to 34 | 2.63 (-21.58, 34.33) | 2 (-25.7, 40.03) | -8.54 (-34.62, 27.96) |
| Qatar | 35 to 39 | 2.03 (-19.79, 29.8) | 1.34 (-23.51, 34.27) | -5.17 (-33.18, 34.59) |
| Qatar | 40 to 44 | 1.28 (-19.13, 26.84) | 0.61 (-22.56, 30.71) | -2.51 (-31.89, 39.55) |
| Qatar | 45 to 49 | 1.03 (-18.47, 25.18) | 0.29 (-21.87, 28.73) | 0.25 (-29.44, 42.44) |
| Qatar | 50 to 54 | 0.78 (-18.17, 24.13) | 0.35 (-22.19, 29.41) | 0.67 (-27.95, 40.64) |
| Qatar | 55 to 59 | 1.15 (-17.65, 24.23) | 0.61 (-22.98, 31.43) | -0.29 (-26.94, 36.08) |
| Qatar | 60 to 64 | 1.91 (-17.02, 25.17) | 1.48 (-23.28, 34.23) | 0.58 (-25.01, 34.9) |
| Qatar | 65 to 69 | 2.4 (-17.56, 27.19) | 2.18 (-25.11, 39.41) | 1.62 (-24.39, 36.56) |
| Qatar | 70 to 74 | 2.89 (-18.92, 30.56) | 3.36 (-28.41, 49.25) | 1.95 (-26.12, 40.68) |
| Qatar | 75 to 79 | 3.78 (-24.08, 41.86) | 2.72 (-32.35, 55.95) | 3.3 (-30.41, 53.33) |
| Qatar | 80 to 84 | 4.09 (-33.59, 63.16) | -5.68 (-31.54, 29.95) | -3.94 (-27.98, 28.13) |
| Qatar | 85 to 89 | 2.57 (-45.9, 94.48) | -12 (-39.75, 28.54) | -9.67 (-33.3, 22.33) |
| Republic of Korea | 25 to 29 | 1.49 (-37.73, 65.43) | 1.41 (-48.29, 98.85) | -0.29 (-51.52, 105.09) |
| Republic of Korea | 30 to 34 | 1.59 (-22.81, 33.7) | 2.04 (-28.72, 46.07) | -0.67 (-39.24, 62.4) |
| Republic of Korea | 35 to 39 | 1.25 (-15.25, 20.97) | 2.12 (-18.07, 27.27) | -0.53 (-26.74, 35.07) |
| Republic of Korea | 40 to 44 | 0.67 (-11.82, 14.93) | 1.44 (-13.95, 19.58) | -0.66 (-20.42, 24.01) |
| Republic of Korea | 45 to 49 | 0.56 (-8.62, 10.67) | 1.31 (-10.24, 14.35) | -0.76 (-15.34, 16.32) |
| Republic of Korea | 50 to 54 | 0.09 (-7.06, 7.8) | 0.89 (-8.23, 10.91) | -1.3 (-12.57, 11.43) |
| Republic of Korea | 55 to 59 | -0.05 (-5.78, 6.02) | 0.73 (-6.7, 8.75) | -1.53 (-10.49, 8.32) |
| Republic of Korea | 60 to 64 | -0.07 (-5.14, 5.27) | 0.81 (-6.01, 8.13) | -1.43 (-9.03, 6.82) |
| Republic of Korea | 65 to 69 | 0 (-4.84, 5.08) | 0.79 (-6.03, 8.11) | -1.08 (-8.01, 6.38) |
| Republic of Korea | 70 to 74 | -0.04 (-4.53, 4.67) | 0.54 (-6.11, 7.66) | -0.75 (-6.89, 5.79) |
| Republic of Korea | 75 to 79 | 0.07 (-4.23, 4.57) | 0.39 (-6.39, 7.67) | -0.25 (-5.8, 5.62) |
| Republic of Korea | 80 to 84 | 0.01 (-4.73, 4.99) | 0.2 (-7.66, 8.75) | -0.2 (-6.07, 6.05) |
| Republic of Korea | 85 to 89 | 0.54 (-6.48, 8.08) | 0.58 (-11.06, 13.74) | 0.34 (-8.27, 9.77) |
| Republic of Moldova | 25 to 29 | 0.43 (-22.18, 29.61) | 0.11 (-21.57, 27.78) | 0.46 (-21.48, 28.54) |
| Republic of Moldova | 30 to 34 | 0.69 (-17.33, 22.64) | -0.02 (-17.47, 21.13) | 0.52 (-17.07, 21.84) |
| Republic of Moldova | 35 to 39 | 0.29 (-17.07, 21.28) | 0.67 (-15.63, 20.13) | 1.17 (-15.2, 20.7) |
| Republic of Moldova | 40 to 44 | -1.23 (-20.04, 22) | 0.95 (-15.07, 19.98) | 1.41 (-14.88, 20.81) |
| Republic of Moldova | 45 to 49 | -4.69 (-26, 22.75) | -1.33 (-17.45, 17.95) | -0.75 (-17.26, 19.06) |
| Republic of Moldova | 50 to 54 | -6.36 (-29.65, 24.63) | -2.66 (-20.09, 18.57) | -2.96 (-20.72, 18.78) |
| Republic of Moldova | 55 to 59 | -3.35 (-32.68, 38.77) | -3.49 (-22.48, 20.14) | -5.53 (-25.68, 20.09) |
| Republic of Moldova | 60 to 64 | 0.5 (-33.31, 51.44) | -3.14 (-22.16, 20.53) | -8 (-30.92, 22.53) |
| Republic of Moldova | 65 to 69 | 3.58 (-34.42, 63.61) | -1.69 (-19.1, 19.46) | -8.47 (-34.13, 27.19) |
| Republic of Moldova | 70 to 74 | 0.06 (-37.22, 59.46) | -0.41 (-16, 18.08) | -7.35 (-33.84, 29.75) |
| Republic of Moldova | 75 to 79 | -5.94 (-40.73, 49.28) | -2.32 (-17.96, 16.29) | -7.21 (-31.8, 26.24) |
| Republic of Moldova | 80 to 84 | -9.64 (-43.59, 44.73) | -2.82 (-19.71, 17.62) | -5.36 (-26.74, 22.27) |
| Republic of Moldova | 85 to 89 | -10.03 (-42.58, 40.99) | -3.46 (-24.34, 23.2) | -4.02 (-25.26, 23.26) |
| Romania | 25 to 29 | 3.82 (-10.57, 20.51) | 2.94 (-17.32, 28.17) | 4.56 (-14.87, 28.43) |
| Romania | 30 to 34 | 2.97 (-7.95, 15.18) | 2.84 (-11.55, 19.58) | 3.13 (-12.69, 21.82) |
| Romania | 35 to 39 | 2.86 (-6.12, 12.7) | 2.96 (-8.81, 16.25) | 2.74 (-10.63, 18.11) |
| Romania | 40 to 44 | 2.65 (-4.88, 10.78) | 2.79 (-7.11, 13.74) | 2.5 (-8.73, 15.12) |
| Romania | 45 to 49 | 3.06 (-3.58, 10.15) | 3.12 (-5.74, 12.82) | 2.98 (-6.77, 13.74) |
| Romania | 50 to 54 | 3.55 (-2.29, 9.74) | 3.7 (-4.24, 12.29) | 3.39 (-5.05, 12.58) |
| Romania | 55 to 59 | 4.24 (-0.91, 9.64) | 4.57 (-2.52, 12.18) | 3.9 (-3.44, 11.8) |
| Romania | 60 to 64 | 4.76 (0.32, 9.38) | 5.06 (-1.18, 11.69) | 4.47 (-1.73, 11.07) |
| Romania | 65 to 69 | 5.36 (1.14, 9.75) | 5.5 (-0.53, 11.9) | 5.24 (-0.58, 11.4) |
| Romania | 70 to 74 | 5.75 (1.36, 10.34) | 5.65 (-0.77, 12.49) | 5.82 (-0.12, 12.11) |
| Romania | 75 to 79 | 5.89 (0.46, 11.62) | 5.53 (-2.5, 14.22) | 6.15 (-1.08, 13.9) |
| Romania | 80 to 84 | 5.65 (-0.5, 12.19) | 5.53 (-3.69, 15.63) | 5.81 (-2.33, 14.63) |
| Romania | 85 to 89 | 4.34 (-3.98, 13.37) | 5.01 (-8.01, 19.88) | 3.95 (-6.59, 15.69) |
| Russian Federation | 25 to 29 | -3.31 (-14.59, 9.47) | -3.29 (-18.04, 14.13) | -3.08 (-19.3, 16.42) |
| Russian Federation | 30 to 34 | -2.43 (-10.51, 6.37) | -2.33 (-13.04, 9.71) | -2.57 (-14.39, 10.89) |
| Russian Federation | 35 to 39 | -1.64 (-8.74, 6.01) | -1.29 (-10.96, 9.43) | -1.99 (-12.17, 9.37) |
| Russian Federation | 40 to 44 | -1.17 (-7.53, 5.62) | -0.63 (-9.64, 9.27) | -1.68 (-10.55, 8.07) |
| Russian Federation | 45 to 49 | -0.62 (-6.27, 5.38) | 0.09 (-8.31, 9.25) | -1.16 (-8.76, 7.07) |
| Russian Federation | 50 to 54 | -0.17 (-4.95, 4.85) | 0.57 (-6.97, 8.72) | -0.62 (-6.81, 5.99) |
| Russian Federation | 55 to 59 | 1.05 (-2.88, 5.13) | 1.64 (-4.98, 8.73) | 0.75 (-4.11, 5.85) |
| Russian Federation | 60 to 64 | 2.36 (-1.13, 5.97) | 2.82 (-3.47, 9.52) | 2.16 (-2.02, 6.52) |
| Russian Federation | 65 to 69 | 3.74 (0.36, 7.23) | 3.92 (-2.48, 10.75) | 3.68 (-0.27, 7.78) |
| Russian Federation | 70 to 74 | 4.95 (0.97, 9.09) | 4.57 (-3.44, 13.23) | 5.1 (0.54, 9.87) |
| Russian Federation | 75 to 79 | 6.2 (1.34, 11.3) | 4.71 (-4.7, 15.04) | 6.68 (1.07, 12.59) |
| Russian Federation | 80 to 84 | 6.8 (0.62, 13.36) | 3.89 (-6.98, 16.02) | 7.77 (0.37, 15.72) |
| Russian Federation | 85 to 89 | 7.44 (-2.26, 18.1) | 2.68 (-12.55, 20.57) | 9.12 (-3.11, 22.89) |
| Rwanda | 25 to 29 | -13.39 (-53.95, 62.9) | -4.17 (-25.7, 23.61) | -4.18 (-26.88, 25.59) |
| Rwanda | 30 to 34 | -8.91 (-42.63, 44.63) | -3.32 (-23.27, 21.82) | -4.51 (-27.43, 25.65) |
| Rwanda | 35 to 39 | -2.18 (-29.39, 35.51) | -1.88 (-27.88, 33.5) | -2.78 (-28.08, 31.41) |
| Rwanda | 40 to 44 | -1.14 (-27.97, 35.69) | -0.27 (-29.79, 41.66) | -1.35 (-29.86, 38.74) |
| Rwanda | 45 to 49 | -0.23 (-24.76, 32.28) | -0.09 (-30.24, 43.09) | -0.3 (-29, 40.02) |
| Rwanda | 50 to 54 | -0.54 (-21.79, 26.48) | 0.02 (-28.08, 39.1) | -0.19 (-27.52, 37.44) |
| Rwanda | 55 to 59 | -0.86 (-19.33, 21.84) | -1.22 (-26.38, 32.53) | 0.06 (-24.46, 32.53) |
| Rwanda | 60 to 64 | -0.59 (-17.51, 19.79) | -1.14 (-24.39, 29.26) | 0.02 (-22.93, 29.79) |
| Rwanda | 65 to 69 | -0.08 (-16.63, 19.76) | -0.66 (-23.31, 28.69) | 0.55 (-22.13, 29.83) |
| Rwanda | 70 to 74 | 0.08 (-16.66, 20.18) | -0.4 (-22.69, 28.31) | 1.09 (-22.09, 31.18) |
| Rwanda | 75 to 79 | 0.82 (-19.06, 25.57) | 0.38 (-27.48, 38.92) | 1.23 (-24.94, 36.51) |
| Rwanda | 80 to 84 | 1.45 (-24.97, 37.17) | 0.02 (-34.09, 51.79) | 1.18 (-31.59, 49.66) |
| Rwanda | 85 to 89 | 1.63 (-35.99, 61.34) | -0.25 (-49.11, 95.51) | 1.79 (-45.99, 91.86) |
| Saint Kitts and Nevis | 25 to 29 | 0.07 (-22.11, 28.59) | -0.55 (-22.28, 27.25) | -1.09 (-22.45, 26.17) |
| Saint Kitts and Nevis | 30 to 34 | -0.74 (-18.34, 20.66) | -1.18 (-18.44, 19.72) | -1.24 (-18.39, 19.51) |
| Saint Kitts and Nevis | 35 to 39 | -1.78 (-18.54, 18.44) | -1.58 (-17.66, 17.65) | -1.39 (-17.14, 17.34) |
| Saint Kitts and Nevis | 40 to 44 | -4.36 (-21.46, 16.46) | -3.24 (-19.02, 15.61) | -2.47 (-17.58, 15.41) |
| Saint Kitts and Nevis | 45 to 49 | -7.74 (-26.65, 16.05) | -5.51 (-21.92, 14.34) | -4.11 (-18.79, 13.22) |
| Saint Kitts and Nevis | 50 to 54 | -10.51 (-30.51, 15.26) | -7.7 (-25.33, 14.09) | -5.46 (-19.82, 11.47) |
| Saint Kitts and Nevis | 55 to 59 | -11.66 (-32.8, 16.14) | -8.06 (-26.2, 14.55) | -5.39 (-19.75, 11.54) |
| Saint Kitts and Nevis | 60 to 64 | -11.35 (-32.89, 17.09) | -6.93 (-25.08, 15.61) | -4.3 (-18.84, 12.83) |
| Saint Kitts and Nevis | 65 to 69 | -8.2 (-29.86, 20.16) | -3.62 (-20.69, 17.11) | -2 (-17, 15.71) |
| Saint Kitts and Nevis | 70 to 74 | -3.96 (-26.52, 25.51) | -0.53 (-17.23, 19.54) | -0.09 (-15.57, 18.22) |
| Saint Kitts and Nevis | 75 to 79 | -0.49 (-24.94, 31.93) | 1.3 (-15.03, 20.78) | 0.84 (-15.26, 20) |
| Saint Kitts and Nevis | 80 to 84 | -1.35 (-23.66, 27.48) | 0.64 (-16.91, 21.9) | 0.42 (-17.02, 21.53) |
| Saint Kitts and Nevis | 85 to 89 | -1.77 (-24.45, 27.72) | -1.18 (-22.6, 26.17) | -0.49 (-21.99, 26.92) |
| Saint Lucia | 25 to 29 | -8.25 (-49.64, 67.17) | -0.6 (-23.26, 28.75) | 0.64 (-21.7, 29.34) |
| Saint Lucia | 30 to 34 | -3.76 (-29.44, 31.25) | -1.38 (-19.48, 20.79) | -0.42 (-18.04, 20.99) |
| Saint Lucia | 35 to 39 | -5.41 (-34.22, 36.01) | -3.05 (-22.56, 21.37) | -2.15 (-18.81, 17.94) |
| Saint Lucia | 40 to 44 | -7.17 (-34.93, 32.44) | -6.57 (-29.83, 24.4) | -4.82 (-22.54, 16.95) |
| Saint Lucia | 45 to 49 | -6.24 (-36.69, 38.85) | -9.28 (-35.86, 28.32) | -6.77 (-26.39, 18.08) |
| Saint Lucia | 50 to 54 | -4.67 (-37.1, 44.49) | -12.63 (-39.12, 25.38) | -7.55 (-27.52, 17.91) |
| Saint Lucia | 55 to 59 | -2.29 (-36.87, 51.21) | -15.02 (-39.76, 19.87) | -6.99 (-26.13, 17.1) |
| Saint Lucia | 60 to 64 | -0.79 (-36.99, 56.2) | -15.46 (-40.28, 19.67) | -5.65 (-23.79, 16.82) |
| Saint Lucia | 65 to 69 | 0.77 (-37.29, 61.95) | -13.3 (-40.32, 25.96) | -3.91 (-21.71, 17.93) |
| Saint Lucia | 70 to 74 | -0.68 (-38.83, 61.26) | -9.68 (-42.05, 40.76) | -5.3 (-25.26, 19.98) |
| Saint Lucia | 75 to 79 | -0.29 (-39.44, 64.18) | -9.65 (-42.86, 42.86) | -8.63 (-31.24, 21.4) |
| Saint Lucia | 80 to 84 | -0.66 (-42.62, 71.97) | -11.55 (-42.72, 36.57) | -12.55 (-37.59, 22.53) |
| Saint Lucia | 85 to 89 | -2.53 (-52.72, 100.91) | -13.9 (-45.4, 35.78) | -15.37 (-44.42, 28.85) |
| Saint Vincent and the Grenadines | 25 to 29 | -7.54 (-49.04, 67.76) | 1.75 (-21.24, 31.45) | 1.81 (-20.81, 30.89) |
| Saint Vincent and the Grenadines | 30 to 34 | -2.02 (-23.54, 25.56) | 0.64 (-17.75, 23.15) | -0.14 (-18.21, 21.92) |
| Saint Vincent and the Grenadines | 35 to 39 | -2.24 (-26.73, 30.44) | -2.34 (-21.29, 21.18) | -2.84 (-21.2, 19.8) |
| Saint Vincent and the Grenadines | 40 to 44 | -5.81 (-31.1, 28.75) | -6.63 (-28.07, 21.21) | -6.44 (-27.14, 20.14) |
| Saint Vincent and the Grenadines | 45 to 49 | -9.14 (-32.49, 22.29) | -9.8 (-33.04, 21.49) | -8.14 (-29.51, 19.71) |
| Saint Vincent and the Grenadines | 50 to 54 | -10.69 (-32.58, 18.3) | -12.69 (-35.4, 18.01) | -7.99 (-28.44, 18.3) |
| Saint Vincent and the Grenadines | 55 to 59 | -11.68 (-33.49, 17.28) | -14.61 (-37.85, 17.34) | -7.17 (-26.57, 17.36) |
| Saint Vincent and the Grenadines | 60 to 64 | -11.82 (-34.34, 18.41) | -14.5 (-38.05, 17.99) | -5.45 (-24.02, 17.67) |
| Saint Vincent and the Grenadines | 65 to 69 | -10 (-35.48, 25.53) | -13.78 (-37.52, 18.97) | -4.37 (-22.5, 18) |
| Saint Vincent and the Grenadines | 70 to 74 | -9.21 (-35.16, 27.13) | -13.93 (-37.48, 18.49) | -6.03 (-26.68, 20.44) |
| Saint Vincent and the Grenadines | 75 to 79 | -8.84 (-34.53, 26.93) | -12.37 (-36.36, 20.65) | -8.15 (-30.86, 22.01) |
| Saint Vincent and the Grenadines | 80 to 84 | -10.84 (-34.61, 21.57) | -11.64 (-36.58, 23.11) | -9.99 (-34.72, 24.1) |
| Saint Vincent and the Grenadines | 85 to 89 | -12.35 (-40.37, 28.82) | -10.61 (-37.47, 27.78) | -11.44 (-39.53, 29.7) |
| Samoa | 25 to 29 | -1.34 (-24.84, 29.5) | -0.49 (-23.11, 28.8) | -0.6 (-23.12, 28.51) |
| Samoa | 30 to 34 | -2.99 (-27.16, 29.19) | -0.44 (-18.66, 21.88) | -1.09 (-19.21, 21.1) |
| Samoa | 35 to 39 | -4.32 (-29.23, 29.36) | -0.95 (-19.93, 22.54) | -2.3 (-21.82, 22.09) |
| Samoa | 40 to 44 | -6.67 (-32.93, 29.87) | -3.79 (-25.35, 23.99) | -5.29 (-28.44, 25.36) |
| Samoa | 45 to 49 | -7.75 (-33.14, 27.29) | -7.53 (-30.65, 23.29) | -8.02 (-32.71, 25.74) |
| Samoa | 50 to 54 | -5.57 (-32.17, 31.46) | -9.42 (-35.61, 27.44) | -8.28 (-34.49, 28.4) |
| Samoa | 55 to 59 | -1.43 (-30.7, 40.22) | -10.41 (-37.36, 28.15) | -5.52 (-34.61, 36.5) |
| Samoa | 60 to 64 | 1.68 (-29.3, 46.22) | -7.07 (-37.9, 39.07) | -1.83 (-34.45, 47.04) |
| Samoa | 65 to 69 | 4.11 (-28.16, 50.88) | -2.36 (-36.94, 51.16) | 2.41 (-33.45, 57.59) |
| Samoa | 70 to 74 | 4.66 (-29.69, 55.81) | 0.59 (-37.14, 60.97) | 3.6 (-34.1, 62.88) |
| Samoa | 75 to 79 | 3.88 (-33.47, 62.22) | -5.99 (-37.28, 40.92) | -4.54 (-33.9, 37.85) |
| Samoa | 80 to 84 | -3 (-36.1, 47.25) | -10.77 (-39.75, 32.15) | -10.69 (-37.21, 27.03) |
| Samoa | 85 to 89 | -8.02 (-40.19, 41.46) | -14.73 (-44.81, 31.74) | -13.41 (-43.17, 31.93) |
| San Marino | 25 to 29 | 1.41 (-20.57, 29.48) | 1.41 (-20.53, 29.4) | 1.68 (-20.32, 29.74) |
| San Marino | 30 to 34 | 1.03 (-16.53, 22.29) | 1.16 (-16.41, 22.43) | 1.07 (-16.49, 22.33) |
| San Marino | 35 to 39 | -0.25 (-16.18, 18.71) | 0.05 (-15.93, 19.06) | -0.49 (-16.38, 18.42) |
| San Marino | 40 to 44 | -1.57 (-16.82, 16.47) | -1.14 (-16.46, 16.98) | -2.01 (-17.19, 15.96) |
| San Marino | 45 to 49 | -2.27 (-17.23, 15.39) | -1.8 (-16.83, 15.95) | -2.74 (-17.63, 14.83) |
| San Marino | 50 to 54 | -2.59 (-17.4, 14.87) | -2.21 (-17.06, 15.31) | -2.89 (-17.64, 14.5) |
| San Marino | 55 to 59 | -2.39 (-17.35, 15.28) | -2.21 (-17.06, 15.3) | -2.54 (-17.34, 14.91) |
| San Marino | 60 to 64 | -1.81 (-17.27, 16.54) | -2.01 (-16.99, 15.68) | -1.9 (-16.9, 15.8) |
| San Marino | 65 to 69 | -1.78 (-17.4, 16.79) | -1.98 (-17.14, 15.95) | -1.53 (-16.75, 16.49) |
| San Marino | 70 to 74 | -1.94 (-18.71, 18.29) | -2.02 (-17.59, 16.49) | -1.43 (-17.09, 17.2) |
| San Marino | 75 to 79 | -2.14 (-22.57, 23.67) | -2.7 (-18.94, 16.8) | -2.2 (-18.53, 17.39) |
| San Marino | 80 to 84 | -7.46 (-33.87, 29.5) | -3.88 (-22.84, 19.75) | -3.17 (-22.27, 20.63) |
| San Marino | 85 to 89 | -7.27 (-52.59, 81.36) | -6.01 (-31.51, 28.98) | -5.06 (-30.82, 30.28) |
| Sao Tome and Principe | 25 to 29 | -2.52 (-23.62, 24.43) | -2.78 (-23.78, 24) | -2.48 (-23.54, 24.39) |
| Sao Tome and Principe | 30 to 34 | -3.14 (-19.97, 17.23) | -3.49 (-20.25, 16.79) | -2.94 (-19.8, 17.46) |
| Sao Tome and Principe | 35 to 39 | -3.58 (-18.97, 14.75) | -3.97 (-19.3, 14.28) | -3.21 (-18.66, 15.19) |
| Sao Tome and Principe | 40 to 44 | -3.81 (-18.71, 13.83) | -4.22 (-19.06, 13.34) | -3.38 (-18.35, 14.33) |
| Sao Tome and Principe | 45 to 49 | -4.07 (-18.76, 13.28) | -4.4 (-19.03, 12.87) | -3.64 (-18.39, 13.77) |
| Sao Tome and Principe | 50 to 54 | -3.61 (-18.36, 13.8) | -3.93 (-18.52, 13.27) | -3.37 (-18.04, 13.94) |
| Sao Tome and Principe | 55 to 59 | -2.34 (-17.41, 15.47) | -2.7 (-17.47, 14.71) | -2.47 (-17.27, 14.99) |
| Sao Tome and Principe | 60 to 64 | -1.64 (-16.86, 16.37) | -1.53 (-16.49, 16.11) | -1.56 (-16.51, 16.07) |
| Sao Tome and Principe | 65 to 69 | -1.28 (-17.31, 17.85) | -0.48 (-15.71, 17.51) | -0.79 (-15.97, 17.14) |
| Sao Tome and Principe | 70 to 74 | -1.61 (-18.93, 19.4) | -0.26 (-15.71, 18.03) | -0.76 (-16.13, 17.44) |
| Sao Tome and Principe | 75 to 79 | -2.69 (-21.68, 20.9) | -0.8 (-16.64, 18.05) | -1.52 (-17.25, 17.19) |
| Sao Tome and Principe | 80 to 84 | -3.2 (-23.12, 21.9) | -1.62 (-18.7, 19.06) | -1.83 (-18.88, 18.81) |
| Sao Tome and Principe | 85 to 89 | -2.09 (-23.93, 26.02) | -2.36 (-23.45, 24.54) | -0.83 (-22.25, 26.49) |
| Saudi Arabia | 25 to 29 | 5.33 (1.03, 9.82) | 5.71 (-0.99, 12.86) | 5.04 (-0.5, 10.88) |
| Saudi Arabia | 30 to 34 | 4.88 (1.44, 8.43) | 5.22 (-0.1, 10.82) | 4.58 (0.13, 9.24) |
| Saudi Arabia | 35 to 39 | 4.32 (1.38, 7.35) | 4.45 (-0.07, 9.19) | 4.12 (0.28, 8.1) |
| Saudi Arabia | 40 to 44 | 3.79 (1.18, 6.47) | 3.69 (-0.31, 7.85) | 3.69 (0.27, 7.22) |
| Saudi Arabia | 45 to 49 | 3.25 (0.88, 5.69) | 2.99 (-0.64, 6.76) | 3.21 (0.09, 6.43) |
| Saudi Arabia | 50 to 54 | 2.67 (0.42, 4.98) | 2.5 (-0.96, 6.09) | 2.63 (-0.32, 5.67) |
| Saudi Arabia | 55 to 59 | 2.11 (-0.13, 4.4) | 2.25 (-1.21, 5.83) | 2.05 (-0.88, 5.06) |
| Saudi Arabia | 60 to 64 | 1.7 (-0.6, 4.06) | 2.13 (-1.43, 5.81) | 1.63 (-1.39, 4.74) |
| Saudi Arabia | 65 to 69 | 1.73 (-0.7, 4.22) | 2.18 (-1.53, 6.03) | 1.7 (-1.53, 5.03) |
| Saudi Arabia | 70 to 74 | 2.09 (-0.71, 4.97) | 2.4 (-1.75, 6.72) | 2.08 (-1.7, 6.02) |
| Saudi Arabia | 75 to 79 | 2.54 (-0.85, 6.05) | 2.71 (-2.29, 7.97) | 2.5 (-2.06, 7.27) |
| Saudi Arabia | 80 to 84 | 2.92 (-1.58, 7.62) | 2.99 (-3.7, 10.15) | 2.86 (-3.12, 9.2) |
| Saudi Arabia | 85 to 89 | 3.21 (-3.94, 10.89) | 3.13 (-7.5, 14.98) | 3.22 (-6.11, 13.49) |
| Senegal | 25 to 29 | 2.27 (-30.55, 50.61) | -2.67 (-40.28, 58.62) | -0.33 (-46.61, 86.09) |
| Senegal | 30 to 34 | 2.38 (-28.67, 46.94) | -4.54 (-35.65, 41.63) | -0.15 (-40.87, 68.6) |
| Senegal | 35 to 39 | 2.48 (-26.58, 43.05) | -3.52 (-35.32, 43.92) | 0.22 (-36.36, 57.82) |
| Senegal | 40 to 44 | 2.16 (-24.56, 38.36) | -2.05 (-33.42, 44.08) | -0.14 (-34.75, 52.84) |
| Senegal | 45 to 49 | 1.8 (-22.19, 33.18) | -1.18 (-31.91, 43.41) | 0.7 (-30.52, 45.95) |
| Senegal | 50 to 54 | 1.4 (-20, 28.51) | 1.28 (-26.43, 39.42) | 0.85 (-27.33, 39.95) |
| Senegal | 55 to 59 | 1.03 (-18.49, 25.23) | 1.08 (-24.78, 35.82) | 0.8 (-25.51, 36.41) |
| Senegal | 60 to 64 | 0.84 (-17.54, 23.32) | 0.52 (-23.76, 32.53) | 0.8 (-24.33, 34.26) |
| Senegal | 65 to 69 | 0.74 (-17.14, 22.48) | 0.81 (-23.18, 32.31) | 0.71 (-23.79, 33.08) |
| Senegal | 70 to 74 | 0.82 (-17.12, 22.65) | 1.37 (-23.07, 33.57) | 0.41 (-24.14, 32.92) |
| Senegal | 75 to 79 | 0.91 (-18.38, 24.76) | 1.05 (-25.19, 36.5) | 0.31 (-25.05, 34.24) |
| Senegal | 80 to 84 | 0.74 (-21.98, 30.08) | 1.48 (-31.79, 50.97) | -0.15 (-28.38, 39.19) |
| Senegal | 85 to 89 | 0.1 (-29.47, 42.07) | 2.92 (-45.62, 94.8) | -0.32 (-38.62, 61.89) |
| Serbia | 25 to 29 | 0.12 (-28.1, 39.42) | 0.23 (-35.16, 54.94) | 0.69 (-36.38, 59.36) |
| Serbia | 30 to 34 | 0.29 (-22.08, 29.09) | 0.1 (-29.35, 41.83) | -0.18 (-31.77, 46.02) |
| Serbia | 35 to 39 | 0.16 (-18.35, 22.86) | 0.44 (-23.61, 32.07) | 0.16 (-25.25, 34.2) |
| Serbia | 40 to 44 | -0.24 (-15.7, 18.06) | 0.02 (-20.61, 26.02) | -0.23 (-21.38, 26.6) |
| Serbia | 45 to 49 | -0.23 (-12.95, 14.34) | 0.03 (-17.35, 21.07) | -0.26 (-17.68, 20.85) |
| Serbia | 50 to 54 | -0.13 (-10.3, 11.2) | 0.22 (-13.82, 16.55) | -0.27 (-14.3, 16.05) |
| Serbia | 55 to 59 | -0.24 (-8.29, 8.52) | 0.14 (-11.03, 12.7) | -0.49 (-11.65, 12.07) |
| Serbia | 60 to 64 | -0.41 (-6.97, 6.61) | -0.07 (-9.25, 10.04) | -0.72 (-9.84, 9.32) |
| Serbia | 65 to 69 | -0.31 (-6.03, 5.76) | 0 (-8.1, 8.81) | -0.58 (-8.47, 7.99) |
| Serbia | 70 to 74 | 0.16 (-5.37, 6.01) | 0.4 (-7.39, 8.85) | -0.02 (-7.69, 8.29) |
| Serbia | 75 to 79 | 1.07 (-5, 7.52) | 1.19 (-7.45, 10.65) | 0.99 (-7.37, 10.11) |
| Serbia | 80 to 84 | 1.15 (-8.03, 11.25) | 1.56 (-13.37, 19.06) | 0.81 (-10.61, 13.68) |
| Serbia | 85 to 89 | 0.34 (-10.64, 12.67) | 0.33 (-16.34, 20.32) | 0.34 (-13.94, 17) |
| Seychelles | 25 to 29 | 0.07 (-21.86, 28.15) | -1.13 (-22.49, 26.1) | 0.07 (-21.54, 27.64) |
| Seychelles | 30 to 34 | -0.8 (-18.12, 20.19) | -1.52 (-18.62, 19.18) | -0.26 (-17.59, 20.7) |
| Seychelles | 35 to 39 | -1.7 (-17.65, 17.33) | -1.94 (-17.6, 16.69) | -1.08 (-16.87, 17.72) |
| Seychelles | 40 to 44 | -3.31 (-19.08, 15.52) | -2.91 (-17.95, 14.89) | -2.66 (-17.74, 15.18) |
| Seychelles | 45 to 49 | -4.9 (-21.2, 14.77) | -3.88 (-18.59, 13.49) | -4.01 (-18.7, 13.33) |
| Seychelles | 50 to 54 | -5.82 (-23.17, 15.46) | -4.47 (-18.98, 12.64) | -4.29 (-18.82, 12.86) |
| Seychelles | 55 to 59 | -5.91 (-23.72, 16.06) | -4.5 (-19, 12.59) | -3.74 (-18.35, 13.49) |
| Seychelles | 60 to 64 | -4.84 (-23, 17.59) | -3.55 (-18.2, 13.73) | -2.44 (-17.26, 15.03) |
| Seychelles | 65 to 69 | -3.29 (-21.2, 18.7) | -2.23 (-17.2, 15.43) | -1.17 (-16.29, 16.69) |
| Seychelles | 70 to 74 | -2.45 (-19.92, 18.82) | -1.59 (-16.84, 16.45) | -0.88 (-16.24, 17.29) |
| Seychelles | 75 to 79 | -2.11 (-19.16, 18.53) | -1.41 (-17.16, 17.32) | -1.2 (-16.97, 17.58) |
| Seychelles | 80 to 84 | -2.03 (-19.28, 18.9) | -1.96 (-18.98, 18.65) | -1.46 (-18.57, 19.26) |
| Seychelles | 85 to 89 | -2.18 (-23.45, 24.99) | -2.15 (-23.29, 24.8) | -1.55 (-22.82, 25.57) |
| Sierra Leone | 25 to 29 | -12.14 (-46.75, 44.98) | -9.76 (-50.29, 63.82) | -9.64 (-50.14, 63.76) |
| Sierra Leone | 30 to 34 | -13.33 (-45.87, 38.77) | -5.34 (-26.44, 21.8) | -3.63 (-23.43, 21.29) |
| Sierra Leone | 35 to 39 | -11.63 (-40.82, 31.96) | -6.11 (-29.28, 24.64) | -2.94 (-19.82, 17.51) |
| Sierra Leone | 40 to 44 | -8.51 (-34.36, 27.52) | -5.76 (-29.26, 25.54) | -4.07 (-22.34, 18.51) |
| Sierra Leone | 45 to 49 | -8.13 (-35.54, 30.95) | -6.69 (-30.98, 26.15) | -5.18 (-26.94, 23.06) |
| Sierra Leone | 50 to 54 | -5.45 (-33.36, 34.15) | -6.63 (-32.24, 28.65) | -5.94 (-30.88, 28) |
| Sierra Leone | 55 to 59 | -2.86 (-32.66, 40.11) | -4.62 (-33.54, 36.86) | -7.96 (-33.87, 28.1) |
| Sierra Leone | 60 to 64 | 0.28 (-30.55, 44.8) | -2.34 (-34.92, 46.55) | -8.86 (-35.55, 28.88) |
| Sierra Leone | 65 to 69 | 1.88 (-29.12, 46.45) | -0.2 (-35.27, 53.85) | -6 (-36.89, 40) |
| Sierra Leone | 70 to 74 | 1.74 (-28.12, 44.02) | 1.42 (-34.99, 58.23) | -3.34 (-38.17, 51.12) |
| Sierra Leone | 75 to 79 | 0.54 (-28.76, 41.89) | 0.82 (-36.25, 59.46) | -0.8 (-39.38, 62.34) |
| Sierra Leone | 80 to 84 | 0.54 (-32.54, 49.82) | -0.9 (-43.68, 74.36) | 1.31 (-42.51, 78.53) |
| Sierra Leone | 85 to 89 | 1.29 (-47.94, 97.07) | -1.99 (-53.71, 107.51) | 0.01 (-52.77, 111.76) |
| Singapore | 25 to 29 | -11.24 (-52.98, 67.55) | -1.17 (-23.12, 27.04) | -1.55 (-23.16, 26.14) |
| Singapore | 30 to 34 | -10.8 (-44.49, 43.33) | -2.44 (-20.51, 19.74) | -2.15 (-20.21, 19.99) |
| Singapore | 35 to 39 | -8.45 (-37.98, 35.14) | -3.35 (-23.51, 22.11) | -2.32 (-21.97, 22.26) |
| Singapore | 40 to 44 | -2.8 (-28.26, 31.69) | -3.78 (-28.34, 29.19) | -2.62 (-25.76, 27.73) |
| Singapore | 45 to 49 | -1.28 (-25.12, 30.13) | -3.09 (-28.91, 32.11) | -1.02 (-24.75, 30.2) |
| Singapore | 50 to 54 | 0.4 (-20.98, 27.55) | -1.12 (-26.71, 33.42) | -0.12 (-23.69, 30.74) |
| Singapore | 55 to 59 | 2.27 (-16.72, 25.59) | 0.28 (-25.19, 34.42) | 1.38 (-21.14, 30.33) |
| Singapore | 60 to 64 | 3.04 (-13.72, 23.04) | 1.9 (-22.28, 33.61) | 3.24 (-17.39, 29.02) |
| Singapore | 65 to 69 | 3.57 (-12.17, 22.12) | 3.1 (-20.61, 33.91) | 5.07 (-14.33, 28.85) |
| Singapore | 70 to 74 | 3.85 (-11.81, 22.31) | 2.81 (-21.65, 34.91) | 4.67 (-14.06, 27.48) |
| Singapore | 75 to 79 | 4.18 (-11.72, 22.95) | 3.03 (-23.04, 37.93) | 4.99 (-13.74, 27.79) |
| Singapore | 80 to 84 | 4.05 (-14.01, 25.91) | 3.49 (-29.41, 51.73) | 4.48 (-16.03, 30.01) |
| Singapore | 85 to 89 | 4.23 (-21.44, 38.28) | 2.7 (-36.12, 65.11) | 4.77 (-26.62, 49.59) |
| Slovakia | 25 to 29 | 2.47 (-35.64, 63.14) | -0.29 (-51.5, 104.99) | -8.23 (-52.43, 77.04) |
| Slovakia | 30 to 34 | 1.22 (-30.65, 47.74) | -0.33 (-41.92, 71.04) | -8.71 (-43.37, 47.17) |
| Slovakia | 35 to 39 | 0.59 (-25.89, 36.54) | -0.11 (-33.72, 50.56) | -6.67 (-37.92, 40.31) |
| Slovakia | 40 to 44 | -0.04 (-21.71, 27.63) | -0.8 (-28.93, 38.47) | -3.34 (-30.25, 33.97) |
| Slovakia | 45 to 49 | -0.55 (-18.34, 21.12) | -1.12 (-24.47, 29.46) | -2 (-26.14, 30.03) |
| Slovakia | 50 to 54 | -0.79 (-14.83, 15.56) | -1.22 (-20.32, 22.45) | -0.1 (-19.47, 23.92) |
| Slovakia | 55 to 59 | -1.14 (-12.42, 11.6) | -1.21 (-16.84, 17.37) | -1.02 (-16.56, 17.42) |
| Slovakia | 60 to 64 | -1.21 (-10.29, 8.79) | -1.15 (-14.19, 13.88) | -1.42 (-13.6, 12.48) |
| Slovakia | 65 to 69 | -1.17 (-9.11, 7.46) | -1.14 (-13.04, 12.39) | -1.37 (-11.7, 10.18) |
| Slovakia | 70 to 74 | -0.66 (-8.15, 7.45) | -0.8 (-12.36, 12.27) | -0.7 (-10.27, 9.89) |
| Slovakia | 75 to 79 | 0.17 (-8.44, 9.59) | -0.18 (-13.62, 15.35) | 0.41 (-10.51, 12.66) |
| Slovakia | 80 to 84 | 0.87 (-8.66, 11.4) | 0.3 (-14.65, 17.87) | 1.31 (-10.72, 14.95) |
| Slovakia | 85 to 89 | 1.21 (-12.43, 16.99) | 0.32 (-20.47, 26.54) | 1.75 (-15.1, 21.95) |
| Slovenia | 25 to 29 | 0.95 (-52.35, 113.87) | 1.98 (-21.74, 32.88) | 1.17 (-21.69, 30.69) |
| Slovenia | 30 to 34 | -0.24 (-44.42, 79.05) | -0.86 (-22.38, 26.63) | -0.06 (-21, 26.41) |
| Slovenia | 35 to 39 | -1.3 (-40.53, 63.82) | -3 (-32.76, 39.94) | -3.42 (-28.22, 29.95) |
| Slovenia | 40 to 44 | -1.9 (-36.57, 51.73) | -3.45 (-39, 52.84) | -4.54 (-33.95, 37.97) |
| Slovenia | 45 to 49 | -1.54 (-31.37, 41.25) | 0.08 (-34.95, 53.96) | -3.7 (-33.97, 40.46) |
| Slovenia | 50 to 54 | -1.26 (-26.17, 32.06) | -0.25 (-33.65, 49.97) | -2.34 (-30.81, 37.85) |
| Slovenia | 55 to 59 | -0.72 (-21.77, 25.99) | -0.56 (-30.42, 42.12) | -0.8 (-27.14, 35.07) |
| Slovenia | 60 to 64 | -0.52 (-18.58, 21.54) | -0.9 (-27.6, 35.64) | 0.72 (-22.56, 31) |
| Slovenia | 65 to 69 | -0.24 (-16.49, 19.18) | -0.88 (-25.86, 32.53) | -0.01 (-20.86, 26.33) |
| Slovenia | 70 to 74 | 0.38 (-15.09, 18.68) | -0.06 (-24.63, 32.52) | 0.54 (-18.72, 24.35) |
| Slovenia | 75 to 79 | 1.77 (-14.18, 20.68) | 1.42 (-25.25, 37.6) | 1.76 (-17.36, 25.31) |
| Slovenia | 80 to 84 | 3.19 (-13.5, 23.08) | 3.07 (-25.2, 42.03) | 3.27 (-16.43, 27.61) |
| Slovenia | 85 to 89 | 5.27 (-20.44, 39.3) | 4.31 (-33.84, 64.47) | 5.85 (-25.83, 51.07) |
| Solomon Islands | 25 to 29 | -2.74 (-24.95, 26.04) | -2.08 (-23.6, 25.51) | -1.87 (-23.29, 25.54) |
| Solomon Islands | 30 to 34 | -3.83 (-23.67, 21.17) | -2.7 (-20.44, 18.99) | -3 (-20.45, 18.27) |
| Solomon Islands | 35 to 39 | -5.32 (-30.82, 29.57) | -4.49 (-22.59, 17.84) | -4.74 (-21.95, 16.26) |
| Solomon Islands | 40 to 44 | -8.78 (-36.37, 30.78) | -7.15 (-27.85, 19.48) | -6.1 (-24.97, 17.51) |
| Solomon Islands | 45 to 49 | -10.78 (-38.63, 29.7) | -8.23 (-31.2, 22.42) | -6.4 (-25.51, 17.6) |
| Solomon Islands | 50 to 54 | -8.98 (-40.06, 38.21) | -8.34 (-31.58, 22.78) | -5.77 (-25.2, 18.7) |
| Solomon Islands | 55 to 59 | -6.04 (-40.98, 49.58) | -7.76 (-31, 23.3) | -4.61 (-22.6, 17.57) |
| Solomon Islands | 60 to 64 | -4.32 (-41.22, 55.73) | -5.94 (-27.33, 21.75) | -3.31 (-19.42, 16.04) |
| Solomon Islands | 65 to 69 | -3.32 (-41.05, 58.57) | -4.01 (-22.44, 18.8) | -2.93 (-18.5, 15.61) |
| Solomon Islands | 70 to 74 | -5.08 (-40.76, 52.09) | -3.37 (-20.43, 17.35) | -3.21 (-18.22, 14.56) |
| Solomon Islands | 75 to 79 | -8.79 (-39.35, 37.18) | -3 (-18.99, 16.13) | -4.37 (-19.74, 13.95) |
| Solomon Islands | 80 to 84 | -8.73 (-34.68, 27.52) | -3.64 (-20.79, 17.21) | -5.29 (-21.77, 14.66) |
| Solomon Islands | 85 to 89 | -5.95 (-28.16, 23.14) | -4.29 (-25.28, 22.59) | -5.65 (-26.1, 20.45) |
| Somalia | 25 to 29 | -7.32 (-43.65, 52.45) | -14.06 (-54.53, 62.41) | -5.55 (-28.41, 24.61) |
| Somalia | 30 to 34 | -4.97 (-36.43, 42.05) | -12.33 (-44.88, 39.44) | -7.33 (-30.92, 24.32) |
| Somalia | 35 to 39 | -3.31 (-31.96, 37.39) | -7.4 (-37.02, 36.16) | -6.41 (-32.06, 28.91) |
| Somalia | 40 to 44 | -1.71 (-26.2, 30.89) | -2.95 (-29.82, 34.21) | -3.83 (-30.42, 32.93) |
| Somalia | 45 to 49 | -0.37 (-21.99, 27.22) | 0.34 (-24.42, 33.21) | -0.51 (-26.06, 33.87) |
| Somalia | 50 to 54 | 0.73 (-17.65, 23.21) | 0.86 (-22.31, 30.95) | 1.04 (-22.86, 32.35) |
| Somalia | 55 to 59 | -0.03 (-15.36, 18.07) | 0.38 (-20.68, 27.03) | 1.01 (-20.26, 27.96) |
| Somalia | 60 to 64 | -0.04 (-14.35, 16.67) | 0.15 (-19.62, 24.78) | 0.43 (-19.3, 24.98) |
| Somalia | 65 to 69 | 0.14 (-15, 17.98) | 0.21 (-20.83, 26.83) | 0.53 (-19.72, 25.88) |
| Somalia | 70 to 74 | 0.25 (-17.07, 21.19) | 0.31 (-23.8, 32.04) | 0.34 (-22.52, 29.95) |
| Somalia | 75 to 79 | 0.44 (-22.06, 29.45) | 0.34 (-30.47, 44.8) | 0.91 (-28.9, 43.24) |
| Somalia | 80 to 84 | 0.44 (-27.67, 39.47) | 0.91 (-39.78, 69.09) | 0.38 (-34.28, 53.3) |
| Somalia | 85 to 89 | 0.63 (-38.95, 65.87) | 1.25 (-51.46, 111.18) | 0.64 (-48.29, 95.88) |
| South Africa | 25 to 29 | 0.6 (-6.13, 7.81) | 1.7 (-7.76, 12.15) | -0.39 (-9.8, 10.01) |
| South Africa | 30 to 34 | 1.42 (-3.83, 6.95) | 1.9 (-5.6, 9.99) | 1.08 (-6.19, 8.92) |
| South Africa | 35 to 39 | 2.84 (-1.94, 7.86) | 2.57 (-4.2, 9.82) | 3.18 (-3.5, 10.31) |
| South Africa | 40 to 44 | 4.08 (-0.38, 8.73) | 3.17 (-3.04, 9.78) | 5.03 (-1.32, 11.8) |
| South Africa | 45 to 49 | 4.31 (0.11, 8.67) | 3.27 (-2.46, 9.35) | 5.43 (-0.67, 11.92) |
| South Africa | 50 to 54 | 3.88 (-0.06, 7.97) | 3.04 (-2.32, 8.69) | 4.84 (-0.93, 10.93) |
| South Africa | 55 to 59 | 3.53 (-0.24, 7.44) | 3.01 (-2.15, 8.44) | 4.13 (-1.33, 9.89) |
| South Africa | 60 to 64 | 3.25 (-0.45, 7.08) | 3 (-2.12, 8.38) | 3.49 (-1.81, 9.07) |
| South Africa | 65 to 69 | 3.2 (-0.55, 7.08) | 2.96 (-2.27, 8.47) | 3.36 (-1.95, 8.96) |
| South Africa | 70 to 74 | 3.18 (-0.69, 7.2) | 2.91 (-2.58, 8.7) | 3.39 (-2.04, 9.11) |
| South Africa | 75 to 79 | 3.27 (-0.91, 7.63) | 2.9 (-3.21, 9.41) | 3.57 (-2.13, 9.59) |
| South Africa | 80 to 84 | 2.95 (-1.85, 7.99) | 2.63 (-4.63, 10.45) | 3.24 (-3.12, 10.01) |
| South Africa | 85 to 89 | 2.88 (-3.51, 9.7) | 2.3 (-7.35, 12.95) | 3.32 (-5.01, 12.38) |
| South Sudan | 25 to 29 | 4.66 (-32.04, 61.17) | 3.63 (-43.14, 88.87) | -4.16 (-41.71, 57.57) |
| South Sudan | 30 to 34 | 3.81 (-25.56, 44.78) | 3.26 (-34.46, 62.68) | -2.33 (-34.75, 46.18) |
| South Sudan | 35 to 39 | 3.37 (-20.5, 34.4) | 2.97 (-27.18, 45.61) | -0.35 (-30.36, 42.59) |
| South Sudan | 40 to 44 | 2.83 (-16.63, 26.83) | 2.58 (-22.22, 35.27) | 1.34 (-25.15, 37.19) |
| South Sudan | 45 to 49 | 2.57 (-13.61, 21.78) | 2.41 (-18.31, 28.4) | 2.33 (-20.98, 32.52) |
| South Sudan | 50 to 54 | 2.36 (-11.17, 17.95) | 2.2 (-15.25, 23.24) | 3.28 (-16.79, 28.19) |
| South Sudan | 55 to 59 | 2.18 (-9.33, 15.15) | 1.8 (-13.07, 19.21) | 2.48 (-14.74, 23.19) |
| South Sudan | 60 to 64 | 1.98 (-8.38, 13.51) | 1.51 (-11.67, 16.65) | 2.37 (-13.56, 21.24) |
| South Sudan | 65 to 69 | 1.79 (-8.04, 12.68) | 1.25 (-11.15, 15.37) | 2.39 (-13.2, 20.78) |
| South Sudan | 70 to 74 | 1.48 (-7.77, 11.65) | 0.96 (-10.52, 13.91) | 2.38 (-12.86, 20.28) |
| South Sudan | 75 to 79 | 1.5 (-8.66, 12.8) | 0.91 (-11.86, 15.54) | 2.51 (-13.75, 21.83) |
| South Sudan | 80 to 84 | 1.91 (-11.46, 17.31) | 1.27 (-16.46, 22.77) | 2.74 (-16.25, 26.04) |
| South Sudan | 85 to 89 | 2.33 (-18.18, 27.98) | 1.54 (-26.89, 41.03) | 2.84 (-22.9, 37.18) |
| Spain | 25 to 29 | -0.56 (-18.58, 21.46) | -1.1 (-24.25, 29.12) | 0.64 (-26.03, 36.92) |
| Spain | 30 to 34 | -1.35 (-15.59, 15.3) | -2.15 (-20.04, 19.72) | -0.13 (-22.17, 28.15) |
| Spain | 35 to 39 | -1.64 (-13.35, 11.66) | -2.58 (-17.41, 14.9) | -0.34 (-18.91, 22.48) |
| Spain | 40 to 44 | -1.57 (-11.49, 9.46) | -2.31 (-14.75, 11.93) | -0.38 (-16.28, 18.54) |
| Spain | 45 to 49 | -1.12 (-9.35, 7.87) | -1.6 (-11.99, 10.02) | -0.38 (-13.57, 14.83) |
| Spain | 50 to 54 | -1.07 (-7.74, 6.09) | -1.14 (-9.71, 8.24) | -0.78 (-11.18, 10.84) |
| Spain | 55 to 59 | -1.38 (-6.82, 4.39) | -1.11 (-8.25, 6.58) | -1.6 (-9.86, 7.42) |
| Spain | 60 to 64 | -1.58 (-5.96, 3.01) | -1.13 (-7.02, 5.13) | -2.09 (-8.59, 4.88) |
| Spain | 65 to 69 | -1.71 (-5.28, 1.99) | -1.16 (-6.06, 3.99) | -2.31 (-7.49, 3.16) |
| Spain | 70 to 74 | -1.53 (-4.4, 1.42) | -1.05 (-5.12, 3.19) | -2.02 (-6.05, 2.19) |
| Spain | 75 to 79 | -1.09 (-3.49, 1.37) | -0.73 (-4.33, 3.01) | -1.43 (-4.67, 1.91) |
| Spain | 80 to 84 | -0.7 (-2.74, 1.38) | -0.5 (-3.78, 2.9) | -0.87 (-3.48, 1.8) |
| Spain | 85 to 89 | -0.96 (-3.24, 1.37) | -0.98 (-4.8, 2.99) | -0.94 (-3.76, 1.97) |
| Sri Lanka | 25 to 29 | 2.36 (-32.49, 55.2) | 3.46 (-39.44, 76.72) | 0.9 (-48.82, 98.9) |
| Sri Lanka | 30 to 34 | 1.86 (-20.65, 30.77) | 2.06 (-26.95, 42.59) | 1.68 (-30.17, 48.07) |
| Sri Lanka | 35 to 39 | 1.8 (-13.91, 20.37) | 1.78 (-18.65, 27.34) | 1.9 (-20.9, 31.29) |
| Sri Lanka | 40 to 44 | 1.65 (-10.56, 15.52) | 1.63 (-14.07, 20.19) | 1.79 (-16.47, 24.04) |
| Sri Lanka | 45 to 49 | 1.68 (-8.22, 12.65) | 1.67 (-11.16, 16.36) | 1.84 (-13.03, 19.24) |
| Sri Lanka | 50 to 54 | 1.61 (-6.79, 10.76) | 1.72 (-9.2, 13.96) | 1.6 (-11.06, 16.05) |
| Sri Lanka | 55 to 59 | 1.42 (-6.11, 9.54) | 1.62 (-8.29, 12.6) | 1.3 (-9.95, 13.96) |
| Sri Lanka | 60 to 64 | 1.29 (-5.75, 8.85) | 1.54 (-7.87, 11.92) | 1.1 (-9.24, 12.62) |
| Sri Lanka | 65 to 69 | 1.35 (-5.56, 8.78) | 1.59 (-7.79, 11.93) | 1.18 (-8.82, 12.28) |
| Sri Lanka | 70 to 74 | 1.67 (-5.48, 9.35) | 1.87 (-7.96, 12.75) | 1.53 (-8.63, 12.81) |
| Sri Lanka | 75 to 79 | 2.29 (-5.83, 11.11) | 2.44 (-8.8, 15.06) | 2.2 (-9.22, 15.05) |
| Sri Lanka | 80 to 84 | 2.47 (-7.43, 13.42) | 2.58 (-11.17, 18.45) | 2.42 (-11.32, 18.29) |
| Sri Lanka | 85 to 89 | 1.95 (-12.67, 19.02) | 2.17 (-18.08, 27.43) | 1.89 (-18.04, 26.68) |
| Sudan | 25 to 29 | 3.07 (-16.8, 27.7) | 2.83 (-27.03, 44.91) | 3.39 (-21.44, 36.08) |
| Sudan | 30 to 34 | 3.26 (-13.64, 23.46) | 3.02 (-22.62, 37.15) | 3.48 (-17.86, 30.37) |
| Sudan | 35 to 39 | 3.12 (-12.18, 21.07) | 2.82 (-20.53, 33.03) | 3.2 (-16.01, 26.8) |
| Sudan | 40 to 44 | 2.81 (-11.1, 18.89) | 2.44 (-18.8, 29.24) | 2.94 (-14.5, 23.94) |
| Sudan | 45 to 49 | 2.34 (-10.21, 16.63) | 1.93 (-17.02, 25.21) | 2.56 (-13.39, 21.43) |
| Sudan | 50 to 54 | 1.95 (-9.47, 14.8) | 1.7 (-15.48, 22.36) | 2.12 (-12.54, 19.23) |
| Sudan | 55 to 59 | 1.81 (-8.96, 13.87) | 1.66 (-14.25, 20.53) | 1.89 (-12.13, 18.14) |
| Sudan | 60 to 64 | 1.85 (-8.43, 13.3) | 1.76 (-13.07, 19.13) | 1.96 (-11.75, 17.79) |
| Sudan | 65 to 69 | 2.07 (-8.15, 13.44) | 1.95 (-12.42, 18.69) | 2.19 (-11.8, 18.39) |
| Sudan | 70 to 74 | 2.32 (-7.9, 13.68) | 2.23 (-11.84, 18.55) | 2.49 (-11.79, 19.07) |
| Sudan | 75 to 79 | 2.56 (-8.57, 15.05) | 2.41 (-12.73, 20.17) | 2.82 (-12.82, 21.26) |
| Sudan | 80 to 84 | 2.81 (-12.14, 20.31) | 2.36 (-17.02, 26.27) | 3.13 (-17.95, 29.63) |
| Sudan | 85 to 89 | 2.84 (-20.84, 33.6) | 2.72 (-28.7, 48.01) | 2.81 (-29.33, 49.58) |
| Suriname | 25 to 29 | 2.31 (-31.46, 52.72) | 3.45 (-42.31, 85.49) | 4.84 (-39.1, 80.47) |
| Suriname | 30 to 34 | 2.83 (-24.05, 39.23) | 2.73 (-33.41, 58.47) | 4.71 (-32.48, 62.37) |
| Suriname | 35 to 39 | 2.61 (-21.66, 34.4) | 1.8 (-30.45, 49.01) | 4.17 (-29.98, 55) |
| Suriname | 40 to 44 | 2.32 (-20.07, 30.98) | 1.97 (-27.01, 42.46) | 3.53 (-29.31, 51.62) |
| Suriname | 45 to 49 | 1.98 (-18.66, 27.86) | 1.8 (-23.92, 36.22) | 2.79 (-28, 46.74) |
| Suriname | 50 to 54 | 1.81 (-17.34, 25.4) | 1.5 (-21.92, 31.95) | 1.45 (-26.78, 40.56) |
| Suriname | 55 to 59 | 1.62 (-16.63, 23.87) | 1.86 (-20.51, 30.52) | 0.6 (-26.91, 38.46) |
| Suriname | 60 to 64 | 1.28 (-16.99, 23.57) | 1.74 (-20.98, 30.98) | 0.42 (-27.41, 38.91) |
| Suriname | 65 to 69 | 1.39 (-18.04, 25.43) | 1.72 (-22.04, 32.72) | 0.97 (-29.25, 44.11) |
| Suriname | 70 to 74 | 1.95 (-19.78, 29.55) | 2.3 (-24.45, 38.53) | 1.95 (-30.39, 49.31) |
| Suriname | 75 to 79 | 2.5 (-22.27, 35.18) | 3.42 (-30.77, 54.5) | 3.29 (-32.63, 58.34) |
| Suriname | 80 to 84 | 3.1 (-29.33, 50.42) | 3.15 (-37, 68.87) | 2.48 (-37.59, 68.25) |
| Suriname | 85 to 89 | 2.22 (-37.05, 66) | 2.48 (-47.97, 101.87) | 2.01 (-47.78, 99.26) |
| Sweden | 25 to 29 | -7.61 (-42.56, 48.6) | -11.79 (-53.09, 65.87) | -10.99 (-52.51, 66.83) |
| Sweden | 30 to 34 | -8.77 (-38.75, 35.89) | -11.19 (-44.36, 41.76) | -7.74 (-40.28, 42.52) |
| Sweden | 35 to 39 | -8.46 (-35.98, 30.88) | -8.4 (-37.69, 34.68) | -2.24 (-23.71, 25.26) |
| Sweden | 40 to 44 | -6.13 (-32.26, 30.08) | -3.03 (-27.55, 29.8) | -2.79 (-25.08, 26.13) |
| Sweden | 45 to 49 | -0.68 (-25.57, 32.55) | -1.51 (-26.64, 32.23) | -1.66 (-23.98, 27.2) |
| Sweden | 50 to 54 | 1.88 (-19.72, 29.29) | 1.09 (-22.66, 32.13) | 0.63 (-21.99, 29.81) |
| Sweden | 55 to 59 | 3.41 (-15.08, 25.92) | 2.9 (-18.96, 30.65) | 2.54 (-19.69, 30.92) |
| Sweden | 60 to 64 | 3.85 (-10.64, 20.68) | 3.15 (-15.52, 25.95) | 3.76 (-15.89, 28.02) |
| Sweden | 65 to 69 | 3.15 (-8.27, 15.98) | 2.82 (-12.12, 20.3) | 4.3 (-11.93, 23.52) |
| Sweden | 70 to 74 | 2.83 (-6.05, 12.55) | 2.31 (-9.35, 15.47) | 4.16 (-8.81, 18.97) |
| Sweden | 75 to 79 | 3.31 (-4.17, 11.38) | 2.58 (-7.29, 13.51) | 4.38 (-6.41, 16.4) |
| Sweden | 80 to 84 | 4.34 (-2.38, 11.52) | 3.2 (-5.93, 13.22) | 5.41 (-4.06, 15.82) |
| Sweden | 85 to 89 | 5.33 (-2.87, 14.22) | 3.77 (-7.85, 16.85) | 6.19 (-4.9, 18.57) |
| Switzerland | 25 to 29 | -0.79 (-53.05, 109.61) | -2.56 (-49.55, 88.2) | -6.73 (-48.66, 69.44) |
| Switzerland | 30 to 34 | -0.27 (-44.32, 78.63) | -4.24 (-32.78, 36.42) | -1.2 (-22.26, 25.56) |
| Switzerland | 35 to 39 | 0.19 (-39.4, 65.67) | -7.29 (-39.35, 41.73) | -1.25 (-21.42, 24.08) |
| Switzerland | 40 to 44 | 0.91 (-34.42, 55.28) | -4.24 (-31.89, 34.63) | -1.68 (-25.3, 29.42) |
| Switzerland | 45 to 49 | 1.55 (-26.97, 41.21) | -3.2 (-30.19, 34.22) | -0.92 (-25.46, 31.71) |
| Switzerland | 50 to 54 | 1.33 (-21.57, 30.92) | -1.36 (-25.66, 30.89) | -0.31 (-25.28, 32.99) |
| Switzerland | 55 to 59 | 0.66 (-18.12, 23.76) | -0.39 (-23.15, 29.11) | 0.4 (-22.84, 30.64) |
| Switzerland | 60 to 64 | 0.33 (-14.75, 18.09) | 0.48 (-19.21, 24.98) | 0.58 (-19.85, 26.23) |
| Switzerland | 65 to 69 | -0.12 (-12.48, 13.98) | 0.23 (-16.69, 20.58) | 0.15 (-16.68, 20.37) |
| Switzerland | 70 to 74 | 0.17 (-9.78, 11.22) | 0.06 (-14.12, 16.58) | 0.32 (-13.02, 15.69) |
| Switzerland | 75 to 79 | 0.81 (-7.58, 9.96) | 0.61 (-11.77, 14.71) | 1.1 (-9.9, 13.45) |
| Switzerland | 80 to 84 | 2.21 (-5.1, 10.08) | 1.75 (-9.72, 14.67) | 2.7 (-6.64, 12.97) |
| Switzerland | 85 to 89 | 3.29 (-5.07, 12.39) | 2.17 (-11.21, 17.56) | 3.79 (-6.55, 15.28) |
| Syrian Arab Republic | 25 to 29 | 0.4 (-22.57, 30.19) | 0.66 (-34.91, 55.66) | 0.01 (-27.79, 38.51) |
| Syrian Arab Republic | 30 to 34 | 0.03 (-13.16, 15.22) | 0.56 (-18.53, 24.13) | -0.48 (-17.74, 20.4) |
| Syrian Arab Republic | 35 to 39 | -0.58 (-11.02, 11.08) | -0.31 (-15.53, 17.66) | -0.85 (-14.64, 15.17) |
| Syrian Arab Republic | 40 to 44 | -0.8 (-9.85, 9.17) | -0.71 (-14.05, 14.71) | -0.85 (-12.78, 12.72) |
| Syrian Arab Republic | 45 to 49 | -1.07 (-8.82, 7.34) | -1.08 (-12.66, 12.03) | -1.02 (-11.18, 10.31) |
| Syrian Arab Republic | 50 to 54 | -1.22 (-8.01, 6.07) | -1.35 (-11.5, 9.96) | -1.13 (-10.03, 8.65) |
| Syrian Arab Republic | 55 to 59 | -1.36 (-7.56, 5.25) | -1.51 (-10.8, 8.76) | -1.29 (-9.41, 7.56) |
| Syrian Arab Republic | 60 to 64 | -1.4 (-7.21, 4.78) | -1.52 (-10.28, 8.09) | -1.32 (-8.95, 6.95) |
| Syrian Arab Republic | 65 to 69 | -1.52 (-7.34, 4.66) | -1.57 (-10.27, 7.97) | -1.56 (-9.23, 6.75) |
| Syrian Arab Republic | 70 to 74 | -1.44 (-7.65, 5.18) | -1.4 (-10.59, 8.74) | -1.6 (-9.83, 7.37) |
| Syrian Arab Republic | 75 to 79 | -1.29 (-8.27, 6.23) | -1.18 (-11.52, 10.37) | -1.44 (-10.68, 8.76) |
| Syrian Arab Republic | 80 to 84 | -0.75 (-9.46, 8.79) | -0.94 (-13.44, 13.37) | -0.52 (-12.24, 12.78) |
| Syrian Arab Republic | 85 to 89 | -0.52 (-12.59, 13.22) | -0.72 (-18.02, 20.24) | -0.12 (-16.22, 19.07) |
| Taiwan (Province of China) | 25 to 29 | 1.35 (-31.82, 50.66) | 1.52 (-38.6, 67.86) | 0.83 (-48.49, 97.35) |
| Taiwan (Province of China) | 30 to 34 | 2.07 (-18.15, 27.28) | 1.53 (-25.87, 39.06) | 1.53 (-28.66, 44.48) |
| Taiwan (Province of China) | 35 to 39 | 2.61 (-11.24, 18.61) | 2.82 (-15.55, 25.19) | 2.3 (-17.73, 27.21) |
| Taiwan (Province of China) | 40 to 44 | 2.99 (-7.83, 15.08) | 3.56 (-10.86, 20.31) | 2.35 (-13.28, 20.79) |
| Taiwan (Province of China) | 45 to 49 | 3.11 (-5.88, 12.96) | 3.78 (-8.49, 17.7) | 2.31 (-10.45, 16.89) |
| Taiwan (Province of China) | 50 to 54 | 2.79 (-4.5, 10.63) | 3.57 (-6.57, 14.8) | 1.87 (-8.4, 13.3) |
| Taiwan (Province of China) | 55 to 59 | 2.25 (-3.73, 8.61) | 3.08 (-5.37, 12.27) | 1.34 (-6.98, 10.41) |
| Taiwan (Province of China) | 60 to 64 | 1.77 (-3.2, 7) | 2.46 (-4.63, 10.08) | 1.03 (-5.8, 8.36) |
| Taiwan (Province of China) | 65 to 69 | 1.6 (-2.8, 6.21) | 2.2 (-4.12, 8.92) | 0.93 (-5.1, 7.35) |
| Taiwan (Province of China) | 70 to 74 | 1.39 (-2.75, 5.71) | 1.94 (-4.07, 8.33) | 0.76 (-4.86, 6.71) |
| Taiwan (Province of China) | 75 to 79 | 0.89 (-3.01, 4.96) | 1.48 (-4.33, 7.65) | 0.27 (-4.92, 5.75) |
| Taiwan (Province of China) | 80 to 84 | 0.02 (-4.07, 4.28) | 0.6 (-5.62, 7.24) | -0.48 (-5.83, 5.18) |
| Taiwan (Province of China) | 85 to 89 | -0.75 (-6.25, 5.07) | -0.19 (-8.87, 9.31) | -1.15 (-8.13, 6.36) |
| Tajikistan | 25 to 29 | -8.89 (-49.43, 64.17) | -2.84 (-23.82, 23.93) | -2.65 (-23.68, 24.17) |
| Tajikistan | 30 to 34 | -2.88 (-22.56, 21.8) | -2.55 (-19.48, 17.93) | -2.42 (-19.36, 18.09) |
| Tajikistan | 35 to 39 | -2.61 (-18.97, 17.06) | -2.46 (-18.04, 16.08) | -2.32 (-17.92, 16.24) |
| Tajikistan | 40 to 44 | -4.41 (-21.28, 16.07) | -3.18 (-18.18, 14.56) | -3.14 (-18.14, 14.62) |
| Tajikistan | 45 to 49 | -6.93 (-25.17, 15.75) | -4.79 (-19.36, 12.42) | -5 (-19.54, 12.17) |
| Tajikistan | 50 to 54 | -7.58 (-26.06, 15.52) | -5.07 (-19.49, 11.93) | -5.63 (-19.97, 11.27) |
| Tajikistan | 55 to 59 | -6.46 (-24.95, 16.59) | -4.12 (-18.68, 13.04) | -4.5 (-19, 12.59) |
| Tajikistan | 60 to 64 | -3.86 (-20.89, 16.83) | -2.47 (-17.28, 15) | -2.57 (-17.37, 14.88) |
| Tajikistan | 65 to 69 | -1.28 (-16.57, 16.8) | -1.06 (-16.21, 16.81) | -0.82 (-16, 17.1) |
| Tajikistan | 70 to 74 | -0.67 (-16.08, 17.58) | -1.51 (-16.77, 16.54) | 0.11 (-15.4, 18.46) |
| Tajikistan | 75 to 79 | -1.8 (-17.49, 16.88) | -3.34 (-18.78, 15.02) | -0.67 (-16.53, 18.2) |
| Tajikistan | 80 to 84 | -2.35 (-19.33, 18.2) | -3.89 (-20.58, 16.31) | -1.15 (-18.32, 19.63) |
| Tajikistan | 85 to 89 | -1.88 (-23.14, 25.26) | -2.88 (-23.86, 23.87) | -0.75 (-22.18, 26.6) |
| Thailand | 25 to 29 | 2.03 (-11.83, 18.07) | 2.64 (-16.38, 25.98) | 1.39 (-17.77, 25) |
| Thailand | 30 to 34 | 2.71 (-5.21, 11.28) | 2.96 (-7.9, 15.1) | 2.28 (-8.91, 14.84) |
| Thailand | 35 to 39 | 3.23 (-2.13, 8.88) | 3.35 (-4.05, 11.31) | 2.89 (-4.74, 11.12) |
| Thailand | 40 to 44 | 3.38 (-0.76, 7.69) | 3.48 (-2.29, 9.58) | 3.11 (-2.76, 9.34) |
| Thailand | 45 to 49 | 3.53 (0.13, 7.05) | 3.76 (-1.09, 8.85) | 3.22 (-1.51, 8.17) |
| Thailand | 50 to 54 | 3.41 (0.48, 6.42) | 3.81 (-0.48, 8.29) | 2.99 (-0.99, 7.12) |
| Thailand | 55 to 59 | 3.23 (0.61, 5.92) | 3.71 (-0.23, 7.81) | 2.81 (-0.68, 6.43) |
| Thailand | 60 to 64 | 3.3 (0.86, 5.81) | 3.78 (-0.01, 7.71) | 2.96 (-0.23, 6.25) |
| Thailand | 65 to 69 | 3.47 (1.03, 5.96) | 3.94 (0.04, 8) | 3.17 (0.06, 6.38) |
| Thailand | 70 to 74 | 3.14 (0.6, 5.74) | 3.59 (-0.61, 7.97) | 2.9 (-0.28, 6.18) |
| Thailand | 75 to 79 | 2.77 (-0.02, 5.64) | 3.13 (-1.67, 8.16) | 2.62 (-0.81, 6.16) |
| Thailand | 80 to 84 | 2.03 (-1.32, 5.49) | 2.45 (-3.51, 8.79) | 1.86 (-2.17, 6.05) |
| Thailand | 85 to 89 | 1.11 (-3.62, 6.07) | 1.42 (-6.88, 10.46) | 0.95 (-4.72, 6.97) |
| Timor-Leste | 25 to 29 | -1.31 (-23.22, 26.85) | -1.01 (-22.51, 26.45) | -1.44 (-22.84, 25.91) |
| Timor-Leste | 30 to 34 | -0.89 (-18.42, 20.41) | -1.07 (-18.27, 19.73) | -1.31 (-18.46, 19.45) |
| Timor-Leste | 35 to 39 | -1.1 (-17.28, 18.25) | -1.15 (-17.03, 17.76) | -1.09 (-16.98, 17.83) |
| Timor-Leste | 40 to 44 | -1.7 (-18.29, 18.26) | -1.2 (-16.74, 17.23) | -0.85 (-16.44, 17.67) |
| Timor-Leste | 45 to 49 | -3.42 (-21.34, 18.59) | -1.94 (-17.33, 16.31) | -1.11 (-16.85, 17.6) |
| Timor-Leste | 50 to 54 | -5.12 (-25.97, 21.6) | -3.04 (-18.87, 15.87) | -2.94 (-18.8, 16.02) |
| Timor-Leste | 55 to 59 | -7.26 (-29.69, 22.32) | -4.88 (-22.1, 16.15) | -5.62 (-23.1, 15.83) |
| Timor-Leste | 60 to 64 | -8.12 (-32.65, 25.35) | -6.34 (-25.66, 18) | -7.38 (-27.31, 18.03) |
| Timor-Leste | 65 to 69 | -12.98 (-35.19, 16.85) | -7.06 (-26.91, 18.18) | -8.54 (-29.04, 17.89) |
| Timor-Leste | 70 to 74 | -15.04 (-37.49, 15.48) | -6.78 (-25.51, 16.65) | -8.38 (-27.79, 16.23) |
| Timor-Leste | 75 to 79 | -15.83 (-38.8, 15.78) | -6.68 (-23.89, 14.42) | -7.38 (-24.47, 13.58) |
| Timor-Leste | 80 to 84 | -14.86 (-37.95, 16.82) | -6.81 (-23.23, 13.11) | -7.18 (-23.53, 12.67) |
| Timor-Leste | 85 to 89 | -11.58 (-35.85, 21.86) | -6.3 (-26.63, 19.66) | -6.52 (-26.8, 19.38) |
| Togo | 25 to 29 | -12.88 (-53.97, 64.9) | -9.89 (-50.3, 63.38) | -2.94 (-24.83, 25.32) |
| Togo | 30 to 34 | -13.58 (-45.93, 38.13) | -5.31 (-26.94, 22.71) | -4.14 (-23, 19.33) |
| Togo | 35 to 39 | -12.19 (-41.38, 31.52) | -7.26 (-31.13, 24.89) | -5.22 (-26.85, 22.8) |
| Togo | 40 to 44 | -9.25 (-35.46, 27.59) | -9.02 (-34.43, 26.23) | -7.03 (-29.67, 22.89) |
| Togo | 45 to 49 | -5.71 (-29.36, 25.86) | -10.28 (-36.16, 26.09) | -8.16 (-33.23, 26.33) |
| Togo | 50 to 54 | -4.35 (-28.25, 27.52) | -9.22 (-32.75, 22.55) | -8.46 (-32.98, 25.04) |
| Togo | 55 to 59 | -2.01 (-27.34, 32.15) | -6.42 (-32.59, 29.92) | -5.34 (-32.87, 33.49) |
| Togo | 60 to 64 | -0.08 (-26.81, 36.42) | -2.51 (-31.89, 39.54) | -2.74 (-32.73, 40.6) |
| Togo | 65 to 69 | 1.45 (-26.37, 39.79) | 0.55 (-32.36, 49.47) | 0.12 (-32.54, 48.59) |
| Togo | 70 to 74 | 2.11 (-26.01, 40.92) | 2.37 (-33.86, 58.46) | 1.73 (-33.6, 55.87) |
| Togo | 75 to 79 | 1.39 (-27.34, 41.48) | 2.87 (-35.95, 65.22) | 2.13 (-35.37, 61.39) |
| Togo | 80 to 84 | 3.21 (-35.54, 65.24) | 1.61 (-41.65, 76.94) | 0.63 (-40.55, 70.32) |
| Togo | 85 to 89 | 3.99 (-46.48, 102.04) | -6.26 (-44.53, 58.41) | -6.36 (-40.67, 47.78) |
| Tokelau | 25 to 29 | 0.33 (-21.34, 27.97) | 0.04 (-21.57, 27.61) | 0.5 (-21.21, 28.19) |
| Tokelau | 30 to 34 | 0.82 (-16.69, 22) | 0.39 (-17.05, 21.49) | 1.26 (-16.33, 22.54) |
| Tokelau | 35 to 39 | -0.32 (-16.24, 18.62) | -0.39 (-16.29, 18.54) | -0.26 (-16.19, 18.69) |
| Tokelau | 40 to 44 | 0.84 (-14.78, 19.33) | 0.37 (-15.18, 18.77) | 1.32 (-14.37, 19.9) |
| Tokelau | 45 to 49 | -0.81 (-15.99, 17.11) | -0.97 (-16.13, 16.92) | -0.58 (-15.79, 17.39) |
| Tokelau | 50 to 54 | -0.95 (-15.99, 16.79) | -1.58 (-16.53, 16.05) | -0.41 (-15.54, 17.42) |
| Tokelau | 55 to 59 | -0.51 (-15.61, 17.3) | -1.12 (-16.13, 16.58) | 0.03 (-15.15, 17.93) |
| Tokelau | 60 to 64 | -0.39 (-15.52, 17.45) | -0.72 (-15.8, 17.06) | -0.16 (-15.32, 17.73) |
| Tokelau | 65 to 69 | 0.74 (-14.68, 18.94) | 1.37 (-14.15, 19.68) | 0.09 (-15.23, 18.17) |
| Tokelau | 70 to 74 | 0.68 (-14.92, 19.13) | 1.2 (-14.48, 19.75) | 0.17 (-15.35, 18.53) |
| Tokelau | 75 to 79 | -0.46 (-16.36, 18.45) | 0.3 (-15.72, 19.35) | -1.09 (-16.88, 17.71) |
| Tokelau | 80 to 84 | -1.6 (-18.69, 19.09) | -1.43 (-18.55, 19.29) | -1.6 (-18.69, 19.08) |
| Tokelau | 85 to 89 | -1.83 (-23.03, 25.22) | -1.77 (-22.99, 25.29) | -1.58 (-22.84, 25.54) |
| Tonga | 25 to 29 | 0.69 (-21.27, 28.77) | 0.49 (-21.22, 28.18) | 0.24 (-21.55, 28.09) |
| Tonga | 30 to 34 | -0.05 (-17.51, 21.12) | -0.02 (-17.39, 20.99) | -0.46 (-17.84, 20.61) |
| Tonga | 35 to 39 | -0.93 (-16.84, 18.03) | -0.89 (-16.71, 17.95) | -1.05 (-16.94, 17.87) |
| Tonga | 40 to 44 | -1.73 (-17.23, 16.66) | -1.61 (-16.86, 16.42) | -1.49 (-17.02, 16.94) |
| Tonga | 45 to 49 | -2.08 (-17.98, 16.91) | -1.97 (-16.98, 15.74) | -1.62 (-17.58, 17.42) |
| Tonga | 50 to 54 | -1.86 (-18.89, 18.75) | -1.67 (-16.6, 15.94) | -1.96 (-18.62, 18.1) |
| Tonga | 55 to 59 | -2.67 (-21.06, 20) | -0.96 (-16, 16.76) | -2.45 (-20.74, 20.07) |
| Tonga | 60 to 64 | -3.87 (-23.55, 20.86) | -0.28 (-15.43, 17.58) | -2.64 (-21.58, 20.88) |
| Tonga | 65 to 69 | -4.84 (-25.87, 22.17) | -0.23 (-15.5, 17.8) | -2.77 (-21.68, 20.7) |
| Tonga | 70 to 74 | -5.42 (-26.95, 22.46) | -0.69 (-16.08, 17.51) | -2.85 (-21.16, 19.71) |
| Tonga | 75 to 79 | -5.15 (-25.86, 21.34) | -1.26 (-17.03, 17.5) | -2.52 (-19.52, 18.07) |
| Tonga | 80 to 84 | -4.03 (-23.37, 20.19) | -1.86 (-18.91, 18.76) | -2.72 (-19.83, 18.05) |
| Tonga | 85 to 89 | -3.58 (-24.79, 23.61) | -2.47 (-23.54, 24.4) | -3.3 (-24.3, 23.52) |
| Trinidad and Tobago | 25 to 29 | 4.37 (-20.7, 37.36) | 4.41 (-31.99, 60.29) | 5.18 (-27.51, 52.6) |
| Trinidad and Tobago | 30 to 34 | 4.08 (-15.89, 28.8) | 4.06 (-23.65, 41.83) | 4.75 (-22.5, 41.59) |
| Trinidad and Tobago | 35 to 39 | 4.05 (-13.23, 24.77) | 4.01 (-18.14, 32.14) | 4.63 (-20.26, 37.28) |
| Trinidad and Tobago | 40 to 44 | 4.12 (-11.29, 22.22) | 3.65 (-15.65, 27.36) | 4.67 (-18.77, 34.88) |
| Trinidad and Tobago | 45 to 49 | 3.97 (-9.79, 19.83) | 3.63 (-13.3, 23.86) | 4.11 (-17.71, 31.71) |
| Trinidad and Tobago | 50 to 54 | 4.05 (-8.57, 18.42) | 3.94 (-11.44, 22) | 3.79 (-16.38, 28.83) |
| Trinidad and Tobago | 55 to 59 | 4.25 (-7.77, 17.83) | 4.33 (-10.33, 21.37) | 3.56 (-15.83, 27.42) |
| Trinidad and Tobago | 60 to 64 | 4.61 (-7.38, 18.15) | 4.93 (-9.78, 22.04) | 3.57 (-15.96, 27.64) |
| Trinidad and Tobago | 65 to 69 | 4.64 (-7.74, 18.69) | 5.06 (-10.11, 22.79) | 3.5 (-16.64, 28.51) |
| Trinidad and Tobago | 70 to 74 | 4.59 (-8.23, 19.21) | 4.96 (-10.93, 23.68) | 3.85 (-16.54, 29.22) |
| Trinidad and Tobago | 75 to 79 | 4.41 (-9.72, 20.75) | 4.66 (-12.95, 25.83) | 3.95 (-17.81, 31.46) |
| Trinidad and Tobago | 80 to 84 | 4.29 (-13.28, 25.43) | 4.23 (-18.2, 32.82) | 3.84 (-21.03, 36.55) |
| Trinidad and Tobago | 85 to 89 | 3.85 (-22.24, 38.69) | 3.24 (-29.14, 50.4) | 4.33 (-33.51, 63.73) |
| Tunisia | 25 to 29 | 3.84 (-14.69, 26.39) | 3.79 (-20.07, 34.77) | 3.79 (-23.17, 40.21) |
| Tunisia | 30 to 34 | 3.52 (-11.05, 20.48) | 3.45 (-16.2, 27.72) | 3.27 (-17.33, 29) |
| Tunisia | 35 to 39 | 3.22 (-9.27, 17.42) | 3.19 (-14.18, 24.08) | 3.01 (-14.08, 23.5) |
| Tunisia | 40 to 44 | 2.83 (-8.1, 15.06) | 2.81 (-12.68, 21.03) | 2.71 (-12.02, 19.89) |
| Tunisia | 45 to 49 | 2.42 (-7.31, 13.17) | 2.29 (-11.55, 18.29) | 2.45 (-10.64, 17.46) |
| Tunisia | 50 to 54 | 2.14 (-6.5, 11.57) | 1.91 (-10.35, 15.85) | 2.23 (-9.44, 15.39) |
| Tunisia | 55 to 59 | 1.95 (-5.71, 10.22) | 1.69 (-9.16, 13.84) | 2.05 (-8.38, 13.67) |
| Tunisia | 60 to 64 | 1.91 (-5.04, 9.38) | 1.7 (-8.09, 12.53) | 2.02 (-7.57, 12.61) |
| Tunisia | 65 to 69 | 2.06 (-4.62, 9.21) | 1.94 (-7.42, 12.25) | 2.18 (-7.12, 12.4) |
| Tunisia | 70 to 74 | 2.3 (-4.39, 9.44) | 2.31 (-7, 12.54) | 2.33 (-7.04, 12.65) |
| Tunisia | 75 to 79 | 2.63 (-4.47, 10.25) | 2.63 (-6.97, 13.23) | 2.7 (-7.57, 14.12) |
| Tunisia | 80 to 84 | 2.94 (-5.72, 12.39) | 3.11 (-8.3, 15.94) | 2.84 (-9.97, 17.48) |
| Tunisia | 85 to 89 | 2.87 (-10.49, 18.22) | 2.81 (-14.04, 22.96) | 2.78 (-16.84, 27.03) |
| Turkey | 25 to 29 | 2.33 (-5.34, 10.63) | 9.07 (-19.85, 48.44) | 7.93 (-22.16, 49.66) |
| Turkey | 30 to 34 | 2.15 (-3.81, 8.48) | 8.63 (-17.69, 43.38) | 7.47 (-19.62, 43.68) |
| Turkey | 35 to 39 | 1.99 (-2.92, 7.15) | 7.89 (-16.81, 39.93) | 7.08 (-18.21, 40.2) |
| Turkey | 40 to 44 | 2.05 (-2.06, 6.34) | 7.12 (-16, 36.6) | 6.41 (-17.19, 36.72) |
| Turkey | 45 to 49 | 2.21 (-1.17, 5.72) | 6.18 (-15.87, 34.01) | 5.86 (-17.18, 35.33) |
| Turkey | 50 to 54 | 1.92 (-0.88, 4.81) | 5.74 (-15.52, 32.35) | 5.46 (-16.27, 32.84) |
| Turkey | 55 to 59 | 1.58 (-0.79, 4) | 5.51 (-15.03, 31.01) | 5.2 (-15.31, 30.67) |
| Turkey | 60 to 64 | 1.16 (-0.91, 3.28) | 5.96 (-15.52, 32.9) | 4.77 (-15.5, 29.9) |
| Turkey | 65 to 69 | 1.06 (-0.91, 3.07) | 6.42 (-17.04, 36.52) | 5.03 (-16.26, 31.73) |
| Turkey | 70 to 74 | 1.3 (-0.75, 3.4) | 6.49 (-21.94, 45.27) | 4.97 (-19.3, 36.53) |
| Turkey | 75 to 79 | 1.83 (-0.55, 4.27) | 6.24 (-28.48, 57.8) | 4.46 (-23.01, 41.74) |
| Turkey | 80 to 84 | 1.98 (-0.79, 4.83) | -0.84 (-31.33, 43.19) | 5.23 (-33.38, 66.22) |
| Turkey | 85 to 89 | 1.7 (-1.86, 5.38) | -7.03 (-36.57, 36.27) | -2.59 (-33.82, 43.39) |
| Turkmenistan | 25 to 29 | 7.75 (-13.33, 33.96) | -1.94 (-23.12, 25.08) | -0.89 (-22.3, 26.42) |
| Turkmenistan | 30 to 34 | 7.67 (-11.43, 30.9) | -1.67 (-18.75, 19) | -0.28 (-17.6, 20.67) |
| Turkmenistan | 35 to 39 | 7.1 (-10.86, 28.67) | -1.33 (-17.09, 17.42) | 0.21 (-15.79, 19.26) |
| Turkmenistan | 40 to 44 | 6.56 (-10.23, 26.49) | -1.28 (-16.57, 16.82) | 0.23 (-15.3, 18.6) |
| Turkmenistan | 45 to 49 | 5.93 (-10.35, 25.15) | -1.71 (-16.75, 16.05) | -0.31 (-15.57, 17.71) |
| Turkmenistan | 50 to 54 | 5.44 (-10.02, 23.56) | -2.25 (-17.1, 15.26) | -1.06 (-16.09, 16.66) |
| Turkmenistan | 55 to 59 | 5.16 (-9.63, 22.38) | -2.55 (-17.35, 14.88) | -1.7 (-16.62, 15.89) |
| Turkmenistan | 60 to 64 | 5.12 (-9.92, 22.68) | -2.13 (-17, 15.39) | -1.78 (-16.7, 15.81) |
| Turkmenistan | 65 to 69 | 5.21 (-10.75, 24.02) | -0.88 (-16.05, 17.04) | -1.21 (-16.33, 16.64) |
| Turkmenistan | 70 to 74 | 5.19 (-13.32, 27.64) | -0.1 (-15.58, 18.21) | -0.81 (-16.18, 17.37) |
| Turkmenistan | 75 to 79 | 5.4 (-17.41, 34.52) | -1.09 (-16.88, 17.71) | -1.09 (-16.89, 17.7) |
| Turkmenistan | 80 to 84 | 6.34 (-26.18, 53.18) | -3.08 (-19.91, 17.29) | -2.01 (-19.02, 18.59) |
| Turkmenistan | 85 to 89 | 6.19 (-42.82, 97.21) | -5.06 (-25.57, 21.1) | -3.41 (-24.27, 23.2) |
| Tuvalu | 25 to 29 | -1.42 (-22.71, 25.74) | 2.53 (-7.43, 13.55) | 2.25 (-9.42, 15.43) |
| Tuvalu | 30 to 34 | -0.99 (-18.18, 19.83) | 2.51 (-5.54, 11.24) | 1.84 (-6.84, 11.33) |
| Tuvalu | 35 to 39 | -0.56 (-16.44, 18.34) | 2.54 (-4.27, 9.83) | 1.48 (-5.5, 8.98) |
| Tuvalu | 40 to 44 | -0.51 (-15.93, 17.72) | 2.67 (-3.16, 8.85) | 1.5 (-4.25, 7.6) |
| Tuvalu | 45 to 49 | -0.98 (-16.14, 16.91) | 2.78 (-2.15, 7.95) | 1.77 (-2.85, 6.62) |
| Tuvalu | 50 to 54 | -1.62 (-16.56, 16) | 2.44 (-1.7, 6.76) | 1.53 (-2.27, 5.48) |
| Tuvalu | 55 to 59 | -2.1 (-16.96, 15.42) | 1.93 (-1.65, 5.63) | 1.34 (-1.81, 4.59) |
| Tuvalu | 60 to 64 | -1.94 (-16.84, 15.62) | 1.42 (-1.74, 4.68) | 1 (-1.73, 3.81) |
| Tuvalu | 65 to 69 | -1.08 (-16.22, 16.79) | 1.11 (-1.91, 4.23) | 1.04 (-1.54, 3.69) |
| Tuvalu | 70 to 74 | -0.51 (-15.92, 17.73) | 0.69 (-2.42, 3.9) | 1.72 (-1, 4.52) |
| Tuvalu | 75 to 79 | -1.11 (-16.9, 17.68) | 0.4 (-3.09, 4.03) | 2.81 (-0.42, 6.15) |
| Tuvalu | 80 to 84 | -2.54 (-19.47, 17.94) | -0.11 (-4.11, 4.07) | 3.43 (-0.38, 7.39) |
| Tuvalu | 85 to 89 | -4.16 (-24.86, 22.25) | -0.42 (-5.69, 5.15) | 3.14 (-1.63, 8.14) |
| Uganda | 25 to 29 | 1.81 (-31.24, 50.75) | 1.35 (-39.08, 68.61) | -5.76 (-42.33, 53.99) |
| Uganda | 30 to 34 | 1.73 (-25.13, 38.22) | 1.45 (-31.96, 51.28) | -2.89 (-34.2, 43.3) |
| Uganda | 35 to 39 | 1.48 (-20.62, 29.73) | 1.16 (-25.91, 38.11) | -1.86 (-31.44, 40.48) |
| Uganda | 40 to 44 | 1.12 (-17.07, 23.29) | 0.89 (-21.53, 29.71) | -0.67 (-27.41, 35.93) |
| Uganda | 45 to 49 | 0.98 (-14.25, 18.93) | 0.58 (-18.63, 24.34) | 0.74 (-22.73, 31.35) |
| Uganda | 50 to 54 | 0.72 (-12.04, 15.32) | 0.57 (-15.59, 19.83) | 1.76 (-18.12, 26.46) |
| Uganda | 55 to 59 | 0.61 (-10.28, 12.81) | 0.46 (-13.46, 16.61) | 0.9 (-15.83, 20.94) |
| Uganda | 60 to 64 | 0.63 (-9.3, 11.64) | 0.39 (-12.36, 14.98) | 0.93 (-14.36, 18.94) |
| Uganda | 65 to 69 | 0.75 (-8.91, 11.44) | 0.29 (-12.29, 14.67) | 1.1 (-13.5, 18.15) |
| Uganda | 70 to 74 | 0.85 (-8.79, 11.52) | 0.5 (-12.26, 15.12) | 1.26 (-13.08, 17.97) |
| Uganda | 75 to 79 | 1.12 (-9.61, 13.13) | 0.92 (-13.99, 18.4) | 1.49 (-13.78, 19.46) |
| Uganda | 80 to 84 | 1.53 (-12.52, 17.85) | 0.92 (-18.63, 25.17) | 1.75 (-16.59, 24.12) |
| Uganda | 85 to 89 | 1.74 (-19.48, 28.55) | 1.36 (-30.62, 48.09) | 2.1 (-24.1, 37.35) |
| Ukraine | 25 to 29 | -2 (-29.02, 35.31) | -5.24 (-36.27, 40.9) | -8.66 (-42.46, 45) |
| Ukraine | 30 to 34 | -2.59 (-23.84, 24.59) | -5.84 (-30.23, 27.07) | -7.81 (-35.13, 31.01) |
| Ukraine | 35 to 39 | -2.52 (-22.3, 22.29) | -6.24 (-29.23, 24.23) | -6.26 (-31.78, 28.81) |
| Ukraine | 40 to 44 | -1.76 (-20.93, 22.05) | -6.51 (-29.06, 23.21) | -3.48 (-25.99, 25.86) |
| Ukraine | 45 to 49 | -0.87 (-18.77, 20.97) | -5.98 (-27.24, 21.51) | -3.31 (-22.95, 21.34) |
| Ukraine | 50 to 54 | 0.62 (-16, 20.53) | -5.14 (-24.83, 19.72) | -2.62 (-20.11, 18.7) |
| Ukraine | 55 to 59 | 2.51 (-14.61, 23.07) | -4.13 (-23.05, 19.43) | -1.49 (-17.95, 18.28) |
| Ukraine | 60 to 64 | 3.55 (-13.28, 23.66) | -4.18 (-22.62, 18.66) | 0.1 (-16.29, 19.71) |
| Ukraine | 65 to 69 | 5.38 (-16.85, 33.56) | -4.12 (-23.05, 19.46) | 1.29 (-15.76, 21.77) |
| Ukraine | 70 to 74 | 4.74 (-18.09, 33.93) | -5.48 (-24.96, 19.06) | 0.54 (-17.16, 22.02) |
| Ukraine | 75 to 79 | 1.39 (-18.08, 25.49) | -7.79 (-27.44, 17.18) | -2.9 (-19.95, 17.77) |
| Ukraine | 80 to 84 | -0.3 (-22.9, 28.94) | -7.69 (-29.59, 21.01) | -4.42 (-23.4, 19.27) |
| Ukraine | 85 to 89 | -1.65 (-26.79, 32.12) | -6.08 (-33.73, 33.1) | -4.47 (-28.64, 27.89) |
| United Arab Emirates | 25 to 29 | 2.41 (-12.8, 20.28) | 0.05 (-21.15, 26.95) | 4.6 (-18.13, 33.63) |
| United Arab Emirates | 30 to 34 | -0.57 (-11.68, 11.93) | -1.48 (-14.88, 14.02) | 1.28 (-17.8, 24.8) |
| United Arab Emirates | 35 to 39 | -1.35 (-11.02, 9.38) | -1.61 (-13.09, 11.4) | -1.23 (-18.35, 19.49) |
| United Arab Emirates | 40 to 44 | -1.57 (-10.82, 8.64) | -1.64 (-12.54, 10.62) | -1.98 (-18.48, 17.86) |
| United Arab Emirates | 45 to 49 | -2.38 (-11.32, 7.45) | -1.77 (-12.67, 10.5) | -3.51 (-18.98, 14.92) |
| United Arab Emirates | 50 to 54 | -2.56 (-11.77, 7.6) | -1.85 (-13.6, 11.49) | -3.58 (-18.64, 14.27) |
| United Arab Emirates | 55 to 59 | -1.33 (-11.27, 9.72) | -1.32 (-14.76, 14.23) | -1.63 (-16.81, 16.32) |
| United Arab Emirates | 60 to 64 | 0.88 (-10.07, 13.17) | -0.42 (-16.05, 18.11) | 1.75 (-13.89, 20.22) |
| United Arab Emirates | 65 to 69 | 3.41 (-9.2, 17.78) | 0.98 (-18.16, 24.61) | 6.05 (-11.12, 26.53) |
| United Arab Emirates | 70 to 74 | 6.4 (-9.12, 24.58) | 2.54 (-21.18, 33.4) | 10.91 (-10.07, 36.78) |
| United Arab Emirates | 75 to 79 | 7.96 (-13.91, 35.38) | 3.65 (-27.18, 47.52) | 12.5 (-15.3, 49.44) |
| United Arab Emirates | 80 to 84 | 8.03 (-18.88, 43.88) | 3.08 (-30.13, 52.07) | 13.09 (-23.22, 66.57) |
| United Arab Emirates | 85 to 89 | 6.68 (-32.43, 68.44) | 2.57 (-46.29, 95.86) | 11.94 (-40.93, 112.11) |
| United Kingdom | 25 to 29 | 3.33 (-13.26, 23.09) | 2.91 (-19.24, 31.14) | 4.3 (-19.43, 35.03) |
| United Kingdom | 30 to 34 | 3.59 (-10.42, 19.8) | 3.01 (-15.69, 25.87) | 4.7 (-15.64, 29.94) |
| United Kingdom | 35 to 39 | 3.83 (-9.27, 18.81) | 3.1 (-14.45, 24.24) | 4.84 (-14.13, 27.99) |
| United Kingdom | 40 to 44 | 3.79 (-8.43, 17.64) | 3.12 (-13.3, 22.64) | 4.59 (-12.97, 25.69) |
| United Kingdom | 45 to 49 | 3.77 (-6.9, 15.66) | 3.19 (-11.3, 20.06) | 4.44 (-10.71, 22.16) |
| United Kingdom | 50 to 54 | 3.16 (-5.87, 13.06) | 2.77 (-9.66, 16.91) | 3.62 (-9.1, 18.12) |
| United Kingdom | 55 to 59 | 2.29 (-5.19, 10.37) | 1.95 (-8.49, 13.57) | 2.69 (-7.77, 14.33) |
| United Kingdom | 60 to 64 | 1.49 (-4.52, 7.88) | 1.17 (-7.3, 10.41) | 1.88 (-6.48, 10.99) |
| United Kingdom | 65 to 69 | 0.99 (-3.76, 5.98) | 0.59 (-6.15, 7.81) | 1.47 (-5.11, 8.5) |
| United Kingdom | 70 to 74 | 0.61 (-3.12, 4.49) | -0.05 (-5.4, 5.61) | 1.19 (-3.95, 6.61) |
| United Kingdom | 75 to 79 | 1.5 (-1.66, 4.76) | 0.63 (-4, 5.48) | 2.14 (-2.14, 6.61) |
| United Kingdom | 80 to 84 | 2.15 (-0.67, 5.06) | 1.05 (-3.26, 5.55) | 2.81 (-0.9, 6.67) |
| United Kingdom | 85 to 89 | 2.9 (-0.59, 6.51) | 1.63 (-4.04, 7.64) | 3.56 (-0.82, 8.13) |
| United Republic of Tanzania | 25 to 29 | 2.51 (-10.58, 17.52) | 2.41 (-14.51, 22.68) | 2.75 (-16.75, 26.82) |
| United Republic of Tanzania | 30 to 34 | 2.37 (-10.35, 16.89) | 2.14 (-14.77, 22.4) | 2.77 (-15.5, 24.99) |
| United Republic of Tanzania | 35 to 39 | 2 (-10.33, 16.04) | 1.59 (-14.85, 21.2) | 2.52 (-15.28, 24.07) |
| United Republic of Tanzania | 40 to 44 | 1.63 (-9.84, 14.56) | 1.04 (-14.39, 19.25) | 2.44 (-14.12, 22.19) |
| United Republic of Tanzania | 45 to 49 | 1.33 (-8.92, 12.74) | 0.58 (-13.3, 16.67) | 2.3 (-12.37, 19.44) |
| United Republic of Tanzania | 50 to 54 | 1.16 (-7.59, 10.75) | 0.29 (-11.73, 13.95) | 2.12 (-10.3, 16.26) |
| United Republic of Tanzania | 55 to 59 | 1 (-6.37, 8.96) | 0.05 (-10.24, 11.52) | 1.97 (-8.35, 13.45) |
| United Republic of Tanzania | 60 to 64 | 0.82 (-5.72, 7.8) | -0.15 (-9.24, 9.85) | 1.81 (-7.38, 11.92) |
| United Republic of Tanzania | 65 to 69 | 0.72 (-5.45, 7.29) | -0.21 (-8.74, 9.13) | 1.67 (-7.07, 11.24) |
| United Republic of Tanzania | 70 to 74 | 0.79 (-5.39, 7.37) | -0.15 (-8.65, 9.15) | 1.75 (-7.07, 11.42) |
| United Republic of Tanzania | 75 to 79 | 0.98 (-5.97, 8.45) | -0.09 (-9.99, 10.89) | 1.92 (-7.72, 12.56) |
| United Republic of Tanzania | 80 to 84 | 1.3 (-7.65, 11.11) | 0.24 (-13.33, 15.94) | 2.11 (-9.61, 15.35) |
| United Republic of Tanzania | 85 to 89 | 1.51 (-12.55, 17.83) | 0.57 (-22.62, 30.72) | 2.27 (-15.23, 23.39) |
| United States of America | 25 to 29 | 5.14 (2.47, 7.88) | 4.42 (0.96, 8) | 6.01 (1.87, 10.32) |
| United States of America | 30 to 34 | 5.7 (3.75, 7.69) | 5 (2.44, 7.62) | 6.54 (3.55, 9.62) |
| United States of America | 35 to 39 | 5.97 (4.41, 7.55) | 5.37 (3.3, 7.48) | 6.66 (4.3, 9.07) |
| United States of America | 40 to 44 | 5.83 (4.54, 7.14) | 5.34 (3.62, 7.09) | 6.35 (4.4, 8.33) |
| United States of America | 45 to 49 | 5.41 (4.32, 6.5) | 5.13 (3.67, 6.6) | 5.69 (4.08, 7.33) |
| United States of America | 50 to 54 | 4.83 (3.89, 5.77) | 4.9 (3.61, 6.2) | 4.75 (3.38, 6.14) |
| United States of America | 55 to 59 | 4.21 (3.38, 5.05) | 4.68 (3.51, 5.86) | 3.75 (2.57, 4.95) |
| United States of America | 60 to 64 | 3.63 (2.89, 4.38) | 4.5 (3.43, 5.59) | 2.8 (1.76, 3.85) |
| United States of America | 65 to 69 | 3.29 (2.61, 3.98) | 4.44 (3.44, 5.46) | 2.22 (1.28, 3.16) |
| United States of America | 70 to 74 | 3.11 (2.48, 3.74) | 4.2 (3.26, 5.14) | 2.09 (1.23, 2.95) |
| United States of America | 75 to 79 | 3.11 (2.49, 3.72) | 3.85 (2.93, 4.77) | 2.36 (1.54, 3.2) |
| United States of America | 80 to 84 | 3.19 (2.5, 3.89) | 3.37 (2.32, 4.43) | 2.83 (1.9, 3.77) |
| United States of America | 85 to 89 | 3.14 (2.08, 4.2) | 2.79 (1.19, 4.43) | 3.08 (1.69, 4.5) |
| United States Virgin Islands | 25 to 29 | -5.49 (-51.51, 84.21) | 1.17 (-22.38, 31.85) | -0.25 (-22.95, 29.14) |
| United States Virgin Islands | 30 to 34 | -6.16 (-42.75, 53.81) | 1.22 (-18.22, 25.28) | 1.22 (-17.33, 23.93) |
| United States Virgin Islands | 35 to 39 | -4.87 (-40.19, 51.3) | -0.14 (-23.85, 30.97) | 1.73 (-17.47, 25.41) |
| United States Virgin Islands | 40 to 44 | -2.84 (-38.56, 53.64) | -3.57 (-31.51, 35.77) | 1.22 (-18.53, 25.76) |
| United States Virgin Islands | 45 to 49 | -0.53 (-35.87, 54.29) | -4.05 (-34.93, 41.48) | 0.29 (-24.78, 33.73) |
| United States Virgin Islands | 50 to 54 | 1.2 (-33.56, 54.14) | -3.05 (-35.88, 46.58) | -2.79 (-26.82, 29.13) |
| United States Virgin Islands | 55 to 59 | 1.86 (-31.72, 51.98) | -0.92 (-35.63, 52.51) | -7.17 (-33.65, 29.89) |
| United States Virgin Islands | 60 to 64 | 1.15 (-31.74, 49.91) | 0.33 (-35.69, 56.51) | -11.6 (-36.71, 23.46) |
| United States Virgin Islands | 65 to 69 | 0.56 (-32.88, 50.66) | 0.68 (-35.9, 58.12) | -12.19 (-39.08, 26.57) |
| United States Virgin Islands | 70 to 74 | 0.26 (-33.61, 51.41) | -1.56 (-38.07, 56.47) | -13.12 (-39.94, 25.66) |
| United States Virgin Islands | 75 to 79 | 1.03 (-35.8, 59) | -7.5 (-36.74, 35.26) | -12.01 (-38.95, 26.82) |
| United States Virgin Islands | 80 to 84 | 0.91 (-40.33, 70.66) | -11.31 (-38.17, 27.22) | -13.08 (-39.92, 25.77) |
| United States Virgin Islands | 85 to 89 | 1.38 (-49.68, 104.23) | -13.28 (-44.9, 36.49) | -12.9 (-43.72, 34.8) |
| Uruguay | 25 to 29 | 4.58 (-32.27, 61.48) | 5.3 (-37.65, 77.85) | -10.5 (-52.38, 68.22) |
| Uruguay | 30 to 34 | 3.97 (-26.43, 46.95) | 4.43 (-32.16, 60.75) | -8.04 (-42.08, 46.01) |
| Uruguay | 35 to 39 | 3.32 (-22.45, 37.65) | 3.56 (-28.42, 49.81) | -2.99 (-32.93, 40.32) |
| Uruguay | 40 to 44 | 2.8 (-18, 28.86) | 2.37 (-24.73, 39.23) | 0.69 (-23.72, 32.92) |
| Uruguay | 45 to 49 | 2.26 (-14.57, 22.39) | 1.79 (-20.15, 29.76) | 2.14 (-19.84, 30.15) |
| Uruguay | 50 to 54 | 2.14 (-11.21, 17.49) | 1.65 (-16.02, 23.03) | 3.17 (-15.28, 25.63) |
| Uruguay | 55 to 59 | 2.24 (-8.45, 14.19) | 1.74 (-12.48, 18.27) | 3.11 (-12.29, 21.2) |
| Uruguay | 60 to 64 | 2.14 (-6.69, 11.81) | 1.61 (-10.3, 15.1) | 2.66 (-10.01, 17.1) |
| Uruguay | 65 to 69 | 1.97 (-5.81, 10.39) | 1.73 (-8.97, 13.67) | 2.19 (-8.85, 14.56) |
| Uruguay | 70 to 74 | 1.91 (-5.09, 9.42) | 1.92 (-7.93, 12.82) | 1.94 (-7.79, 12.7) |
| Uruguay | 75 to 79 | 1.97 (-4.55, 8.93) | 2 (-7.59, 12.59) | 1.92 (-6.82, 11.47) |
| Uruguay | 80 to 84 | 2.05 (-4.52, 9.07) | 1.87 (-8.22, 13.08) | 2.13 (-6.32, 11.34) |
| Uruguay | 85 to 89 | 2.72 (-6.35, 12.66) | 2.54 (-11.89, 19.33) | 2.84 (-8.46, 15.54) |
| Uzbekistan | 25 to 29 | 5.04 (-8.24, 20.25) | 5.11 (-12.23, 25.88) | 5.12 (-14.09, 28.62) |
| Uzbekistan | 30 to 34 | 5.2 (-6.92, 18.9) | 5.21 (-11.13, 24.54) | 5.19 (-11.88, 25.55) |
| Uzbekistan | 35 to 39 | 4.83 (-6.89, 18.03) | 5.09 (-11.29, 24.49) | 4.47 (-11.39, 23.17) |
| Uzbekistan | 40 to 44 | 4.45 (-6.95, 17.24) | 5.06 (-11.46, 24.66) | 3.76 (-11.14, 21.17) |
| Uzbekistan | 45 to 49 | 3.63 (-7.48, 16.07) | 4.67 (-12.13, 24.67) | 2.79 (-11.49, 19.38) |
| Uzbekistan | 50 to 54 | 3.01 (-7.42, 14.61) | 4.33 (-11.89, 23.53) | 1.9 (-11.23, 16.98) |
| Uzbekistan | 55 to 59 | 2.27 (-7.43, 12.98) | 3.91 (-11.66, 22.23) | 1.01 (-11.05, 14.7) |
| Uzbekistan | 60 to 64 | 1.87 (-7.59, 12.3) | 3.78 (-11.84, 22.16) | 0.47 (-11.1, 13.54) |
| Uzbekistan | 65 to 69 | 1.9 (-7.81, 12.63) | 4.32 (-12.34, 24.14) | 0.37 (-11.38, 13.68) |
| Uzbekistan | 70 to 74 | 2.17 (-8.79, 14.44) | 5.4 (-15.21, 31.01) | 0.58 (-12.35, 15.41) |
| Uzbekistan | 75 to 79 | 2.41 (-10.48, 17.15) | 6.34 (-20.68, 42.56) | 0.7 (-13.67, 17.47) |
| Uzbekistan | 80 to 84 | 2.51 (-12.9, 20.65) | 7.22 (-26.46, 56.32) | 0.97 (-16.52, 22.13) |
| Uzbekistan | 85 to 89 | 2.77 (-20.25, 32.44) | 8.37 (-41.69, 101.43) | 1.26 (-23.99, 34.9) |
| Vanuatu | 25 to 29 | -2.4 (-24.44, 26.08) | -1.93 (-23.46, 25.67) | -1.64 (-23.35, 26.22) |
| Vanuatu | 30 to 34 | -3.53 (-22.54, 20.15) | -2.3 (-19.58, 18.7) | -2.33 (-19.61, 18.67) |
| Vanuatu | 35 to 39 | -4.83 (-26.76, 23.67) | -2.66 (-19.23, 17.31) | -3.02 (-19.74, 17.2) |
| Vanuatu | 40 to 44 | -6.52 (-29.77, 24.43) | -3.95 (-21.17, 17.02) | -4.74 (-22.54, 17.15) |
| Vanuatu | 45 to 49 | -7.91 (-34.31, 29.09) | -5.85 (-25.13, 18.39) | -7.15 (-27.36, 18.69) |
| Vanuatu | 50 to 54 | -9.27 (-35.72, 28.06) | -8.41 (-29.3, 18.64) | -9.76 (-31.47, 18.83) |
| Vanuatu | 55 to 59 | -6.26 (-36.69, 38.81) | -10.41 (-32.87, 19.58) | -11.76 (-34.18, 18.29) |
| Vanuatu | 60 to 64 | -6.62 (-39.15, 43.29) | -10.61 (-33.19, 19.6) | -12.11 (-34.82, 18.51) |
| Vanuatu | 65 to 69 | -5.99 (-40.9, 49.54) | -8.64 (-29.63, 18.6) | -10.08 (-31.61, 18.23) |
| Vanuatu | 70 to 74 | -6.52 (-40.49, 46.85) | -5.88 (-24.3, 17.03) | -7.25 (-26.68, 17.34) |
| Vanuatu | 75 to 79 | -6.81 (-36.99, 37.82) | -3.51 (-19.41, 15.53) | -4.96 (-22.17, 16.05) |
| Vanuatu | 80 to 84 | -7.6 (-33.13, 27.67) | -3.12 (-20.06, 17.4) | -4.14 (-21.06, 16.41) |
| Vanuatu | 85 to 89 | -4.86 (-26.45, 23.06) | -4.14 (-24.98, 22.48) | -5.19 (-25.86, 21.25) |
| Venezuela (Bolivarian Republic of) | 25 to 29 | 3.9 (-5.54, 14.28) | 3.98 (-8.82, 18.57) | 3.88 (-9.55, 19.3) |
| Venezuela (Bolivarian Republic of) | 30 to 34 | 3.34 (-4.56, 11.9) | 3.31 (-7.44, 15.32) | 3.37 (-7.92, 16.03) |
| Venezuela (Bolivarian Republic of) | 35 to 39 | 2.88 (-4.21, 10.49) | 2.75 (-6.97, 13.48) | 3.02 (-7.05, 14.18) |
| Venezuela (Bolivarian Republic of) | 40 to 44 | 2.53 (-3.72, 9.19) | 2.33 (-6.25, 11.68) | 2.75 (-6.15, 12.49) |
| Venezuela (Bolivarian Republic of) | 45 to 49 | 2.37 (-3, 8.05) | 2.11 (-5.24, 10.03) | 2.63 (-5.07, 10.96) |
| Venezuela (Bolivarian Republic of) | 50 to 54 | 2.26 (-2.4, 7.14) | 1.96 (-4.41, 8.75) | 2.54 (-4.15, 9.68) |
| Venezuela (Bolivarian Republic of) | 55 to 59 | 2.14 (-2.06, 6.52) | 1.84 (-3.92, 7.95) | 2.42 (-3.6, 8.82) |
| Venezuela (Bolivarian Republic of) | 60 to 64 | 2.02 (-1.93, 6.14) | 1.75 (-3.72, 7.53) | 2.3 (-3.34, 8.28) |
| Venezuela (Bolivarian Republic of) | 65 to 69 | 2.07 (-1.88, 6.18) | 1.71 (-3.73, 7.45) | 2.44 (-3.23, 8.43) |
| Venezuela (Bolivarian Republic of) | 70 to 74 | 2.28 (-1.93, 6.67) | 1.85 (-3.91, 7.96) | 2.69 (-3.36, 9.13) |
| Venezuela (Bolivarian Republic of) | 75 to 79 | 2.65 (-2.11, 7.65) | 2.11 (-4.41, 9.08) | 3.19 (-3.64, 10.51) |
| Venezuela (Bolivarian Republic of) | 80 to 84 | 3.01 (-2.92, 9.31) | 2.51 (-5.73, 11.47) | 3.48 (-4.86, 12.56) |
| Venezuela (Bolivarian Republic of) | 85 to 89 | 3.82 (-5.6, 14.17) | 3.47 (-9.61, 18.44) | 4.19 (-8.87, 19.11) |
| Viet Nam | 25 to 29 | 5.31 (-29.83, 58.05) | -4.6 (-39.93, 51.51) | 3.66 (-38.66, 75.19) |
| Viet Nam | 30 to 34 | 4.86 (-17.29, 32.96) | 0.93 (-29.02, 43.53) | 4.38 (-22.65, 40.86) |
| Viet Nam | 35 to 39 | 4.76 (-10.93, 23.22) | 3 (-18.59, 30.32) | 4.44 (-15.81, 29.54) |
| Viet Nam | 40 to 44 | 4.77 (-7.44, 18.59) | 4.04 (-13.01, 24.43) | 4.53 (-11.69, 23.73) |
| Viet Nam | 45 to 49 | 4.61 (-5.49, 15.79) | 4.55 (-9.73, 21.09) | 4.52 (-8.99, 20.04) |
| Viet Nam | 50 to 54 | 4.33 (-4.35, 13.78) | 4.28 (-8.29, 18.59) | 4.25 (-7.33, 17.27) |
| Viet Nam | 55 to 59 | 4.05 (-3.58, 12.28) | 3.94 (-7.25, 16.48) | 4.02 (-6.1, 15.23) |
| Viet Nam | 60 to 64 | 3.91 (-2.95, 11.26) | 3.79 (-6.37, 15.04) | 3.95 (-5.14, 13.92) |
| Viet Nam | 65 to 69 | 3.82 (-2.71, 10.79) | 3.7 (-5.99, 14.39) | 3.9 (-4.74, 13.32) |
| Viet Nam | 70 to 74 | 3.94 (-2.57, 10.88) | 3.76 (-6, 14.53) | 4.06 (-4.5, 13.38) |
| Viet Nam | 75 to 79 | 4.1 (-2.98, 11.7) | 3.79 (-7.04, 15.88) | 4.25 (-4.9, 14.28) |
| Viet Nam | 80 to 84 | 4.16 (-4.54, 13.65) | 3.66 (-10.19, 19.65) | 4.42 (-6.48, 16.6) |
| Viet Nam | 85 to 89 | 4.4 (-8.24, 18.78) | 3.59 (-17.5, 30.08) | 4.62 (-10.28, 21.98) |
| Yemen | 25 to 29 | -0.64 (-44.68, 78.45) | -13.27 (-54.15, 64.07) | -14.15 (-54.42, 61.69) |
| Yemen | 30 to 34 | -0.59 (-36.53, 55.7) | -12.14 (-44.98, 40.29) | -11.67 (-44.57, 40.76) |
| Yemen | 35 to 39 | -0.41 (-30.77, 43.27) | -7.98 (-38.93, 38.63) | -5.95 (-36.38, 39.04) |
| Yemen | 40 to 44 | -0.38 (-26.26, 34.58) | -4.86 (-34.57, 38.35) | -1.65 (-29.62, 37.45) |
| Yemen | 45 to 49 | -0.42 (-23.01, 28.79) | -1.86 (-29.43, 36.48) | -0.01 (-27.1, 37.14) |
| Yemen | 50 to 54 | -0.45 (-20.11, 24.05) | -0.88 (-26.31, 33.33) | 0.16 (-25.75, 35.11) |
| Yemen | 55 to 59 | -0.66 (-18.26, 20.74) | -1.1 (-24.75, 29.99) | -0.12 (-24.13, 31.5) |
| Yemen | 60 to 64 | -0.71 (-16.95, 18.7) | -1.27 (-23.06, 26.69) | -0.59 (-23.2, 28.68) |
| Yemen | 65 to 69 | -0.89 (-16.71, 17.94) | -1.31 (-22.15, 25.11) | -0.89 (-23.01, 27.6) |
| Yemen | 70 to 74 | -0.87 (-17.35, 18.91) | -1.49 (-23.18, 26.33) | -0.77 (-23.63, 28.92) |
| Yemen | 75 to 79 | -0.84 (-19.5, 22.16) | -1.19 (-27.12, 33.95) | 0.07 (-26.56, 36.37) |
| Yemen | 80 to 84 | -0.51 (-25.1, 32.15) | -1.16 (-34.03, 48.1) | 0.49 (-33.17, 51.13) |
| Yemen | 85 to 89 | -0.32 (-37.24, 58.31) | -1.51 (-48.57, 88.62) | 0.69 (-47.75, 94.03) |
| Zambia | 25 to 29 | 2.55 (-27.02, 44.1) | -0.43 (-35.67, 54.12) | 2.01 (-39.01, 70.6) |
| Zambia | 30 to 34 | 1.88 (-24.14, 36.81) | -2.89 (-31.46, 37.59) | 0.5 (-35.48, 56.55) |
| Zambia | 35 to 39 | 1.32 (-22.14, 31.83) | -1.99 (-29.24, 35.75) | -0.28 (-32.75, 47.87) |
| Zambia | 40 to 44 | 1.05 (-19.56, 26.94) | -0.61 (-25.8, 33.12) | -0.6 (-29.12, 39.38) |
| Zambia | 45 to 49 | 1.04 (-16.92, 22.89) | 0.07 (-22.63, 29.44) | -0.37 (-25.5, 33.24) |
| Zambia | 50 to 54 | 0.87 (-14.48, 18.97) | 0.75 (-19.61, 26.25) | -0.37 (-21.84, 27.01) |
| Zambia | 55 to 59 | 0.52 (-12.73, 15.78) | 1.27 (-16.77, 23.23) | -0.4 (-18.9, 22.31) |
| Zambia | 60 to 64 | 0.1 (-11.97, 13.83) | 0.22 (-15.89, 19.42) | -0.44 (-17.34, 19.91) |
| Zambia | 65 to 69 | -0.11 (-11.86, 13.21) | -0.02 (-15.67, 18.53) | -0.43 (-17.08, 19.56) |
| Zambia | 70 to 74 | -0.13 (-12.24, 13.66) | 0.04 (-15.88, 18.96) | -0.36 (-17.76, 20.72) |
| Zambia | 75 to 79 | 0.04 (-14.35, 16.83) | 0.23 (-18.74, 23.62) | -0.1 (-20.74, 25.9) |
| Zambia | 80 to 84 | 0.39 (-18.63, 23.87) | 0.6 (-24.65, 34.32) | 0.4 (-26.21, 36.61) |
| Zambia | 85 to 89 | 0.98 (-27.55, 40.76) | 1.05 (-37.16, 62.5) | 0.16 (-33.3, 50.41) |
| Zimbabwe | 25 to 29 | 3.23 (-17.3, 28.85) | 2.18 (-25.57, 40.26) | 5.48 (-24.41, 47.18) |
| Zimbabwe | 30 to 34 | 3.27 (-14.02, 24.02) | 2.4 (-20.66, 32.15) | 5.22 (-21.01, 40.14) |
| Zimbabwe | 35 to 39 | 3.16 (-12.71, 21.92) | 2.21 (-18.73, 28.55) | 4.91 (-19.51, 36.73) |
| Zimbabwe | 40 to 44 | 3.04 (-11.78, 20.34) | 2.15 (-17.22, 26.05) | 4.6 (-18.25, 33.83) |
| Zimbabwe | 45 to 49 | 2.67 (-11.42, 19) | 1.85 (-16.29, 23.92) | 4.25 (-17.27, 31.37) |
| Zimbabwe | 50 to 54 | 2.31 (-10.97, 17.58) | 1.38 (-15.8, 22.06) | 3.97 (-16.41, 29.32) |
| Zimbabwe | 55 to 59 | 1.91 (-10.59, 16.15) | 1.07 (-15.14, 20.38) | 3.64 (-15.7, 27.42) |
| Zimbabwe | 60 to 64 | 1.56 (-10.43, 15.17) | 0.74 (-14.73, 19.01) | 3.23 (-15.54, 26.17) |
| Zimbabwe | 65 to 69 | 1.26 (-10.53, 14.6) | 0.39 (-14.87, 18.37) | 2.9 (-15.55, 25.37) |
| Zimbabwe | 70 to 74 | 1.1 (-11.09, 14.98) | 0.2 (-15.87, 19.33) | 2.77 (-15.76, 25.37) |
| Zimbabwe | 75 to 79 | 1.04 (-12.54, 16.73) | 0 (-18.23, 22.28) | 2.36 (-17.31, 26.69) |
| Zimbabwe | 80 to 84 | 0.86 (-16.45, 21.76) | -0.12 (-24.31, 31.8) | 1.48 (-21.86, 31.78) |
| Zimbabwe | 85 to 89 | 0.74 (-25.57, 36.35) | -0.12 (-37.54, 59.72) | 0.77 (-32.46, 50.33) |

**Appendix 6.1** Age effects on the mortality of SSBs-related CKD across SDI

| Age | Global | Low SDI | Low-middle SDI | Middle SDI | High-middle SDI | High SDI |
| --- | --- | --- | --- | --- | --- | --- |
| 25 to 29 | 0.003 (0.003, 0.004) | 0.004 (0.001, 0.009) | 0.002 (0.001, 0.003) | 0.005 (0.004, 0.006) | 0.003 (0.002, 0.005) | 0.003 (0.002, 0.004) |
| 30 to 34 | 0.004 (0.003, 0.005) | 0.003 (0.001, 0.008) | 0.002 (0.001, 0.004) | 0.006 (0.005, 0.008) | 0.003 (0.002, 0.005) | 0.004 (0.003, 0.006) |
| 35 to 39 | 0.007 (0.006, 0.008) | 0.004 (0.002, 0.01) | 0.004 (0.002, 0.006) | 0.009 (0.007, 0.012) | 0.005 (0.003, 0.008) | 0.008 (0.006, 0.011) |
| 40 to 44 | 0.012 (0.011, 0.014) | 0.007 (0.004, 0.015) | 0.007 (0.005, 0.01) | 0.017 (0.014, 0.02) | 0.009 (0.006, 0.012) | 0.016 (0.013, 0.02) |
| 45 to 49 | 0.025 (0.023, 0.028) | 0.012 (0.007, 0.022) | 0.015 (0.011, 0.02) | 0.035 (0.03, 0.04) | 0.016 (0.012, 0.021) | 0.033 (0.028, 0.04) |
| 50 to 54 | 0.051 (0.047, 0.055) | 0.024 (0.015, 0.04) | 0.034 (0.026, 0.043) | 0.068 (0.06, 0.078) | 0.031 (0.025, 0.038) | 0.065 (0.056, 0.076) |
| 55 to 59 | 0.086 (0.08, 0.092) | 0.042 (0.028, 0.065) | 0.058 (0.047, 0.072) | 0.115 (0.103, 0.129) | 0.054 (0.045, 0.065) | 0.109 (0.096, 0.124) |
| 60 to 64 | 0.133 (0.125, 0.142) | 0.061 (0.041, 0.091) | 0.087 (0.072, 0.106) | 0.178 (0.161, 0.197) | 0.089 (0.075, 0.104) | 0.169 (0.15, 0.189) |
| 65 to 69 | 0.217 (0.204, 0.23) | 0.085 (0.058, 0.125) | 0.146 (0.122, 0.175) | 0.276 (0.25, 0.304) | 0.149 (0.128, 0.173) | 0.286 (0.257, 0.317) |
| 70 to 74 | 0.37 (0.349, 0.392) | 0.133 (0.091, 0.195) | 0.225 (0.188, 0.27) | 0.426 (0.386, 0.47) | 0.253 (0.219, 0.291) | 0.543 (0.493, 0.599) |
| 75 to 79 | 0.655 (0.611, 0.702) | 0.18 (0.113, 0.287) | 0.351 (0.279, 0.441) | 0.681 (0.603, 0.77) | 0.439 (0.371, 0.519) | 1.035 (0.922, 1.161) |
| 80 to 84 | 1.118 (1.038, 1.203) | 0.24 (0.141, 0.409) | 0.472 (0.363, 0.613) | 1.011 (0.881, 1.161) | 0.758 (0.636, 0.902) | 1.821 (1.617, 2.051) |
| 85 to 89 | 2.549 (2.359, 2.754) | 0.454 (0.248, 0.834) | 1.017 (0.761, 1.36) | 2.282 (1.961, 2.655) | 1.918 (1.603, 2.295) | 3.731 (3.301, 4.218) |

**Appendix 6.2** Age effects on the mortality of SSBs-related CKD across SDI in males

| Age | Global | Low SDI | Low-middle SDI | Middle SDI | High-middle SDI | High SDI |
| --- | --- | --- | --- | --- | --- | --- |
| 25 to 29 | 0.003 (0.003, 0.004) | 0.004 (0.001, 0.014) | 0.002 (0.001, 0.004) | 0.005 (0.003, 0.007) | 0.003 (0.001, 0.006) | 0.003 (0.002, 0.005) |
| 30 to 34 | 0.004 (0.003, 0.005) | 0.003 (0.001, 0.01) | 0.002 (0.001, 0.004) | 0.006 (0.004, 0.008) | 0.003 (0.002, 0.006) | 0.005 (0.003, 0.008) |
| 35 to 39 | 0.007 (0.005, 0.008) | 0.005 (0.002, 0.014) | 0.004 (0.002, 0.006) | 0.009 (0.006, 0.012) | 0.005 (0.003, 0.009) | 0.009 (0.006, 0.013) |
| 40 to 44 | 0.013 (0.011, 0.015) | 0.008 (0.003, 0.021) | 0.007 (0.004, 0.011) | 0.016 (0.012, 0.021) | 0.009 (0.006, 0.014) | 0.018 (0.013, 0.024) |
| 45 to 49 | 0.026 (0.022, 0.029) | 0.012 (0.005, 0.028) | 0.015 (0.01, 0.022) | 0.033 (0.027, 0.041) | 0.016 (0.011, 0.024) | 0.036 (0.028, 0.046) |
| 50 to 54 | 0.052 (0.047, 0.059) | 0.025 (0.013, 0.049) | 0.033 (0.024, 0.047) | 0.068 (0.057, 0.082) | 0.031 (0.023, 0.042) | 0.072 (0.059, 0.088) |
| 55 to 59 | 0.087 (0.079, 0.096) | 0.043 (0.024, 0.077) | 0.06 (0.045, 0.081) | 0.116 (0.099, 0.135) | 0.053 (0.04, 0.069) | 0.113 (0.094, 0.135) |
| 60 to 64 | 0.137 (0.125, 0.15) | 0.067 (0.039, 0.114) | 0.091 (0.069, 0.119) | 0.18 (0.156, 0.208) | 0.087 (0.068, 0.11) | 0.177 (0.151, 0.208) |
| 65 to 69 | 0.225 (0.207, 0.245) | 0.089 (0.052, 0.151) | 0.152 (0.118, 0.196) | 0.286 (0.249, 0.328) | 0.145 (0.116, 0.18) | 0.304 (0.263, 0.353) |
| 70 to 74 | 0.401 (0.37, 0.435) | 0.145 (0.086, 0.243) | 0.238 (0.184, 0.307) | 0.448 (0.389, 0.516) | 0.255 (0.206, 0.314) | 0.629 (0.551, 0.718) |
| 75 to 79 | 0.715 (0.648, 0.79) | 0.187 (0.098, 0.354) | 0.352 (0.255, 0.486) | 0.722 (0.606, 0.859) | 0.44 (0.344, 0.564) | 1.24 (1.053, 1.46) |
| 80 to 84 | 1.267 (1.139, 1.41) | 0.223 (0.105, 0.472) | 0.493 (0.34, 0.714) | 1.103 (0.904, 1.345) | 0.79 (0.61, 1.025) | 2.309 (1.946, 2.739) |
| 85 to 89 | 2.927 (2.613, 3.279) | 0.38 (0.156, 0.926) | 1.057 (0.698, 1.599) | 2.626 (2.107, 3.273) | 2.016 (1.539, 2.642) | 4.811 (4.021, 5.758) |

**Appendix 6.3** Age effects on the mortality of SSBs-related CKD across SDI in females

| Age | Global | Low SDI | Low-middle SDI | Middle SDI | High-middle SDI | High SDI |
| --- | --- | --- | --- | --- | --- | --- |
| 25 to 29 | 0.003 (0.002, 0.004) | 0.003 (0.001, 0.012) | 0.002 (0.001, 0.004) | 0.005 (0.004, 0.008) | 0.003 (0.001, 0.006) | 0.002 (0.001, 0.004) |
| 30 to 34 | 0.004 (0.003, 0.005) | 0.003 (0.001, 0.012) | 0.002 (0.001, 0.005) | 0.007 (0.005, 0.01) | 0.003 (0.002, 0.006) | 0.004 (0.002, 0.006) |
| 35 to 39 | 0.007 (0.006, 0.008) | 0.004 (0.001, 0.013) | 0.004 (0.002, 0.007) | 0.01 (0.007, 0.014) | 0.005 (0.003, 0.009) | 0.008 (0.005, 0.011) |
| 40 to 44 | 0.012 (0.01, 0.014) | 0.007 (0.002, 0.019) | 0.007 (0.004, 0.011) | 0.018 (0.014, 0.023) | 0.009 (0.005, 0.014) | 0.014 (0.01, 0.02) |
| 45 to 49 | 0.025 (0.022, 0.029) | 0.012 (0.005, 0.028) | 0.015 (0.01, 0.023) | 0.036 (0.029, 0.045) | 0.016 (0.011, 0.024) | 0.03 (0.023, 0.039) |
| 50 to 54 | 0.049 (0.043, 0.055) | 0.024 (0.012, 0.048) | 0.034 (0.024, 0.048) | 0.068 (0.057, 0.082) | 0.031 (0.023, 0.042) | 0.059 (0.047, 0.073) |
| 55 to 59 | 0.084 (0.076, 0.093) | 0.042 (0.023, 0.078) | 0.056 (0.042, 0.076) | 0.115 (0.098, 0.135) | 0.054 (0.042, 0.07) | 0.105 (0.088, 0.126) |
| 60 to 64 | 0.13 (0.118, 0.142) | 0.056 (0.031, 0.101) | 0.084 (0.064, 0.111) | 0.176 (0.152, 0.203) | 0.091 (0.072, 0.113) | 0.16 (0.136, 0.188) |
| 65 to 69 | 0.209 (0.192, 0.227) | 0.082 (0.047, 0.144) | 0.14 (0.108, 0.182) | 0.267 (0.232, 0.306) | 0.153 (0.125, 0.188) | 0.266 (0.229, 0.309) |
| 70 to 74 | 0.342 (0.315, 0.372) | 0.122 (0.07, 0.213) | 0.214 (0.165, 0.278) | 0.406 (0.353, 0.467) | 0.251 (0.207, 0.305) | 0.468 (0.406, 0.539) |
| 75 to 79 | 0.604 (0.548, 0.666) | 0.175 (0.088, 0.346) | 0.351 (0.254, 0.484) | 0.646 (0.544, 0.768) | 0.441 (0.351, 0.554) | 0.867 (0.736, 1.02) |
| 80 to 84 | 1.006 (0.908, 1.115) | 0.262 (0.123, 0.558) | 0.456 (0.315, 0.66) | 0.941 (0.777, 1.141) | 0.746 (0.589, 0.945) | 1.468 (1.242, 1.735) |
| 85 to 89 | 2.294 (2.063, 2.552) | 0.538 (0.231, 1.249) | 0.988 (0.657, 1.486) | 2.059 (1.67, 2.539) | 1.915 (1.505, 2.437) | 3.011 (2.54, 3.57) |

**Appendix 7.1** Period effects on the mortality of SSBs-related CKD across SDI

| Period | Global | Low SDI | Low-middle SDI | Middle SDI | High-middle SDI | High SDI |
| --- | --- | --- | --- | --- | --- | --- |
| 1992 to 1996 | 0.69 (0.64, 0.73) | 1.08 (0.73, 1.61) | 0.74 (0.59, 0.93) | 0.62 (0.55, 0.7) | 0.84 (0.72, 0.99) | 0.61 (0.55, 0.69) |
| 1997 to 2001 | 0.86 (0.81, 0.92) | 1.02 (0.69, 1.49) | 0.87 (0.71, 1.06) | 0.85 (0.76, 0.94) | 0.93 (0.81, 1.07) | 0.82 (0.75, 0.9) |
| 2002 to 2006 | 1 (1, 1) | 1 (1, 1) | 1 (1, 1) | 1 (1, 1) | 1 (1, 1) | 1 (1, 1) |
| 2007 to 2011 | 1.12 (1.06, 1.18) | 1.02 (0.72, 1.46) | 1.15 (0.96, 1.37) | 1.17 (1.07, 1.28) | 1.11 (0.98, 1.26) | 1.11 (1.03, 1.21) |
| 2012 to 2016 | 1.26 (1.2, 1.33) | 1.14 (0.81, 1.6) | 1.33 (1.13, 1.57) | 1.21 (1.11, 1.32) | 1.17 (1.03, 1.33) | 1.43 (1.32, 1.55) |
| 2017 to 2021 | 1.34 (1.27, 1.4) | 1.21 (0.87, 1.69) | 1.48 (1.26, 1.74) | 1.23 (1.13, 1.33) | 1.24 (1.09, 1.41) | 1.61 (1.48, 1.74) |

**Appendix 7.2** Period effects on the mortality of SSBs-related CKD across SDI in males

| Period | Global | Low SDI | Low-middle SDI | Middle SDI | High-middle SDI | High SDI |
| --- | --- | --- | --- | --- | --- | --- |
| 1992 to 1996 | 0.7 (0.64, 0.78) | 1.07 (0.62, 1.83) | 0.74 (0.54, 1.01) | 0.62 (0.52, 0.73) | 0.85 (0.68, 1.07) | 0.66 (0.56, 0.77) |
| 1997 to 2001 | 0.87 (0.8, 0.95) | 1.01 (0.6, 1.7) | 0.86 (0.65, 1.14) | 0.84 (0.72, 0.97) | 0.93 (0.76, 1.14) | 0.85 (0.74, 0.98) |
| 2002 to 2006 | 1 (1, 1) | 1 (1, 1) | 1 (1, 1) | 1 (1, 1) | 1 (1, 1) | 1 (1, 1) |
| 2007 to 2011 | 1.13 (1.05, 1.22) | 1.02 (0.63, 1.67) | 1.15 (0.9, 1.47) | 1.19 (1.05, 1.35) | 1.1 (0.91, 1.32) | 1.12 (0.99, 1.27) |
| 2012 to 2016 | 1.3 (1.21, 1.4) | 1.11 (0.7, 1.78) | 1.34 (1.06, 1.69) | 1.25 (1.11, 1.41) | 1.14 (0.95, 1.37) | 1.5 (1.33, 1.69) |
| 2017 to 2021 | 1.4 (1.3, 1.5) | 1.17 (0.74, 1.84) | 1.48 (1.18, 1.85) | 1.27 (1.13, 1.43) | 1.19 (0.99, 1.43) | 1.73 (1.54, 1.94) |

**Appendix 7.3** Period effects on the mortality of SSBs-related CKD across SDI in females

| Period | Global | Low SDI | Low-middle SDI | Middle SDI | High-middle SDI | High SDI |
| --- | --- | --- | --- | --- | --- | --- |
| 1992 to 1996 | 0.67 (0.61, 0.74) | 1.09 (0.61, 1.96) | 0.74 (0.54, 1.02) | 0.63 (0.53, 0.75) | 0.84 (0.68, 1.04) | 0.58 (0.5, 0.67) |
| 1997 to 2001 | 0.86 (0.79, 0.93) | 1.02 (0.58, 1.78) | 0.88 (0.66, 1.17) | 0.86 (0.75, 1) | 0.93 (0.77, 1.12) | 0.8 (0.71, 0.9) |
| 2002 to 2006 | 1 (1, 1) | 1 (1, 1) | 1 (1, 1) | 1 (1, 1) | 1 (1, 1) | 1 (1, 1) |
| 2007 to 2011 | 1.11 (1.03, 1.19) | 1.02 (0.61, 1.72) | 1.15 (0.89, 1.47) | 1.15 (1.02, 1.3) | 1.13 (0.95, 1.33) | 1.11 (0.99, 1.24) |
| 2012 to 2016 | 1.22 (1.14, 1.31) | 1.16 (0.71, 1.9) | 1.33 (1.05, 1.68) | 1.18 (1.04, 1.33) | 1.19 (1, 1.41) | 1.37 (1.22, 1.53) |
| 2017 to 2021 | 1.28 (1.19, 1.37) | 1.26 (0.78, 2.03) | 1.49 (1.19, 1.87) | 1.18 (1.05, 1.33) | 1.27 (1.07, 1.51) | 1.5 (1.34, 1.68) |

**Appendix 8.1** Cohort effects on the mortality of SSBs-related CKD across SDI

| Cohort | Global | Low SDI | Low-middle SDI | Middle SDI | High-middle SDI | High SDI |
| --- | --- | --- | --- | --- | --- | --- |
| 1905 to 1909 | 0.48 (0.41, 0.57) | 0.68 (0.14, 3.36) | 0.34 (0.15, 0.77) | 0.45 (0.3, 0.67) | 0.64 (0.46, 0.88) | 0.43 (0.34, 0.53) |
| 1910 to 1914 | 0.52 (0.47, 0.58) | 0.8 (0.3, 2.14) | 0.42 (0.25, 0.7) | 0.44 (0.34, 0.57) | 0.7 (0.55, 0.89) | 0.46 (0.39, 0.55) |
| 1915 to 1919 | 0.57 (0.51, 0.62) | 0.89 (0.44, 1.82) | 0.5 (0.34, 0.73) | 0.45 (0.37, 0.56) | 0.75 (0.6, 0.93) | 0.54 (0.47, 0.63) |
| 1920 to 1924 | 0.62 (0.57, 0.67) | 0.94 (0.53, 1.68) | 0.57 (0.42, 0.78) | 0.52 (0.44, 0.61) | 0.75 (0.61, 0.91) | 0.6 (0.52, 0.68) |
| 1925 to 1929 | 0.65 (0.6, 0.7) | 0.98 (0.59, 1.62) | 0.64 (0.5, 0.83) | 0.58 (0.51, 0.67) | 0.74 (0.62, 0.88) | 0.64 (0.56, 0.72) |
| 1930 to 1934 | 0.7 (0.65, 0.75) | 1 (0.64, 1.58) | 0.7 (0.55, 0.88) | 0.67 (0.59, 0.75) | 0.8 (0.68, 0.94) | 0.69 (0.61, 0.77) |
| 1935 to 1939 | 0.76 (0.71, 0.81) | 1 (0.65, 1.53) | 0.76 (0.61, 0.94) | 0.77 (0.69, 0.87) | 0.82 (0.7, 0.97) | 0.75 (0.67, 0.84) |
| 1940 to 1944 | 0.89 (0.83, 0.95) | 1 (0.65, 1.51) | 0.86 (0.71, 1.06) | 0.9 (0.81, 1) | 0.95 (0.81, 1.12) | 0.86 (0.77, 0.97) |
| 1945 to 1949 | 1 (1, 1) | 1 (1, 1) | 1 (1, 1) | 1 (1, 1) | 1 (1, 1) | 1 (1, 1) |
| 1950 to 1954 | 1.12 (1.05, 1.2) | 1.02 (0.65, 1.58) | 1.17 (0.95, 1.44) | 1.1 (0.98, 1.23) | 1.04 (0.87, 1.24) | 1.22 (1.08, 1.38) |
| 1955 to 1959 | 1.33 (1.23, 1.44) | 1.06 (0.65, 1.73) | 1.35 (1.07, 1.71) | 1.3 (1.15, 1.47) | 1.14 (0.93, 1.4) | 1.56 (1.35, 1.79) |
| 1960 to 1964 | 1.54 (1.41, 1.69) | 1.12 (0.65, 1.93) | 1.62 (1.25, 2.1) | 1.44 (1.25, 1.66) | 1.24 (0.97, 1.59) | 1.97 (1.68, 2.31) |
| 1965 to 1969 | 1.73 (1.55, 1.92) | 1.11 (0.59, 2.11) | 1.8 (1.31, 2.45) | 1.56 (1.32, 1.85) | 1.34 (1, 1.8) | 2.48 (2.05, 3.01) |
| 1970 to 1974 | 2.09 (1.84, 2.38) | 1.16 (0.55, 2.45) | 2.13 (1.45, 3.11) | 1.9 (1.55, 2.31) | 1.51 (1.05, 2.18) | 3.35 (2.66, 4.23) |
| 1975 to 1979 | 2.62 (2.24, 3.06) | 1.21 (0.51, 2.86) | 2.64 (1.68, 4.15) | 2.35 (1.85, 2.99) | 1.82 (1.16, 2.86) | 4.72 (3.56, 6.26) |
| 1980 to 1984 | 3.14 (2.61, 3.78) | 1.27 (0.48, 3.4) | 3.06 (1.8, 5.2) | 2.75 (2.07, 3.66) | 2.08 (1.2, 3.6) | 6.37 (4.54, 8.93) |
| 1985 to 1989 | 3.55 (2.84, 4.44) | 1.38 (0.45, 4.19) | 3.53 (1.87, 6.65) | 3.14 (2.23, 4.41) | 2.08 (1.04, 4.14) | 8.11 (5.3, 12.42) |
| 1990 to 1994 | 4.41 (3.28, 5.93) | 1.44 (0.38, 5.46) | 4.07 (1.81, 9.15) | 4.23 (2.72, 6.57) | 2.51 (0.96, 6.56) | 10.37 (5.74, 18.76) |

**Appendix 8.2** Cohort effects on the mortality of SSBs-related CKD across SDI in males

| Cohort | Global | Low SDI | Low-middle SDI | Middle SDI | High-middle SDI | High SDI |
| --- | --- | --- | --- | --- | --- | --- |
| 1905 to 1909 | 0.47 (0.37, 0.6) | 0.78 (0.07, 8.5) | 0.33 (0.1, 1.06) | 0.4 (0.22, 0.74) | 0.81 (0.49, 1.36) | 0.4 (0.28, 0.57) |
| 1910 to 1914 | 0.51 (0.43, 0.61) | 0.89 (0.22, 3.72) | 0.42 (0.2, 0.87) | 0.44 (0.3, 0.66) | 0.87 (0.6, 1.26) | 0.42 (0.33, 0.54) |
| 1915 to 1919 | 0.54 (0.47, 0.63) | 0.99 (0.37, 2.68) | 0.5 (0.29, 0.86) | 0.46 (0.34, 0.63) | 0.88 (0.63, 1.22) | 0.48 (0.38, 0.59) |
| 1920 to 1924 | 0.59 (0.52, 0.66) | 1.03 (0.47, 2.25) | 0.57 (0.37, 0.88) | 0.52 (0.41, 0.66) | 0.86 (0.65, 1.15) | 0.51 (0.42, 0.62) |
| 1925 to 1929 | 0.61 (0.55, 0.68) | 1.02 (0.51, 2.04) | 0.65 (0.45, 0.93) | 0.58 (0.47, 0.71) | 0.81 (0.63, 1.06) | 0.54 (0.46, 0.65) |
| 1930 to 1934 | 0.67 (0.61, 0.74) | 1.04 (0.56, 1.94) | 0.7 (0.51, 0.97) | 0.65 (0.55, 0.78) | 0.84 (0.66, 1.08) | 0.61 (0.51, 0.72) |
| 1935 to 1939 | 0.74 (0.68, 0.82) | 1.03 (0.57, 1.86) | 0.77 (0.57, 1.04) | 0.75 (0.64, 0.89) | 0.85 (0.67, 1.08) | 0.69 (0.59, 0.81) |
| 1940 to 1944 | 0.88 (0.8, 0.96) | 1.01 (0.57, 1.79) | 0.87 (0.65, 1.15) | 0.89 (0.76, 1.03) | 0.97 (0.76, 1.23) | 0.84 (0.72, 0.98) |
| 1945 to 1949 | 1 (1, 1) | 1 (1, 1) | 1 (1, 1) | 1 (1, 1) | 1 (1, 1) | 1 (1, 1) |
| 1950 to 1954 | 1.14 (1.03, 1.26) | 1.02 (0.55, 1.88) | 1.17 (0.87, 1.57) | 1.13 (0.96, 1.31) | 1.03 (0.8, 1.35) | 1.24 (1.05, 1.48) |
| 1955 to 1959 | 1.36 (1.22, 1.51) | 1.07 (0.55, 2.08) | 1.36 (0.98, 1.88) | 1.35 (1.14, 1.61) | 1.14 (0.85, 1.54) | 1.57 (1.29, 1.9) |
| 1960 to 1964 | 1.57 (1.38, 1.78) | 1.11 (0.52, 2.37) | 1.59 (1.1, 2.31) | 1.53 (1.25, 1.86) | 1.25 (0.88, 1.78) | 1.95 (1.56, 2.43) |
| 1965 to 1969 | 1.75 (1.51, 2.03) | 1.11 (0.46, 2.7) | 1.79 (1.15, 2.78) | 1.68 (1.33, 2.12) | 1.38 (0.91, 2.09) | 2.38 (1.83, 3.1) |
| 1970 to 1974 | 2.11 (1.77, 2.53) | 1.16 (0.41, 3.29) | 2.13 (1.24, 3.65) | 2.05 (1.55, 2.71) | 1.56 (0.93, 2.59) | 3.1 (2.25, 4.27) |
| 1975 to 1979 | 2.66 (2.14, 3.3) | 1.25 (0.38, 4.06) | 2.7 (1.42, 5.14) | 2.59 (1.85, 3.63) | 1.88 (1, 3.51) | 4.26 (2.9, 6.27) |
| 1980 to 1984 | 3.19 (2.47, 4.13) | 1.33 (0.34, 5.19) | 3.16 (1.48, 6.73) | 3.11 (2.09, 4.64) | 2.14 (1, 4.57) | 5.51 (3.46, 8.78) |
| 1985 to 1989 | 3.62 (2.65, 4.93) | 1.46 (0.31, 6.85) | 3.72 (1.52, 9.12) | 3.66 (2.28, 5.87) | 2.14 (0.83, 5.5) | 6.71 (3.75, 12.03) |
| 1990 to 1994 | 4.46 (2.97, 6.68) | 1.52 (0.26, 9.08) | 4.29 (1.39, 13.23) | 4.99 (2.73, 9.11) | 2.52 (0.68, 9.39) | 8.32 (3.69, 18.8) |

**Appendix 8.3** Cohort effects on the mortality of SSBs-related CKD across SDI in females

| Cohort | Global | Low SDI | Low-middle SDI | Middle SDI | High-middle SDI | High SDI |
| --- | --- | --- | --- | --- | --- | --- |
| 1905 to 1909 | 0.51 (0.42, 0.63) | 0.6 (0.07, 5.12) | 0.35 (0.11, 1.07) | 0.49 (0.3, 0.83) | 0.55 (0.36, 0.84) | 0.49 (0.37, 0.66) |
| 1910 to 1914 | 0.54 (0.47, 0.63) | 0.71 (0.18, 2.8) | 0.41 (0.2, 0.86) | 0.44 (0.31, 0.63) | 0.61 (0.44, 0.84) | 0.53 (0.43, 0.66) |
| 1915 to 1919 | 0.6 (0.52, 0.68) | 0.8 (0.29, 2.23) | 0.5 (0.29, 0.85) | 0.45 (0.34, 0.6) | 0.67 (0.5, 0.9) | 0.63 (0.51, 0.77) |
| 1920 to 1924 | 0.65 (0.58, 0.73) | 0.86 (0.37, 2) | 0.57 (0.37, 0.88) | 0.51 (0.41, 0.64) | 0.67 (0.52, 0.87) | 0.69 (0.58, 0.83) |
| 1925 to 1929 | 0.68 (0.61, 0.76) | 0.92 (0.44, 1.93) | 0.64 (0.44, 0.93) | 0.59 (0.48, 0.72) | 0.68 (0.53, 0.87) | 0.75 (0.63, 0.89) |
| 1930 to 1934 | 0.73 (0.67, 0.81) | 0.95 (0.49, 1.86) | 0.69 (0.5, 0.96) | 0.68 (0.57, 0.81) | 0.76 (0.61, 0.95) | 0.79 (0.67, 0.93) |
| 1935 to 1939 | 0.78 (0.71, 0.86) | 0.95 (0.51, 1.8) | 0.75 (0.55, 1.02) | 0.79 (0.67, 0.93) | 0.79 (0.64, 0.99) | 0.81 (0.69, 0.96) |
| 1940 to 1944 | 0.9 (0.82, 0.99) | 0.98 (0.53, 1.81) | 0.86 (0.65, 1.15) | 0.91 (0.78, 1.06) | 0.94 (0.75, 1.18) | 0.9 (0.76, 1.05) |
| 1945 to 1949 | 1 (1, 1) | 1 (1, 1) | 1 (1, 1) | 1 (1, 1) | 1 (1, 1) | 1 (1, 1) |
| 1950 to 1954 | 1.11 (1, 1.22) | 1.01 (0.53, 1.93) | 1.18 (0.87, 1.59) | 1.07 (0.91, 1.25) | 1.04 (0.81, 1.33) | 1.2 (1, 1.44) |
| 1955 to 1959 | 1.31 (1.17, 1.46) | 1.06 (0.52, 2.16) | 1.35 (0.97, 1.88) | 1.24 (1.04, 1.48) | 1.14 (0.85, 1.52) | 1.55 (1.27, 1.9) |
| 1960 to 1964 | 1.52 (1.34, 1.73) | 1.13 (0.52, 2.47) | 1.65 (1.14, 2.39) | 1.36 (1.11, 1.67) | 1.24 (0.88, 1.74) | 1.99 (1.58, 2.51) |
| 1965 to 1969 | 1.7 (1.46, 1.98) | 1.12 (0.44, 2.82) | 1.81 (1.16, 2.81) | 1.45 (1.14, 1.84) | 1.31 (0.86, 1.98) | 2.59 (1.95, 3.43) |
| 1970 to 1974 | 2.07 (1.72, 2.49) | 1.16 (0.39, 3.43) | 2.13 (1.25, 3.63) | 1.75 (1.32, 2.32) | 1.47 (0.87, 2.49) | 3.64 (2.6, 5.12) |
| 1975 to 1979 | 2.58 (2.06, 3.22) | 1.18 (0.34, 4.12) | 2.58 (1.36, 4.87) | 2.13 (1.51, 3) | 1.77 (0.92, 3.39) | 5.28 (3.5, 7.97) |
| 1980 to 1984 | 3.09 (2.37, 4.02) | 1.21 (0.29, 5.04) | 2.96 (1.4, 6.26) | 2.42 (1.61, 3.64) | 2.02 (0.91, 4.51) | 7.44 (4.55, 12.18) |
| 1985 to 1989 | 3.47 (2.51, 4.8) | 1.3 (0.26, 6.48) | 3.35 (1.36, 8.21) | 2.67 (1.64, 4.36) | 2.03 (0.74, 5.58) | 9.97 (5.33, 18.63) |
| 1990 to 1994 | 4.33 (2.81, 6.68) | 1.35 (0.18, 10.23) | 3.85 (1.2, 12.4) | 3.53 (1.85, 6.74) | 2.54 (0.62, 10.39) | 13.19 (5.56, 31.32) |

**Appendix 9** Age effects on the mortality of SSBs-related CKD across countries

| Country | Age | Age effects | | |
| --- | --- | --- | --- | --- |
|  |  | Both | Male | Female |
| Afghanistan | 25 to 29 | 0.002 (0, 1.701) | 0.009 (0, 657.621) | 0.002 (0, 7.647) |
| Afghanistan | 30 to 34 | 0.002 (0, 1.337) | 0.018 (0, 84.772) | 0.003 (0, 4.488) |
| Afghanistan | 35 to 39 | 0.005 (0, 0.943) | 0.031 (0, 41.413) | 0.005 (0, 3.315) |
| Afghanistan | 40 to 44 | 0.009 (0, 0.756) | 0.014 (0, 64.103) | 0.011 (0, 2.116) |
| Afghanistan | 45 to 49 | 0.017 (0, 0.641) | 0.017 (0, 22.272) | 0.022 (0, 1.511) |
| Afghanistan | 50 to 54 | 0.033 (0.002, 0.682) | 0.019 (0, 8.114) | 0.045 (0.001, 1.459) |
| Afghanistan | 55 to 59 | 0.049 (0.003, 0.779) | 0.023 (0, 5.066) | 0.066 (0.003, 1.679) |
| Afghanistan | 60 to 64 | 0.064 (0.005, 0.886) | 0.037 (0, 5.167) | 0.087 (0.004, 1.92) |
| Afghanistan | 65 to 69 | 0.083 (0.006, 1.095) | 0.051 (0, 6.118) | 0.111 (0.005, 2.369) |
| Afghanistan | 70 to 74 | 0.12 (0.009, 1.601) | 0.067 (0.001, 8.312) | 0.162 (0.007, 3.536) |
| Afghanistan | 75 to 79 | 0.165 (0.007, 3.899) | 0.097 (0, 25.116) | 0.215 (0.004, 10.559) |
| Afghanistan | 80 to 84 | 0.2 (0.004, 9.149) | 0.113 (0, 73.561) | 0.282 (0.002, 31.851) |
| Afghanistan | 85 to 89 | 0.314 (0.002, 41.8) | 0.203 (0, 422.403) | 0.414 (0.001, 278.439) |
| Albania | 25 to 29 | 0.001 (0, 115.141) | 0.03 (0, 1807.842) | 0.023 (0, 262.153) |
| Albania | 30 to 34 | 0.002 (0, 47.145) | 0.037 (0, 178.403) | 0.043 (0, 85.633) |
| Albania | 35 to 39 | 0.002 (0, 23.812) | 0.036 (0, 79.38) | 0.023 (0, 61.447) |
| Albania | 40 to 44 | 0.004 (0, 12.122) | 0.006 (0, 161.186) | 0.01 (0, 129.098) |
| Albania | 45 to 49 | 0.008 (0, 5.977) | 0.012 (0, 64.808) | 0.013 (0, 47.366) |
| Albania | 50 to 54 | 0.017 (0, 3.766) | 0.019 (0, 39.136) | 0.023 (0, 27.623) |
| Albania | 55 to 59 | 0.033 (0, 2.848) | 0.036 (0, 18.522) | 0.037 (0, 18.328) |
| Albania | 60 to 64 | 0.062 (0.001, 3.002) | 0.06 (0, 13.852) | 0.066 (0, 11.918) |
| Albania | 65 to 69 | 0.123 (0.005, 3.363) | 0.119 (0.001, 14.275) | 0.135 (0.002, 12.102) |
| Albania | 70 to 74 | 0.247 (0.011, 5.7) | 0.231 (0.002, 22.483) | 0.251 (0.004, 17.158) |
| Albania | 75 to 79 | 0.517 (0.012, 21.574) | 0.458 (0.002, 101.656) | 0.52 (0.003, 80.602) |
| Albania | 80 to 84 | 0.837 (0.015, 47.004) | 0.784 (0.002, 253.624) | 0.801 (0.003, 186.582) |
| Albania | 85 to 89 | 1.885 (0.026, 139.185) | 1.861 (0.004, 941.048) | 1.756 (0.005, 577.765) |
| Algeria | 25 to 29 | 0.003 (0, 0.041) | 0.004 (0, 0.193) | 0.003 (0, 0.088) |
| Algeria | 30 to 34 | 0.005 (0, 0.046) | 0.005 (0, 0.18) | 0.005 (0, 0.092) |
| Algeria | 35 to 39 | 0.008 (0.001, 0.063) | 0.006 (0, 0.188) | 0.01 (0.001, 0.126) |
| Algeria | 40 to 44 | 0.015 (0.003, 0.088) | 0.011 (0.001, 0.221) | 0.018 (0.002, 0.17) |
| Algeria | 45 to 49 | 0.028 (0.006, 0.131) | 0.02 (0.001, 0.265) | 0.036 (0.005, 0.244) |
| Algeria | 50 to 54 | 0.055 (0.014, 0.207) | 0.041 (0.004, 0.367) | 0.068 (0.013, 0.363) |
| Algeria | 55 to 59 | 0.085 (0.026, 0.278) | 0.063 (0.009, 0.443) | 0.105 (0.023, 0.476) |
| Algeria | 60 to 64 | 0.142 (0.05, 0.408) | 0.113 (0.021, 0.61) | 0.171 (0.044, 0.66) |
| Algeria | 65 to 69 | 0.211 (0.075, 0.593) | 0.155 (0.029, 0.831) | 0.269 (0.073, 0.996) |
| Algeria | 70 to 74 | 0.342 (0.124, 0.941) | 0.269 (0.053, 1.353) | 0.417 (0.113, 1.53) |
| Algeria | 75 to 79 | 0.662 (0.196, 2.24) | 0.503 (0.072, 3.514) | 0.825 (0.172, 3.96) |
| Algeria | 80 to 84 | 1.268 (0.342, 4.703) | 0.932 (0.117, 7.441) | 1.633 (0.301, 8.873) |
| Algeria | 85 to 89 | 5.464 (1.412, 21.147) | 2.946 (0.332, 26.11) | 8.912 (1.573, 50.511) |
| American Samoa | 25 to 29 | 10.786 (0.003, 35330.116) | 12.804 (0.013, 12857.401) | 13.414 (0.015, 11772.778) |
| American Samoa | 30 to 34 | 11.21 (0.004, 29501.629) | 13.494 (0.016, 11185.522) | 14.056 (0.02, 9930.436) |
| American Samoa | 35 to 39 | 11.077 (0.006, 21935.485) | 14.383 (0.021, 9769.319) | 15.02 (0.026, 8633.164) |
| American Samoa | 40 to 44 | 5.002 (0.002, 10226.969) | 14.255 (0.03, 6778.725) | 14.998 (0.037, 6136.286) |
| American Samoa | 45 to 49 | 2.625 (0.008, 822.493) | 11.118 (0.078, 1579.908) | 11.851 (0.091, 1543.739) |
| American Samoa | 50 to 54 | 3.161 (0.001, 7262.695) | 7.064 (0.06, 829.072) | 8.144 (0.072, 915.192) |
| American Samoa | 55 to 59 | 1.923 (0.001, 7268.22) | 3.741 (0.005, 2782.406) | 4.829 (0.008, 2886.82) |
| American Samoa | 60 to 64 | 2.175 (0.001, 4512.186) | 3.693 (0.002, 5935.373) | 4.802 (0.004, 5302.77) |
| American Samoa | 65 to 69 | 2.991 (0.002, 3755.378) | 2.553 (0.001, 8659.17) | 3.047 (0.002, 5264.272) |
| American Samoa | 70 to 74 | 3.998 (0.003, 5105.813) | 1.477 (0, 5731.671) | 1.616 (0.001, 3333.997) |
| American Samoa | 75 to 79 | 6.533 (0.001, 48552.784) | 1.489 (0, 8299.584) | 1.145 (0, 5060.121) |
| American Samoa | 80 to 84 | 10.692 (0.001, 173291.393) | 2.101 (0, 19132.6) | 1.651 (0, 10173.685) |
| American Samoa | 85 to 89 | 22.08 (0.001, 901743.44) | 4.189 (0, 68529.197) | 4.16 (0.001, 22806.822) |
| Andorra | 25 to 29 | 2.533 (0.014, 449.208) | 4.709 (0.032, 689.591) | 5.633 (0.036, 889.531) |
| Andorra | 30 to 34 | 2.24 (0.017, 292.985) | 4.143 (0.039, 443.897) | 5.02 (0.044, 574.413) |
| Andorra | 35 to 39 | 2.189 (0.021, 224.251) | 4.023 (0.048, 336.266) | 4.929 (0.055, 440.82) |
| Andorra | 40 to 44 | 2.195 (0.026, 185.706) | 3.995 (0.06, 266.088) | 4.935 (0.069, 354.492) |
| Andorra | 45 to 49 | 2.217 (0.045, 110.364) | 4.035 (0.098, 165.698) | 4.973 (0.114, 216.976) |
| Andorra | 50 to 54 | 2.289 (0.051, 103.187) | 4.221 (0.12, 148.643) | 5.051 (0.132, 193.11) |
| Andorra | 55 to 59 | 2.333 (0.053, 102.729) | 4.46 (0.137, 145.164) | 5.221 (0.143, 190.148) |
| Andorra | 60 to 64 | 2.419 (0.049, 118.466) | 4.903 (0.151, 159.586) | 5.538 (0.145, 211.171) |
| Andorra | 65 to 69 | 1.766 (0.005, 613.025) | 5.492 (0.154, 195.525) | 5.878 (0.132, 261.241) |
| Andorra | 70 to 74 | 0.262 (0, 2007.775) | 6.198 (0.14, 273.864) | 5.856 (0.052, 665.293) |
| Andorra | 75 to 79 | 0.435 (0, 1973.116) | 0.747 (0, 5806.227) | 0.712 (0, 6730.405) |
| Andorra | 80 to 84 | 0.814 (0, 2758.413) | 0.985 (0, 13731.183) | 0.871 (0, 14389.307) |
| Andorra | 85 to 89 | 1.489 (0, 6686.351) | 1.527 (0, 60695.533) | 1.862 (0, 11631.313) |
| Angola | 25 to 29 | 0.001 (0, 1.048) | 0.002 (0, 6.462) | 0.001 (0, 34.091) |
| Angola | 30 to 34 | 0.001 (0, 0.698) | 0.001 (0, 4.886) | 0.001 (0, 17.747) |
| Angola | 35 to 39 | 0.002 (0, 0.553) | 0.003 (0, 3.426) | 0.002 (0, 11.797) |
| Angola | 40 to 44 | 0.005 (0, 0.512) | 0.006 (0, 2.343) | 0.004 (0, 7.682) |
| Angola | 45 to 49 | 0.01 (0, 0.517) | 0.012 (0, 1.928) | 0.008 (0, 4.719) |
| Angola | 50 to 54 | 0.022 (0.001, 0.59) | 0.027 (0, 1.858) | 0.018 (0, 3.477) |
| Angola | 55 to 59 | 0.043 (0.002, 0.766) | 0.055 (0.001, 2.174) | 0.033 (0, 3.383) |
| Angola | 60 to 64 | 0.069 (0.005, 1.007) | 0.094 (0.003, 2.705) | 0.046 (0.001, 4.1) |
| Angola | 65 to 69 | 0.098 (0.007, 1.425) | 0.133 (0.005, 3.892) | 0.073 (0.001, 5.331) |
| Angola | 70 to 74 | 0.186 (0.013, 2.613) | 0.275 (0.01, 7.529) | 0.12 (0.002, 9.527) |
| Angola | 75 to 79 | 0.304 (0.011, 8.42) | 0.447 (0.007, 29.13) | 0.223 (0.001, 49.387) |
| Angola | 80 to 84 | 0.49 (0.01, 24.096) | 0.658 (0.004, 108.188) | 0.399 (0.001, 177.215) |
| Angola | 85 to 89 | 1.111 (0.012, 103.572) | 1.234 (0.002, 758.598) | 1.08 (0.001, 889.273) |
| Antigua and Barbuda | 25 to 29 | 5.905 (0.019, 1836.79) | 7.817 (0.047, 1299.584) | 6.045 (0.041, 883.52) |
| Antigua and Barbuda | 30 to 34 | 6.033 (0.023, 1558.461) | 7.73 (0.06, 993.692) | 5.776 (0.054, 616.891) |
| Antigua and Barbuda | 35 to 39 | 5.976 (0.029, 1249.19) | 7.61 (0.075, 775.354) | 5.648 (0.068, 469.989) |
| Antigua and Barbuda | 40 to 44 | 5.742 (0.037, 889.078) | 7.409 (0.095, 577.289) | 5.721 (0.086, 379.322) |
| Antigua and Barbuda | 45 to 49 | 5.218 (0.066, 410.972) | 7.326 (0.158, 338.583) | 5.874 (0.144, 240.448) |
| Antigua and Barbuda | 50 to 54 | 3.638 (0.066, 199.541) | 7.433 (0.198, 278.788) | 6.268 (0.18, 218.796) |
| Antigua and Barbuda | 55 to 59 | 3.124 (0.062, 156.917) | 7.358 (0.202, 268.098) | 6.746 (0.21, 216.53) |
| Antigua and Barbuda | 60 to 64 | 2.605 (0.042, 160.121) | 7.299 (0.193, 276.495) | 6.962 (0.217, 223.546) |
| Antigua and Barbuda | 65 to 69 | 2.531 (0.038, 168.884) | 8.101 (0.231, 284.37) | 7.494 (0.214, 262.004) |
| Antigua and Barbuda | 70 to 74 | 1.89 (0.009, 414.65) | 9.181 (0.222, 380.048) | 8.491 (0.207, 348.284) |
| Antigua and Barbuda | 75 to 79 | 1.445 (0.002, 970.201) | 11.286 (0.167, 763.456) | 10.115 (0.151, 675.389) |
| Antigua and Barbuda | 80 to 84 | 1.704 (0.001, 2153.937) | 17.394 (0.201, 1506.468) | 13.913 (0.163, 1185.614) |
| Antigua and Barbuda | 85 to 89 | 3.554 (0.003, 3971.416) | 35.288 (0.296, 4209.881) | 24.504 (0.209, 2871.416) |
| Argentina | 25 to 29 | 0.046 (0.016, 0.129) | 0.048 (0.011, 0.213) | 0.043 (0.01, 0.182) |
| Argentina | 30 to 34 | 0.044 (0.017, 0.116) | 0.047 (0.012, 0.186) | 0.041 (0.01, 0.16) |
| Argentina | 35 to 39 | 0.062 (0.027, 0.141) | 0.065 (0.02, 0.214) | 0.058 (0.018, 0.186) |
| Argentina | 40 to 44 | 0.095 (0.048, 0.19) | 0.1 (0.038, 0.265) | 0.091 (0.035, 0.239) |
| Argentina | 45 to 49 | 0.171 (0.101, 0.291) | 0.177 (0.083, 0.377) | 0.165 (0.079, 0.348) |
| Argentina | 50 to 54 | 0.329 (0.218, 0.497) | 0.345 (0.192, 0.62) | 0.313 (0.175, 0.561) |
| Argentina | 55 to 59 | 0.538 (0.38, 0.761) | 0.572 (0.35, 0.933) | 0.505 (0.309, 0.827) |
| Argentina | 60 to 64 | 0.833 (0.613, 1.131) | 0.86 (0.555, 1.333) | 0.806 (0.525, 1.239) |
| Argentina | 65 to 69 | 1.303 (0.98, 1.732) | 1.323 (0.876, 1.996) | 1.286 (0.866, 1.909) |
| Argentina | 70 to 74 | 1.979 (1.496, 2.618) | 2.031 (1.351, 3.054) | 1.946 (1.324, 2.86) |
| Argentina | 75 to 79 | 2.952 (2.144, 4.065) | 2.933 (1.841, 4.673) | 3.02 (1.942, 4.696) |
| Argentina | 80 to 84 | 4.499 (3.228, 6.271) | 4.403 (2.7, 7.179) | 4.727 (3, 7.45) |
| Argentina | 85 to 89 | 8.624 (6.118, 12.157) | 7.975 (4.749, 13.392) | 9.519 (5.979, 15.153) |
| Armenia | 25 to 29 | 0.176 (0, 512.096) | 0.449 (0, 443.566) | 0.326 (0, 250.717) |
| Armenia | 30 to 34 | 0.244 (0, 340.379) | 0.494 (0.001, 392.769) | 0.358 (0.001, 225.043) |
| Armenia | 35 to 39 | 0.148 (0, 132.066) | 0.498 (0.001, 285.094) | 0.367 (0.001, 176.108) |
| Armenia | 40 to 44 | 0.087 (0, 106.155) | 0.354 (0.001, 128.359) | 0.359 (0.001, 117.348) |
| Armenia | 45 to 49 | 0.045 (0, 10.948) | 0.214 (0.002, 24.654) | 0.185 (0.002, 21.192) |
| Armenia | 50 to 54 | 0.061 (0, 9.649) | 0.152 (0.002, 13.479) | 0.129 (0.001, 11.395) |
| Armenia | 55 to 59 | 0.048 (0, 37.663) | 0.129 (0.001, 13.191) | 0.104 (0.001, 10.558) |
| Armenia | 60 to 64 | 0.041 (0, 45.188) | 0.111 (0, 36.218) | 0.084 (0, 27.12) |
| Armenia | 65 to 69 | 0.039 (0, 42.23) | 0.056 (0, 116.915) | 0.042 (0, 76.652) |
| Armenia | 70 to 74 | 0.043 (0, 29.982) | 0.031 (0, 35.904) | 0.027 (0, 29.755) |
| Armenia | 75 to 79 | 0.046 (0, 44.769) | 0.043 (0, 51.43) | 0.037 (0, 32.042) |
| Armenia | 80 to 84 | 0.069 (0, 99.414) | 0.057 (0, 108.284) | 0.049 (0, 53.231) |
| Armenia | 85 to 89 | 0.149 (0, 422.537) | 0.122 (0, 355.324) | 0.08 (0, 217.747) |
| Australia | 25 to 29 | 0.001 (0, 0.387) | 0.001 (0, 4.087) | 0.001 (0, 7.535) |
| Australia | 30 to 34 | 0.001 (0, 0.277) | 0.001 (0, 2.234) | 0.001 (0, 2.709) |
| Australia | 35 to 39 | 0.001 (0, 0.144) | 0.001 (0, 0.988) | 0.001 (0, 0.915) |
| Australia | 40 to 44 | 0.003 (0, 0.101) | 0.002 (0, 0.556) | 0.003 (0, 0.5) |
| Australia | 45 to 49 | 0.006 (0, 0.098) | 0.006 (0, 0.295) | 0.006 (0, 0.317) |
| Australia | 50 to 54 | 0.015 (0.002, 0.117) | 0.014 (0.001, 0.282) | 0.016 (0.001, 0.266) |
| Australia | 55 to 59 | 0.026 (0.005, 0.145) | 0.025 (0.002, 0.298) | 0.028 (0.003, 0.292) |
[truncated: 676,878 more chars]
